# Chiral Polyacetylene‐PDMS‐Copolymer‐Gels as Enantiodifferentiating Alignment Media

**DOI:** 10.1002/mrc.70025

**Published:** 2025-08-18

**Authors:** Jochen Kornett, Michael Reggelin

**Affiliations:** ^1^ Technical University of Darmstadt Clemens Schöpf Institut for Organic Chemistry and Biochemistry Darmstadt Germany

**Keywords:** computational chemistry, enantiodifferentiation, NMR spectroscopy, polyacetylenes, residual dipolar couplings, strain induced alignment in a gel

## Abstract

Helically chiral polyaryl acetylenes based on 4‐ethinylated benzoic acid amid derivatives of different amino acids crosslinked by polydimethyl siloxane diynes form stable gels suited as new chiral SAG (*strain‐induced alignment in a gel*) media. Their robust synthesis is hardly error‐prone; they swell in CDCl_3_, dichloromethane, THF and toluene; the alignment strength is scalable; and their ability to differentiate the enantiomers of chiral analytes is unrivaled as compared with other SAG media.

## Graphical abstract

The crosslinking copolymerization of various amino acid derived 4‐ethinylbenzoic acid amides with different types of diynes delivered polymeric materials that can be used as chiral alignment (SAG) media for the measurement of anisotropic NMR data. Polysiloxane crosslinked gels are particularly suited in this respect. Moreover, they show exceptional enantiodifferentiating capabilities for a number of chiral analytes.

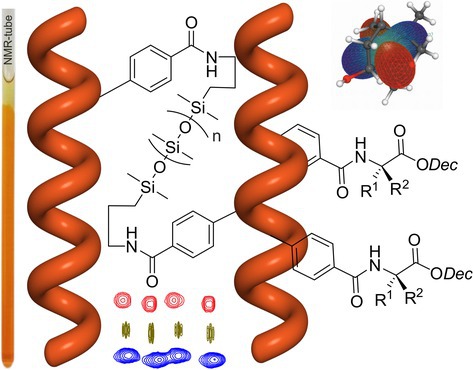

## 1 Introduction

The measurement of residual dipolar couplings (RDCs) in lyotropic liquid crystalline phases (LLCs) and anisotropically stretched polymer gels has established itself as a valuable analytical method with increasing importance in the analysis of small molecules [1]. It supplements conventional NMR structure‐elucidation methods and provides additional information related to the configuration and conformation of a given (chiral) analyte. RDCs obtained from chiral alignment media are of particular interest in this context, as they allow the differentiation of enantiomers of substances that do not crystallize or are incompatible with chiral derivatization agents.

Chiral LLCs of polypeptides have been studied and used as alignment media since decades [2]. Their commercial availability and easy handling makes them a popular choice among analysts. Moreover, derivatives with fascinating new properties are currently under investigation [3]. Other helically chiral polymers, such as poly guanidines [4], poly arylisocyanides, [5] and especially poly arylacetylenes [3, 6] with chiral amino acid‐derived side chains, have been developed as enantiodifferentiating alignment media in the last decade. In contrast to this vivid field of research, the development of chiral gels exploiting the “strain‐induced alignment in a gel” (SAG) proceeds very slow. To the best of our knowledge only three such media, compatible with organic solvents have been published to date: e^−^‐gelatin [7], chiral polyacrylamide gels [8], and crosslinked‐PBLG (cl‐PBLG) [9]. Although gels have some advantages over LLCs, such as their compatibility with a wide range of solvents and the possibility to easily adjust the degree of anisotropy (alignment strength), they possess their own range of problems, such as long swelling times and the high polymer concentration in the sample. The former problem has successfully been addressed by ingenious stretching or compressing devices [10], some of which are even commercially available these days [11]. The latter problem is of increasing importance with low‐concentrated samples where intense signals from the polymer backbone or trapped monomers and oligomers may hamper the extraction of anisotropic data. In addition, some of the already published chiral gels are very time consuming to synthesize [9] or require special equipment not easily accessible by other researchers [7].

**SCHEME 1:**
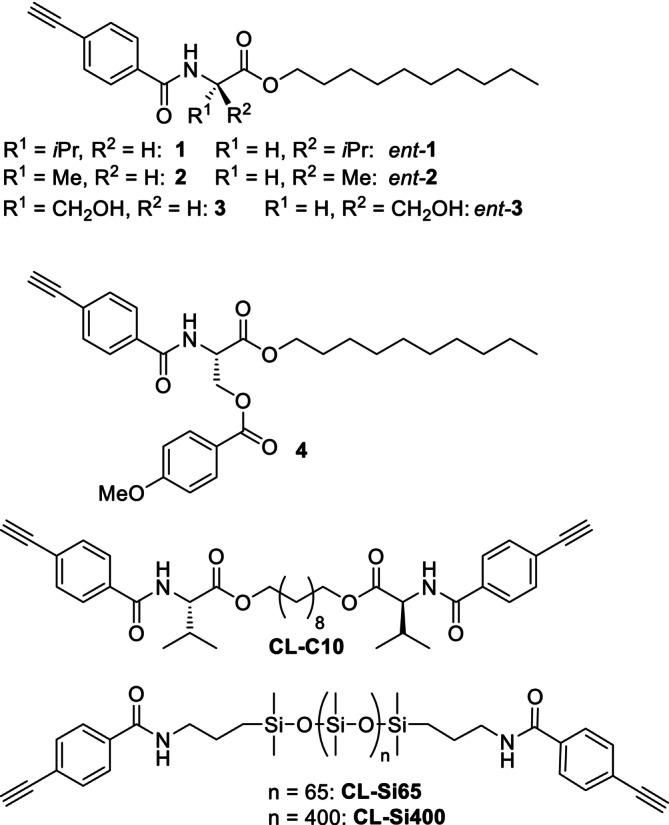
Structures of the monomers **1**–**4** and the crosslinkers **CL‐C10**, **CL‐Si65**, and **CL‐Si400**. Details of their polymerization to **p1**–**p4** are given in the SI.

Encouraged by the excellent enantiodifferentiating properties of the polyphenylacetylenes (PPAs) and inspired by the opportunities related to the application of the stretching/compressing devices mentioned above, we looked for ways to combine the positive characteristics of the LLC with the SAG phases. Initial experiments with polyacetylenes embedded in cross‐linked polystyrene were met with only moderate success. Although these materials swelled in chloroform and oriented analytes strongly enough to measure RDCs, the enantiodifferentiating capability of the medium was almost completely lost [12]. From these results, we concluded that the preservation of the (rather sensitive) [13] helical structure of the polyacetylene should be given special attention. To simplify the gel preparation, we combined the polymerization of the chiral monomers with the crosslinking step using symmetric bis‐acetylenic structures as crosslinking agents (Scheme 1).

Four different monomers **1**–**4**, whose suitability as LLC phase forming polymers was known [3, 6, 12, 14], were chosen to explore possibilities to fabricate gels and to compare their performance with the corresponding LLC phases. As far as the linkers are concerned, we have opted for two different types. The diyne **CL‐C10** can be regarded as a C_2_‐symmetric dimer of monomer **1** with a C10 spacer between the two *N*‐acylated valine moieties. This one was designed to maximize the structural similarity between linker and monomer. With the poly siloxane‐based diynes (**CL‐Si65** and **CL‐Si400**), we pursued the goal to prepare a universal linker without stereogenic centers and no structural resemblance to the monomers (random coil vs. helical backbone). In addition, we expected the dimethylsiloxane (PDMS) sections of different lengths to have a positive effect on the mechanical properties of the gels as well as the structural integrity of the polyacetylene helices. The main purpose of all linkers was to spatially separate the growing polymer chains (**p1**–**p4**), thus minimizing detrimental effects on their helical conformations.

The C‐10 crosslinker **CL‐C10** can easily be synthesized by a route analogous to that of the valine monomer **1** [3d] starting from 1,10‐decanediol and *l*‐valine as described in the Supporting information S1 (1.2.7). The poly siloxane linkers **CL‐Si65** and **CL‐Si400** are simple acylation products of commercially available poly siloxanes with aminopropyl end groups. (see Supporting information S1 1.2.8). The preparation of the gels was carried out in hydrophobized glass tubes with an inner diameter 6 mm. In these tubes, a solution of both the monomers **p1**–**p4** and the crosslinker in dry, degassed THF was mixed with a solution of a triphenyl vinyl based rhodium catalyst [3, 15] under Schlenk conditions. This mixture formed a stable gel in a matter of minutes. The gels were left to dry in their tubes at room temperature for 5 days, after which they can easily be removed. To get rid of the catalyst and most of the remaining monomer or oligomers from the gels, they were left to swell first in dichloromethane, then in THF after which they were dried again. For a complete removal of unwanted ingredients, the polymer sticks can be extracted using a Soxhlet‐Extractor.

## 2 Results and Discussion

We started our investigation with the **p1**/**CL‐C10** system. Gels with varying amounts of crosslinker, as well as gels obtained from monomer solutions of different concentrations were investigated (Table S2).

**FIGURE 1:**
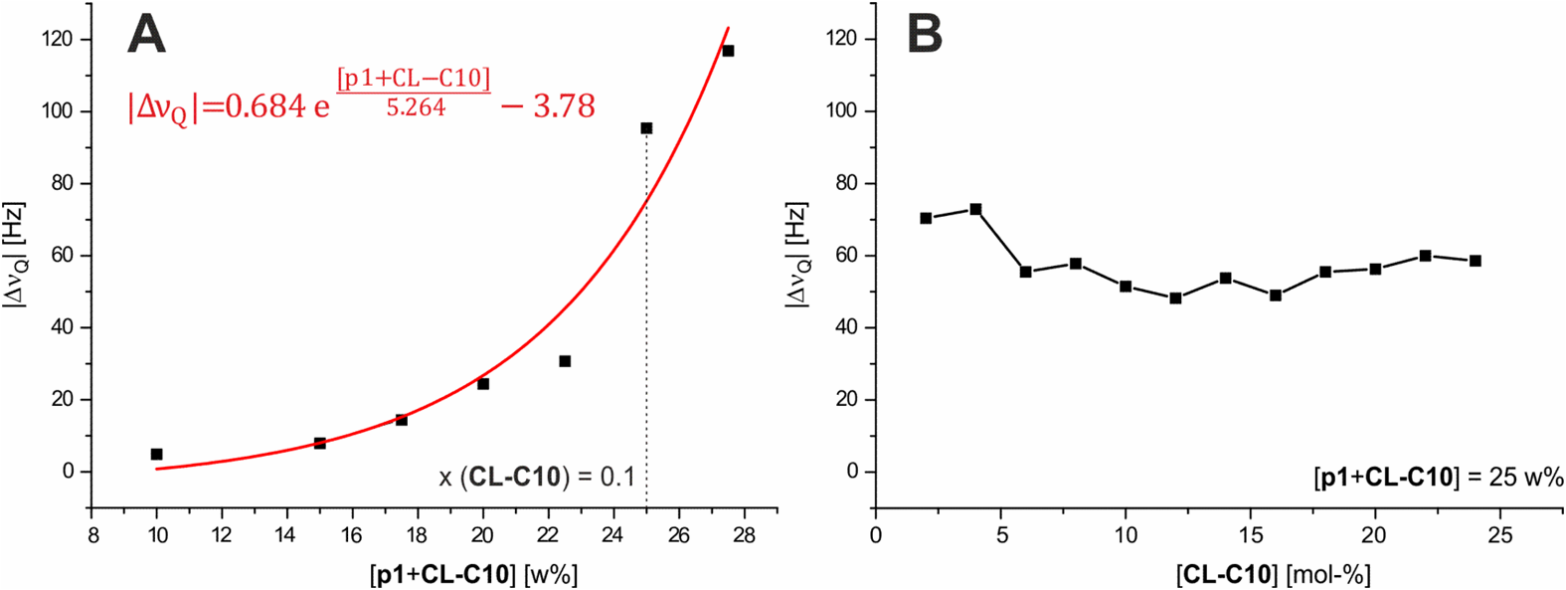
(A) Quadrupolar splitting of CDCl_3_ in **p1**/**CL‐C10** polymer gels as a function of the combined concentration of all polymerizable ingredients at constant crosslinker concentration. The ^2^H‐spectra used can be found in Figure S3. (B) Quadrupolar splitting of CDCl_3_ in **p1**/**CL‐C10** polymer gels as a function of the crosslinker concentration at constant concentration of all polymerizable ingredients.

All gels showed good swelling behavior in a range of weakly polar organic solvents such as chloroform, dichloromethane, and THF as well as toluene as representative of an unpolar solvent. To judge their usability as an alignment medium, the gel sticks were cut to a length of approximately 2 cm, placed inside standard 5‐mm NMR tubes, and left to swell with a solution of an analyte in dry deuterated chloroform. The swelling process of the sticks was observed by regularly measuring ^2^H‐NMR images [5, 16]. When the quadrupolar splitting Δν_Q_ of the solvents deuterium signal was uniform across the whole gel, it was ready to use. Although all sticks eventually showed homogenous quadrupolar splitting along their whole length, the time it took to achieve this state and the strength of the splitting varied strongly with their composition. Because the concentration of the monomer solution directly affects the diameter of the resulting dried stick, it can be used to adjust the final degree of anisotropy of the swollen gel, as shown in Figure 1.

Contrasting previously published literature on SAG media [17], there was no strong correlation between the amount of crosslinker used and the achieved quadrupolar splitting of the gels (Figure 1B). This might be an indication that helical polymers, as PPAs obtained with the catalyst system used here [18], behave differently in this regard than random coil polymers. The gels obtained from solutions of ≤ 27.5 w% of polymerizable components (monomer plus crosslinker), as well as ≤ 10 mol% of crosslinker, typically show homogenous splitting after 14 days of swelling. A reduction in the concentration of both components only leads to a slightly reduced swelling time, whereas an increase above the specified values leads to a significant extension of this time. In addition, the line width for the analytes increases significantly, which is why these compositions were avoided. Therefore, we defined a “standard composition” as follows: 25%–27.5% polymerizable ingredients containing 10 mol% of crosslinker.

### 2.1 Orientational Properties of the Gels

**FIGURE 2:**
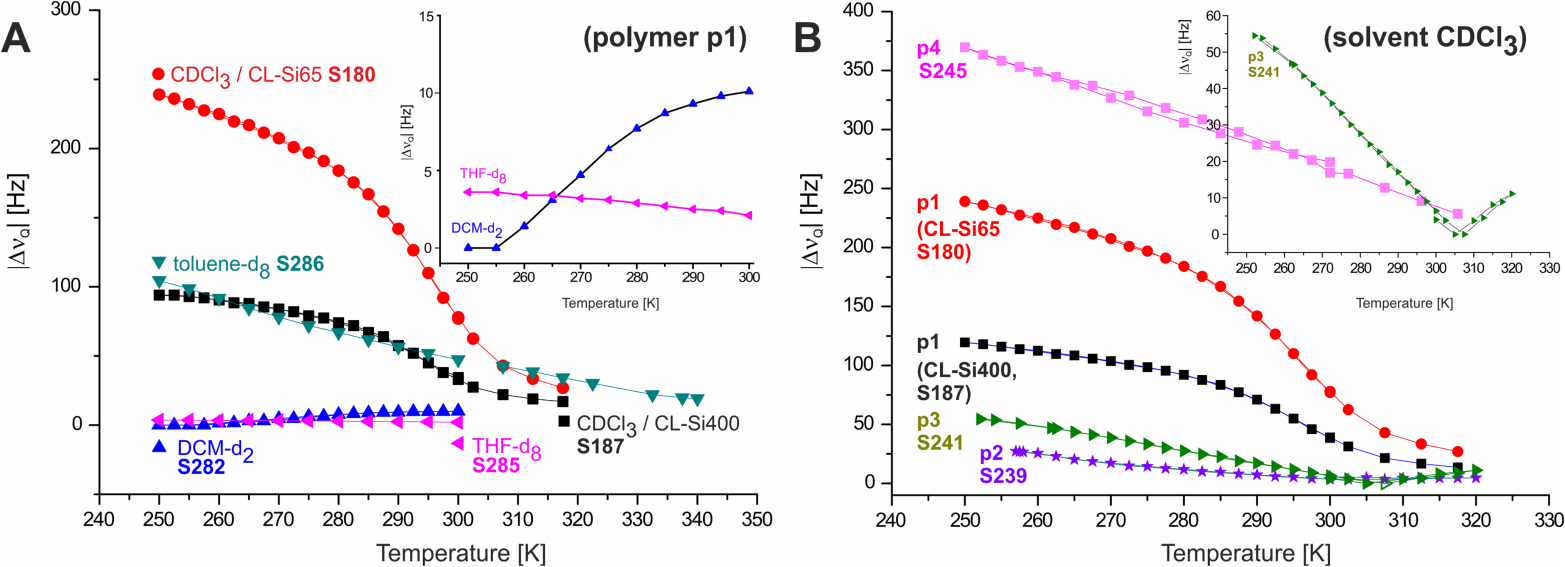
(A) Temperature dependence of the quadrupolar splitting in different deuterated solvents measured in **p1**/**CL‐Si65** gels of standard composition. (B) Temperature dependence of the quadrupolar splitting of gels containing different polymers and crosslinkers measured in CDCl_3_. **S#** denotes the gel sticks as defined in Table S2. Typical swelling times at ambient temperature for the different solvents are: Toluene‐d_8_: 22d; CDCl_3_: 14d; DCM‐d_2_: 7d; THF‐d_8_: < 60 h.

It is expected that the quadrupolar splitting of a deuterated solvent in an anisotropic phase is temperature dependent. In fact, we have observed this behavior more or less pronounced in all cases during the investigation of a large number of LLC phases [6, 12, 14, 19]. If, and only if (!), the phase composition remains constant, then this change in the quadrupolar splitting of the solvent correlates with the strengths of the orientation of the analyte dissolved in the LLC phase. When changing the solvent or the polymer, this correlation is lost. There is no (obvious) relation between the quadrupolar splitting of the solvent and the alignment strength of an analyte when changing either the solvent or the polymer or even both. This is also true for the gels under investigation here. Usually, a solvent change alone already has dramatic effects on the size and the temperature dependence of the quadrupolar splitting (Figure 2A). As is often observed, the orientation of chloroform is mostly much more pronounced than that of the closely related solvent dichloromethane (DCM). Another interesting fact is the very small quadrupolar splitting with THF, although this solvent swells the polymer sticks most rapidly.

Although chloroform‐*d*_*1*_, THF‐*d*_*8*_, and toluene‐*d*_*8*_ show the usual decrease of the quadrupolar splitting with increasing temperature, the corresponding dependency for dichloromethane‐*d*_*2*_ (DCM) is different. No splitting is observed between 250 and 257.5 K, implying that the solvent is in an isotropic environment! Above 257.5 K, the system enters an anisotropic state with a slowly growing splitting, finally reaching a plateau at around 300 K (due to the low boiling point of DCM [315 K] no further increase of the temperature was risked). The very small splittings in THF as compared with chloroform follow the trends we already observed with the corresponding LLC‐phases. **p1** at 25% in THF is isotropic above 265 K, and the quadrupolar splitting at 250 K is as small as 10 Hz for the high‐field signal. The same polymer at only 18% and 300 K displays a 160‐Hz quadrupolar splitting of CDCl_3_. A similar behavior is observed with **p4** in these two solvents [14, 19]. A change of the amino acid side chain in the polymer has at least as dramatic an effect as a change in the solvent (Figure 2B).

**FIGURE 3:**
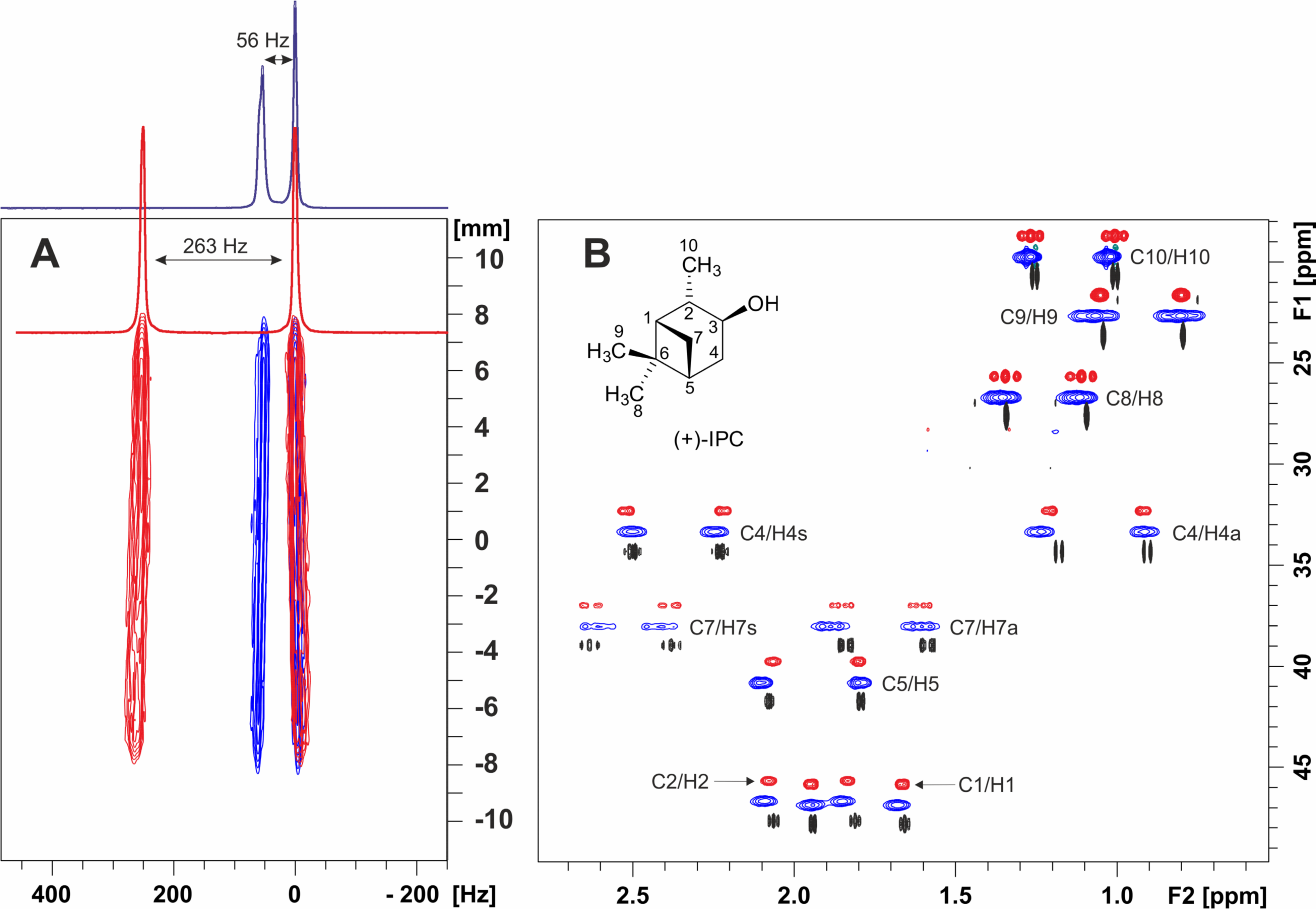
(A) Quadrupolar splitting and ^2^H‐images of **p1**/**CL‐Si65** (**S179**; blue contours) and **p4**/**CL‐Si65** (**S245**; red contours). (B) Superposition of three CLIP‐HSQC‐spectra of (+)‐IPC. Black contours: isotropic; blue and red contours as defined before. Only the isotropic spectrum is calibrated.

The gels loaded with **p4** (MetSerPhenol; MSP) [20] exhibit by far the largest quadrupole splitting over the entire temperature range from 250 to 320 K. The coupling changes strictly linearly between 370 and 223 Hz with a slope of −2.1 Hz/K. Apart from very small deviations, these temperature‐induced changes were completely reversible, which was tested by comparing the measurements with falling and rising temperatures. As already mentioned above, these very large splittings of the *solvent* do not mean that the orientation of a given *analyte* is too strong to measure RDCs in these **p4**‐containing gels! Although the quadrupolar splitting at 300 K was 56 Hz with a **p1**/**CL‐Si65**‐gel and 263 Hz with a **p4**/**CL‐Si65**‐gel, the corresponding ^1^ *D*_CH_‐couplings for isopinocampheol (**IPC**) are very similar, and the spectral quality of the CLIP‐HSQCs involving the latter gel was excellent (Figure 3).

Very small values for all temperatures are measured for the alanine and serine containing gels **p2** and **p3**, respectively. Moreover, the latter shows a zero crossing at around 308 K (open triangle in the green curve and insert in Figure 2B). Intermediate levels of the quadrupolar splitting are observed with the valine‐derived gels (**p1**), which show similar curve shapes, albeit with a vertical upshift for the gels with the shorter crosslinker (**CL‐Si65**). These gels have proven to be the most versatile ones with respect to the constitutional scope of the analytes and the orientational strength. Gels with the longer crosslinker **CL‐Si400** are preferable when compounds with strong interacting functional groups (e.g., perilla acid) or large aspect ratios (e.g., cholesterol) are investigated, as will be discussed later.

**FIGURE 4:**
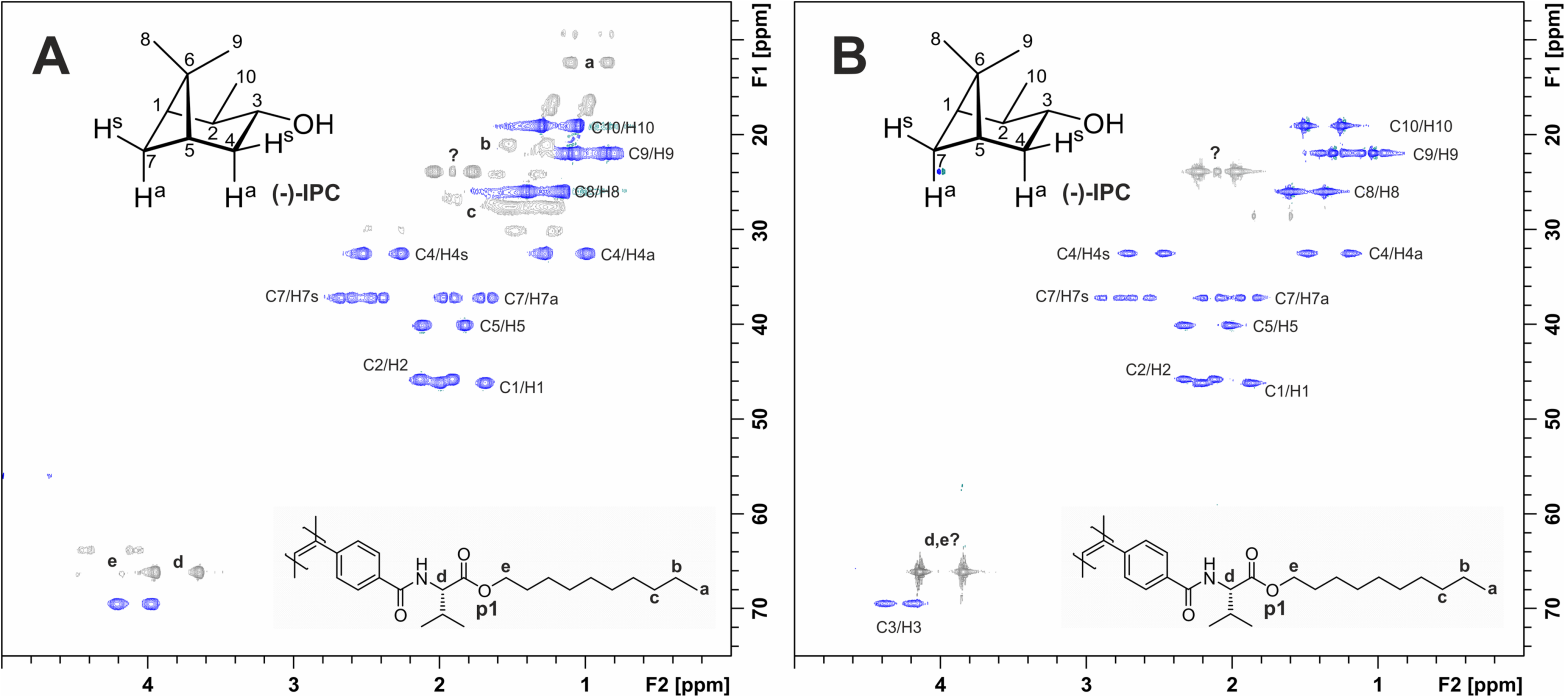
(A) Highfield part of a 500‐MHz CLIP‐HSQC spectrum of 38.8 mg (−)‐IPC in a pristine **p1/CL‐C10** gel (**S16**). Note the rather strong artifacts (gray contours) from trapped monomers and oligomers. (B) Same spectral region after washing the gel by sequential isotropic swelling in dichloromethane and THF (32 mg (−)‐IPC; **S138**). **S#** refers to the gel ID and composition as depicted in Table S2.

In the course of these studies, we observed more or less intense signals from impurities in the pristine gels that turned out to be trapped monomer residuals as well as oligomers. After sequential isotropic swelling in DCM and THF (Supporting information S1 1.3) the situation improved very much, but some impurities seemed to remain stubbornly in the gel, which caused unwanted signals in the CLIP‐HSQC spectra that could interfere with RDC acquisition, especially with small quantities of analyte (Figure 4).

As can be seen in Figure 4A, the residual signals (gray contours) appear partly with a higher intensity than those of the analyte signals, even at a high concentration of **IPC** (38.8 mg/0.7 mL CDCl_3_). After the washing procedure, the intensity of many disturbing signals is reduced considerably, but especially two strong artifacts at around 2 and 4 ppm remain (Figure 4B). Although these results were already very encouraging, there was obviously still room for improvement. In particular, we envisioned a new linker design to meet the following five demands:

1. Easy removal of impurities (residual monomers, oligomers).
2. Soft, elastic polymer sticks which have as little influence as possible on the helical structure of the polyarylacetylenes during both the polymerizing, cross‐linking, and the swelling process.
3. Achiral, readily available starting materials.
4. Decoupling of crosslinker structure and functional monomer—one linker for all.
5. Residual signals from the linker should be as weak as possible and preferentially in a spectral region not interfering with analyte signals.

Facing this list of requirements, the choice fell on bisaminopropyl‐terminated polydimethylsiloxanes (PDMS). These polymers are commercially available with different molecular weights at a reasonable price. They are characterized by very low glass transition temperatures (typically below −120°C) [21] and their ^1^H and ^13^C resonance frequencies are typically far off any analyte signals. Moreover, if wanted, off‐resonance decoupling or simply folding in F1 will remove any potential problems with polymer signals. High molecular weight PDMS gels crosslinked by 10‐MeV β‐rays have already been employed as SAG media in various solvents, showing excellent swelling properties [17b]. The crosslinkers **CL‐Si65** and **CL‐Si400** can be used for gel preparations in the same way as **CL‐C10** while acting both as a crosslinker and as a random‐coil matrix for the PPA‐helices (Scheme 1; for details of their preparation, see Supporting information S1 1.2.8).

**FIGURE 5:**
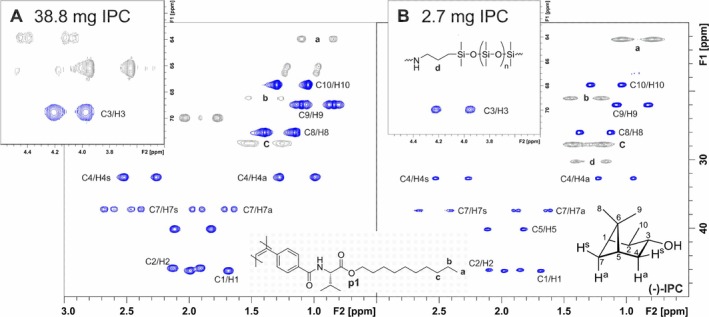
The 500‐MHz CLIP‐HSQC spectra of (−)‐IPC. (A) (**S16**): 38.8‐mg IPC in **p1**/**CL‐C10** gel. (B) (**S133**): 2.7‐mg IPC in **p1**/**CL‐Si400** gel. Note the nearly complete absence of artifact signals in the mid‐field region of (B) (insert) despite a 14.4‐fold lesser concentration of the analyte as compared with (A). The nondisturbing residual signal of the siloxane around 0 ppm is not shown.

When used in combination with **1** or *ent*‐**1**, the optimum amount of crosslinker was found to be 10 mol% for **CL‐Si65** and 5 mol% for **CL‐Si400**, which corresponds to a PDMS/PPA mass ratio of 58/42 for **CL‐Si65** and 80/20 for **CL‐Si400**, respectively. Gels of these compositions, with a monomer concentration of 25 w% in the starting solution and a monomer/initiator ratio of 250:1, were synthesized, washed as previously described, and, after drying, swollen again with solutions of both enantiomers of **IPC** in deuterated chloroform. To our delight, the intensity of the previously observed residual signals has decreased dramatically as compared with the PPA homopolymer gels with the **CL‐C10** linker. Furthermore, equilibrium swelling times could be cut down to 10 days for gels based on **CL‐Si65** and just 7 days for those based on **CL‐Si400**. The line widths of the analyte signals obtained from these gels were substantially lower than those obtained with gels based on **CL‐C10**, indicating the expected enhanced mobility of the analyte in the “soft” polysiloxane network. In the sequel, we attempted to remove the nonpolymeric impurities even further by extracting the pristine polymer sticks based on **CL‐Si65** and **CL‐Si400** (**S133**) in a Soxhlet extractor with dichloromethane for 72 h. After the extraction, the sticks were dried, which proceeded without any deformation or cracking. As an example, **S133** was swollen in 0.7 mL of a solution containing only 2.7 mg of (−)‐**IPC** in CDCl_3_. The CLIP‐HSQC spectrum obtained from this stick was compared with a 38.8‐mg (−)‐**IPC**‐containing **S16/CL‐C10** stick (Figure 5). To our delight, the intensity of the previously observed residual signals was reduced to such an extent that the precise extraction of RDCs was possible even at such a low analyte concentration. Of course, at such low concentrations of the analyte, some unwanted signals still show up, but this time, these broad signals do not belong to the residual oligomers, but to the PPA backbone itself. Fortunately, these peaks are relaxation broadened to such an extent that they can be easily identified as artifacts. Moreover, the application of a T_2_‐filter remains an option to reduce these signals further.

**FIGURE 6:**
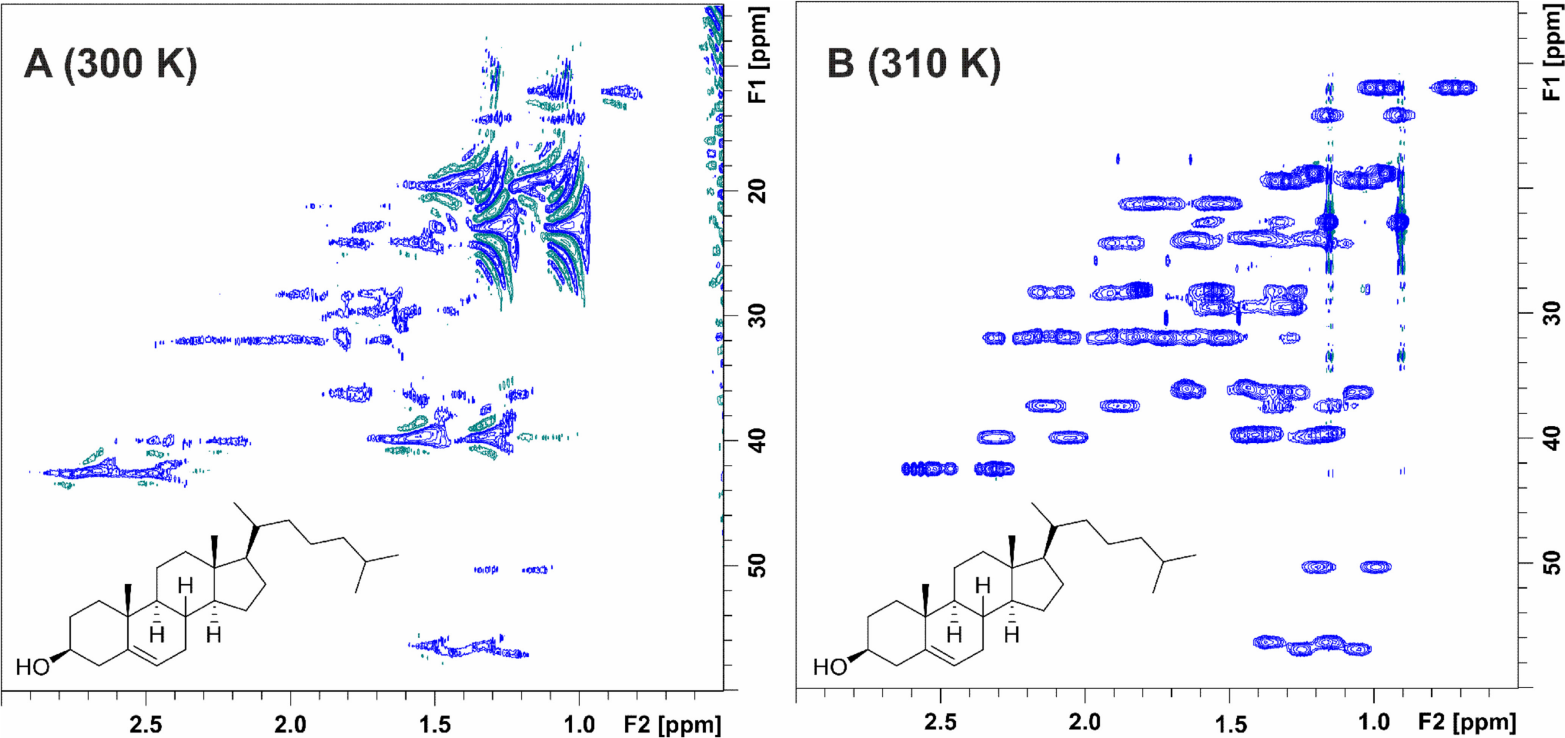
Comparison of 500‐MHz CLIP‐HSQC spectra of cholesterol acquired at (A) 300 K and (B) at 310 K (**S187**).

Finally, the pronounced response of the polysiloxane crosslinked gels to temperature changes is very useful to manipulate the degree of the analyte orientation if needed by increasing the temperature to such an extent that it becomes possible to analyze their NMR spectra. A 500‐MHz CLIP‐HSQC spectrum of cholesterol in a **p1/CL‐Si400** gel (**S187**, Table S2) measured at 300 K showed heavily distorted line shapes, which make the extraction of ^1^ *T*_CH_‐couplings impossible (Figure 6A). This is a typical observation with analytes that either interact strongly with the medium or have a high aspect ratio like perilla acid or cholesterol, respectively. A simple increase of the temperature by 10 K improved the spectral quality to such an extent that the couplings can be measured with great accuracy (Figure 6B).

### 2.2 Enantiodifferentiation

The polymer gels prepared are chiral and of uniform absolute configuration. The centrochirality of the amino acids in the sidechains determines the helical sense of the polymer backbone, entailing a mutual dependence of these two elements of chirality.

**FIGURE 7:**
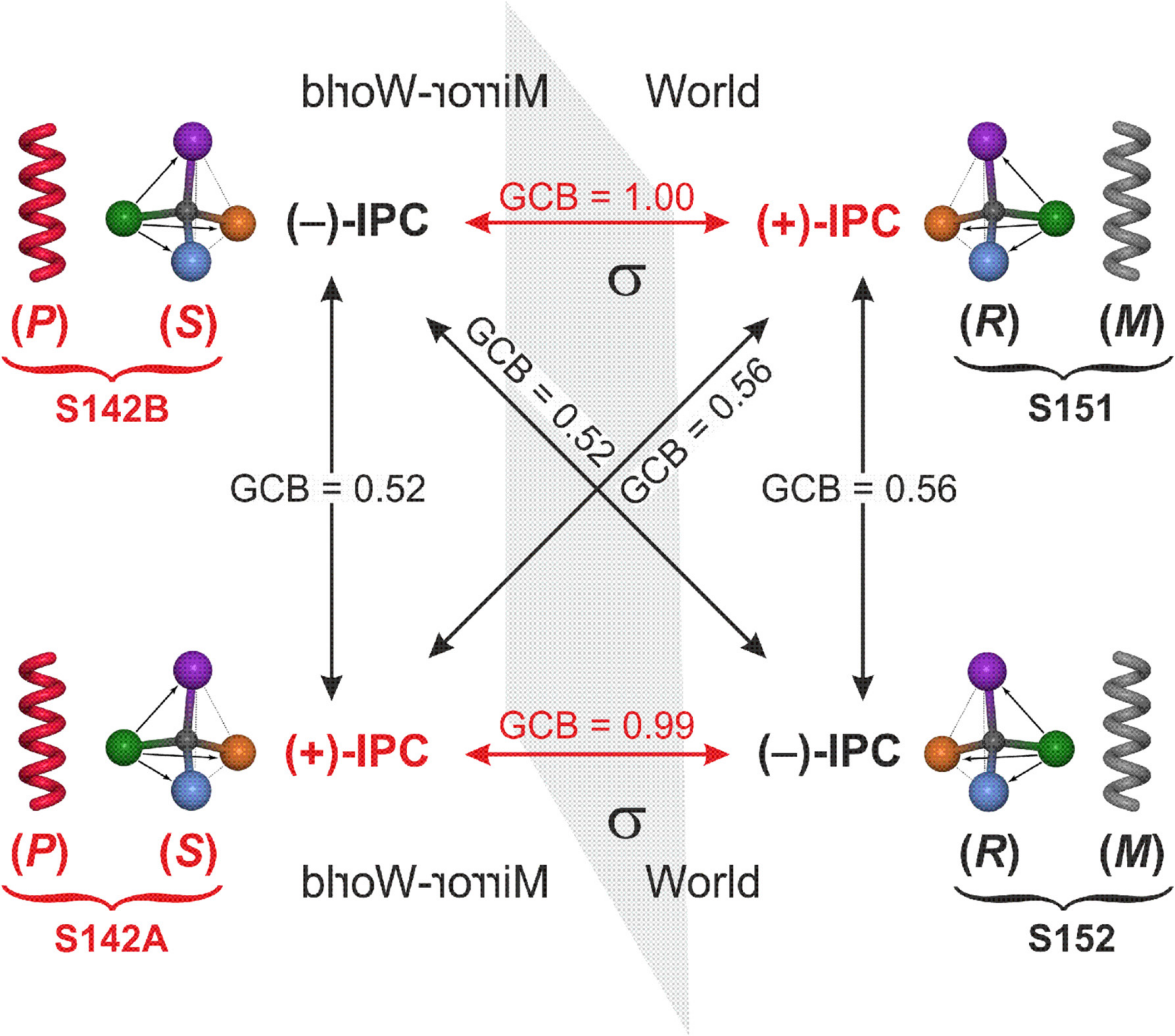
Tensor comparisons calculated for all possible combinations of stereogenic elements in the gels as well as in the analytes. The different designations for polymers of identical composition are due to the fact that the measurements were carried out in different gel preparations.

In case of the valine derived **p1** the (natural) configuration (*S* or *L*, respectively) induces a P‐helix [22] and vice versa. Given the advantageous properties of the PDMS crosslinked gels, we decided to focus our attention on these systems. In the context of enantiodifferentiation, a natural goal is to maximize the difference of the alignment tensor orientations for the enantiomers in the chiral gels (heterochiral comparison). Moreover, we intended to check the reproducibility of the stick preparations by comparing the tensors calculated for the two possible mirror‐image situations (upper and lower rows in Figure 7). This comparison should yield an intertensor angle (ITA) as close as possible to 0 (or *the generalized cosine β* [GCB] [23] as close as possible to 1). This is important because the enantiomers are measured in different gels. Finally, the gels produced with an inverted absolute configuration of the polyacetylene are important to be able to analyze analytes that are available in only one absolute configuration (typical for natural products, for example). These experiments were done using four different gels loaded with both **IPC**‐enantiomers (Table 1 and Figure 7).

**TABLE 1:** Gel parameters and analytes used for the tensor comparisons.

Stick (**S#**)	x (**CL**)	pn	w [%]^a^	M:I^b^	Δv_Q_ [Hz]^c^	Analyte/solvent
**142A**	0.10 **CL‐Si65**	p1	25	250	20	(+)‐IPC (31.5 mg) CDCl_3_
**142B**	0.10 **CL‐Si65**	p1	25	250	19	(−)‐IPC (31.8 mg) CDCl_3_
**151**	0.10 **CL‐Si65**	*ent‐*p1	27.5	500	37.5	(+)‐IPC (32.0 mg) CDCl_3_
**152**	0.10 **CL‐Si65**	*ent‐*p1	27.5	500	37.2	(−)‐IPC (29.0 mg) CDCl_3_

^a^ Combined w/w percentage of crosslinker and polymer.

^b^ Monomer to initiator ratio.

^c^ Absolute value of quadrupolar splitting.

As chiral analyte, we decided to use isopinocampheol (**IPC**) for a number of reasons: The compound displays a rigid molecular framework, thus avoiding problems from conformational averaging. It is available in both enantiomeric forms, and it has been used many times before by us [3, 4, 5, 6, 14, 24] and others [25] to judge the enantiodifferentiating capability of chiral media in the context of anisotropic NMR‐spectroscopy.

The tensor calculations were based on one‐bond ^1^H‐^13^C‐RDCs (^1^ *D*_CH_) obtained by extracting total coupling constants ^1^ *T*_CH_ from CLIP‐HSQC spectra [26] and calculating the RDCs using the relation: ^1^ *T*_CH_ = ^1^ *J*_CH_ + 2 ^1^ *D*_CH_. The datasets, together with a structure model, were used to calculate the alignment tensors by singular value decomposition [27] using the recently introduced software *ConArch+* (see Supporting information S1, sect. 3) [28]. The tensors were compared by calculating the normalized 9D scalar product, the GCB‐value (Figure 7) [23].

**FIGURE 8:**
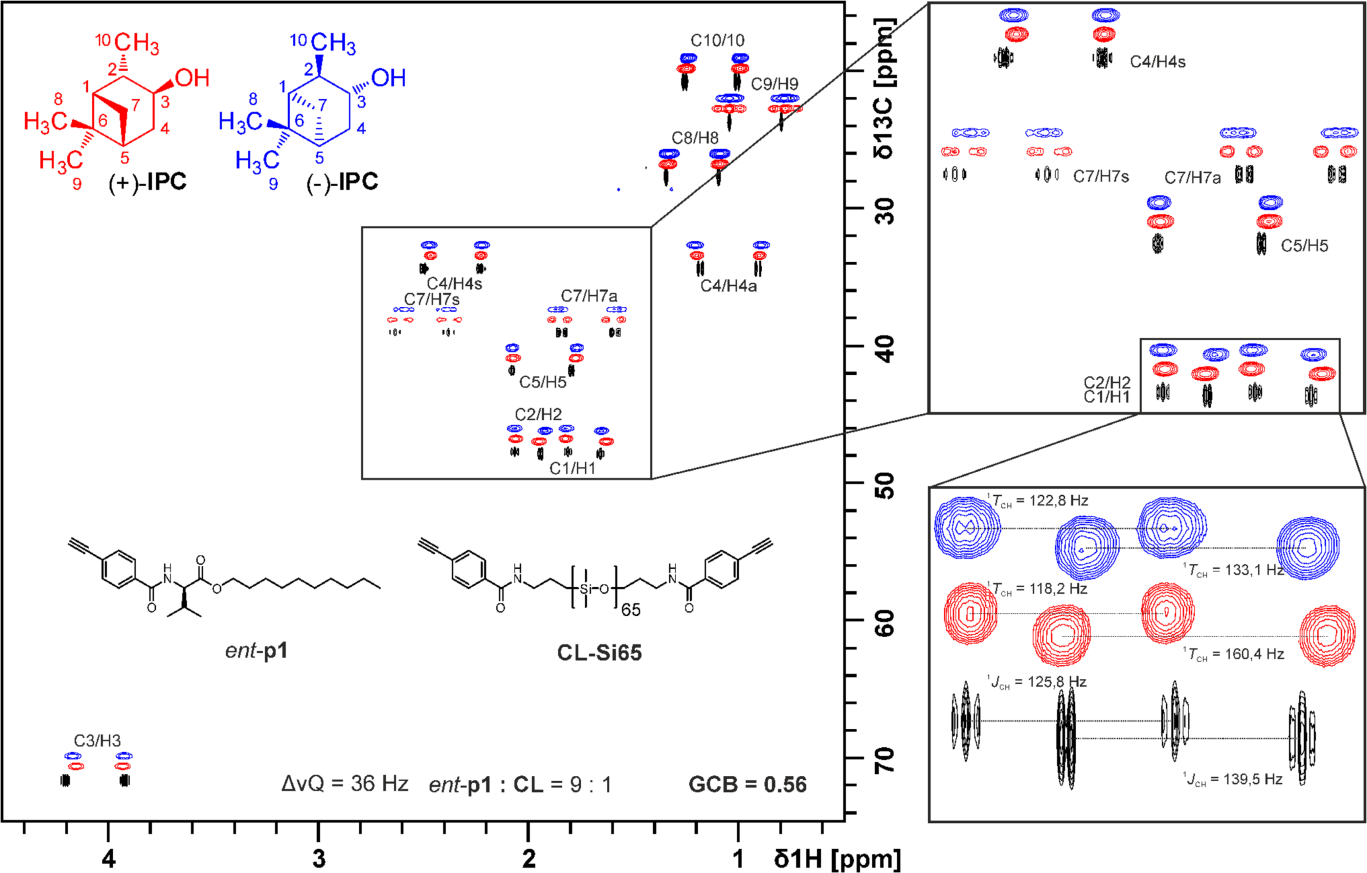
Superposition of 500‐MHz CLIP‐HSQC spectra (CDCl_3_; 300 K). Black contours: isotropic; red contours: (+)‐**IPC (S151)**; blue contours: (−)‐**IPC (S152)**. For stick compositions, see Table S2.

**FIGURE 9:**
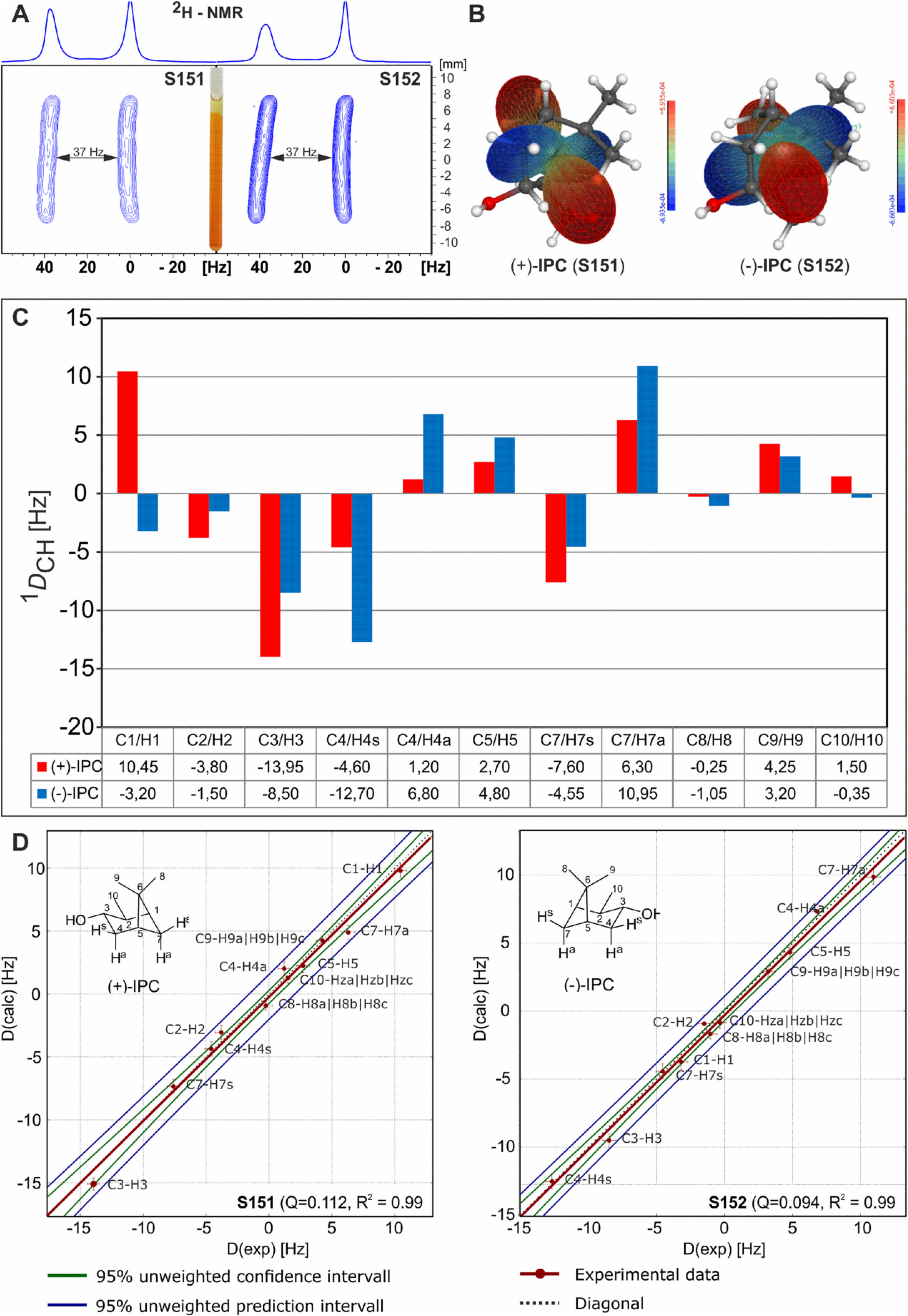
RDC analysis of the enantiomers of **IPC** in *ent*‐**p1**/**CL‐Si65** gels. (A) ^2^H‐NMR and ^2^H‐Image of CDCl_3_ in the gels **S151** and **S152**. (B) Graphical representations of the alignment tensors for (+)‐ and (−)‐IPC, respectively. (C) Box plot of RDCs (^1^ *D*_CH_) for the two enantiomers (red: (+)‐IPC; blue: (−)‐IPC). (D) Correlation of calculated versus experimental RDCs with Cornilescu Q‐factor (Q) [29] [30] [6d] and the coefficient of determination (*R* ^2^). Data were collected in CDCl_3_ at 300 K.

To determine the enantiodifferentiating capability of these valine‐derived gels, we first measured the enantiomers of **IPC** in *ent*‐**p1**‐containing gels (**S151** and **S152**; Figures 8 and 9). Although the ^2^H images of the gels show minor spatial inhomogeneities of the quadrupolar splitting (Figure 9A), the CLIP‐HSQC spectra are of very good quality and nearly completely devoid of any background signals (the siloxane resonances—not shown—appear around 0 ppm). The only slightly broadened lines compared with the isotropic spectrum allow for a precise extraction of the couplings being in a favorable range (−14 Hz ≤ ^1^ *D*_CH_ ≤ + 11 Hz. (see Supporting information S1 sects. 3.1.10 and 3.1.11 and Figure 9C). From the data, it was possible to calculate the alignment tensors for both **IPC**‐enantiomers (for a graphical representation, see Figure 9B), and together with the structure model, theoretical RDCs could be calculated. These latter values show very good correlations with the experimental ones (Figure 9D).

To our delight, we found a pronounced enantiodifferentiation as judged from the rather low GCB‐value of 0.56 corresponding to an ITA of 56°, which is, to the best of our knowledge, the lowest (highest, respectively) value ever measured for a chiral compound in a chiral gel (GCB [**IPC**] in cl‐PBLG: 0.89 [9]; GCB [different amine hydrochlorides] > 0.95) [8]. However, it should be noted here that in the *LLC‐phase* of **p1** (solvent CDCl_3_), this GCB‐value is even close to zero, which means that in this latter medium, the enantiodifferentiation is close to the theoretical maximum (orthogonal alignment tensors) [6d].

As depicted in Figure 7, there are six possible stereoheterotopic combinations resulting from the fact that *both* the polymer *and* the analyte can adopt two different absolute configurations. This entails the expectation that the two “horizontal” mirror‐image relations (red arrows) should yield GCB values close to 1 and that we have two alternatives to generate diastereomorphic relations: (a) keep the polymer constant and invert the analyte's absolute configuration (vertical relations) or (b) keep the analyte's absolute configuration and invert the polymer (diagonal relations). In the latter cases, we aim at small GCBs (high enantiodifferentiation) and expect them to be mutually equal. To our delight, although four different gels were used, all expectations are nearly perfectly fulfilled. This demonstrates the reproducibility of the gel preparations and the robustness of the preparations against experimental errors. The diagonal relations are the ones of importance in the structure elucidations of natural products (only one absolute configuration is available) as mentioned above.

### 2.3 Constitutional Scope

**FIGURE 10:**
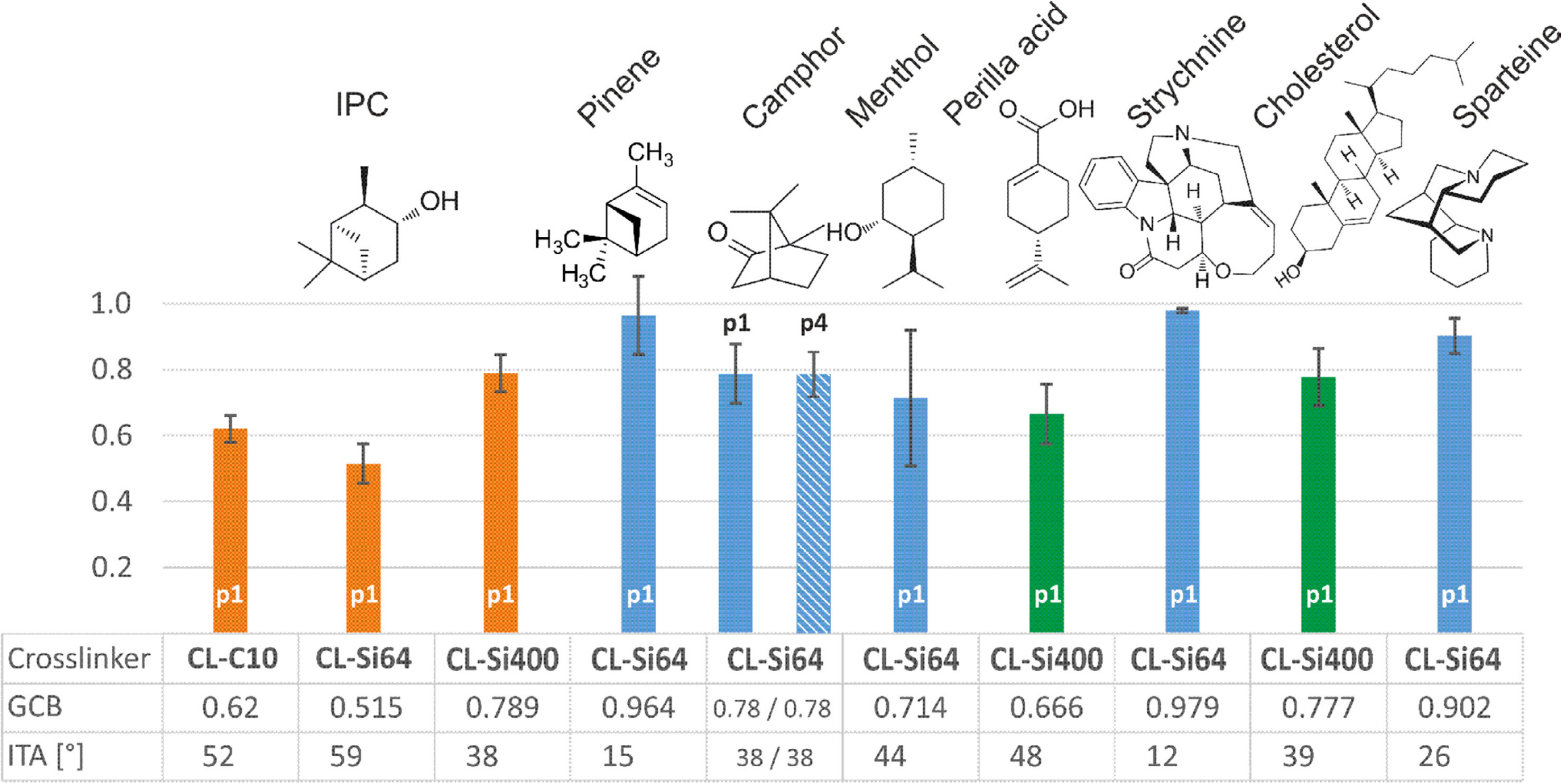
GCB values and intertensor angles (ITA) for 11 analyte/crosslinker combinations with **p1** and **p4** as chirality source. All measurements at 300 K except cholesterol and perilla acid (310 K).

To learn more about the compatibility of the PDMS‐crosslinked polymer **p1** gels with different analytes and the corresponding enantiodifferentiation, we investigated seven other compounds with different functional groups and molecular shapes (Figure 10). The first interesting observation is that for **IPC**, the ITA calculated for the gel with the **CL‐C10** linker is (a little) lower than the one for the **CL‐Si65** system. This is quite unexpected because the fraction of the helically chiral polymer chains is much higher in the former system than in the latter. Therefore, one could expect a higher enantiodifferentiation with the **CL‐C10** systems, which was not observed. A possible explanation may be the fact that in the **CL‐Si65** crosslinked gels, the structural integrity of the **p1** helices is higher than in the close‐meshed, rigid system found with the short **C10** crosslinker. This interpretation is in accordance with our earlier observation that the uniform helicity of the polymer chains is the most important factor governing the enantiodifferentiation [14c]. Moreover, the color of the PDMS‐crosslinked gels is much lighter (yellow, comparable with the noncrosslinked **p1**) than the **C10**‐crosslinked gels (dark red). Given the fact that polyarylacetylenes change color under pressure (from the yellow *cis‐transoid* to the red *trans‐transoid* form) [31], it is reasonable to compare the stress on the polymer chains exerted by pressure with the one generated by forcing the chains into a stiff, tight polymer network. Of course, when the chiral information is diluted further, there must be a point where the capability to differentiate the enantiomers decreases. This, indeed, can be observed with the **p1**/**CL‐Si400** system, as expected. Therefore, the lower crosslink density or the softer network structure of the **CL‐Si400**‐based systems may be detrimental in terms of enantioselectivity but is useful when it comes to analytes with larger aspect ratios and/or strong interactions with the matrix, as is the case for cholesterol and perilla acid, respectively (Figure 10, green bars, and Figure 6). For these two compounds, it was impossible to extract RDCs from CLIP spectra using the shorter **CL‐Si65** crosslinker, even at elevated temperatures. Only the use of the **CL‐Si400** linker brought the desired success with measurements at 310 K.

As far as enantioselectivity is concerned, we found substantial differentiation for menthol (GCB = 0.71; ITA = 44°), camphor (GCB = 0.79; ITA = 38°), and sparteine (GCB = 0.90; ITA = 26°) with **CL‐Si65** as crosslinker and **p1** as chirality source. The camphor results merit a comment. With the *LLC‐phase* of **p1** in CDCl_3_, the camphor enantiomers are hardly differentiated at all (GCB = 0.99) [6d]. Therefore, with the **p1**‐containing *gels*, a substantial improvement can be observed. The opposite is true for the **p4**‐containing systems. Here, the corresponding LLC‐phase performs much better than the gel (GCB = 0.21 [6c] vs. 0.78, respectively). With the **p1**/**CL‐Si400** system, we also noted good enantioselectivities for cholesterol (GCB = 0.77; ITA = 39°) and perilla acid (GCB = 0.66; ITA = 48°). As observed with the *LLC‐phases* of **p1**, strychnine remains a problem and pinene is difficult too. We think that the problem with pinene lies more in its spherical shape than in the lack of functional groups. In an LLC‐phase of **p1**, the enantiomers of the unfunctionalized, but nonspheric, hydrocarbon 3‐methyl hexane are strongly differentiated! [3d].

### 2.4 Temperature Dependence of the Analyte Orientation

Up to this point, we discussed the temperature dependence of the orientational *strength* of the *solvent* on the polymer structure and the linker (Figure 2). Next, we were interested in the question of what influence the temperature has not only on the *strength* of the orientation (size of the alignment tensor), but in particular on *the orientation itself* (shape of the tensor). This is an important question for two reasons:

If only the *size* of the tensor changes after a temperature change, but not its orientation, then the ITA does not change either. This behavior is usually observed with the LLC phases and is advantageous if two enantiomers can only be measured at two different temperatures. This ensures that the ITA only depends on the extent of enantiodifferentiation and is not super positioned by temperature effects. On the other hand, *a temperature‐dependent change in tensor orientation* would be desirable when it comes to determining *relative* configurations of chiral compounds. In this context, it turned out that the determination of anisotropic parameters from only one alignment medium may not be sufficient to fix the relative configuration with the necessary confidence [28, 32]. In such a case, it would be desirable to measure several linearly independent tensors from the same sample by simply changing the temperature without having to change the alignment medium. This would be very interesting, especially in connection with structure determinations of natural substances, which are typically only available in very small quantities and where a loss of material caused by changing the alignment medium is not tolerable.

Choosing the enantiomers of **IPC** as analytes, we measured two series of nine CLIP‐HSQCs at nine different temperatures from 280 to 320 K (step size 5 K) using the standard **p1**/**CL‐Si65** (Table 2; column pair A) and **p4**/**CL‐Si65** (column pair B) gels. Every spectrum allowed for the extraction of the 11 possible ^1^ *D*_CH_‐couplings (SI, Table S5) which were used as usual to calculate the corresponding alignment tensors. Next, we compared these tensors in two ways: First, the tensors of the same **IPC** enantiomer at different temperatures were compared (homochiral comparison), and second, those of the enantiomers at different temperatures (heterochiral comparison; Table 2).

**TABLE 2:** Temperature dependent homo‐ and heterochiral tensor comparisons (GCB‐values) for (+)‐ and (−)‐**IPC** in two different gel compositions.^a^

		**A p1**/**CL‐Si65; S179/181**		**B p4**/**CL‐Si65; S245/246**	
T [K]		280	280	280	280
	**IPC**	(+)	(−)	(+)	(−)
280	(+)	0.992	0.293	0.992	0.930
	(−)	0.293	0.986	0.930	0.986
285	(+)	0.900	0.220	0.990	0.944
	(−)	0.370	0.894	0.894	0.978
290	(+)	0.668	0.323	0.990	0.941
	(−)	0.258	0.722	0.854	0.966
295	(+)	0.357	0.393	0.988	0.949
	(−)	0.207	0.602	0.850	0.963
300	(+)	0.178	0.433	0.987	0.944
	(−)	0.209	0.540	0.816	0.942
305	(+)	0.118	0.460	0.985	0.946
	(−)	0.237	0.519	0.793	0.930
310	(+)	0.140	0.450	0.983	0.948
	(−)	0.258	0.504	0.760	0.912
315	(+)	0.180	0.470	0.982	0.945
	(−)	0.280	0.497	0.758	0.908
320	(+)	0.212	0.481	0.974	0.940
	(−)	0.309	0.495	0.707	0.872

^a^ Homochiral comparisons are colorized using a color spectrum from green in case of collinearity to red indicating orthogonality. **S#**: Stick‐ID as defined in Table S2.

**FIGURE 11:**
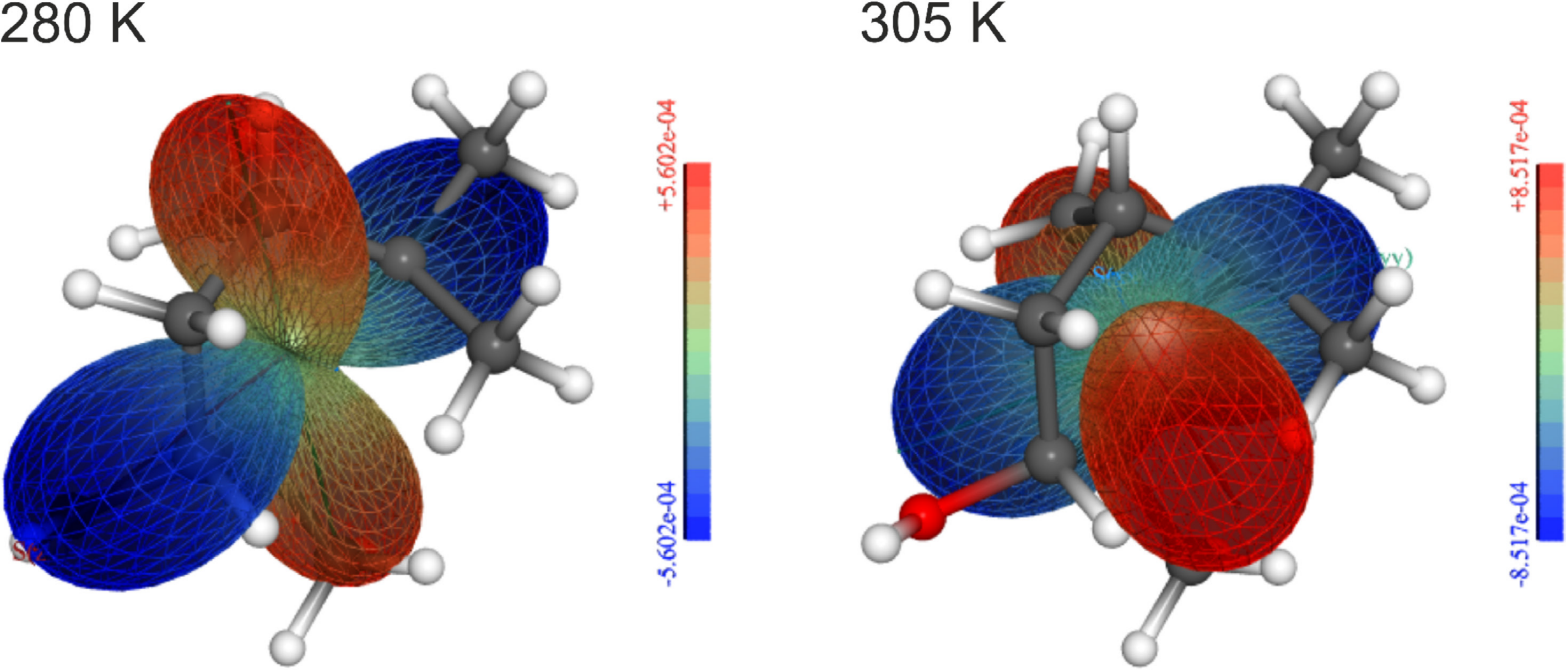
Homochiral tensor comparison for (+)‐**IPC** in **p1**/**CL‐Si65** gels at 280 and 305 K. The intertensor angle (ITA) is 83° (GCB = 0.118).

Especially from the **A**‐series (**p1**/**CL‐Si65**), it is obvious that the temperature‐related changes in the orientation of **IPC** are different for the two enantiomers. This applies in two respects: The change in orientation as a function of temperature occurs within **IPC** of the same absolute configuration (homochiral comparison) and of opposite configuration (heterochiral comparison). This has two important consequences: The homochiral comparison of (+)‐**IPC** at 280 K with (+)‐**IPC** at other temperatures delivers quite different GCB values. This is true especially for the comparison with the tensor at 305 K in this series. Here a GCB value of 0.118 can be calculated, which corresponds to an ITA of approximately 83°! (Figure 11 and Movie S1—animation; .mp4‐file).

In fact, the orientation changes so much even with small temperature changes that this behavior could be used to generate multi‐alignment data sets. *Under small temperature changes, the system behaves like one that could have been achieved by using several independent media*.

**FIGURE 12:**
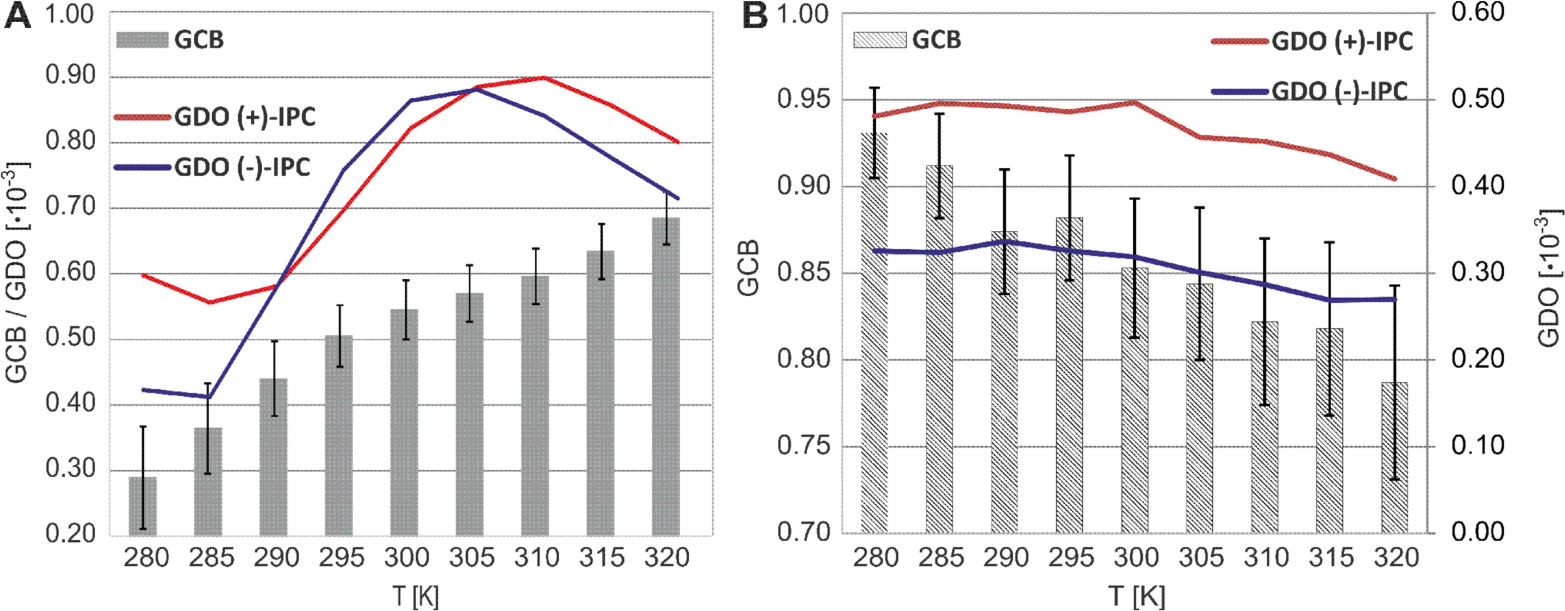
Temperature dependence of GDO and GCB values for the **IPC** enantiomers in **p1**/**CL‐Si65** (A; **S179/S181**) and **p4**/**CL‐Si65** (B; **S245**/**S246**) gels.

As a second consequence, the enantiodifferentiating capability of the system is strongly dependent on the measuring temperature (Figure 12A). As already mentioned earlier, the GCB for **IPC** in the **p1**‐containing gel is 0.56 (ITA = 56°) at 300 K (Figure 8). Not unexpectedly, this value falls as the temperature drops, reaching 0.29 at 280 K (ITA = 73°), indicating an exceptional enantiodifferentiation at this temperature. Interestingly, there is no correlation between the general degree of order (GDO) for the individual enantiomers and the GCB. At 305 K, both enantiomers have the same GDO but are differentiated considerably based on their different tensor orientations. Moreover, in the temperature range between 285 and 305 K, the ordering of **IPC** increases, whereas the alignment of the solvent CDCl_3_ decreases (Figure 2; **p1**/**CL‐Si65**). Obviously, there are two mutual superimposing modes of interaction between the alignment medium and **IPC**: one stereounselective mode related to interactions caused by properties like shape, electrostatics, or H‐bonding, and another mode related to the spatial orientation of the interacting moieties.

Given this interesting finding, we next investigated whether the **p4**‐based gels (Table 2, Series B) also exhibit a comparable behavior. As with the **p1**‐containing gels, we again measured CLIP‐HSQC spectra of the **IPC** enantiomers in the temperature range between 280 and 320 K, incrementing the temperature in 5 K steps. Again, we determined the GDO for the individual enantiomers and the GCB values for both the homochiral and heterochiral comparisons. This time, the homochiral comparisons (colorized cells) show only weak dependencies from the temperature, reaching a maximum difference in orientation of only ITA = 29° (GCB = 0.872) for the 280/320 K pair. This behavior resembles the one usually observed with the chiral LLC phases. A surprising fact, on the other hand, is the *increase* in enantiodifferentiation with increasing temperature (Figure 12B)!

An (admittedly rather speculative) explanation for this surprising behavior may be found in the different flexibility of the different polymer chains (**p1** vs. **p4**) embedded in the crosslinked gels. It is known that polyacetylenes are highly dynamic polymers with low energy barriers for helix reversals [13, 33]. These barriers are expected to be higher in the crosslinked network of a gel as compared with the LLC state or even in isotropic solution. If one now assumes that conformational changes in the sense of an “induced fit” are necessary to maximize the enantiomer‐differentiating capability of a given helical polymer; then at the same temperature, the more flexible one will show a better enantiodifferentiation than the less flexible polymer. If we now make the (reasonable) assumption that **p4** with the more sterically demanding side chains (as compared with **p1**) reaches this flexibility only at higher temperatures, then this may be the reason for the experimental outcome described above.

## 3 Conclusions

Helically chiral polyacetylenes with amino acid sidechains form crosslinked gels in a crosslinking copolymerization reaction using different types of diynes as crosslinking agents. Difunctionalized polydimethylsiloxanes (PDMS) of different molecular weights with 4‐ethinylbenzoic acid amides at their respective chain ends turned out to be the superior choice. The resulting gels swell in different solvents (CDCl_3_, toluene, dichloromethane, and THF) generating an anisotropic environment $0.5\cdot 10^{‐3}\leq GDO\leq 1.0\cdot 10^{‐3}$ suited to measure one‐bond carbon‐proton RDCs (^1^ *D*_CH_) precisely from CLIP‐HSQC spectra of different analytes. An additional advantageous property of these gels is the favorable spectral position of the residual polymer signals close to zero ppm related to the siloxane. The alignment strength is adjustable by varying the monomer concentration (macro crosslinker and functional polymer) or, more convenient, just by temperature variation. The capability of the gels to differentiate the enantiomers of chiral analytes is unreached by any other chiral gel described so far. This is especially true for the valine derived system **p1**/**CL‐Si65**, which generates an ITA of 56° (GCB = 0.56) at 300 K and even 73° (GCB = 0.29) at 280 K for the **IPC**‐enantiomers (heterochiral tensor comparison). In addition, this system has the remarkable property of orienting both (+)‐ and (−)‐**IPC** individually very differently depending on the temperature (homochiral tensor comparison). This, principally, offers the opportunity to measure “multi‐alignment data sets” without changing the sample. Work along these lines is currently under way.

## 4 Experimental Section

Experimental details for the monomer, polymer, and phase preparations as well as sample compositions can be found in Supporting information S1. Moreover, characterization data (ORD‐ and NMR data) and tensor calculations can be found there.

## Conflicts of Interest

The authors declare no conflicts of interest.

## Supporting information


**Table S1:** Reagents and solvents used for sticks **S** **142A** and **S** **142B**.
**Table S2:** Synthesis parameters of all sticks used in NMR alignment experiments.
**Table S3:** Results of the NMR alignment experiments by stick.
**Table S4:** Numbering of the nuclei, assignment of the chemical shifts δ and scalar 1JCH couplings for the enantiomers of IPC. *s* = syn to the methylene bridge; *a* = anti.
**Table S5:** RDCs of IPC in the indicated sticks. For stick pairs in which both enantiomers were measured, the (+) enantiomer was measured in the first‐mentioned stick and the (−) enantiomer in the second‐mentioned stick.
**Table S6:** Numbering of the nuclei, assignment of the chemical shifts δ and scalar *1JCH* couplings for the enantiomers of camphor. *s = syn* to the methylene bridge; *a = anti*.
**Table S7:** RDCs of Camphor in the indicated sticks.
**Table S8:** Numbering of the nuclei, assignment of the chemical shifts δ and scalar *1JCH* couplings for the enantiomers of α‐pinene. *s = syn* to the methylene bridge; *a = anti*.
**Table S9:** RDCs of Pinene in the indicated sticks.
**Table S10:** Numbering of the nuclei, assignment of the chemical shifts δ and scalar *1JCH* couplings for the enantiomers of menthol. *a = axial; e = equatorial*.
**Table S11:** RDCs of menthol in the indicated sticks.
**Table S12:** Numbering of the nuclei, assignment of the chemical shifts δ and scalar *1JCH* couplings for perilla acid.
**Table S13:** RDCs of perilla acid in the indicated sticks.
**Table S14:** Numbering of the nuclei, assignment of the chemical shifts δ and scalar *1JCH* couplings for strychnine.
**Table S15:** RDCs of strychnine in the indicated sticks.
**Table S16:** Numbering of the nuclei, assignment of the chemical shifts δ and scalar *1JCH* couplings for sparteine. *a = axial; e = equatorial*.
**Table S17:** RDCs of sparteine in the indicated sticks.
**Table S18:** Numbering of the nuclei, assignment of the chemical shifts δ and scalar *1JCH* couplings for cholesterol. *a = alpha‐, b = beta‐*side of the steroid structure.
**Figure S1:** 1H‐NMR spectra of DMS‐A21 (**SI‐11**; above) and **SI‐13** (**CL‐SI65**) (below).
**Figure S2: Left:** Schematic representation of different stages of the polymerization procedure. **Right:**The real apparatus showing different stages of the gelation process.
**Figure S3:**. Quadrupolar splitting of **p1‐CL‐C10** sticks at different concentrations (Data for Figure 1A in the manuscript).
**Figure S4:**. Traces of CLIP‐HSQCs of (+)‐IPC (C3/H3‐crosspeak). Blue: Anisotropic in **S151**; Red: Isotropic. Both traces were extracted from the HSQCs (same resolution in F2—8 k); inverse Fourier transformed (ift) – pseudo raw data generated (genfid) then zero‐filled to 32 k and transformed again without apodisation.
**Figure S5:** RDCs of Cholesterol in Stick‐**S187** (*ent*‐**1**) und Stick‐**S** **190** (**1**).

**Movie S1:** Supporting Information.

## Data Availability

The data that support the findings of this study are available from the corresponding author upon reasonable request.
