# Supplementary material for: Chiral Polyacetylene‐PDMS‐Copolymer‐Gels as Enantiodifferentiating Alignment Media
Source: Magn Reson Chem. 2025 Aug 18;63(11):912–26. doi: 10.1002/mrc.70025 (PMC12500359; doi:10.1002/mrc.70025)
Supplement: Supplementary file 1 — Table S1: Reagents and solvents used for sticks S 142A and S 142B. Table S2: Synthesis parameters of all sticks used in NMR alignment experiments. Table S3: Results of the NMR alignment experiments by stick. Table S4: Numbering of the nuclei, assignment of the chemical shifts δ and scalar 1JCH couplings for the enantiomers of IPC. s = syn to the methylene bridge; a = anti. Table S5: RDCs of IPC in the indicated sticks. For stick pairs in which both enantiomers were measured, the (+) enantiomer was measured in the first‐mentioned stick and the (−) enantiomer in the second‐mentioned stick. Table S6: Numbering of the nuclei, assignment of the chemical shifts δ and scalar 1JCH couplings for the enantiomers of camphor. s = syn to the methylene bridge; a = anti. Table S7: RDCs of Camphor in the indicated sticks. Table S8: Numbering of the nuclei, assignment of the chemical shifts δ and scalar 1JCH couplings for the enantiomers of α‐pinene. s = syn to the methylene bridge; a = anti. Table S9: RDCs of Pinene in the indicated sticks. Table S10: Numbering of the nuclei, assignment of the chemical shifts δ and scalar 1JCH couplings for the enantiomers of menthol. a = axial; e = equatorial. Table S11: RDCs of menthol in the indicated sticks. Table S12: Numbering of the nuclei, assignment of the chemical shifts δ and scalar 1JCH couplings for perilla acid. Table S13: RDCs of perilla acid in the indicated sticks. Table S14: Numbering of the nuclei, assignment of the chemical shifts δ and scalar 1JCH couplings for strychnine. Table S15: RDCs of strychnine in the indicated sticks. Table S16: Numbering of the nuclei, assignment of the chemical shifts δ and scalar 1JCH couplings for sparteine. a = axial; e = equatorial. Table S17: RDCs of sparteine in the indicated sticks. Table S18: Numbering of the nuclei, assignment of the chemical shifts δ and scalar 1JCH couplings for cholesterol. a = alpha‐, b = beta‐side of the steroid structure. Figure S1: 1H‐NMR spectra of DMS‐A21 (SI‐11; ab [file MRC-63-912-s001.pdf]

Supporting Information
for

Polyacetylene-PDMS-Copolymer-Gels as Enantiodifferentiating Alignment Media

*Jochen Kornett and Michael Reggelin**

Clemens-Schöpf-Institut für Organische Chemie und Biochemie, Technische Universität Darmstadt, Peter-Grünberg-Straße 4, D-64287 Darmstadt, Germany

Corresponding author: Michael Reggelin (re@chemie.tu-darmstadt.de)

Table of contents

1 Experimental procedures and characterization data 6

1.1 Materials and Instrumentation 6

1.2 Monomer synthesis 7

1.2.1 4-Ethinylbenzoic acid SI-2 7

1.2.2 *p*-Ethinylbenzoyl chloride SI-3 8

1.2.3 GP 1: Synthesis of amino acid alkyl ester ammonium tosylates 8

1.2.4 GP 2: Coupling of 4-ethinylbenzoic acid with amino acid ester ammonium tosylates 9

1.2.5 L-Alanine decylester ammoniumtosylat SI-4 9

1.2.6 *N*-(4-Ethynylbenzoyl)-L-Alanine decylester SI-5 (2) 10

1.2.7 Decanediol bi-*L*-valinate SI-9 10

1.2.8 Bi-acetylene crosslinker SI-10 (CL-C10) 12

1.2.9 Ethinyl-terminated PDMS-crosslinkers Si-13 (CL-Si65) and SI-14 (CL-Si400) 14

1.3 Polymerstick synthesis procedure 17

1.4 Stick composition and synthesis parameters 19

2 NMR studies 21

2.1 Sample preparation 21

2.2 Measurement conditions 21

2.3 ^{2}H-Spectra of Sticks S42-S48 (Data for Figure 1 in Manuscript): 22

2.4 Orientation and differentiation results 23

2.5 RDCs of measured samples by analyte 24

2.5.1 IPC 24

2.5.2 Camphor 28

2.5.3 Pinene 29

2.5.4 Menthol 30

2.5.5 Perilla acid (310 K) 31

2.5.6 Strychnine 32

2.5.7 Sparteine 34

2.5.8 Cholesterol (310 K) 36

3 Calculation of orientational properties 39

3.1 Experimental and calculated RDCs –Tensor properties 40

3.1.1 (-)-IPC in Stick S16 40

3.1.2 (+)-IPC in Stick S137 42

3.1.3 (-)-IPC in Stick S138 44

3.1.4 (+)-IPC in Stick S142A 46

3.1.5 (-)-IPC in Stick S142B 48

3.1.6 (+)-IPC in Stick S143 50
3.1.7 (-)-IPC in Stick S144 52
3.1.8 (+)-Camphor in Stick S149 54
3.1.9 (-)-Camphor in Stick S150 56
3.1.10 (+)-IPC in Stick S151 58
3.1.11 (-)-IPC in Stick S152 60
3.1.12 (+)-(α)-Pinene in Stick S153 62
3.1.13 (-)-(α)-Pinene in Stick S154 64
3.1.14 (+)-Menthol in Stick S155 66
3.1.15 (-)-Menthol in Stick S156 68
3.1.16 (+)-IPC in Stick S167 70
3.1.17 (-)-IPC in Stick S168 72
3.1.18 (-)-Strychnine in Stick S173 74
3.1.19 (-)-Sparteine in Stick S176 77
3.1.20 (+)-IPC in Stick S179 – 300 K out of T-series 79
3.1.21 (-)-Sparteine in S180 81
3.1.22 (-)-IPC in Stick S181 – 300K out of T-series ´ 83
3.1.23 (-)-Strychnine in Stick S185 85
3.1.24 Cholesterine in Stick S187 88
3.1.25 (-)-Perilla acid in Stick S188 @ 310K 91
3.1.26 Cholesterine in Stick S190 93
3.1.27 (-)-Perilla acid (S191) @ 310K 96
3.1.28 (+)-IPC (S282): in dichloromethane @ 300K 98
3.1.29 (+)-IPC in Stick S284: @ 300K in THF 100
3.1.30 (-)-IPC in Stick S285 @ 300K in THF 102

Tables

Table S1 Reagents and solvents used for sticks **S142A** and **S142B**. 18

Table S2 Synthesis parameters of all sticks used in NMR alignment experiments. 19

Table S3 Results of the NMR alignment experiments by stick. 23

Table S4 Numbering of the nuclei, assignment of the chemical shifts δ and scalar $^1J_{CH}$ couplings for the enantiomers of IPC. *s* = syn to the methylene bridge; *a* = anti.................................... 24

Table S5 RDCs of IPC in the indicated sticks. For stick pairs in which both enantiomers were measured, the (+) enantiomer was measured in the first-mentioned stick and the (-) enantiomer in the second-mentioned stick. 24

Table S6 Numbering of the nuclei, assignment of the chemical shifts δ and scalar $^1J_{CH}$ couplings for the enantiomers of camphor. *s* = *syn* to the methylene bridge; *a* = *anti.* 28

Table S7 RDCs of Camphor in the indicated sticks. 28

Table S8 Numbering of the nuclei, assignment of the chemical shifts δ and scalar $^1J_{CH}$ couplings for the enantiomers of α-pinene. *s* = *syn* to the methylene bridge; *a* = *anti.* 29

Table S9 RDCs of Pinene in the indicated sticks. 29

Table S10 Numbering of the nuclei, assignment of the chemical shifts δ and scalar $^1J_{CH}$ couplings for the enantiomers of menthol. *a* = *axial; e* = *equatorial.* 30

Table S11 RDCs of menthol in the indicated sticks. 30

Table S12 Numbering of the nuclei, assignment of the chemical shifts δ and scalar $^1J_{CH}$ couplings for perilla acid. 31

Table S13 RDCs of perilla acid in the indicated sticks. 31

Table S14 Numbering of the nuclei, assignment of the chemical shifts δ and scalar $^1J_{CH}$ couplings for strychnine. 32

Table S15 RDCs of strychnine in the indicated sticks. 33

Table S16 Numbering of the nuclei, assignment of the chemical shifts δ and scalar $^1J_{CH}$ couplings for sparteine. *a* = *axial; e* = *equatorial.* 34

Table S17 RDCs of sparteine in the indicated sticks. 35

Table S18 Numbering of the nuclei, assignment of the chemical shifts δ and scalar $^1J_{CH}$ couplings for cholesterol. *a* = *alpha-, b* = *beta*-side of the steroid structure.. 36

Figures

Figure S1 ^{1}H-NMR spectra of DMS-A21 (**SI-11**; above) and **SI-13** (**CL-SI65**) (below). 16

Figure S2 **Left:** Schematic representation of different stages of the polymerization procedure. **Right:** The real apparatus showing different stages of the gelation process. 17

Figure S3. Quadrupolar splitting of **p1-CL-C10** sticks at different concentrations (Data for Figure 1A in the manuscript). 22

Figure S4. Traces of CLIP-HSQCs of (+)-IPC (C3/H3-crosspeak). Blue: Anisotropic in **S151**; Red: Isotropic. Both traces were extracted from the HSQCs (same resolution in F2 – 8k); inverse Fourier transformed (ift) – pseudo raw data generated (genfid) then zero-filled to 32k and transformed again without apodisation. 27

Figure S5 RDCs of Cholesterol in Stick-**S187** (*ent*-**1**) und Stick-**S190** (**1**). 38

1 Experimental procedures and characterization data

1.1 Materials and Instrumentation

Solvents and reagents: Dichloromethane (DCM) and chloroform [D_1] ($CDCl_3$) were distilled from CaH_2 under argon atmosphere. Tetrahydrofuran (THF) and diethyl ether (Et_2O) were distilled from sodium-benzophenone under argon. Reagents were obtained from commercial sources and used without further purification unless otherwise specified. Moisture and/or air sensitive experiments were conducted under argon atmosphere using typical Schlenk techniques.

NMR-Spectra: ^{1}H-NMR spectra were recorded on Bruker ARX 300 and DRX 500 spectrometers operating at 300 and 500 MHz, respectively at 300 K unless otherwise specified. ^{13}C-NMR spectra were recorded on the same instruments at 75 and 125 MHz, respectively. Chemical shifts (δ) in ^{1}H-NMR and ^{13}C-NMR spectra are reported in ppm. The spectra were referenced against the residual solvent signal as reported in the literature.[1] The fine structure of proton signals was specified as s (singlet), d (doublet), t (triplet), q (quartet), m (multiplet), dd (doublet of doublet), br (broad).

Thin layer chromatography (TLC): Thin-layer chromatography (TLC) was performed using E. Merck silica gel SilG/UV254 by Macherey Nagel & Co., Düren (thickness of layer 0.2 mm) and visualized by UV fluorescence quenching or oxidizing with $KMnO_4$.

IR-spectra were recorded on a Perkin-Elmer spectrometer Paragon 1000 PC or on a Vector 22 spectrometer from Bruker.

Specific optical rotations were determined on a Perkin Elmer Polarimeter 241 with Haake D8 thermostat or on an Anton Paar MCP 300 polarimeter in 1 dm cuvettes respectively.

CD spectra were recorded on a JASCO J-810 or JASCO J-1500 spectrometer equipped with a PTC-423S/15 peltier element as a temperature device.

1.2 Monomer synthesis

For the preparation of **1**/*ent*-**1** see ref [2] for **3**/*ent*-**3** and **4**/*ent*-**4** see ref[3].

1.2.1 4-Ethinylbenzoic acid SI-2

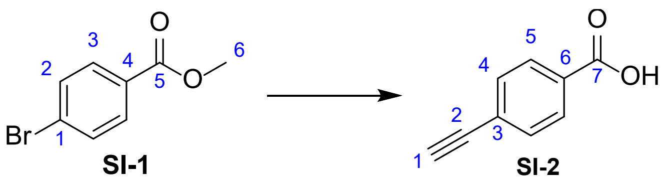

36.280 g (168.7 mmol, 1.00 eq.) of 4-bromo methyl benzoate **SI-1** is placed in a flame-dried SCHLENK flask and dissolved in 700 mL of a solvent mixture of dry THF and triethylamine (v/v = 1:1). Argon is passed through the solution over 15 min with vigorous stirring using a cannula. Then 0.643 g (3.4 mmol, 0.02 eq) copper iodide, 0.885 g (3.4 mmol, 0.02 eq) triphenylphosphane and 1.184 g (1.7 mmol, 0.01 eq) $Pd(PPh_3)_2Cl_2$ are added. After the mixture is cooled to 0°C, 36.0 mL (24.9 g, 253.1 mmol, 1.50 eq.) of trimethylsilyl acetylene were added and stirred at room temperature for 16 h until complete conversion of the bromide.

The crude reaction mixture is filtered through a silica gel frit and rinsed with THF until the filtrate is almost colorless. The filtrate is concentrated to dryness in vacuo, the residue is mixed with 800 mL of a solution of methanol and 1 M sodium hydroxide solution (v/v = 1:1) and stirred overnight at room temperature. The product mixture is then filtered and methanol is removed in vacuo. The remaining aqueous phase is extracted three times with DCM and twice with ether and then acidified with conc. hydrochloric acid to pH<1, forming a precipitate. This is filtered off and the residue is washed with a little water. The collected precipitate is dissolved in ethyl acetate on the glass frit, leaving an insoluble part behind. The two-phase filtrate is separated in a separating funnel and the combined aqueous mother liquor is extracted twice with ethyl acetate. The combined ethyl acetate phases are washed with saturated NaCl solution, dried over $MgSO_4$ and the solvent is completely removed in vacuo leaving 23.238 g (159.0 mmol, 94 %) of 4-ethynylbenzoic acid **SI-2**.

R_F = 0.47 (PE/EE = 1:1)

^{1}H-NMR (DMSO-d$_6$, 300 MHz, 300 K): δ = 4.425 (s, 1-H), 7.591 (d, 4-H$_2$), 7.932 (d, 5-H$_2$), 13.139 (bs, COOH) ppm.

$^3J_{3,4}$ = 8.2 Hz.

1.2.2 *p*-Ethinylbenzoyl chloride SI-3

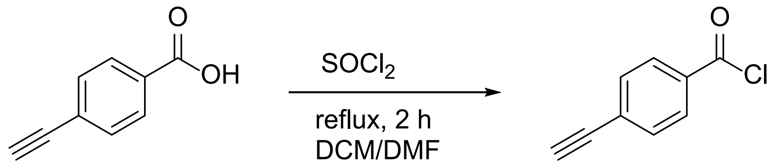

SI-2 **SI-3**

The Synthesis of *p*-ethinylbenzoyl chloride is carried out following a modified procedure from Cheuk et al.[4]. 8.00 g (54.7 mmol; 1 eq) *p*-ethinylbenzoic acid **SI-2** are suspended in 150 ml of dichloromethane in a three-necked flask equipped with a reflux condenser under an argon atmosphere. 0.4 ml of dry dimethylformamide and 11.07 g (93.1 mmol; 1.7 eq) of thionyl chloride are added slowly and the mixture is heated to reflux for 2 hours. During this time, the suspension turns into a clear solution of reddish colour. After cooling to ambient temperature, the mixture is filtered through a celite pad, the solvent and excess thionyl chloride are removed in vacuo. The product is obtained as a brownish, crystalline solid with a yield of 8.90 g (54.1 mmol; 99%). The identity and purity of the product were verified by NMR spectroscopy. The NMR signals are consistent with the literature. The product was stored under argon at -16 °C and was stable for multiple days under these conditions.

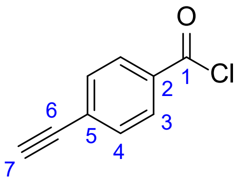

SI-3

^{1}H-NMR (DMSO-d6, 300 MHz, 300 K): δ= 7.93 (d; J=8.5 Hz; 2H; 3-H), 7.58 (d; J=8.5 Hz; 2H; 4-H), 4.41 (s; 1H; 7-H) ppm.

^{13}C-NMR (DMSO-d6, 125 MHz, 300 K): δ= 166.47 (1-C), 132.10 (2-C), 131.86 (4-C), 129.44 (3-C), 126.03 (5-C), 83.50 (6-C), 82.69 (7-C) ppm.

1.2.3 GP 1: Synthesis of amino acid alkyl ester ammonium tosylates

The corresponding amino acid (1.0 eq.), decan-1-ol (0.95 eq.) and *p*-toluene sulfonic acid monohydrate (1.1 eq.) are heated to reflux in toluene (c=0.25 M with respect to amino acid) in a round bottom flask with a DEAN STARK trap attached.

After all water has been separated and the reaction solution is clear, it is cooled to room temperature and the solvent is removed on a rotary evaporator. The residue is taken up in $CHCl_3$ (V (chloroform) = 1/3 V (toluene)). The organic phase is washed with water and dried over $MgSO_4$. The solvent is again removed in vacuo. The crude product is recrystallized from petroleum ether/EtOAc.

1.2.4 GP 2: Coupling of 4-ethinylbenzoic acid with amino acid ester ammonium tosylates

Following the literature[5], 4-ethynylbenzoic acid **SI-2** (1.0 eq.) is dissolved in THF/DCM (exact volumes see the special procedure) in a three-neck flask with a bubble counter attached and cooled to 0°C. Carbonyldiimidazole (1.1 eq.) is added in one portion to the ice-cold solution, stirred for 10 min at 0°C and then at room temperature until gas evolution stops.
The corresponding amino acid ester ammonium tosylate (1.01 eq.) is dissolved in DCM (c = 0.2M) and triturated with saturated Na_2CO_3 solution (V = 1/2 V(DCM)). The organic phase is dried over $MgSO_4$ and the solvent is removed on a rotary evaporator. The colorless oil is taken up in dry DCM (c = 0.45 M).
The reaction solution is cooled again to 0°C and the amine is added over a period of 1 h after which time stirring was continued for 1 h at 0°C and then overnight at room temperature.
The solvent is removed in vacuo and the residue is taken up in chloroform (c = 0.4 M with respect to benzoic acid). The organic phase is washed twice with water and once with saturated NaCl solution. After drying over magnesium sulphate, the solvent is removed and the crude product is purified by flash column chromatography on silica gel (PE/EE 10:1) and/or recrystallised from n hexane.

1.2.5 L-Alanine decylester ammoniumtosylat SI-4

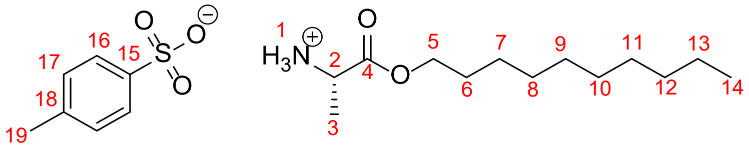

SI-4

According to GP 1, L-alanine (15.23 g, 170.94 mmol, 1.0 eq.), 1-decanol (25.70 g, 162.40 mmol, 0.95 eq.), and *p*-toluenesulfonic acid monohydrate (35.77 g, 188.04 mmol, 1.1 eq.) are reacted in 650 ml toluene. After recrystallisation, L alanine decyl ester ammonium tosylate **SI-4** (49.00 g, 122.02 mmol, 71%) is obtained as a colorless solid.

^{1}H-NMR (500 MHz, $CDCl_3$, 300 K): δ = 8.17 (bs, 1-H_3), 7.76 (d, 16-H_2), 7.13 (d, 17-H_2), 4.03, 3.99 (m, 2-H, 5-H_2), 2.35 (s, 19-H_3), 1.53 (m, 6-H_2), 1.45 (d, 3-H_2), 1.31-1.24 (m, 7-H_2, 8-H_2, 9-H_2, 10-H_2, 11-H_2, 12-H_2, 13-H_2), 0.89 (t, 14-H_3) ppm.
$^3J_{2,3}$ = 7.1 Hz, $^3J_{13,14}$ = 6.7 Hz, $^3J_{16,17}$ = 7.8 Hz.

^{13}C-NMR (125 MHz, $CDCl_3$, 300 K): δ = 170.1 (4-C), 141.7 (15-C), 140.4 (18-C), 128.9 (17-C), 126.2 (16-C), 66.5 (5-C), 49.2 (2-C), 32.0 (12-C), 29.67, 29.62, 29.42, 29.35, 28.38 (6-C, 8-C, 9-C, 10-C, 11-C), 25.8 (7-C), 22.8 (13-C), 21.4 (19-C), 16.0 (3-C), 14.2 (14-C) ppm.

1.2.6 *N*-(4-Ethynylbenzoyl)-L-Alanine decylester SI-5 (2)

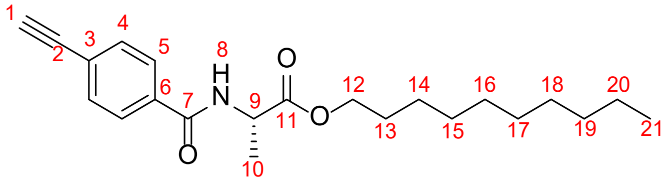

SI-5 (2)

According to GP-2, 4-ethynylbenzoic acid **SI-2** (10.00 g, 68.4 mmol, 1.0 eq.), carbonyldiimidazole (12.20 g, 75.27 mmol, 1.1 eq.) and L-alanine decyl ester ammonium tosylate **SI-4** (27.75 g, 69.1 mmol, 1.01 eq.) are reacted in 750 ml THF and 500 ml DCM.

After flash column chromatographic purification on silica gel and recrystallisation, N-(4-ethynylbenzoyl)-L-alanine decyl ester **SI-7** (17.00 g, 47.5 mmol, 69%) is obtained as colorless needles. The spectroscopic data are in agreement with the literature.[6]

R_f (PE/EE 3:1) = 0.19

^{1}H-NMR (500 MHz, $CDCl_3$, 300 K): δ = 7.76 (d, 5-H_2), 7.54 (d, 4-H_2), 6.77 (bd, 8-H), 4.77 (m, 9-H), 4.18 (m, 12-H_2), 3.19 (s, 1-H), 1.66 (m, 13-H_2), 1.52 (d, 10-H_3), 1.35-1.26 (m, 14-H_2, 15-H_2, 16-H_2, 17-H_2, 18-H_2, 19-H_2, 20-H_2), 0.88 (t, 21-H_3) ppm.

$^3J_{4,5}$ = 8.2, $^3J_{8,9}$ = 7.0, $^3J_{9,10}$ = 7.1, $^3J_{20,21}$ = 6.9 Hz.

^{13}C-NMR (125 MHz, $CDCl_3$, 300 K): δ = 173.4 (11-C), 166.0 (7-C), 134.1 (3-C), 132.4 (4-C), 127.1 (5-C), 125.7 (6-C), 82.9 (2-C), 79.7 (1-C), 66.0 (12-C), 48.8 (9-C), 32.0 (19-C), 29.6, 29.4, 29.3, 28.7, 25.9, 22.8 (13-C, 14-C, 15-C, 16-C, 17-C, 18-C, 20-C), 18.9 (10-C), 14.2 (21-C) ppm.

1.2.7 Decanediol bi-*L*-valinate SI-9

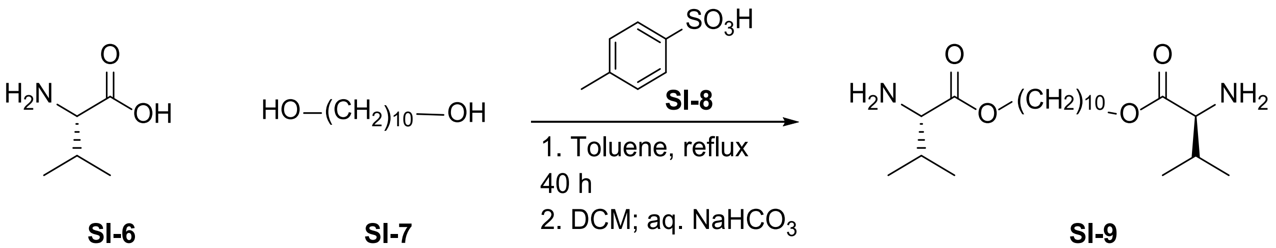

SI-6 **SI-7** **SI-9**

6.00 g (97%; 33.39 mmol; 1 eq) of 1,10-decanediol **SI-7**, as well as 18.42 g (96.84 mmol; 2.9 eq) of p-toluenesulfonic acid **SI-8** and 8.02 g (68.46 mmol; 2.05 eq) of L-valine **SI-6** are suspended in 200 ml of toluene in a round bottom flask. A Dean-Stark apparatus is added on top of the flask and the mixture is heated to reflux for 16 h. After completion of the reaction (verified by TLC), the toluene is removed in vacuo. The remaining white solid is dissolved in EE/aq. $NaHCO_3$. The phases are separated, the organic phase is washed with brine, dried and the solvent removed in vacuo. The product is obtained as 12.20 g (32.75 mmol; 98%) of a viscous, colourless oil, which can be used without further purification.

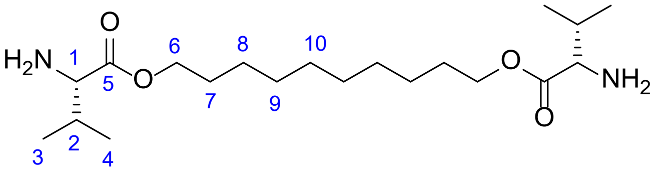

SI-9

^{1}H-NMR ($CDCl_3$, 300 MHz; 300 K) δ = 4.06 (m; 4H; 6-H), 3.23 (d; *J*=4.9 Hz; 2H; 1-H), 1.98 (m; 2H; 2-H), 1.59 (pq; *J*=6.9 Hz; 4H; 7-H), 1.44-1.20 (m; 10H; 8-H+9-H+10-H), 0.93 (d; *J*=6.9 Hz; 6H; 4-H), 0.86 (d; *J*=6.9 Hz; 6H; 3-H) ppm.

^{13}C-NMR ($CDCl_3$, 75 MHz, 300 K) δ = 175.46 (6-C), 64.37 (7-C), 59.93 (2-C), 32.13 (3-C), 28.25 (8-C), 22.50 (9-C), 19.27 (4-C/5-C), 17.17 (4-C/5-C) ppm.

APCI-MS ($C_{15}H_{30}N_2O_4$) m/z $[M+H]^+$: calculated: 303.228 found: 303.228.

ORD ($CHCl_3$): $[\alpha]_{589}^{20}$ =16.65 $[\alpha]_{579}^{20}$ =16.15

$[\alpha]_{546}^{20}$ =20.65 $[\alpha]_{436}^{20}$ =40.40

$[\alpha]_{405}^{20}$ =49.35 $[\alpha]_{365}^{20}$ =72.20

1.2.8 Bi-acetylene crosslinker SI-10 (CL-C10)

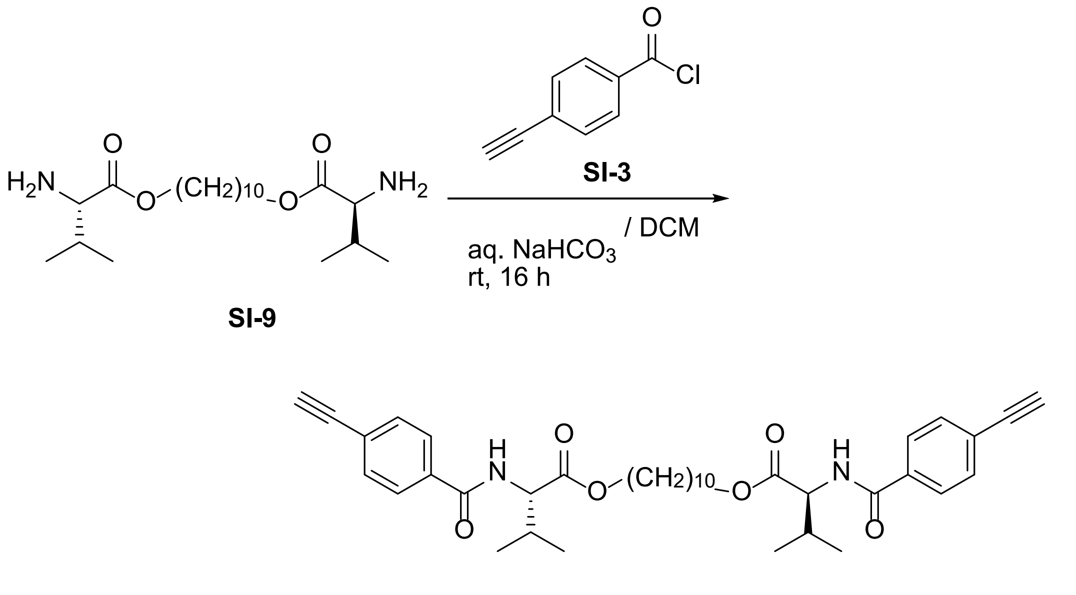

SI-10 (CL-C10)

The reaction is carried out under SCHOTTEN-BAUMANN conditions. To this end, 0.500 g (1.34 mmol, 1 eq) of the decanediol bi-L-valinate **SI-9** are dissolved in 30 ml of dichloromethane in a round-bottom flask. Then 60 ml of saturated aq. $NaHCO_3$ solution are added. A dropping funnel with a solution of 0.508 g (3.09 mmol, 2.3 eq) of p-ethinylbenzoyl chloride **SI-3** in 30 ml of dichloromethane and a gas bubbler are added and the solution is slowly dropped into the stirred reaction mixture. The mixture is left to stir at ambient temperature until the gas formation has subsided, which is the case after approx. 16 h. The phases are separated, the organic phase is dried and the solvent is removed in vacuo. The crude product, which is obtained as a brown crystalline solid, is purified by column chromatography with a 3:1 mixture of hexane and ethyl acetate followed by recrystallisation from hexane/EE yielding 0.752 g (1.19 mmol; 89 %) of product as a white, crystalline solid.

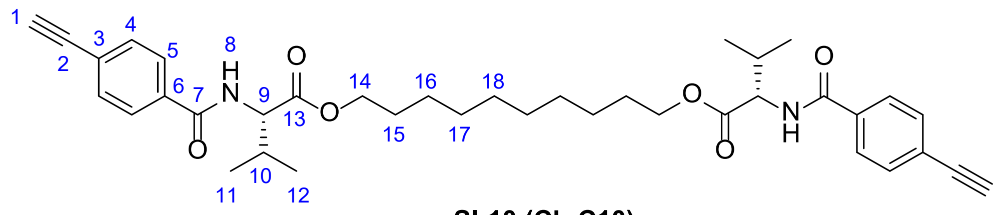

SI-10 (CL-C10)

TLC: R_f = 0,16 (hexane:EE 3:1)

^{1}H-NMR ($CDCl_3$, 300 MHz, 300 K) δ = δ= 7.76 (d; *J*=8.5 Hz; 4H; 5-H), 7.56 (d; *J*=8.5 Hz; 4H; 4-H), 6.63 (d; *J*=8.5 Hz; 2H; 8-H), 4.76 (dd; *J*=8.5/4.7 Hz; 2H; 9-H), 4.23-4.10 (m; 4H; 14-H), 2.34-2.23 (m; 2H; 10-H), 1.66 (pq; *J*=7.0 Hz; 4H; 15-H), 1.40-1.24 (m; 12H; 16-H+17-H+18-H), 1.00 (pt; *J*=14.5 Hz; 12H; 11-H+12-H) ppm.

^{13}C-NMR ($CDCl_3$, 75 MHz, 300 K) δ = 172.18 (13-C), 166.44 (7-C), 134.20 (6-C), 132.33 (4-C), 127.02 (5-C), 125.63 (3-C), 82.76 (2-C), 79.56 (1-C), 65.61 (14-C), 57.50 (9-C), 31.71 (15-C), 29.35 (17-C), 29.11 (18-C), 28.54 (10-C), 25.84 (16-C), 18.99 (12-C), 17.93 (11-C) ppm.

**APCI-MS** ($C_{38}H_{48}N_2O_6$) m/z $[M+H]^+$:	calculated:	629.359 m/z (M+H).
	found:	629.359 m/z (M+H).
**EA:**	calculated:	C 72.58 %; H 7.69 %; N 4.46 %.
	found:	C 72.70 %; H 7.84 %; N 4.36 %.

ORD ($CHCl_3$)**:** $[\alpha]_{589}^{20}$ =28.75 $[\alpha]_{579}^{20}$ =28.75 $[\alpha]_{546}^{20}$ =34.75 $[\alpha]_{436}^{20}$ =63.20

1.2.9 Ethinyl-terminated PDMS-crosslinkers Si-13 (CL-Si65) and SI-14 (CL-Si400)

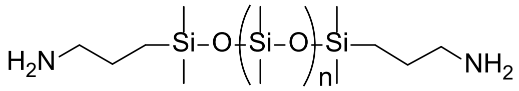

n=65: **SI-11; Gelest** Product Code: DMS-A21; CAS No: 106214-84-0
n=400: **SI-12: Gelest** Product Code: DMS-A32; CAS No: 106214-84-0

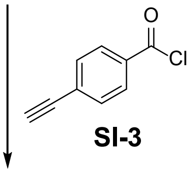

SI-3

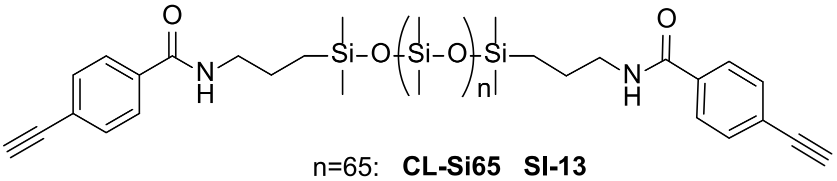

n=65: **CL-Si65 SI-13**
n=400: **CL-Si400 SI-14**

The aminopropyl-terminated polydimethylsiloxanes DMS-A21 **SI-11** (M_w = 5000 g/mol) and DMS-A32 **SI-12** (M_w = 30 000 g/mol) were purchased from *Gelest Inc.* (Morrisville, USA) and used without further purification. The SCHOTTEN-BAUMANN reaction is carried out by dissolving 10 g (2,00 mmol of DMS-A21 **SI-11**; 0,33 mmol of DMS-A32 **SI-12**; 1 eq respectively) of starting material in 200 ml of DCM and placing the solution in a round-bottom flask together with 150 ml of saturated aq. $NaHCO_3$ solution. A dropping funnel with a solution of 1.344 g (16.00 mmol, 8 eq) of *p*-ethinylbenzoyl chloride **SI-3** for DMS-A21, or 0.366 g (4,00 mmol, 12 eq) of *p*-ethinylbenzoyl chloride **SI-3** for DMS-A32, in 50 ml of dichloromethane and a gas bubbler are added to the flask and the solution is slowly dropped into the stirred reaction mixture. The mixture is left to stir at ambient temperatures for 16 h, after which the gas formation has ended. The phases are separated and the organic phase concentrated to approximately 75 ml. The polymer solution is slowly dropped into an ERLENMEYER flask containing 750 ml of methanol under stirring. The resulting suspension of polymer droplets in methanol is left to separate for 90 minutes, after which two separate phases are obtained, the bottom one consisting of polymer. The solvent phase is decanted off, the polymer phase is dissolved in 70 ml of DCM and the procedure is repeated. The obtained oil is dissolved in 200 ml of DCM and a dry load for chromatography is created by adding silica gel and removing the solvent in vacuo. The dry load is added to the top of a short silica column and the product is eluted with diethyl ether. The product is obtained as a pale yellowish oil after removing the solvent in vacuo. The success of the reaction is verified by ^{1}H-NMR spectroscopy, which shows the proton signals of the CH_2 groups, as well as the aromatic "doublets". Comparison with ^{1}H-NMR spectra of the starting materials indicate a full conversion in both cases. The molecular weight of the starting materials and the products were analysed by NMR end-group analysis and compared to ensure that no chain degradation had taken place. The ratio of the intensity of the backbone signal compared to the 3-H-signal of each substance was identical within experimental error.

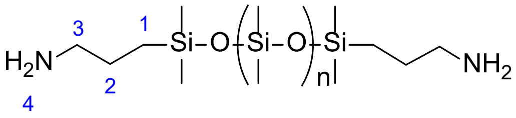

n=65: **DMS-A21 SI-11**
n=400: **DMS-A32 SI-12**

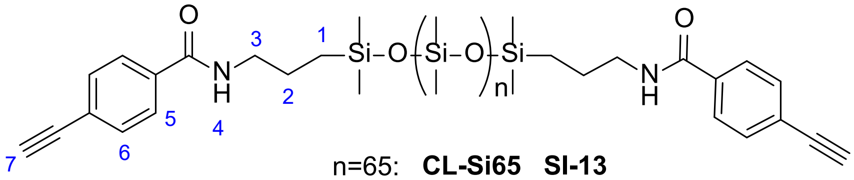

n=65: **CL-Si65 SI-13**
n=400: **CL-Si400 SI-14**

**Yield:**	**CL-SI65**	**SI-13:** 8.83 g (1,67 mmol; 83,5 %).
	**CL-SI400**	**SI-14:** 9.08 g (0,30 mmol; 89,9%).

^{1}H-NMR (500 MHz, $CDCl_3$, 300 K)

Starting materials: δ= 2.67 (t; *J*=7.0 Hz; 4H; 3-H), 1.51-1.40 (m broad; 8H; 2-H+4-H), 0.55-0.52 (m; 4H; 1-H), 0.16-0.01 (s broad; *backbone*).

Products: δ= 7.54 (d; *J*=8.48 Hz; 4H; 5-H), 7.54 (d; *J*=8.48 Hz; 4H; 6-H), 6.15 (s broad; 2H; 4-H), 3.48-3.40 (m; 4H; 3-H), 3.18 (s; 2H; 7-H), 1.71-1.60 (m; 4H; 2-H), 0.63.0.58 (m; 4H; 1-H), 0.16-0.01 (s broad; *backbone*).

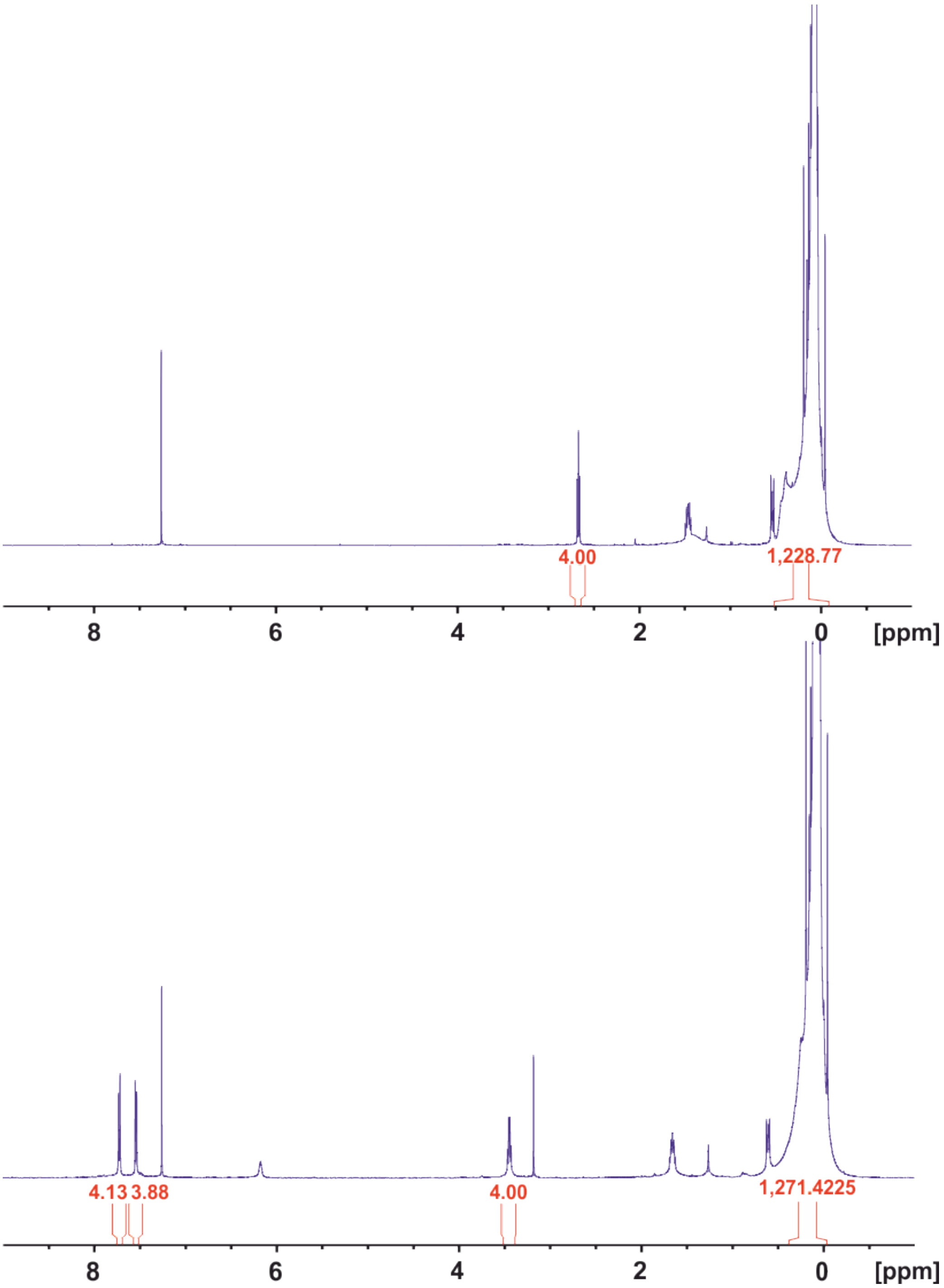

Figure S1 ^{1}H-NMR spectra of DMS-A21 (**SI-11**; above) and **SI-13** (**CL-SI65**) (below).

1.3 Polymerstick synthesis procedure

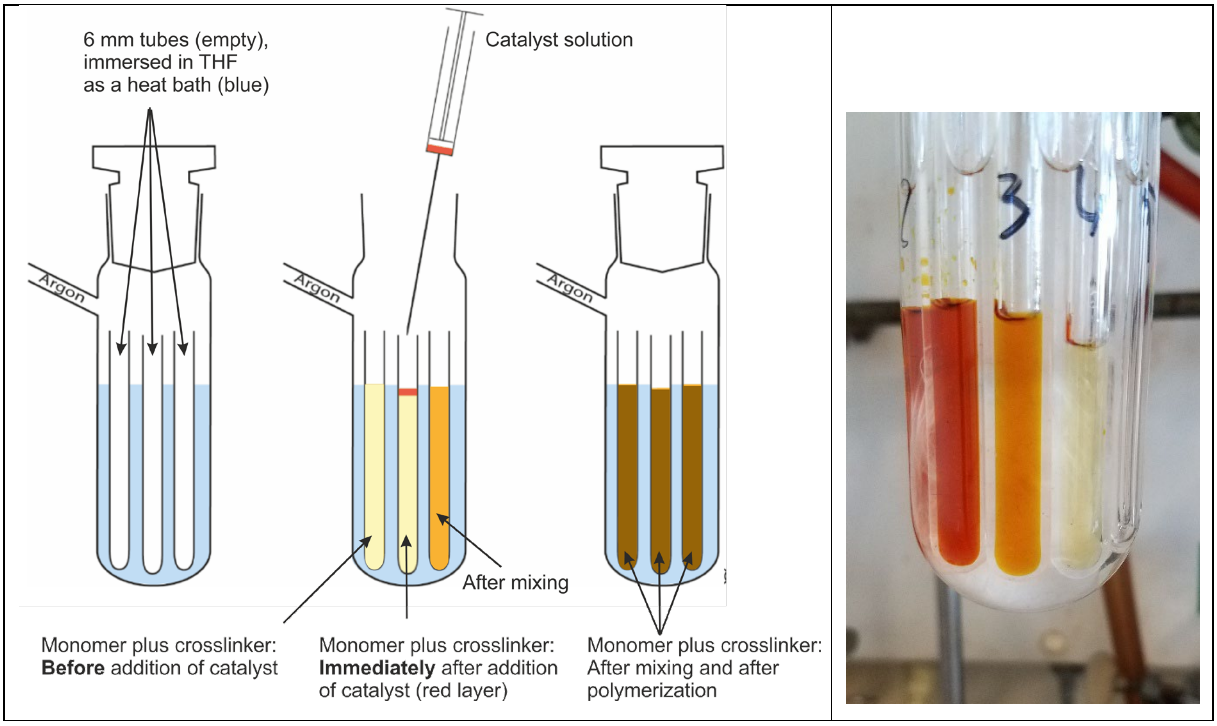

Figure S2 **Left:** Schematic representation of different stages of the polymerization procedure. **Right:** The real apparatus showing different stages of the gelation process.

All polymerizations were carried out in 7 cm hydrophobized glass tubes, fused on one side, with an inner diameter of 6 mm within flame dried Schlenk tubes under dry argon atmosphere. The hydrophilization was carried out by treating the tubes with a 1:1 (v:v) mixture of dimethyldichlorosilane and trimethylchlorosilane for 16 h. The tubes were then washed with DCM and stored at 50°C in a drying oven until further use.

All solvents were dried, freshly distilled and degassed (by using the freeze, pump and thaw technique) prior to use. A vinyl rhodium complex was used as the catalyst for all polymerizations.[2-3, 7] For the preparation of **1**/*ent*-**1** see ref [2] for **3**/*ent*-**3** and **4**/*ent*-**4** see ref[3]. For the Ala-derivative **2** see this SI (1.2.5).

All polymerizations were carried out in THF and initiated with a catalyst solution of 0.01 mol/L in THF/Et_2O (with a density of approx. 0.8 g/mL). Stick properties can be tuned by varying the concentration of polymerizable compounds (w% monomer (M) + crosslinker (CL) in solution), the amount of crosslinker in relation to the total amount of polymerizable material (x(CL)) and the monomer to initiator ratio M:I (with the crosslinker also being considered a "monomer" for this metric). One must keep in mind that the amount of catalyst solution also affects the concentration of polymerizable compounds when calculating their w% in the resulting solution. As an example Table S1 shows the amounts of reagents used for the preparation of the sticks **142A** and **142B** (Valine based monomer **1** with **CL-Si65**; 25w% M + CL in solution; x(CL) = 0.1 and M:I = 250). Table S2 shows the reagents and synthesis parameters for all synthesized sticks. A typical stick synthesis procedure is described below.

Table S1 Reagents and solvents used for sticks 142A and 142B.

Monomer X	CL-Si65	abs. deg. THF	catalyst solution
118.8 mg	181.2 mg	0.888 ml	0.137 ml

To prepare the sticks, the glass tubes are fixed in an upright position in a 50 ml Schlenk tube with glass wool, the vessel is sealed with a glass stopper, and the entire reaction vessel is heated three times under high vacuum to remove surface bound water. Then, the space between the tube and the wall of the Schlenk vessel is flooded with abs. THF to ensure efficient heat transfer and the reaction vessel is tempered in a heating bath at 30°C. In a separate Schlenk vessel, a 50 w% stock solution of the crosslinker in abs. deg. THF is prepared.

When synthesizing a batch of sticks, a separate solution is prepared for each individual stick. The solid monomer is weighed into a 5 ml glass vial, the vessel is sealed and carefully flushed with argon via a cannula. The crosslinker solution and the rest of the required abs. deg. THF are added. The vials are shaken until a completely homogeneous solution is formed.

The solutions are transferred to the tubes with a syringe in an argon countercurrent. A syringe with at least 1 ml additional volume and a needle that reaches the bottom of the tubes is chosen for this. As soon as a tube is filled, the catalyst solution is added to the tube, also in countercurrent, with a second syringe. Then, all the liquid in the tube is immediately and quickly drawn back onto the first syringe that was used to transfer the monomer solution. The plunger of the syringe is withdrawn completely (without pulling it out of the syringe altogether), causing at least 1 ml of argon to flow through the solution and mix it. The solution is transferred back into the tube and the mixing procedure is repeated two more times. The now homogeneous yellow solution gradually turns darker yellow to orange and solidifies within 10-30 min after initiation. The reaction is always carried out for at least 14 h at 30°C.

For the workup, the small glass tubes are removed from the Schlenk tube and stored for one week protected from light but unsealed. After this time, the solvent has completely evaporated and the contracted sticks can be carefully removed from the tubes. To remove the catalyst, as well as any remaining monomer or oligomers, the rods are swollen individually, in sealed 100 ml screw-top vessels in DCM. After 24 h the solvent is exchanged, and after another 24 h replaced by THF. After a final 24 h,

the solvent is removed and the swollen sticks are placed immediately, but with great care, back into hydrophobized glass tubes with an appropriate inner diameter (≥1cm). The tubes are then loosely sealed with aluminum foil, which is then perforated several times. This ensures that drying occurs slowly enough to prevent stress cracks from forming in the gel. After another 5-7 days, drying is complete. **Drying the sticks slowly is essential to prevent them from cracking.**

1.4 Stick composition and synthesis parameters

Table S2 Synthesis parameters of all sticks used in NMR alignment experiments.

Stick (pair)	Monomer	Crosslinker (CL)	w% M+CL in solution	x(CL)	M:I[a)]	w% PPA in dry stick
**16**	**1**	CL-C10	21.7	0.10	500	100
**42**	**1**	CL-C10	27.5	0.10	500	100
**43**	**1**	CL-C10	25.0	0.10	500	100
**44**	**1**	CL-C10	22.5	0.10	500	100
**45**	**1**	CL-C10	20.0	0.10	500	100
**46**	**1**	CL-C10	17.5	0.10	500	100
**47**	**1**	CL-C10	15.0	0.10	500	100
**48**	**1**	CL-C10	10	0.10	500	100
**133**	**1**	CL-Si400	42.5	0.05	250	19.5
**137/138**	**1**	CL-C10	25	0.10	500	100
**142A/142B**	**1**	CL-Si65	25	0.10	250	39.6
**143/144**	**1**	CL-Si65	27.5	0.10	500	39.6
**151/152**	*ent*-**1**	CL-Si65	27.5	0.10	500	39.6
**153/154**	**1**	CL-Si65	27.5	0.10	500	39.6
**149/150**	**1**	CL-Si65	27.5	0.10	500	39.6
**155/156**	**1**	CL-Si65	27.5	0.10	500	39.6
**167/168**	**1**	CL-Si400	30	0.05	500	19.5

**173/185**	**1**/*ent*-**1**	CL-Si65	27.5	10	500	39.6
**176/180**	**1**/*ent*-**1**	CL-Si65	27.5	0.10	500	39.6
**179/181**	**1**	CL-Si65	27.5	0.10	500	39.6
**184**	**1**	CL-Si65	27.5	0.10	500	39.6
**187/190**	**1**/*ent*-**1**	CL-Si65	30	0.05	250	19.5
**188/191**	**1**/*ent*-**1**	CL-Si400	27.5	0.10	500	39.6
**241**	**3**	CL-Si65	27.5	0.10	250	39.6
**245/246**	**4**	CL-Si65	27.5	0.10	250	39.6
**282/283**	**1**	CL-Si65	27.5	0.10	500	39.6
**284/285**	**1**	CL-Si65	27.5	0.10	500	39.6
**286/287**	**1**	CL-Si65	27.5	0.10	500	39.6
**288/289**	**1**	CL-Si65	27.5	0.10	500	39.6

a) **M**onomer to **I**nitiator ratio

2 NMR studies

2.1 Sample preparation

The gels were prepared by cutting the sticks to a length of approx. 2 cm. The gel stick was then added to a standard 5mm NMR tube and pushed gently to the bottom of the tube with a thin steel rod. A stock solution of the analyte in the respective solvent was prepared and added to the NMR tube. The gel stick was fixed in position with the metal rod until isotropic swelling locked it in place, which usually occurred in less than 5 minutes. The metal rod was removed and the NMR tube was sealed with a rubber stopper. It was then stored at RT, protected from direct light until swelling was complete. This was monitored via regular ^{2}H-image NMR measurements,[8] with swelling being deemed complete when the quadrupolar splitting was homogenous along the entire sample.

2.2 Measurement conditions

All spectra of the analytes in isotropic and oriented samples were recorded on a 500 MHz spectrometer (Bruker DRX-500) with a triple resonance inverse probe equipped with a z-gradient and a BCU extreme. All measurements were carried out without sample spinning. The total coupling constants ($^{1}T_{CH}$) and scalar coupling constants ($^{1}J_{CH}$) were measured by CLIP-HSQC experiments (INEPT delay 145 Hz).[9] A total of 8 k data points were sampled in the direct dimension over an appropriate spectral width. In the indirect dimension 256 data points were acquired over an appropriate spectral width. The spectra were processed using a $\pi/2$ shifted squared sine bell in both dimensions. No zero filling was applied in F2, but by a factor of four in the indirect F1 dimension. For each coupling the corresponding traces of the CLIP-HSQC spectra were extracted, subjected to an inverse Fourier transformation and processed by zero filling to 32 k points. A copy of the trace was overlaid with the original one and shifted until the difference signal reached a minimum. Temperature dependent spectra were measured in steps of 2.5 Kelvin (300 K to 250 K; 252.5 K to 317.5 K; 320 K to 300 K) with an equilibration time interval of 180 seconds at each temperature.

NMR spectra of newly synthesized compounds were recorded with DRX 300, AC 300 and DRX 500 spectrometers from Bruker. The 1D and 2D NMR spectra were analyzed using the TopSpin 3.5.pl7 and 4.0.6 software from Bruker. The respective measuring frequency, the solvent used and the measuring temperature are placed in brackets in front of the spectroscopic data. The chemical shifts are given in ppm and were calibrated to the signal of the solvent used for the 1H and 13C NMR spectra:

$CDCl_3$ (δ ($CHCl_3$)) = 7.260 ppm, δ ($CDCl_3$) = 77.16 ppm)
CD_3OD (δ (CHD_2OD)) = 3.310 ppm, δ (CD_3OD) = 49.00 ppm)
DMSO-d_6 (δ ((CHD_2)SO(CD_3)) = 2.500 ppm, δ ((CD_3)SO(CD_3)) = 39.52 ppm)

The fine structures of the proton signals are labeled with the following abbreviations:
s for singlet, *d* for doublet, *t* for triplet, *q* for quartet, *quint* for quintet, *m* for multiplet, *dd* for double doublet, *dt* for double triplet, *ddd* for triple doublet. Apparent multiplicities, which result from an overlap of signals where the distance and a coupling constant coincide (pseudo-multiplets), are marked with a

p in front. If the overlap of two doublets leads to an apparent triplet, for example, this is marked with *pt*. The chemical shift of the ^{13}C signals was taken from the broadband decoupled spectra and their assignment was made from the DEPT and 2D spectra. The numbering in the diagrams does not correspond to the IUPAC rules and is merely for the sake of clarity.

2.3 ^{2}H-Spectra of Sticks S42-S48 (Data for Figure 1 in Manuscript):

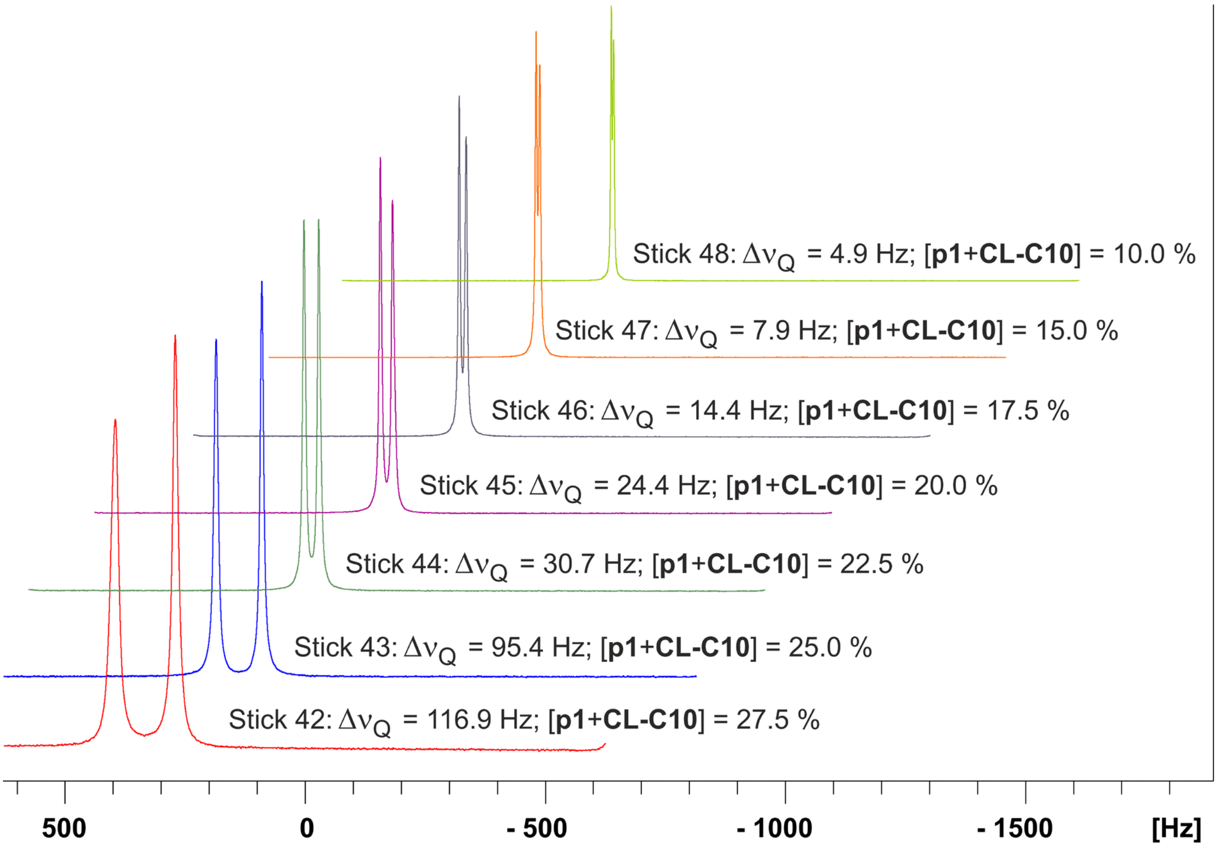

Figure S3. Quadrupolar splitting of p1-CL-C10 sticks at different concentrations (Data for Figure 1A in the manuscript).

2.4 Orientation and differentiation results

Table S3 Results of the NMR alignment experiments by stick.

Stick (pair)	Monomer	Crosslinker	$\Delta\nu_Q$	Analyte m(+) [mg] / m(-) [mg][a)]	GCB	GCB Error
**16**	**1**	Cl-C10	48	**(-)-IPC**	-	-
**133**	**1**	CL-Si400	11.5	**(-)-IPC**	-	-
**137/138**	**1**	CL-C10	61/61	**IPC**	0.620	0.04
**142A/142B**	**1**	CL-Si65	20/20	**IPC** 31.5/31.8	0.515	0.06
**143/144**	**1**	CL-Si65	26/26	**IPC**	0.58	0.054
**149/150**	**1**	CL-Si65	48/63	**Camphor** 28.3/27.2	0.787	0.089
**151/152**	*ent*-**1**	CL-Si65	36	**IPC**	0.559	0.048
**153/154**	**1**	CL-Si65	55/65	**Pinene** 34.0/35.0	0.964	0.118
**155/156**	**1**	CL-Si65	40/50	**Menthol** 36.0/32.2	0.714	0.206
**167/168**	**1**	CL-Si400	15/14.8	**IPC**	0.789	0.056
**173/185**	**1**/*ent*-**1** 34.0/28.4[b)]	CL-Si65	61/76	**Strychnine**	0.979	0.006
**176/180**	**1**/*ent*-**1** 32.0/27.7[b)]	CL-Si65	85/86	**Sparteine**	0.902	0.053
**179/181**	**1**	CL-Si65	53/52	**IPC**	0.545	0.045
**184**	**1**	CL-Si65	54	**(-)-IPC**	-	-
**187/190**	**1**/*ent*-**1** 23.0/21.0[b)]	CL-Si65	56/64	**Cholesterol**	0.777	0.087
**188/191**	**1**/*ent*-**1** 20.3/19.2[b)]	CL-Si400	13.5/14.5	**(-)-Perilla acid**	0.632	0.091
**241**	**3**	CL-Si65	3.9	**(+)-IPC**	-	-
**245/246**	**4**	CL-Si65	299	**IPC**	0.918	0.022
**247/248**	**4**	CL-Si65	369/357	**Camphor** 27.0/18.8	0.781	0.21

**282/283**	**1**	CL-Si65	10.7/9.7	**IPC (DCM)**	0.891	0.03
**284/285**	**1**	CL-Si65	2.2	**IPC (THF)**	0.815	0.041
**286/287**	**1**	CL-Si65	-	**IPC (Toluene)**	-	-
**288/289**	**1**	CL-Si65	-	**IPC (TCE)**	-	-

a) In case both enantiomers of the analyte are available the first number denotes the mass of the (+)-enantiomer, the second corresponds to the mass of the (-)-enantiomer.
b) In case only one enantiomer is available, the first number corresponds to the sample with the L-polymer and the second number to the mass of the D-polymer.

2.5 RDCs of measured samples by analyte

2.5.1 IPC

Table S4 Numbering of the nuclei, assignment of the chemical shifts δ and scalar $^1J_{CH}$ couplings for the enantiomers of IPC. *s* = syn to the methylene bridge; *a* = anti

		δ (^{13}C) [ppm]	δ (^{1}H) [ppm]	$^1J_{CH}$ [Hz]
C1	H1	47.8	1.79	139.5
C2	H2	47.7	1.93	125.8
C3	H3	71.6	4.06	139.6
C4	H4s	39.0	2.50	126.0
C4	H4a	39.0	1.71	126.0
C5	H5	41.8	1.93	143.7
C6	-	38.2	-	-
C7	H7s	34.4	2.37	135.4
C7	H7a	34.4	1.04	136.0
C8	H8	27.7	1.21	123.0
C9	H9	23.7	0.91	124.6
C10	H10	20.7	1.13	124.4

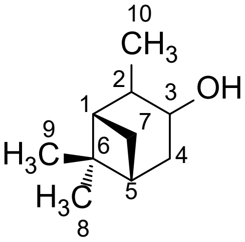

(+)-IPC

Table S5 RDCs of IPC in the indicated sticks. For stick pairs in which both enantiomers were measured, the (+) enantiomer was measured in the first-mentioned stick and the (-) enantiomer in the second-mentioned stick.

Stick-Nr			16	45	137	138	142A	142B	143	144
		$^1J_{CH}$ [Hz]	$^1D_{CH}$ [Hz]	$^1D_{CH}$ [Hz]	$^1D_{CH}$ [Hz]	$^1D_{CH}$ [Hz]	$^1D_{CH}$ [Hz]	$^1D_{CH}$ [Hz]	$^1D_{CH}$ [Hz]	$^1D_{CH}$ [Hz]
C1	H1	139.5	6.50	-13.90	-3.35	14.70	-2.20	7.05	-1.75	8.05
C2	H2	125.8	-7.55	14.90	-5.45	-12.05	-0.45	-1.95	-0.65	-2.30
C3	H3	139.6	-11.55	-12.75	-16.10	-19.65	-5.50	-9.05	-6.10	-10.65
C4	H4s	126	-8.70	-7.00	-20.00	-10.60	-8.50	-2.25	-9.35	-3.35

	H4a	126	1.35	-0.60	4.60	1.10	5.15	0.75	4.95	1.35
C5	H5	143.7	1.25	3.05	10.05	5.55	3.85	1.75	3.55	2.00
C6										
C7	H7s	135.4	-2.85	-2.30	-2.85	-6.85	-4.60	-4.95	-3.65	-5.55
	H7a	136	2.50	2.90	13.55	2.20	7.55	4.00	7.85	5.05
C8	H8	123	-1.60	-1.15	-3.50	-1.30	0.10	0.30	-0.30	-0.10
C9	H9	124.6	3.40	4.00	5.45	6.00	1.80	2.60	1.90	3.10
C10	H10	124.4	0.40	1.10	-0.05	0.30	-0.50	0.85	-0.20	1.10

	**Stick-Nr**		**151**	**152**	**167**	**168**	**245**	**256**	**282**	**283**
		$^{1}J_{CH}$ [Hz]	$^{1}D_{CH}$ [Hz]	$^{1}D_{CH}$ [Hz]	$^{1}D_{CH}$ [Hz]	$^{1}D_{CH}$ [Hz]	$^{1}D_{CH}$ [Hz]	$^{1}D_{CH}$ [Hz]	$^{1}D_{CH}$ [Hz]	$^{1}D_{CH}$ [Hz]
C1	H1	139.5	10.45	-3.20	-0,05	-0,05	-0.90	1.70	-1.15	2.65
C2	H2	125.8	-3.80	-1.50	0,30	0,30	-2.45	-0.60	-1.65	-1.50
C3	H3	139.6	-13.95	-8.50	-3,75	-3,75	-4.05	-4.90	-7.80	-8.85
C4	H4s	126	-4.60	-12.70	-4,50	-4,50	-4.60	-5.00	-9.45	-5.40
	H4a	126	1.20	6.80	3,05	3,05	-1.40	1.00	4.15	1.80
C5	H5	143.7	2.70	4.80	1,00	1,00	-8.35	-4.65	1.20	0.55
C6										
C7	H7s	135.4	-7.60	-4.55	-1,85	-1,85	10.50	3.00	-2.00	-3.00
	H7a	136	6.30	10.95	5,70	5,70	9.25	8.40	8.45	6.80
C8	H8	123	-0.25	-1.05	0,25	0,25	-4.40	-2.50	-1.25	-0.90
C9	H9	124.6	4.25	3.20	1,00	1,00	1.25	1.45	1.85	2.20
C10	H10	124.4	1.50	-0.35	0,35	0,35	4.40	3.40	0.10	0.90

	**Stick-Nr**		**284**	**285**
		$^{1}J_{CH}$ [Hz]	$^{1}D_{CH}$ [Hz]	$^{1}D_{CH}$ [Hz]
C1	H1	139.5	-2.05	3.15
C2	H2	125.8	0.60	0.55
C3	H3	139.6	-5.80	-9.00
C4	H4s	126	-7.45	-4.05
	H4a	126	4.85	3.15
C5	H5	143.7	2.05	1.30
C6				
C7	H7s	135.4	-4.95	-7.30
	H7a	136	7.55	6.60
C8	H8	123	0.00	0.15
C9	H9	124.6	1.15	2.10
C10	H10	124.4	-0.60	0.35

2.5.1.1 Temperature series

Stick 179 (+)		$^1J_{CH}$ [Hz]	280 K $^1D_{CH}$ [Hz]	285 K $^1D_{CH}$ [Hz]	290 K $^1D_{CH}$ [Hz]	295 K $^1D_{CH}$ [Hz]	300 K $^1D_{CH}$ [Hz]	305 K $^1D_{CH}$ [Hz]	310 K $^1D_{CH}$ [Hz]	315 K $^1D_{CH}$ [Hz]	320 K $^1D_{CH}$ [Hz]
C1	H1	139.5	3.10	-1.25	-2.05	-2.95	-4.05	-3.6	-4	-3.6	-2.9
C2	H2	125.8	-2.60	-1.3	-1.4	-2.15	-3.3	-3.1	-3.55	-3.6	-3.5
C3	H3	139.6	-1.95	-2.95	-5.9	-9	-11.15	-11.8	-11.8	-11.35	-10.65
C4	H4s	126	-2.75	-4.75	-8.95	-13.25	-15.6	-17.25	-17.35	-16.8	-15.5
	H4a	126	-0.85	1.1	2.75	4.7	7.05	8.4	8.2	7.9	7.4
C5	H5	143.7	-10.65	-7.45	-3.05	1.5	4.4	5.5	5.2	4.2	3.6
C6											
C7	H7s	135.4	5.20	4.2	1.7	-1.3	-4	-5.15	-5	-4.4	-3.8
	H7a	136	8.65	8.85	10.25	11.6	12.75	12.85	13.7	13	12.35
C8	H8	123	-3.75	-2.95	-3.45	-2.55	-1.75	-2	-1.95	-2.15	-2.15
C9	H9	124.6	-0.30	0.6	1.35	2.45	3.2	3.55	3.55	3.35	3.3
C10	H10	124.4	2.60	2.55	1.6	0.35	-0.45	-0.85	-0.7	-0.55	-0.35

Stick 181 (-)		$^1J_{CH}$ [Hz]	280 K $^1D_{CH}$ [Hz]	285 K $^1D_{CH}$ [Hz]	290 K $^1D_{CH}$ [Hz]	295 K $^1D_{CH}$ [Hz]	300 K $^1D_{CH}$ [Hz]	305 K $^1D_{CH}$ [Hz]	310 K $^1D_{CH}$ [Hz]	315 K $^1D_{CH}$ [Hz]	320 K $^1D_{CH}$ [Hz]
C1	H1	139.5	5.30	6.25	9.25	11.85	12.65	12.25	11.00	9.65	8.15
C2	H2	125.8	-5.55	-4.85	-5.15	-5.15	-5.15	-5.25	-5.00	-4.70	-4.45
C3	H3	139.6	-2.90	-5.40	-10.30	-14.40	-17.15	-17.95	-17.30	-16.00	-14.90
C4	H4s	126	-5.90	-6.15	-5.50	-5.15	-5.30	-5.55	-5.80	-6.05	-6.40
	H4a	126	5.65	3.10	2.35	1.65	1.00	0.70	0.50	0.35	0.30
C5	H5	143.7	-1.60	-0.20	1.30	2.65	3.20	3.20	3.20	3.05	2.70
C6											
C7	H7s	135.4	-1.60	-2.65	-5.80	-8.35	-9.35	-8.85	-8.00	-6.80	-5.60
	H7a	136		3.15	4.00	5.25	6.25	6.85	7.15	7.10	6.75
C8	H8	123	0.05	-0.20	-0.30	-0.25	-0.45	-0.55	-1.10	-1.15	-1.35
C9	H9	124.6	2.45	1.90	3.05	4.15	4.90	5.05	4.75	4.65	4.25
C10	H10	124.4	-0.25	0.05	0.45	0.95	1.30	1.40	1.40	1.35	1.30

Stick 245 (+)		$^{1}J_{CH}$ [Hz]	280 K $^{1}D_{CH}$ [Hz]	285 K $^{1}D_{CH}$ [Hz]	290 K $^{1}D_{CH}$ [Hz]	295 K $^{1}D_{CH}$ [Hz]	300 K $^{1}D_{CH}$ [Hz]	305 K $^{1}D_{CH}$ [Hz]	310 K $^{1}D_{CH}$ [Hz]	315 K $^{1}D_{CH}$ [Hz]	320 K $^{1}D_{CH}$ [Hz]
C1	H1	139.5	-0.05	0.15	0.40	0.70	0.90	0.90	1.15	1.30	1.40
C2	H2	125.8	-1.10	-1.15	-1.00	-1.30	-1.65	-1.65	-1.70	-2.00	-2.15
C3	H3	139.6	-1.80	-1.90	-1.95	-1.95	-2.10	-2.15	-2.15	-2.20	-2.25
C4	H4s	126	-1.20	-1.95	-1.85	-2.05	-2.30	-2.15	-2.25	-2.40	-2.40
	H4a	126	-2.70	-2.40	-2.15	-2.05	-2.55	-2.15	-2.25	-2.25	-2.35
C5	H5	143.7	-6.05	-6.10	-5.60	-5.50	-5.55	-4.90	-4.90	-4.60	-4.30
C6											
C7	H7s	135.4	7.05	7.15	7.55	7.20	7.35	6.75	6.55	6.45	5.80
	H7a	136	3.65	4.35	4.30	4.55	4.50	4.30	4.20	3.85	3.55
C8	H8	123	-2.80	-2.75	-2.70	-2.55	-2.60	-2.40	-2.30	-2.30	-2.15
C9	H9	124.6	-0.05	0.20	0.40	0.55	0.45	0.40	0.70	0.60	0.50
C10	H10	124.4	3.15	3.15	3.15	3.15	3.00	2.80	2.70	2.55	2.35

Stick 246 (-)		$^{1}J_{CH}$ [Hz]	280 K $^{1}D_{CH}$ [Hz]	285 K $^{1}D_{CH}$ [Hz]	290 K $^{1}D_{CH}$ [Hz]	295 K $^{1}D_{CH}$ [Hz]	300 K $^{1}D_{CH}$ [Hz]	305 K $^{1}D_{CH}$ [Hz]	310 K $^{1}D_{CH}$ [Hz]	315 K $^{1}D_{CH}$ [Hz]	320 K $^{1}D_{CH}$ [Hz]
C1	H1	139.5	1.15	1.40	1.95	2.30	2.20	2.20	2.25	2.00	2.10
C2	H2	125.8	0.25	0.20	0.25	0.20	-0.20	-0.40	-0.45	-0.60	-0.75
C3	H3	139.6	-1.95	-2.45	-2.90	-2.75	-3.05	-2.60	-2.80	-2.55	-2.70
C4	H4s	126	-1.70	-2.15	-2.15	-2.25	-2.75	-2.85	-3.00	-3.45	-3.65
	H4a	126	-0.20	0.10	0.70	0.70	0.65	1.00	1.00	0.80	1.20
C5	H5	143.7	-4.55	-3.95	-3.95	-3.65	-3.00	-2.90	-2.80	-2.55	-2.45
C6											
C7	H7s	135.4	3.05	2.40	2.20	2.20	1.70	1.55	1.10	1.00	0.75
	H7a	136	4.55	4.90	5.25	5.05	5.10	4.80	4.65	4.35	4.35
C8	H8	123	-1.10	-1.50	-0.95	-0.85	-1.15	-1.00	-0.80	-0.75	-0.80
C9	H9	124.6	-0.05	0.30	0.75	0.50	0.85	0.95	0.75	0.65	0.50
C10	H10	124.4	1.65	1.60	1.90	2.10	2.00	1.75	1.55	1.35	1.25

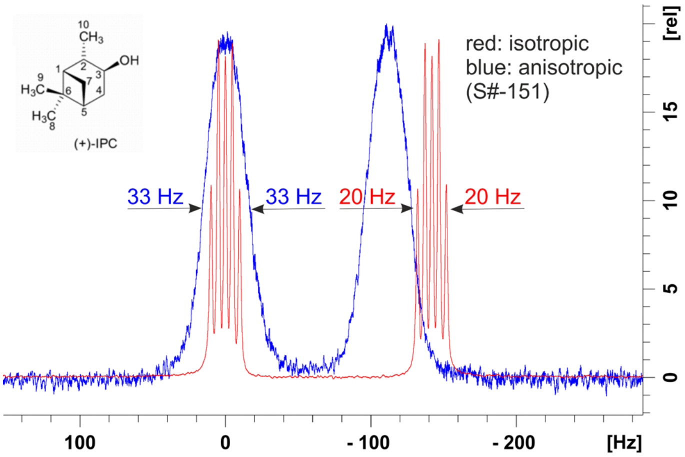

Figure S4. Traces of CLIP-HSQCs of (+)-IPC (C3/H3-crosspeak). Blue: Anisotropic in **S#151**; Red: Isotropic. Both traces were extracted from the HSQCs (same resolution in F2 – 8k); inverse Fourier transformed (ift) – pseudo raw data generated (genfid) then zero-filled to 32k and transformed again without apodisation.

2.5.2 Camphor

Table S6 Numbering of the nuclei, assignment of the chemical shifts δ and scalar ${}^{1}J_{CH}$ couplings for the enantiomers of camphor. *s* = *syn* to the methylene bridge; *a* = *anti.*

		δ (^{13}C) [ppm]	δ (^{1}H) [ppm]	$^{1}J_{CH}$ [Hz]
C1	-	219.7	-	-
C2	-	46.9	-	-
C3	H3s	30.1	1.66	134.1
C3	H3a	30.1	1.39	133.4
C4	H4s	27.2	1.93	131.4
C4	H4a	27.2	1.31	133.6
C5	H5	43.2	2.07	142.7
C6	H6s	43.6	2.32	130.0
C6	H6a	43.6	1.82	133.1
C7	-	57.8	-	-
C8	H8	19.9	0.81	124.9
C9	H9	19.3	0.94	125.1
C10	H10	9.3	0.89	126.0

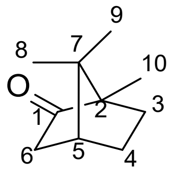

(+)-Campher

Table S7 RDCs of Camphor in the indicated sticks.

Stick-Nr			149 (+)	150 (-)
		$^{1}J_{CH}$ [Hz]	$^{1}D_{CH}$ [Hz]	$^{1}D_{CH}$ [Hz]
C3	H3s	134.1	1.95	1.15
	H3a	133.4	-2.40	-5.00
C4	H4s	131.4	-4.05	-4.00
	H4a	133.6	0.40	-0.60
C5	H5	142.7	-1.25	-0.20
C6	H6s	130	1.80	0.25
	H6a	133.1	0.75	0.90
C8	H8	124.9	-1.00	-0.50
C9	H9	125.1	1.20	1.90
C10	H10	126	-0.90	-0.65

2.5.3 Pinene

Table S8 Numbering of the nuclei, assignment of the chemical shifts δ and scalar ${}^{1}J_{CH}$ couplings for the enantiomers of α-pinene. *s* = *syn* to the methylene bridge; *a* = *anti.*

		δ (^{13}C) [ppm]	δ (^{1}H) [ppm]	$^{1}J_{CH}$ [Hz]
C1	H1	47.2	1.93	143.6
C2	-	144.6	-	-
C3	H3	116.2	5.18	157.1
C4	H4s	31.4	2.18	126.2
C4	H4a	31.4	2.22	127.0
C5	H5	40.9	2.07	142.2
C6	-	38.1	-	-
C7	H7s	31.6	2.33	135.5
C7	H7a	31.6	1.16	140.3
C8	H8	26.5	1.27	124.4
C9	H9	20.9	0.84	123.9
C10	H10	23.1	1.65	125.4

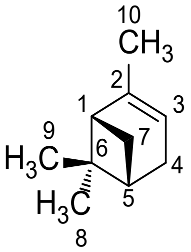

(-)-alpha-Pinen

Table S9 RDCs of Pinene in the indicated sticks.

Stick-Nr		$^{1}J_{CH}$ [Hz]	**153 (+)** $^{1}D_{CH}$ [Hz]	**154 (-)** $^{1}D_{CH}$ [Hz]
C9	H9	123.9	0.55	0.30
C10	H10	125.4	0.15	0.10
C8	H8	124.4	-0.20	-0.40
C4	H4s	126.2	-0.55	0.35
	H4a	127	0.10	-0.05
C7	H7s	135.5	1.40	1.75
	H7a	140.3	-1.40	-1.20
C6	H6	-	-	-
C5	H5	142.2	-0.65	-0.65
C1	H1	143.6	2.00	2.15
C3	H3	157.1	1.30	1.35
C2	H2	-	-	-

2.5.4 Menthol

Table S10 Numbering of the nuclei, assignment of the chemical shifts δ and scalar $^{1}J_{CH}$ couplings for the enantiomers of menthol. *a = axial; e = equatorial.*

		δ (^{13}C) [ppm]	δ (^{1}H) [ppm]	$^{1}J_{CH}$ [Hz]
C1	H1	31.7	1.39	124.7
C2	H2a	45.2	0.95	124.0
C2	H2e	45.2	1.94	127.4
C3	H3	71.6	3.38	138.9
C4	H4	50.2	1.09	122.7
C5	H5a	23.3	0.95	122.0
C5	H5e	23.3	1.58	127.8
C6	H6e	34.6	1.64	126.5
C6	H6a	34.6	0.83	122.9
C7	H7	22.3	0.89	124.7
C8	H8	25.9	2.15	126.0
C9	H9	16.2	0.91	124.2
C10	H10	21.1	0.79	123.6

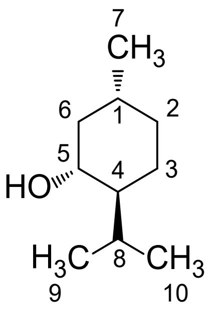

(-)-Menthol

Table S11 RDCs of menthol in the indicated sticks.

Stick-Nr		$^{1}J_{CH}$ [Hz]	155 (+) $^{1}D_{CH}$ [Hz]	156 (-) $^{1}D_{CH}$ [Hz]
C1	H1	124.7	-13.5	-14.9
C2	H2a	124.0	-13.6	-16.3
	H2e	127.4	2.7	4.0
C3	H3	138.9	-12.7	-15.9
C4	H4	122.7	-12.5	-15.4
C5	H5a	122.0	-12.5	
	H5e	127.8	1.4	
C6	H6e	126.5	-3.3	-3.4
	H6a	122.9	-11.5	-14.5
C7	H7	124.7	-3.1	-2.9
C8	H8	126.0	-0.9	-3.4
C10	H10	124.2	0.3	-1.0
C9	H9	123.6	3.6	3.7

2.5.5 Perilla acid (310 K)

Table S12 Numbering of the nuclei, assignment of the chemical shifts δ and scalar ${}^{1}J_{CH}$ couplings for perilla acid.

		δ (^{13}C) [ppm]	δ (^{1}H) [ppm]	$^{1}J_{CH}$ [Hz]
C1	-	31.7	-	-
C2	-	45.2	-	-
C3	H3	142.0	7.14	159.8
C4	H4a	24.3	2.48	-
C4	H4b	24.3	2.21	-
C5	H5	40.1	2.17	127.3
C6	H6a	27.1	1.91	130.1
C6	H6b	27.1	1.48	126.8
C7	H7a	31.4	2.15	-
C7	H7b	31.4	2.37	132.9
C8	-	129.7	-	-
C9	H9	20.9	1.76	126.7
C10	H10t	109.4	4.77	155.2
C10	H10c	109.4	4.73	154.2

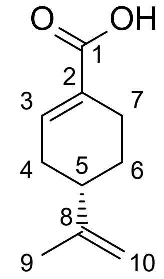

(-)-Perillasäure

Table S13 RDCs of perilla acid in the indicated sticks.

	Stick-Nr	$^{1}J_{CH}$ [Hz]	**188 (1)** $^{1}D_{CH}$ [Hz]	**191 (*ent*-1)** $^{1}D_{CH}$ [Hz]
C9	H9	125.7	0.50	0.20
C4	H4A			
	H4B			
C6	H6A	130.1	-4.20	-7.65
	H6B	126.8	1.50	-5.40
C7	H7B	132.9	-19.60	-4.55
	H7A			
C5	H5	127.3	-0.80	-4.10
C10	H10T	155.2	18.30	17.95
	H10C	154.2	-1.75	-1.40
C8	-			
C3	H3	159.8	-5.10	-4.50
C2	-			
C1	-			

2.5.6 Strychnine

Table S14 Numbering of the nuclei, assignment of the chemical shifts δ and scalar $^{1}J_{CH}$ couplings for strychnine.

		δ (^{13}C) [ppm]	δ (^{1}H) [ppm]	$^{1}J_{CH}$ [Hz]
C1	H1	122.3	7.17	159
C2	H2	124.3	7.1	161.6
C3	H3	128.7	7.26	159.6
C4	H4	116.3	8.09	168
C5	-	142.2	-	-
C6	-	132.8	-	-
C7	-	51.9	-	-
C8	H8	60	3.87	144.9
C10	-	169.3	-	-
C11	H11a	42.5	3.13	135.5
C11	H11b	42.5	2.67	126.3
C12	H12	77.6	4.29	149.2
C13	H13	46.2	1.28	125.7
C14	H14	31.6	3.16	132.4
C15	H15a	26.7	2.37	131.2
C15	H15b	26.7	1.48	130.3
C16	H16	60.3	3.99	146.6
C17	H17a	42.7	1.91	132.9
C17	H17b	42.7	1.91	132.9
C18	H18a	50.4	3.25	146.2
C18	H18b	50.4	2.89	131.2
C20	H20a	52.7	3.74	139.3
C20	H20b	52.7	2.77	139.3
C21	-	140.6	-	-
C22	H22	127.9	5.93	157.7
C23	H23a	64.6	4.16	145.3
C23	H23b	64.6	4.07	137.2

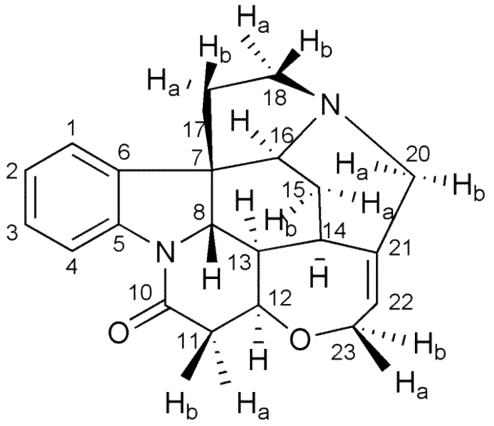

Table S15 RDCs of strychnine in the indicated sticks.

Stick-Nr		$^{1}J_{CH}$ [Hz]	**173** (*ent*-**1**) $^{1}D_{CH}$ [Hz]	**185 (1)** $^{1}D_{CH}$ [Hz]
C3	H3	158.5	5.70	7.25
C22	H22	158.6	-0.85	-3.20
C2	H2	159.7	16.80	19.50
C1	H1	158.3	-20.40	-19.80
C4	H4	168.3	-20.55	-22.50
C12	H12	148.9	2.30	2.60
C23	H23a	145.3	13.55	14.70
	H23b	137	3.70	-1.05
C16	H16	146.5	8.05	7.15
C8	H8	144.8	6.40	3.20
C20	H20a	138.3	-4.65	-5.70
	H20b	138.1	3.95	4.10
C18	H18a	146.7		
	H18b	130.8		
C13	H13	124.8	2.40	-0.15
C17	H17a	133	1.15	0.35
	H17b	133	1.15	0.35
C11	H11a	135.2	-13.85	-16.05
	H11b	125.6	3.00	0.65
C14	H14	131	4.55	6.50
C15	H15a	130.9	-11.05	-12.15
	H15b	129.7	2.25	-4.40

2.5.7 Sparteine

Table S16 Numbering of the nuclei, assignment of the chemical shifts δ and scalar $^{1}J_{CH}$ couplings for sparteine. *a = axial; e = equatorial.*

		δ (^{13}C) [ppm]	δ (^{1}H) [ppm]	$^{1}J_{CH}$ [Hz]
C2	H2e	56.4	2.67	136.3
C2	H2a		1.94	126.1
C3	H3a	26.0	1.51	
C3	H3e		1.51	
C4	H4e	24.8	1.67	
C4	H4a	24.8	1.67	
C5	H5a	29.5	1.35	124.3
C5	H5e		1.21	126.0
C6	H6	66.7	1.70	127.0
C7	H7	33.2	1.81	130.8
C8	H8e	27.8	2.04	132.5
C8	H8a		1.03	127.9
C9	H9	36.2	1.44	129.8
C10	H10e	62.1	2.51	136.2
C10	H10a		1.97	127.5
C11	H11	64.6	1.94	130.4
C12	H12a	34.8	1.49	126.3
C12	H12e		1.29	127.1
C13	H13e	25.0	1.18	
C13	H13a		1.18	
C14	H14a	26.1	1.55	
C14	H14e		1.55	
C15	H15e	55.6	2.77	135.6
C15	H15a		1.97	127.3
C17	H17e	53.7	2.66	135.5
C17	H17a		2.33	131.6

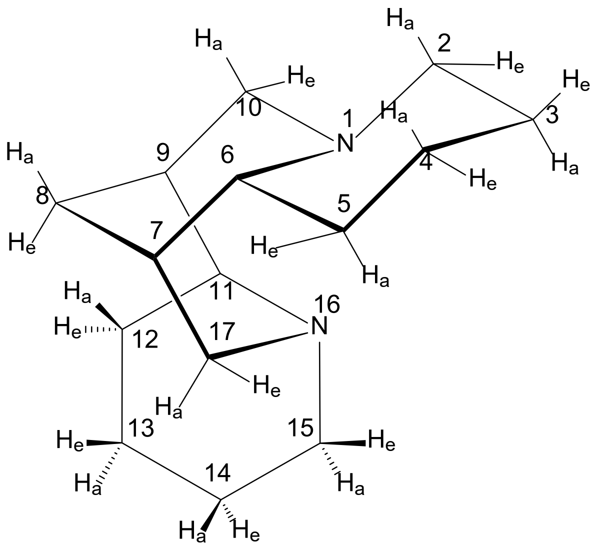

Table S17 RDCs of sparteine in the indicated sticks.

Stick-Nr		$^{1}J_{CH}$ [Hz]	**176** (*ent*-**1**) $^{1}D_{CH}$ [Hz]	**180 (1)** $^{1}D_{CH}$ [Hz]
C6	H6	127.0	0.40	3.40
C11	H11	130.4		
C10	H10e	136.2	1.75	1.90
	H10a	127.5	3.00	4.15
C2	H2e	136.3	2.75	1.10
	H2a	126.1	1.40	2.75
C15	H15e	135.6	5.45	6.05
	H15a	127.3		3.70
C17	H17e	135.5	5.10	
	H17a	131.6	-8.35	-9.35
C9	H9	129.8	8.45	8.15
C12	H12a	126.3		
	H12e	127.1		
C7	H7	130.8	3.15	3.60
C5	H5a	124.3	2.85	3.30
	H5e	126.0	2.65	2.30
C8	H8e	132.5	-10.50	-12.60
	H8a	127.9	1.05	4.85

2.5.8 Cholesterol (310 K)

Table S18 Numbering of the nuclei, assignment of the chemical shifts δ and scalar $^{1}J_{CH}$ couplings for cholesterol. *a* = *alpha-*, *b* = *beta*-side of the steroid structure.

		δ (^{13}C) [ppm]	δ (^{1}H) [ppm]	$^{1}J_{CH}$ [Hz]
C1	H1a	32.07	1.5	125.2
C1	H1b	32.07	1.96	126.9
C2	H2	121.83	5.339	152.6
C3		140.93		
C4	H4a	42.47	2.283	129.4
C4	H4b	42.47	2.232	124.4
C5	H5	71.92	3.508	142.4
C6	H6a	31.83	1.821	129.1
C6	H6b	31.83	1.489	125.4
C7	H7a	37.43	1.065	124.3
C7	H7b	37.43	1.835	128.7
C8		36.66		
C9	H9	19.54	0.995	125.4
C10	H10	50.32	0.918	122.3
C11	H11	32.07	1.449	130
C12	H12a	21.25	1.494	124.2
C12	H12b	21.25	1.449	122.4
C13	H13a	39.96	1.143	123.4
C13	H13b	39.96	1.998	126.8
C14		42.47		
C15	H15	12.01	0.678	124.1
C16	H16	56.95	0.974	122
C17	H17a	24.45	1.561	130.9
C17	H17b	24.45	1.056	126.5
C18	H18a	28.38	1.818	131.5
C18	H18b	28.38	1.249	124.9
C19	H19	56.35	1.075	122.9
C20	H20	35.94	1.362	124

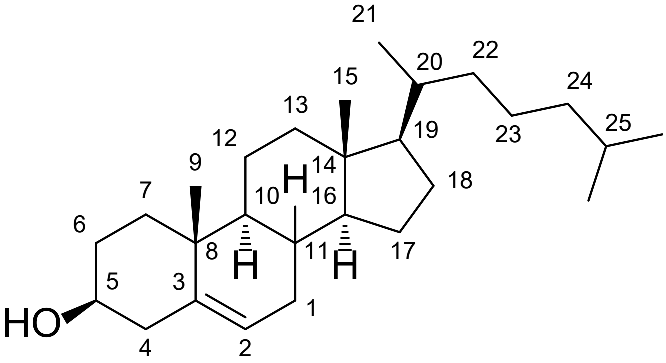

C21	H21	18.88	0.914	124
C22	H221	36.36	0.98	119.6
C22	H222	36.36	1.325	125.4
C23	H231	24.1		
C23	H232	24.1		
C24	H241	39.68		
C24	H242	39.68	1.11	123.2
C25	H25	28.15	1.511	124.9
C26	H26	22.71	0.855	124
C27	H27	22.95	0.869	124

Figure S5 RDCs of Cholesterol in Stick-**S187** (*ent*-**1**) und Stick-190 (**1**).

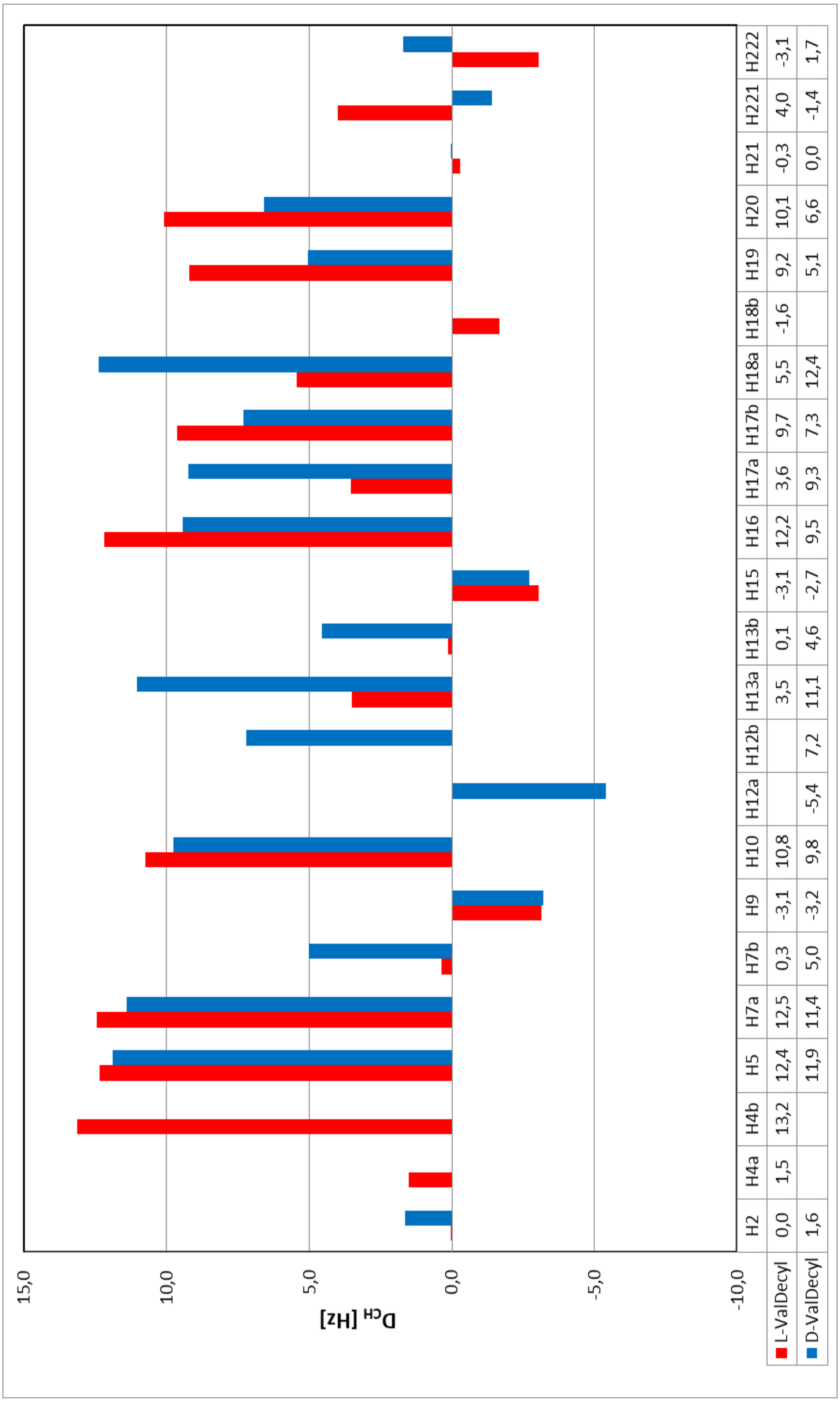

3 Calculation of orientational properties

The orientational properties were calculated using the program *ConArch*$^+$.[10] For that purpose the coordinates of the analytes (DFT; B3LYP/6-311+G(d,p)[11] as well as the measured RDCs were provided as input. The latter were calculated using ${}^{1}T_{CH} = {}^{1}J_{CH} + 2{}^{1}D_{CH}$ with ${}^{1}T_{CH}$ extracted from the CLIP-HSQC-spectra as described above. All uncertainties were calculated using a Monte-Carlo simulation with 10000 steps as described in the literature.[12]

The cosine of the generalized angle between two alignment tensors has been calculated as follows.[13]

$$GCB = cos\beta = \frac{\langle A^{(1)} | A^{(2)} \rangle}{|A^{(1)}||A^{(2)}|}$$

$$\text{with } \langle A^{(1)} | A^{(2)} \rangle = \sum_{i,j} A_{i,j}^{(1)} A_{i,j}^{(2)} \text{ und } |A| = \sqrt{\langle A | A \rangle} = \sqrt{\sum_{i,j} A_{i,j}^{2}}$$

3.1 Experimental and calculated RDCs –Tensor properties

3.1.1 (-)-IPC in Stick S16

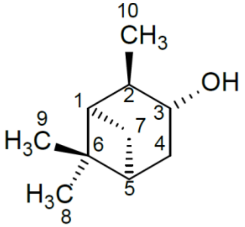

(-)-IPC

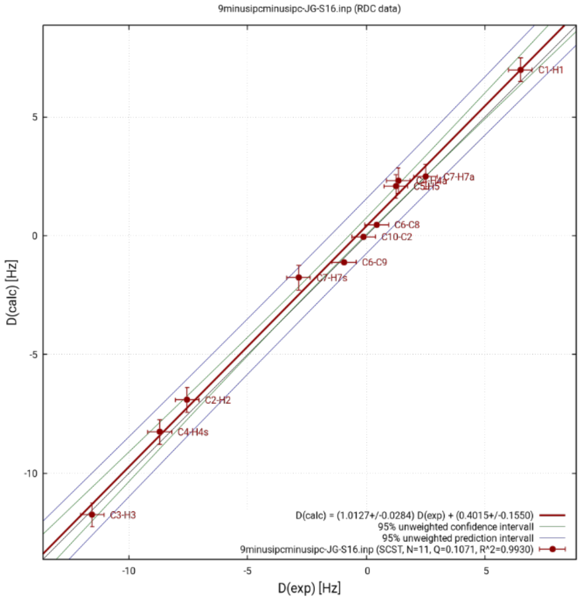


```
================================================================================
info : Start Analysis for Structure 'CalculateOnlyRDCs= 1' in file '9minusipcminusipc-JG-S16.inp'
================================================================================

--------------------------------------------------------------------------------
info : Start Single-Conformer Single-Tensor (SCST) Fit with 11 RDCs
info : File: '9minusipcminusipc-JG-S16.inp', Title: 'CalculateOnlyRDCs= 1'
--------------------------------------------------------------------------------

--------------------------------------------------------------------------------
SVD Best-Fit Saupe Vector S(zz), S(xx-yy), S(xy), S(xz), S(yz):
  7.626961e-05 -2.165732e-04 -1.876400e-04  4.121228e-04 -1.824618e-04
Saupe Tensor (S):                                    Alignment Tensor (A):
 -1.464214e-04 -1.876400e-04  4.121228e-04            -9.761427e-05 -1.250933e-04  2.747485e-04
 -1.876400e-04  7.015179e-05 -1.824618e-04            -1.250933e-04  4.676786e-05 -1.216412e-04
  4.121228e-04 -1.824618e-04  7.626961e-05             2.747485e-04 -1.216412e-04  5.084640e-05
Trace of Saupe Tensor:        1.355253e-20           Trace of Alignment Tensor:    9.035018e-21
Eigenvectors of Saupe Tensor (S):                    Eigenvectors of Alignment Tensor (A):
  2.285519e-01 -8.062365e-01  5.456618e-01             2.285519e-01 -8.062365e-01  5.456618e-01
  8.695704e-01 -8.295504e-02 -4.867913e-01             8.695704e-01 -8.295504e-02 -4.867913e-01
  4.377343e-01  5.857484e-01  6.821198e-01             4.377343e-01  5.857484e-01  6.821198e-01
Eigenvalues of Saupe Tensor S(xx), S(yy), S(zz):     Eigenvalues of Alignment Tensor A(xx), A(yy), A(zz):
 -7.101593e-05 -4.651442e-04  5.361601e-04            -4.734396e-05 -3.100961e-04  3.574401e-04

Alignment Tensor Irreducible Representation (A0, A1R, A1I, A2R, A2I):
  1.209126e-04  5.334588e-04 -2.361817e-04 -1.401680e-04 -2.428845e-04

Tensor Properties: *)
A(axial)      =   5.361601e-04         # alignment tensor axial component   = 3/2*A(zz) = S(zz)
A(rhombic)    =   2.627522e-04         # alignment tensor rhombic component = A(xx) - A(yy) = 2/3*(S(xx) - S(yy))
A(rhombicity) =   4.900629e-01         # alignment tensor rhombicity        = A(rhombic) / A(axial)
A(asymmetry)  =   7.350943e-01         # alignment tensor asymmetry         = 3/2*A(rhombicity) = (A(xx) - A(yy))/A(zz) = (S(xx) - S(yy))/S(zz)
GDO           =   5.824489e-04         # generalized degree of order        = sqrt(3/2)*|A(xx),A(yy),A(zz)| = sqrt(2/3)*|S(xx),S(yy),S(zz)|
----------
*) F. Kramer, M.V. Deshmukh, H. Kessler and S.J. Glaser, Concepts Magn. Res. A, 2004, 21A, 21-40.



--------------------------------------------------------------------------------
```

```
Results for Multi-Parameter SVD Fit of Calculated and Experimental Data:
           D(calc)[HZ]      +/- Error    D(exp)[HZ]      +/- Error  Rel. Weights             D(exp)-D(calc)        Normalized Weights    Atom Labels
D[01] =       6.988075              -      6.500000       0.500000      1.000000    r[01] =      -0.488075    w[01] =       0.090909    C1-H1
D[02] =      -6.910577              -     -7.550000       0.500000      1.000000    r[02] =      -0.639423    w[02] =       0.090909    C2-H2
D[03] =     -11.752321              -    -11.550000       0.500000      1.000000    r[03] =       0.202321    w[03] =       0.090909    C3-H3
D[04] =      -8.259128              -     -8.700000       0.500000      1.000000    r[04] =      -0.440872    w[04] =       0.090909    C4-H4s
D[05] =       2.327651              -      1.350000       0.500000      1.000000    r[05] =      -0.977651    w[05] =       0.090909    C4-H4a
D[06] =       2.082696              -      1.250000       0.500000      1.000000    r[06] =      -0.832696    w[06] =       0.090909    C5-H5
D[07] =      -1.763125              -     -2.850000       0.500000      1.000000    r[07] =      -1.086875    w[07] =       0.090909    C7-H7s
D[08] =       2.488272              -      2.500000       0.500000      1.000000    r[08] =       0.011728    w[08] =       0.090909    C7-H7a
D[09] =       0.466255              -      0.440000       0.500000      1.000000    r[09] =      -0.026255    w[09] =       0.090909    C6-C8
D[10] =      -1.108405              -     -0.930000       0.500000      1.000000    r[10] =       0.178405    w[10] =       0.090909    C6-C9
D[11] =      -0.041176              -     -0.110000       0.500000      1.000000    r[11] =      -0.068824    w[11] =       0.090909    C10-C2


Results for Multi-Parameter Fit of Calculated and Experimental Data:
rank  =             5                # rank of cosine matrix (check input if rank < 5)
cond  =   2.128683e+00               # condition number of cosine matrix (check input and singular values if very large)
aic   =      25.001010               # information criterion (AIC) for 5 degrees of freedom
qfac  =       0.107064               # weighted Q-Factor as defined by Cornilescu
r^2   =       0.987160               # coefficient of determination r^2 = 1 - chi^2 / (weighted sum of squares)

(|D|) =       4.017062      3.975455   # mean absolute (calc./exp.) parameter D[i]
|D|min=       0.041176      0.110000   # min. absolute (calc./exp.) parameter D[i]
|D|max=      11.752321     11.550000   # max. absolute (calc./exp.) parameter D[i]
Drange=     -11.752321      6.988075   # min. and max. (calc.)      parameter D[i]
Drange=     -11.550000      6.500000   # min. and max. (exp.)       parameter D[i]

Results for Linear Regression of Calculated and Experimental Data (N=11):
c(b)  =       0.401532 +/-  0.155027   # linear regression intercept and error
c(m)  =       1.012653 +/-  0.028426   # linear regression slope and error
rmsd  =       0.583894                 # unweighted total root-mean-square deviation
chisq =       0.340932                 # weighted total sum of squared residuals
maerr =       0.450284                 # weighted total mean absolute error (sum of weights = 1.000)
R     =       0.996473                 # weighted Pearson correlation coefficient R
R^2   =       0.992958                 # weighted Pearson correlation coefficient R^2

E(RDC)=       1.875126                 # E(RDC)=1/2*K*(sum of weighted deviations (D(exp)-D(calc))^2), K=1.000
```

3.1.2 (+)-IPC in Stick S137

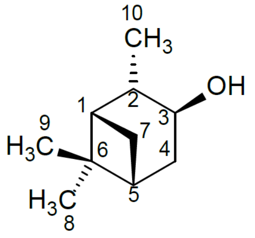

(+)-IPC

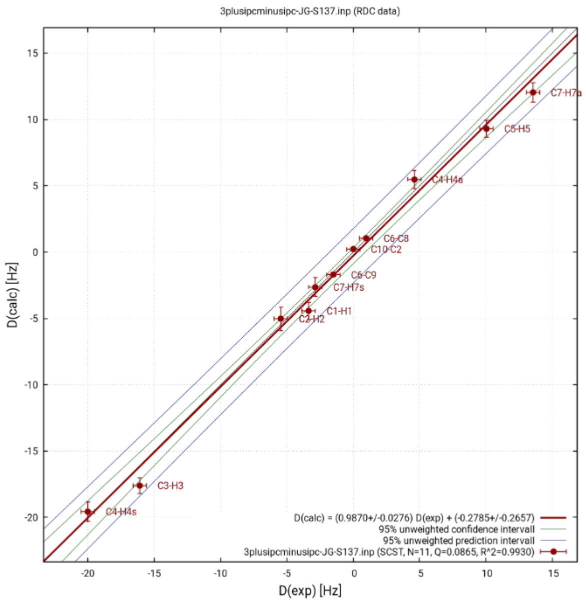


```
--------------------------------------------------------------------------------
info : Start Single-Conformer Single-Tensor (SCST) Fit with 11 RDCs
info : File: '3plusipcminusipc-JG-S137.inp', Title: 'CalculateOnlyRDCs= 1'
--------------------------------------------------------------------------------

--------------------------------------------------------------------------------
SVD Best-Fit Saupe Vector S(zz), S(xx-yy), S(xy), S(xz), S(yz):
  6.822993e-04 -1.448762e-04 -4.322213e-04  3.165271e-04 -4.457116e-04
Saupe Tensor (S):                                      Alignment Tensor (A):
 -4.135877e-04 -4.322213e-04  3.165271e-04               -2.757251e-04 -2.881475e-04  2.110181e-04
 -4.322213e-04 -2.687115e-04 -4.457116e-04               -2.881475e-04 -1.791410e-04 -2.971411e-04
  3.165271e-04 -4.457116e-04  6.822993e-04                2.110181e-04 -2.971411e-04  4.548662e-04
Trace of Saupe Tensor:        0.000000e+00             Trace of Alignment Tensor:    0.000000e+00
Eigenvectors of Saupe Tensor (S):                      Eigenvectors of Alignment Tensor (A):
  5.871957e-01 -7.459504e-01 -3.142598e-01                5.871957e-01 -7.459504e-01 -3.142598e-01
 -6.273060e-01 -6.647317e-01  4.057327e-01               -6.273060e-01 -6.647317e-01  4.057327e-01
 -5.115549e-01 -4.110748e-02 -8.582667e-01               -5.115549e-01 -4.110748e-02 -8.582667e-01
Eigenvalues of Saupe Tensor S(xx), S(yy), S(zz):       Eigenvalues of Alignment Tensor A(xx), A(yy), A(zz):
 -2.275952e-04 -7.813059e-04  1.008901e-03               -1.517301e-04 -5.208706e-04  6.726007e-04

Alignment Tensor Irreducible Representation (A0, A1R, A1I, A2R, A2I):
  1.081670e-03  4.097181e-04 -5.769368e-04 -9.376511e-05 -5.594747e-04

Tensor Properties: *)

A(axial)      =   1.008901e-03         # alignment tensor axial component   = 3/2*A(zz) = S(zz)
A(rhombic)    =   3.691405e-04         # alignment tensor rhombic component = A(xx) - A(yy) = 2/3*(S(xx) - S(yy))
A(rhombicity) =   3.658838e-01         # alignment tensor rhombicity        = A(rhombic) / A(axial)
A(asymmetry)  =   5.488256e-01         # alignment tensor asymmetry         = 3/2*A(rhombicity) = (A(xx) - A(yy))/A(zz) = (S(xx) - S(yy))/S(zz)
GDO           =   1.058338e-03         # generalized degree of order        = sqrt(3/2)*|A(xx),A(yy),A(zz)| = sqrt(2/3)*|S(xx),S(yy),S(zz)|
----------
*) F. Kramer, M.V. Deshmukh, H. Kessler and S.J. Glaser, Concepts Magn. Res. A, 2004, 21A, 21-40.
```

```
--------------------------------------------------------------------------------
Results for Multi-Parameter SVD Fit of Calculated and Experimental Data:
           D(calc)[HZ]     +/- Error    D(exp)[HZ]     +/- Error  Rel. Weights            D(exp)-D(calc)        Normalized Weights    Atom Labels
D[01] =      -4.444081             -     -3.350000      0.500000      1.000000    r[01] =       1.094081    w[01] =       0.090909    C1-H1
D[02] =      -5.027132             -     -5.450000      0.500000      1.000000    r[02] =      -0.422868    w[02] =       0.090909    C2-H2
D[03] =     -17.597730             -    -16.100000      0.500000      1.000000    r[03] =       1.497730    w[03] =       0.090909    C3-H3
D[04] =     -19.559736             -    -20.000000      0.500000      1.000000    r[04] =      -0.440264    w[04] =       0.090909    C4-H4s
D[05] =       5.483015             -      4.600000      0.500000      1.000000    r[05] =      -0.883015    w[05] =       0.090909    C4-H4a
D[06] =       9.301765             -     10.050000      0.500000      1.000000    r[06] =       0.748235    w[06] =       0.090909    C5-H5
D[07] =      -2.636381             -     -2.850000      0.500000      1.000000    r[07] =      -0.213619    w[07] =       0.090909    C7-H7s
D[08] =      12.028318             -     13.550000      0.500000      1.000000    r[08] =       1.521682    w[08] =       0.090909    C7-H7a
D[09] =       1.043096             -      0.950000      0.500000      1.000000    r[09] =      -0.093096    w[09] =       0.090909    C6-C8
D[10] =      -1.710870             -     -1.500000      0.500000      1.000000    r[10] =       0.210870    w[10] =       0.090909    C6-C9
D[11] =       0.207960             -     -0.010000      0.500000      1.000000    r[11] =      -0.217960    w[11] =       0.090909    C10-C2


Results for Multi-Parameter Fit of Calculated and Experimental Data:
rank  =            5                # rank of cosine matrix (check input if rank < 5)
cond  =   2.128683e+00              # condition number of cosine matrix (check input and singular values if very large)
aic   =      40.456880              # information criterion (AIC) for 5 degrees of freedom
qfac  =       0.086520              # weighted Q-Factor as defined by Cornilescu
r^2   =       0.992234              # coefficient of determination r^2 = 1 - chi^2 / (weighted sum of squares)

(|D|) =       7.185462      7.128182   # mean absolute (calc./exp.) parameter D[i]
|D|min=       0.207960      0.010000   # min. absolute (calc./exp.) parameter D[i]
|D|max=      19.559736     20.000000   # max. absolute (calc./exp.) parameter D[i]
Drange=     -19.559736     12.028318   # min. and max. (calc.)      parameter D[i]
Drange=     -20.000000     13.550000   # min. and max. (exp.)       parameter D[i]

Results for Linear Regression of Calculated and Experimental Data (N=11):
c(b)  =      -0.278457 +/-  0.265674   # linear regression intercept and error
c(m)  =       0.987009 +/-  0.027628   # linear regression slope and error
rmsd  =       0.831987                 # unweighted total root-mean-square deviation
chisq =       0.692202                 # weighted total sum of squared residuals
maerr =       0.667584                 # weighted total mean absolute error (sum of weights = 1.000)
R     =       0.996493                 # weighted Pearson correlation coefficient R
R^2   =       0.992998                 # weighted Pearson correlation coefficient R^2

E(RDC)=       3.807110                 # E(RDC)=1/2*K*(sum of weighted deviations (D(exp)-D(calc))^2), K=1.000

--------------------------------------------------------------------------------
```

3.1.3 (-)-IPC in Stick S138

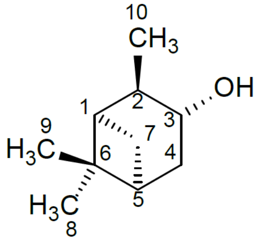

(-)-IPC

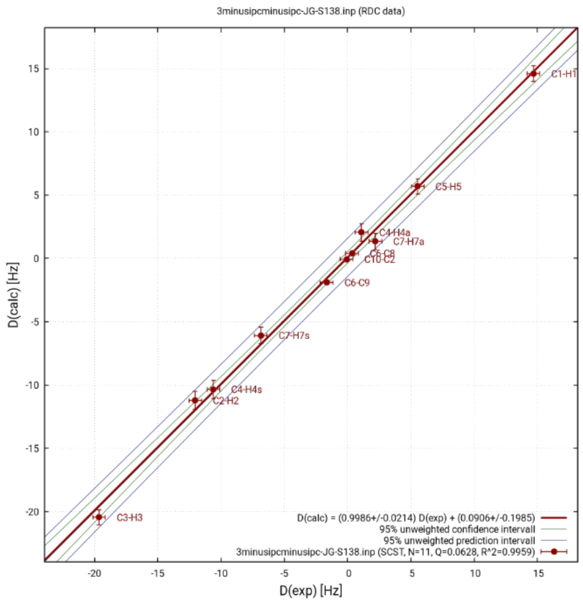


```
===============================================================================
info : Start Analysis for Structure 'CalculateOnlyRDCs= 1' in file '3minusipcminusipc-JG-S138.inp'
===============================================================================

-------------------------------------------------------------------------------
info : Start Single-Conformer Single-Tensor (SCST) Fit with 11 RDCs
info : File: '3minusipcminusipc-JG-S138.inp', Title: 'CalculateOnlyRDCs= 1'
-------------------------------------------------------------------------------

-------------------------------------------------------------------------------
SVD Best-Fit Saupe Vector S(zz), S(xx-yy), S(xy), S(xz), S(yz):
 -6.949705e-05 -3.243619e-04 -2.549556e-04  6.725321e-04 -4.198180e-04
Saupe Tensor (S):                                      Alignment Tensor (A):
 -1.274324e-04 -2.549556e-04  6.725321e-04              -8.495494e-05 -1.699704e-04  4.483548e-04
 -2.549556e-04  1.969295e-04 -4.198180e-04              -1.699704e-04  1.312863e-04 -2.798787e-04
  6.725321e-04 -4.198180e-04 -6.949705e-05               4.483548e-04 -2.798787e-04 -4.633136e-05
Trace of Saupe Tensor:       -2.710505e-20             Trace of Alignment Tensor:    -1.807004e-20
Eigenvectors of Saupe Tensor (S):                      Eigenvectors of Alignment Tensor (A):
  4.859818e-01  6.837648e-01 -5.443228e-01               4.859818e-01  6.837648e-01 -5.443228e-01
  8.148967e-01 -1.294284e-01  5.649706e-01               8.148967e-01 -1.294284e-01  5.649706e-01
  3.158561e-01 -7.181323e-01 -6.200975e-01               3.158561e-01 -7.181323e-01 -6.200975e-01
Eigenvalues of Saupe Tensor S(xx), S(yy), S(zz):       Eigenvalues of Alignment Tensor A(xx), A(yy), A(zz):
 -1.178416e-04 -7.855074e-04  9.033491e-04              -7.856108e-05 -5.236716e-04  6.022327e-04

Alignment Tensor Irreducible Representation (A0, A1R, A1I, A2R, A2I):
 -1.101758e-04  8.705372e-04 -5.434197e-04 -2.099298e-04 -3.300189e-04

Tensor Properties: *)
A(axial)      =   9.033491e-04         # alignment tensor axial component   = 3/2*A(zz) = S(zz)
A(rhombic)    =   4.451105e-04         # alignment tensor rhombic component = A(xx) - A(yy) = 2/3*(S(xx) - S(yy))
A(rhombicity) =   4.927337e-01         # alignment tensor rhombicity        = A(rhombic) / A(axial)
A(asymmetry)  =   7.391006e-01         # alignment tensor asymmetry         = 3/2*A(rhombicity) = (A(xx) - A(yy))/A(zz) = (S(xx) - S(yy))/S(zz)
GDO           =   9.821568e-04         # generalized degree of order        = sqrt(3/2)*|A(xx),A(yy),A(zz)| = sqrt(2/3)*|S(xx),S(yy),S(zz)|
----------
*) F. Kramer, M.V. Deshmukh, H. Kessler and S.J. Glaser, Concepts Magn. Res. A, 2004, 21A, 21-40.
```

```
-------------------------------------------------------------------------------
Results for Multi-Parameter SVD Fit of Calculated and Experimental Data:
           D(calc)[HZ]     +/- Error    D(exp)[HZ]     +/- Error  Rel. Weights            D(exp)-D(calc)        Normalized Weights    Atom Labels
D[01] =      14.585867             -     14.700000      0.500000      1.000000    r[01] =       0.114133    w[01] =       0.090909    C1-H1
D[02] =     -11.219274             -    -12.050000      0.500000      1.000000    r[02] =      -0.830726    w[02] =       0.090909    C2-H2
D[03] =     -20.447631             -    -19.650000      0.500000      1.000000    r[03] =       0.797631    w[03] =       0.090909    C3-H3
D[04] =     -10.335677             -    -10.600000      0.500000      1.000000    r[04] =      -0.264323    w[04] =       0.090909    C4-H4s
D[05] =       2.054301             -      1.100000      0.500000      1.000000    r[05] =      -0.954301    w[05] =       0.090909    C4-H4a
D[06] =       5.689436             -      5.550000      0.500000      1.000000    r[06] =      -0.139436    w[06] =       0.090909    C5-H5
D[07] =      -6.074490             -     -6.850000      0.500000      1.000000    r[07] =      -0.775510    w[07] =       0.090909    C7-H7s
D[08] =       1.353059             -      2.200000      0.500000      1.000000    r[08] =       0.846941    w[08] =       0.090909    C7-H7a
D[09] =       0.410879             -      0.350000      0.500000      1.000000    r[09] =      -0.060879    w[09] =       0.090909    C6-C8
D[10] =      -1.905326             -     -1.650000      0.500000      1.000000    r[10] =       0.255326    w[10] =       0.090909    C6-C9
D[11] =      -0.057996             -     -0.080000      0.500000      1.000000    r[11] =      -0.022004    w[11] =       0.090909    C10-C2


Results for Multi-Parameter Fit of Calculated and Experimental Data:
rank  =              5                # rank of cosine matrix (check input if rank < 5)
cond  =   2.128683e+00                # condition number of cosine matrix (check input and singular values if very large)
aic   =      24.909811                # information criterion (AIC) for 5 degrees of freedom
qfac  =       0.062758                # weighted Q-Factor as defined by Cornilescu
r^2   =       0.995765                # coefficient of determination r^2 = 1 - chi^2 / (weighted sum of squares)

(|D|) =       6.739449      6.798182   # mean absolute (calc./exp.) parameter D[i]
|D|min=       0.057996      0.080000   # min. absolute (calc./exp.) parameter D[i]
|D|max=      20.447631     19.650000   # max. absolute (calc./exp.) parameter D[i]
Drange=     -20.447631     14.585867   # min. and max. (calc.)      parameter D[i]
Drange=     -19.650000     14.700000   # min. and max. (exp.)       parameter D[i]

Results for Linear Regression of Calculated and Experimental Data (N=11):
c(b)  =       0.090597 +/-  0.198520   # linear regression intercept and error
c(m)  =       0.998644 +/-  0.021402   # linear regression slope and error
rmsd  =       0.582116                # unweighted total root-mean-square deviation
chisq =       0.338859                # weighted total sum of squared residuals
maerr =       0.460110                # weighted total mean absolute error (sum of weights = 1.000)
R     =       0.997939                # weighted Pearson correlation coefficient R
R^2   =       0.995883                # weighted Pearson correlation coefficient R^2

E(RDC)=       1.863726                # E(RDC)=1/2*K*(sum of weighted deviations (D(exp)-D(calc))^2), K=1.000

-------------------------------------------------------------------------------
```

3.1.4 (+)-IPC in Stick S142A

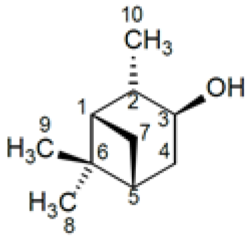

(+)-IPC

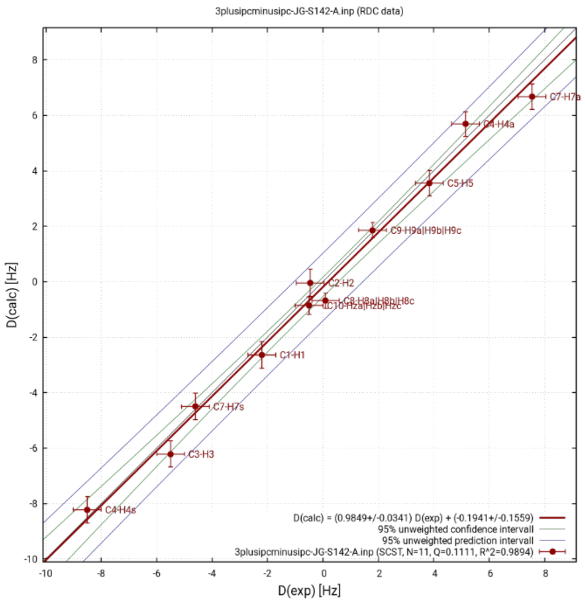


```
===============================================================================
info : Start Analysis for Structure '' in file '3plusipcminusipc-JG-S142-A.inp'
===============================================================================

-------------------------------------------------------------------------------
info : Start Single-Conformer Single-Tensor (SCST) Fit with 11 RDCs
info : File: '3plusipcminusipc-JG-S142-A.inp', Title: ''
-------------------------------------------------------------------------------

-------------------------------------------------------------------------------
SVD Best-Fit Saupe Vector S(zz), S(xx-yy), S(xy), S(xz), S(yz):
  3.262202e-04 -2.519263e-04 -8.440300e-05  9.512892e-05 -2.430767e-04
Saupe Tensor (S):                                      Alignment Tensor (A):
 -2.890732e-04 -8.440300e-05  9.512892e-05              -1.927155e-04 -5.626867e-05  6.341928e-05
 -8.440300e-05 -3.714694e-05 -2.430767e-04              -5.626867e-05 -2.476463e-05 -1.620511e-04
  9.512892e-05 -2.430767e-04  3.262202e-04               6.341928e-05 -1.620511e-04  2.174801e-04
Trace of Saupe Tensor:        5.421011e-20             Trace of Alignment Tensor:    3.614007e-20
Eigenvectors of Saupe Tensor (S):                      Eigenvectors of Alignment Tensor (A):
 -1.986226e-01  9.668385e-01 -1.605377e-01              -1.986226e-01  9.668385e-01 -1.605377e-01
  8.572818e-01  2.507764e-01  4.496433e-01               8.572818e-01  2.507764e-01  4.496433e-01
  4.749916e-01 -4.831671e-02 -8.786629e-01               4.749916e-01 -4.831671e-02 -8.786629e-01
Eigenvalues of Saupe Tensor S(xx), S(yy), S(zz):       Eigenvalues of Alignment Tensor A(xx), A(yy), A(zz):
 -1.522725e-04 -3.157194e-04  4.679919e-04              -1.015150e-04 -2.104796e-04  3.119946e-04

Alignment Tensor Irreducible Representation (A0, A1R, A1I, A2R, A2I):
  5.171669e-04  1.231365e-04 -3.146427e-04 -1.630489e-04 -1.092527e-04

Tensor Properties: *)
A(axial)      =   4.679919e-04        # alignment tensor axial component   = 3/2*A(zz) = S(zz)
A(rhombic)    =   1.089646e-04        # alignment tensor rhombic component = A(xx) - A(yy) = 2/3*(S(xx) - S(yy))
A(rhombicity) =   2.328344e-01        # alignment tensor rhombicity        = A(rhombic) / A(axial)
A(asymmetry)  =   3.492517e-01        # alignment tensor asymmetry         = 3/2*A(rhombicity) = (A(xx) - A(yy))/A(zz) = (S(xx) - S(yy))/S(zz)
GDO           =   4.774112e-04        # generalized degree of order        = sqrt(3/2)*|A(xx),A(yy),A(zz)| = sqrt(2/3)*|S(xx),S(yy),S(zz)|
----------
*) F. Kramer, M.V. Deshmukh, H. Kessler and S.J. Glaser, Concepts Magn. Res. A, 2004, 21A, 21-40.
```

```
--------------------------------------------------------------------------------
Results for Multi-Parameter SVD Fit of Calculated and Experimental Data:
           D(calc)[HZ]     +/- Error    D(exp)[HZ]     +/- Error  Rel. Weights            D(exp)-D(calc)        Normalized Weights    Atom Labels
D[01] =      -2.638497             -     -2.200000      0.500000      1.000000    r[01] =       0.438497    w[01] =       0.090909    C1-H1
D[02] =      -0.042528             -     -0.450000      0.500000      1.000000    r[02] =      -0.407472    w[02] =       0.090909    C2-H2
D[03] =      -6.214485             -     -5.500000      0.500000      1.000000    r[03] =       0.714485    w[03] =       0.090909    C3-H3
D[04] =      -8.221996             -     -8.500000      0.500000      1.000000    r[04] =      -0.278004    w[04] =       0.090909    C4-H4s
D[05] =       5.688429             -      5.150000      0.500000      1.000000    r[05] =      -0.538429    w[05] =       0.090909    C4-H4a
D[06] =       3.558072             -      3.850000      0.500000      1.000000    r[06] =       0.291928    w[06] =       0.090909    C5-H5
D[07] =      -4.503993             -     -4.600000      0.500000      1.000000    r[07] =      -0.096007    w[07] =       0.090909    C7-H7s
D[08] =       6.674975             -      7.550000      0.500000      1.000000    r[08] =       0.875025    w[08] =       0.090909    C7-H7a
D[09] =      -0.684155             -      0.100000      0.500000      1.000000    r[09] =       0.784155    w[09] =       0.090909    C8-H8a|H8b|H8c
              4.949058             -             -             -             -                         -                           -    | [3av] C8-H8a
              0.529518             -             -             -             -                         -                           -    | [3av] C8-H8b
             -7.531041             -             -             -             -                         -                           -    | [3av] C8-H8c
D[10] =       1.858539             -      1.800000      0.500000      1.000000    r[10] =      -0.058539    w[10] =       0.090909    C9-H9a|H9b|H9c
              0.803711             -             -             -             -                         -                           -    | [3av] C9-H9a
              5.011315             -             -             -             -                         -                           -    | [3av] C9-H9b
             -0.239409             -             -             -             -                         -                           -    | [3av] C9-H9c
D[11] =      -0.860114             -     -0.500000      0.500000      1.000000    r[11] =       0.360114    w[11] =       0.090909    C10-Hza|Hzb|Hzc
             -5.246300             -             -             -             -                         -                           -    | [3av] C10-Hza
              0.341385             -             -             -             -                         -                           -    | [3av] C10-Hzb
              2.324573             -             -             -             -                         -                           -    | [3av] C10-Hzc

Results for Multi-Parameter Fit of Calculated and Experimental Data:
rank  =              5                # rank of cosine matrix (check input if rank < 5)
cond  =   2.147425e+00                # condition number of cosine matrix (check input and singular values if very large)
aic   =      21.376440                # information criterion (AIC) for 5 degrees of freedom
qfac  =       0.111068                # weighted Q-Factor as defined by Cornilescu
r^2   =       0.987611                # coefficient of determination r^2 = 1 - chi^2 / (weighted sum of squares)

(|D|) =       3.722344      3.654545   # mean absolute (calc./exp.) parameter D[i]
|D|min=       0.042528      0.100000   # min. absolute (calc./exp.) parameter D[i]
|D|max=       8.221996      8.500000   # max. absolute (calc./exp.) parameter D[i]
Drange=      -8.221996      6.674975   # min. and max. (calc.)      parameter D[i]
Drange=      -8.500000      7.550000   # min. and max. (exp.)       parameter D[i]

Results for Linear Regression of Calculated and Experimental Data (N=11):
c(b)  =      -0.194149 +/-  0.155911   # linear regression intercept and error
c(m)  =       0.984885 +/-  0.034056   # linear regression slope and error
rmsd  =       0.508483                # unweighted total root-mean-square deviation
chisq =       0.258555                # weighted total sum of squared residuals
maerr =       0.440241                # weighted total mean absolute error (sum of weights = 1.000)
R     =       0.994663                # weighted Pearson correlation coefficient R
R^2   =       0.989354                # weighted Pearson correlation coefficient R^2

E(RDC)=       1.422055                # E(RDC)=1/2*K*(sum of weighted deviations (D(exp)-D(calc))^2), K=1.000

--------------------------------------------------------------------------------
```

3.1.5 (-)-IPC in Stick S142B

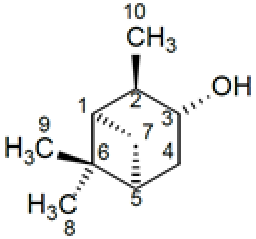

(-)-IPC

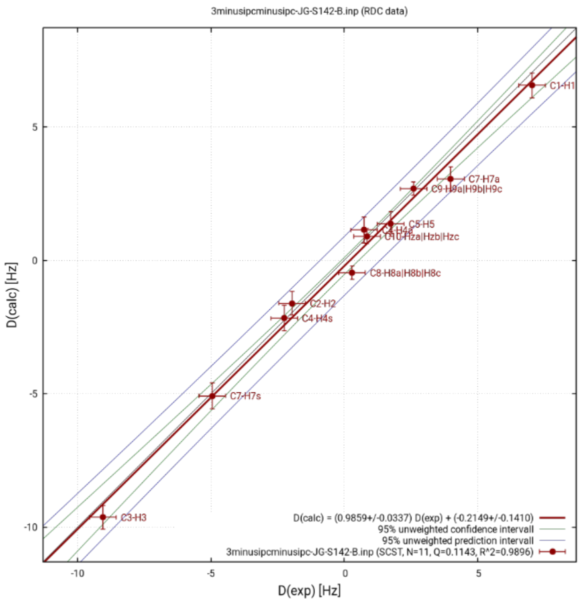


```
================================================================================
info : Start Analysis for Structure '' in file '3minusipcminusipc-JG-S142-B.inp'
================================================================================

--------------------------------------------------------------------------------
info : Start Single-Conformer Single-Tensor (SCST) Fit with 11 RDCs
info : File: '3minusipcminusipc-JG-S142-B.inp', Title: ''
--------------------------------------------------------------------------------

--------------------------------------------------------------------------------
SVD Best-Fit Saupe Vector S(zz), S(xx-yy), S(xy), S(xz), S(yz):
 -9.757161e-05 -3.400584e-04 -1.719893e-04  2.205922e-04 -2.211409e-04
Saupe Tensor (S):                                      Alignment Tensor (A):
 -1.212434e-04 -1.719893e-04  2.205922e-04              -8.082891e-05 -1.146596e-04  1.470614e-04
 -1.719893e-04  2.188150e-04 -2.211409e-04              -1.146596e-04  1.458767e-04 -1.474273e-04
  2.205922e-04 -2.211409e-04 -9.757161e-05               1.470614e-04 -1.474273e-04 -6.504774e-05
Trace of Saupe Tensor:         0.000000e+00            Trace of Alignment Tensor:     0.000000e+00
Eigenvectors of Saupe Tensor (S):                      Eigenvectors of Alignment Tensor (A):
  5.887911e-01  6.918887e-01 -4.178696e-01               5.887911e-01  6.918887e-01 -4.178696e-01
  6.323788e-01 -7.233334e-02  7.712749e-01               6.323788e-01 -7.233334e-02  7.712749e-01
  5.034105e-01 -7.183717e-01 -4.801250e-01               5.034105e-01 -7.183717e-01 -4.801250e-01
Eigenvalues of Saupe Tensor S(xx), S(yy), S(zz):       Eigenvalues of Alignment Tensor A(xx), A(yy), A(zz):
 -1.173608e-04 -3.322984e-04  4.496592e-04              -7.824055e-05 -2.215323e-04  2.997728e-04

Alignment Tensor Irreducible Representation (A0, A1R, A1I, A2R, A2I):
 -1.546833e-04  2.855383e-04 -2.862486e-04 -2.200887e-04 -2.226260e-04

Tensor Properties: *)
A(axial)      =   4.496592e-04         # alignment tensor axial component   = 3/2*A(zz) = S(zz)
A(rhombic)    =   1.432917e-04         # alignment tensor rhombic component = A(xx) - A(yy) = 2/3*(S(xx) - S(yy))
A(rhombicity) =   3.186674e-01         # alignment tensor rhombicity        = A(rhombic) / A(axial)
A(asymmetry)  =   4.780010e-01         # alignment tensor asymmetry         = 3/2*A(rhombicity) = (A(xx) - A(yy))/A(zz) = (S(xx) - S(yy))/S(zz)
GDO           =   4.664685e-04         # generalized degree of order        = sqrt(3/2)*|A(xx),A(yy),A(zz)| = sqrt(2/3)*|S(xx),S(yy),S(zz)|
----------
*) F. Kramer, M.V. Deshmukh, H. Kessler and S.J. Glaser, Concepts Magn. Res. A, 2004, 21A, 21-40.
```

```
--------------------------------------------------------------------------------
Results for Multi-Parameter SVD Fit of Calculated and Experimental Data:
           D(calc)[HZ]     +/- Error    D(exp)[HZ]     +/- Error  Rel. Weights            D(exp)-D(calc)       Normalized Weights    Atom Labels
D[01] =       6.559914             -      7.050000      0.500000      1.000000    r[01] =       0.490086    w[01] =       0.090909    C1-H1
D[02] =      -1.610129             -     -1.950000      0.500000      1.000000    r[02] =      -0.339871    w[02] =       0.090909    C2-H2
D[03] =      -9.634358             -     -9.050000      0.500000      1.000000    r[03] =       0.584358    w[03] =       0.090909    C3-H3
D[04] =      -2.170490             -     -2.250000      0.500000      1.000000    r[04] =      -0.079510    w[04] =       0.090909    C4-H4s
D[05] =       1.139820             -      0.750000      0.500000      1.000000    r[05] =      -0.389820    w[05] =       0.090909    C4-H4a
D[06] =       1.365852             -      1.750000      0.500000      1.000000    r[06] =       0.384148    w[06] =       0.090909    C5-H5
D[07] =      -5.077224             -     -4.950000      0.500000      1.000000    r[07] =       0.127224    w[07] =       0.090909    C7-H7s
D[08] =       3.053800             -      4.000000      0.500000      1.000000    r[08] =       0.946200    w[08] =       0.090909    C7-H7a
D[09] =      -0.464880             -      0.300000      0.500000      1.000000    r[09] =       0.764880    w[09] =       0.090909    C8-H8a|H8b|H8c
              5.480427             -             -             -             -                         -                         -    | [3av] C8-H8a
              3.124911             -             -             -             -                         -                         -    | [3av] C8-H8b
             -9.999978             -             -             -             -                         -                         -    | [3av] C8-H8c
D[10] =       2.684427             -      2.600000      0.500000      1.000000    r[10] =      -0.084427    w[10] =       0.090909    C9-H9a|H9b|H9c
              3.146488             -             -             -             -                         -                         -    | [3av] C9-H9a
              5.336775             -             -             -             -                         -                         -    | [3av] C9-H9b
             -0.429981             -             -             -             -                         -                         -    | [3av] C9-H9c
D[11] =       0.901605             -      0.850000      0.500000      1.000000    r[11] =      -0.051605    w[11] =       0.090909    C10-Hza|Hzb|Hzc
              5.002967             -             -             -             -                         -                         -    | [3av] C10-Hza
             -2.188621             -             -             -             -                         -                         -    | [3av] C10-Hzb
             -0.109529             -             -             -             -                         -                         -    | [3av] C10-Hzc

Results for Multi-Parameter Fit of Calculated and Experimental Data:
rank  =              5                # rank of cosine matrix (check input if rank < 5)
cond  =   2.147425e+00                # condition number of cosine matrix (check input and singular values if very large)
aic   =      20.037342                # information criterion (AIC) for 5 degrees of freedom
qfac  =       0.114271                # weighted Q-Factor as defined by Cornilescu
r^2   =       0.986937                # coefficient of determination r^2 = 1 - chi^2 / (weighted sum of squares)

(|D|) =       3.151136      3.227273   # mean absolute (calc./exp.) parameter D[i]
|D|min=       0.464880      0.300000   # min. absolute (calc./exp.) parameter D[i]
|D|max=       9.634358      9.050000   # max. absolute (calc./exp.) parameter D[i]
Drange=      -9.634358      6.559914   # min. and max. (calc.)      parameter D[i]
Drange=      -9.050000      7.050000   # min. and max. (exp.)       parameter D[i]

Results for Linear Regression of Calculated and Experimental Data (N=11):
c(b)  =      -0.214938 +/-  0.141040   # linear regression intercept and error
c(m)  =       0.985935 +/-  0.033744   # linear regression slope and error
rmsd  =       0.477621                # unweighted total root-mean-square deviation
chisq =       0.228121                # weighted total sum of squared residuals
maerr =       0.385648                # weighted total mean absolute error (sum of weights = 1.000)
R     =       0.994770                # weighted Pearson correlation coefficient R
R^2   =       0.989568                # weighted Pearson correlation coefficient R^2

E(RDC)=       1.254668                # E(RDC)=1/2*K*(sum of weighted deviations (D(exp)-D(calc))^2), K=1.000

--------------------------------------------------------------------------------
```

3.1.6 (+)-IPC in Stick S143

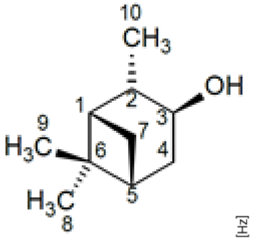

(+)-IPC

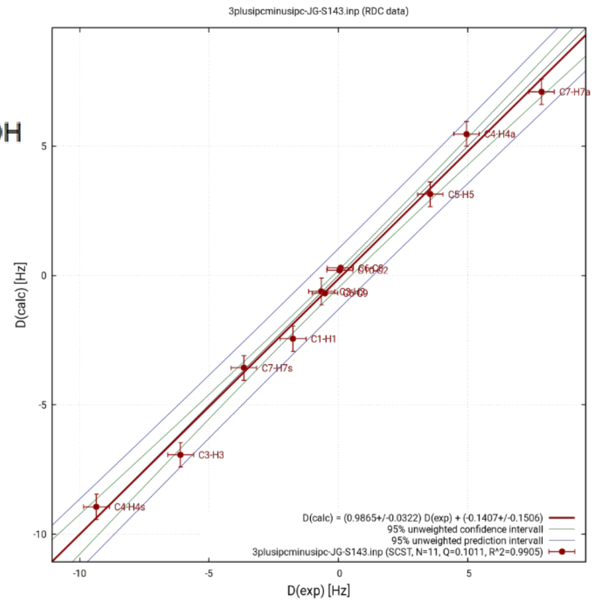


```
================================================================================
info : Start Analysis for Structure 'CalculateOnlyRDCs= 1' in file '3plusipcminusipc-JG-S143.inp'
================================================================================

--------------------------------------------------------------------------------
info : Start Single-Conformer Single-Tensor (SCST) Fit with 11 RDCs
info : File: '3plusipcminusipc-JG-S143.inp', Title: 'CalculateOnlyRDCs= 1'
--------------------------------------------------------------------------------

--------------------------------------------------------------------------------
SVD Best-Fit Saupe Vector S(zz), S(xx-yy), S(xy), S(xz), S(yz):
  3.440815e-04 -2.525691e-04 -1.265048e-04  1.232757e-04 -2.211058e-04
Saupe Tensor (S):                                        Alignment Tensor (A):
 -2.983253e-04 -1.265048e-04  1.232757e-04                -1.988835e-04 -8.433653e-05  8.218382e-05
 -1.265048e-04 -4.575624e-05 -2.211058e-04                -8.433653e-05 -3.050416e-05 -1.474039e-04
  1.232757e-04 -2.211058e-04  3.440815e-04                 8.218382e-05 -1.474039e-04  2.293877e-04
Trace of Saupe Tensor:          0.000000e+00             Trace of Alignment Tensor:     0.000000e+00
Eigenvectors of Saupe Tensor (S):                        Eigenvectors of Alignment Tensor (A):
 -2.816857e-01  9.365569e-01 -2.086007e-01                -2.816857e-01  9.365569e-01 -2.086007e-01
  8.379476e-01  3.460266e-01  4.220300e-01                 8.379476e-01  3.460266e-01  4.220300e-01
  4.674366e-01 -5.591658e-02 -8.822564e-01                 4.674366e-01 -5.591658e-02 -8.822564e-01
Eigenvalues of Saupe Tensor S(xx), S(yy), S(zz):         Eigenvalues of Alignment Tensor A(xx), A(yy), A(zz):
 -1.265708e-04 -3.524247e-04  4.789955e-04                -8.438051e-05 -2.349498e-04  3.193303e-04

Alignment Tensor Irreducible Representation (A0, A1R, A1I, A2R, A2I):
  5.454831e-04  1.595702e-04 -2.862032e-04 -1.634649e-04 -1.637500e-04

Tensor Properties: *)
A(axial)      =   4.789955e-04         # alignment tensor axial component   = 3/2*A(zz) = S(zz)
A(rhombic)    =   1.505693e-04         # alignment tensor rhombic component = A(xx) - A(yy) = 2/3*(S(xx) - S(yy))
A(rhombicity) =   3.143439e-01         # alignment tensor rhombicity        = A(rhombic) / A(axial)
A(asymmetry)  =   4.715159e-01         # alignment tensor asymmetry         = 3/2*A(rhombicity) = (A(xx) - A(yy))/A(zz) = (S(xx) - S(yy))/S(zz)
GDO           =   4.964272e-04         # generalized degree of order        = sqrt(3/2)*|A(xx),A(yy),A(zz)| = sqrt(2/3)*|S(xx),S(yy),S(zz)|
----------
*) F. Kramer, M.V. Deshmukh, H. Kessler and S.J. Glaser, Concepts Magn. Res. A, 2004, 21A, 21-40.
```

```
--------------------------------------------------------------------------------
Results for Multi-Parameter SVD Fit of Calculated and Experimental Data:
           D(calc)[HZ]     +/- Error    D(exp)[HZ]     +/- Error  Rel. Weights            D(exp)-D(calc)        Normalized Weights    Atom Labels
D[01] =      -2.448942            -     -1.750000      0.500000      1.000000    r[01] =       0.698942    w[01] =       0.090909    C1-H1
D[02] =      -0.622681            -     -0.650000      0.500000      1.000000    r[02] =      -0.027319    w[02] =       0.090909    C2-H2
D[03] =      -6.934212            -     -6.100000      0.500000      1.000000    r[03] =       0.834212    w[03] =       0.090909    C3-H3
D[04] =      -8.944479            -     -9.350000      0.500000      1.000000    r[04] =      -0.405521    w[04] =       0.090909    C4-H4s
D[05] =       5.471673            -      4.950000      0.500000      1.000000    r[05] =      -0.521673    w[05] =       0.090909    C4-H4a
D[06] =       3.136312            -      3.550000      0.500000      1.000000    r[06] =       0.413688    w[06] =       0.090909    C5-H5
D[07] =      -3.576163            -     -3.650000      0.500000      1.000000    r[07] =      -0.073837    w[07] =       0.090909    C7-H7s
D[08] =       7.096727            -      7.850000      0.500000      1.000000    r[08] =       0.753273    w[08] =       0.090909    C7-H7a
D[09] =       0.289550            -      0.080000      0.500000      1.000000    r[09] =      -0.209550    w[09] =       0.090909    C6-C8
D[10] =      -0.681243            -     -0.520000      0.500000      1.000000    r[10] =       0.161243    w[10] =       0.090909    C6-C9
D[11] =       0.200701            -      0.050000      0.500000      1.000000    r[11] =      -0.150701    w[11] =       0.090909    C10-C2

Results for Multi-Parameter Fit of Calculated and Experimental Data:
rank  =             5                # rank of cosine matrix (check input if rank < 5)
cond  =   2.128683e+00               # condition number of cosine matrix (check input and singular values if very large)
aic   =      19.833590               # information criterion (AIC) for 5 degrees of freedom
qfac  =       0.101115               # weighted Q-Factor as defined by Cornilescu
r^2   =       0.989656               # coefficient of determination r^2 = 1 - chi^2 / (weighted sum of squares)

(|D|) =       3.582062      3.500000   # mean absolute (calc./exp.) parameter D[i]
|D|min=       0.200701      0.050000   # min. absolute (calc./exp.) parameter D[i]
|D|max=       8.944479      9.350000   # max. absolute (calc./exp.) parameter D[i]
Drange=      -8.944479      7.096727   # min. and max. (calc.)      parameter D[i]
Drange=      -9.350000      7.850000   # min. and max. (exp.)       parameter D[i]

Results for Linear Regression of Calculated and Experimental Data (N=11):
c(b)  =      -0.140669 +/-  0.150560   # linear regression intercept and error
c(m)  =       0.986535 +/-  0.032203   # linear regression slope and error
rmsd  =       0.472748                 # unweighted total root-mean-square deviation
chisq =       0.223491                 # weighted total sum of squared residuals
maerr =       0.386360                 # weighted total mean absolute error (sum of weights = 1.000)
R     =       0.995239                 # weighted Pearson correlation coefficient R
R^2   =       0.990501                 # weighted Pearson correlation coefficient R^2

E(RDC)=       1.229199                 # E(RDC)=1/2*K*(sum of weighted deviations (D(exp)-D(calc))^2), K=1.000

--------------------------------------------------------------------------------
```

3.1.7 (-)-IPC in Stick S144

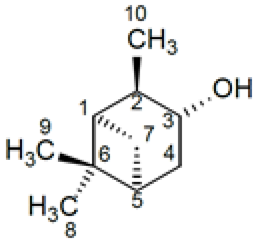

(-)-IPC

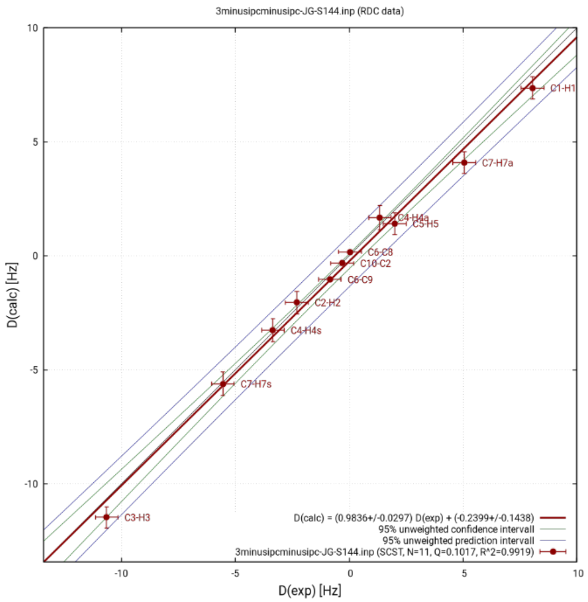


```
===============================================================================
info : Start Analysis for Structure 'CalculateOnlyRDCs= 1' in file '3minusipcminusipc-JG-S144.inp'
===============================================================================

-------------------------------------------------------------------------------
info : Start Single-Conformer Single-Tensor (SCST) Fit with 11 RDCs
info : File: '3minusipcminusipc-JG-S144.inp', Title: 'CalculateOnlyRDCs= 1'
-------------------------------------------------------------------------------

-------------------------------------------------------------------------------
SVD Best-Fit Saupe Vector S(zz), S(xx-yy), S(xy), S(xz), S(yz):
 -8.186400e-05 -4.092361e-04 -2.142486e-04  2.685874e-04 -2.505831e-04
Saupe Tensor (S):                                      Alignment Tensor (A):
 -1.636861e-04 -2.142486e-04  2.685874e-04              -1.091240e-04 -1.428324e-04  1.790583e-04
 -2.142486e-04  2.455501e-04 -2.505831e-04              -1.428324e-04  1.637000e-04 -1.670554e-04
  2.685874e-04 -2.505831e-04 -8.186400e-05               1.790583e-04 -1.670554e-04 -5.457600e-05
Trace of Saupe Tensor:        0.000000e+00             Trace of Alignment Tensor:    0.000000e+00
Eigenvectors of Saupe Tensor (S):                      Eigenvectors of Alignment Tensor (A):
  4.946377e-01  7.577579e-01 -4.256013e-01               4.946377e-01  7.577579e-01 -4.256013e-01
  6.538269e-01 -1.820582e-03  7.566420e-01               6.538269e-01 -1.820582e-03  7.566420e-01
  5.725766e-01 -6.525332e-01 -4.963430e-01               5.725766e-01 -6.525332e-01 -4.963430e-01
Eigenvalues of Saupe Tensor S(xx), S(yy), S(zz):       Eigenvalues of Alignment Tensor A(xx), A(yy), A(zz):
 -1.359782e-04 -3.944618e-04  5.304399e-04              -9.065211e-05 -2.629745e-04  3.536266e-04

Alignment Tensor Irreducible Representation (A0, A1R, A1I, A2R, A2I):
 -1.297815e-04  3.476642e-04 -3.243590e-04 -2.648612e-04 -2.773271e-04

Tensor Properties: *)
A(axial)      =   5.304399e-04         # alignment tensor axial component   = 3/2*A(zz) = S(zz)
A(rhombic)    =   1.723224e-04         # alignment tensor rhombic component = A(xx) - A(yy) = 2/3*(S(xx) - S(yy))
A(rhombicity) =   3.248669e-01         # alignment tensor rhombicity        = A(rhombic) / A(axial)
A(asymmetry)  =   4.873004e-01         # alignment tensor asymmetry         = 3/2*A(rhombicity) = (A(xx) - A(yy))/A(zz) = (S(xx) - S(yy))/S(zz)
GDO           =   5.510334e-04         # generalized degree of order        = sqrt(3/2)*|A(xx),A(yy),A(zz)| = sqrt(2/3)*|S(xx),S(yy),S(zz)|
----------
*) F. Kramer, M.V. Deshmukh, H. Kessler and S.J. Glaser, Concepts Magn. Res. A, 2004, 21A, 21-40.
```

```
--------------------------------------------------------------------------------
Results for Multi-Parameter SVD Fit of Calculated and Experimental Data:
           D(calc)[HZ]     +/- Error    D(exp)[HZ]     +/- Error  Rel. Weights            D(exp)-D(calc)        Normalized Weights    Atom Labels
D[01] =       7.364803             -      8.050000      0.500000      1.000000    r[01] =       0.685197    w[01] =       0.090909    C1-H1
D[02] =      -2.049460             -     -2.300000      0.500000      1.000000    r[02] =      -0.250540    w[02] =       0.090909    C2-H2
D[03] =     -11.474154             -    -10.650000      0.500000      1.000000    r[03] =       0.824154    w[03] =       0.090909    C3-H3
D[04] =      -3.265290             -     -3.350000      0.500000      1.000000    r[04] =      -0.084710    w[04] =       0.090909    C4-H4s
D[05] =       1.681920             -      1.350000      0.500000      1.000000    r[05] =      -0.331920    w[05] =       0.090909    C4-H4a
D[06] =       1.409338             -      2.000000      0.500000      1.000000    r[06] =       0.590662    w[06] =       0.090909    C5-H5
D[07] =      -5.615172             -     -5.550000      0.500000      1.000000    r[07] =       0.065172    w[07] =       0.090909    C7-H7s
D[08] =       4.086313             -      5.050000      0.500000      1.000000    r[08] =       0.963687    w[08] =       0.090909    C7-H7a
D[09] =       0.172070             -      0.030000      0.500000      1.000000    r[09] =      -0.142070    w[09] =       0.090909    C6-C8
D[10] =      -1.036559             -     -0.850000      0.500000      1.000000    r[10] =       0.186559    w[10] =       0.090909    C6-C9
D[11] =      -0.325408             -     -0.300000      0.500000      1.000000    r[11] =       0.025408    w[11] =       0.090909    C10-C2

Results for Multi-Parameter Fit of Calculated and Experimental Data:
rank  =              5                # rank of cosine matrix (check input if rank < 5)
cond  =   2.128683e+00                # condition number of cosine matrix (check input and singular values if very large)
aic   =      20.665192                # information criterion (AIC) for 5 degrees of freedom
qfac  =       0.101722                # weighted Q-Factor as defined by Cornilescu
r^2   =       0.989495                # coefficient of determination r^2 = 1 - chi^2 / (weighted sum of squares)

(|D|) =       3.498226      3.589091   # mean absolute (calc./exp.) parameter D[i]
|D|min=       0.172070      0.030000   # min. absolute (calc./exp.) parameter D[i]
|D|max=      11.474154     10.650000   # max. absolute (calc./exp.) parameter D[i]
Drange=     -11.474154      7.364803   # min. and max. (calc.)      parameter D[i]
Drange=     -10.650000      8.050000   # min. and max. (exp.)       parameter D[i]

Results for Linear Regression of Calculated and Experimental Data (N=11):
c(b)  =      -0.239876 +/-  0.143757   # linear regression intercept and error
c(m)  =       0.983583 +/-  0.029702   # linear regression slope and error
rmsd  =       0.492332                # unweighted total root-mean-square deviation
chisq =       0.242391                # weighted total sum of squared residuals
maerr =       0.377280                # weighted total mean absolute error (sum of weights = 1.000)
R     =       0.995922                # weighted Pearson correlation coefficient R
R^2   =       0.991860                # weighted Pearson correlation coefficient R^2

E(RDC)=       1.333149                # E(RDC)=1/2*K*(sum of weighted deviations (D(exp)-D(calc))^2), K=1.000
```

3.1.8 (+)-Camphor in Stick S149

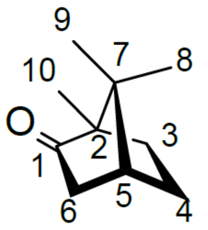

(+)-Camphor

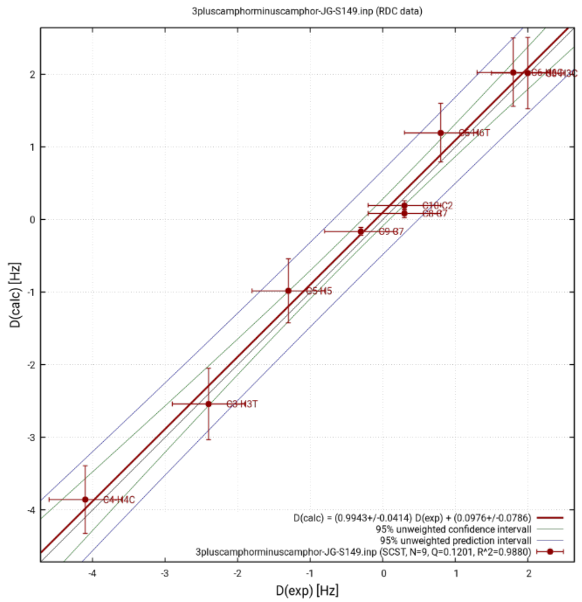


```
================================================================================
info : Start Analysis for Structure '#ak-carvone-1-b3lyp-6311Gdp-1'
in file '3pluscamphorminuscamphor-JG-S149.inp'
================================================================================

--------------------------------------------------------------------------------
info : Start Single-Conformer Single-Tensor (SCST) Fit with 9 RDCs
info : File: '3pluscamphorminuscamphor-JG-S149.inp', Title: '#ak-carvone-1-b3lyp-6311Gdp-1'
--------------------------------------------------------------------------------

--------------------------------------------------------------------------------
SVD Best-Fit Saupe Vector S(zz), S(xx-yy), S(xy), S(xz), S(yz):
 -1.106371e-04  2.629496e-04  8.124900e-05  2.467019e-05 -6.277194e-05
Saupe Tensor (S):                                       Alignment Tensor (A):
  1.867933e-04  8.124900e-05  2.467019e-05                1.245289e-04  5.416600e-05  1.644680e-05
  8.124900e-05 -7.615629e-05 -6.277194e-05                5.416600e-05 -5.077086e-05 -4.184796e-05
  2.467019e-05 -6.277194e-05 -1.106371e-04                1.644680e-05 -4.184796e-05 -7.375803e-05
Trace of Saupe Tensor:        0.000000e+00              Trace of Alignment Tensor:    0.000000e+00
Eigenvectors of Saupe Tensor (S):                       Eigenvectors of Alignment Tensor (A):
  1.840295e-01  1.969572e-01  9.629855e-01                1.840295e-01  1.969572e-01  9.629855e-01
 -7.136077e-01 -6.469674e-01  2.686954e-01               -7.136077e-01 -6.469674e-01  2.686954e-01
  6.759417e-01 -7.366417e-01  2.148911e-02                6.759417e-01 -7.366417e-01  2.148911e-02
Eigenvalues of Saupe Tensor S(xx), S(yy), S(zz):        Eigenvalues of Alignment Tensor A(xx), A(yy), A(zz):
 -3.765060e-05 -1.723636e-04  2.100142e-04               -2.510040e-05 -1.149091e-04  1.400095e-04

Alignment Tensor Irreducible Representation (A0, A1R, A1I, A2R, A2I):
 -1.753963e-04  3.193353e-05 -8.125308e-05  1.701833e-04  1.051701e-04

Tensor Properties: *)
A(axial)      =   2.100142e-04         # alignment tensor axial component   = 3/2*A(zz) = S(zz)
A(rhombic)    =   8.980868e-05         # alignment tensor rhombic component = A(xx) - A(yy) = 2/3*(S(xx) - S(yy))
A(rhombicity) =   4.276314e-01         # alignment tensor rhombicity        = A(rhombic) / A(axial)
A(asymmetry)  =   6.414471e-01         # alignment tensor asymmetry         = 3/2*A(rhombicity) = (A(xx) - A(yy))/A(zz) = (S(xx) - S(yy))/S(zz)
GDO           =   2.239535e-04         # generalized degree of order        = sqrt(3/2)*|A(xx),A(yy),A(zz)| = sqrt(2/3)*|S(xx),S(yy),S(zz)|
----------
*) F. Kramer, M.V. Deshmukh, H. Kessler and S.J. Glaser, Concepts Magn. Res. A, 2004, 21A, 21-40.
```

```
--------------------------------------------------------------------------------
Results for Multi-Parameter SVD Fit of Calculated and Experimental Data:
           D(calc)[HZ]     +/- Error    D(exp)[HZ]     +/- Error  Rel. Weights            D(exp)-D(calc)        Normalized Weights    Atom Labels
D[01] =       2.017491            -      2.000000      0.500000      1.000000    r[01] =      -0.017491    w[01] =       0.111111    C3-H3C
D[02] =      -2.537100            -     -2.400000      0.500000      1.000000    r[02] =       0.137100    w[02] =       0.111111    C3-H3T
D[03] =      -3.854042            -     -4.100000      0.500000      1.000000    r[03] =      -0.245958    w[03] =       0.111111    C4-H4C
D[04] =      -0.980202            -     -1.300000      0.500000      1.000000    r[04] =      -0.319798    w[04] =       0.111111    C5-H5
D[05] =       2.028484            -      1.800000      0.500000      1.000000    r[05] =      -0.228484    w[05] =       0.111111    C6-H6C
D[06] =       1.197469            -      0.800000      0.500000      1.000000    r[06] =      -0.397469    w[06] =       0.111111    C6-H6T
D[07] =       0.086672            -      0.300000      0.500000      1.000000    r[07] =       0.213328    w[07] =       0.111111    C8-C7
D[08] =      -0.160617            -     -0.300000      0.500000      1.000000    r[08] =      -0.139383    w[08] =       0.111111    C9-C7
D[09] =       0.197087            -      0.300000      0.500000      1.000000    r[09] =       0.102913    w[09] =       0.111111    C10-C2

Results for Multi-Parameter Fit of Calculated and Experimental Data:
rank  =             5                # rank of cosine matrix (check input if rank < 5)
cond  =   2.919795e+00               # condition number of cosine matrix (check input and singular values if very large)
aic   =      11.870331               # information criterion (AIC) for 5 degrees of freedom
qfac  =       0.120113               # weighted Q-Factor as defined by Cornilescu
r^2   =       0.985145               # coefficient of determination r^2 = 1 - chi^2 / (weighted sum of squares)

(|D|) =       1.451018      1.477778   # mean absolute (calc./exp.) parameter D[i]
|D|min=       0.086672      0.300000   # min. absolute (calc./exp.) parameter D[i]
|D|max=       3.854042      4.100000   # max. absolute (calc./exp.) parameter D[i]
Drange=      -3.854042      2.028484   # min. and max. (calc.)      parameter D[i]
Drange=      -4.100000      2.000000   # min. and max. (exp.)       parameter D[i]

Results for Linear Regression of Calculated and Experimental Data (N=9):
c(b)  =       0.097638 +/-  0.078550   # linear regression intercept and error
c(m)  =       0.994309 +/-  0.041393   # linear regression slope and error
rmsd  =       0.227933                 # unweighted total root-mean-square deviation
chisq =       0.051954                 # weighted total sum of squared residuals
maerr =       0.200214                 # weighted total mean absolute error (sum of weights = 1.000)
R     =       0.993989                 # weighted Pearson correlation coefficient R
R^2   =       0.988014                 # weighted Pearson correlation coefficient R^2

E(RDC)=       0.233791                 # E(RDC)=1/2*K*(sum of weighted deviations (D(exp)-D(calc))^2), K=1.000

--------------------------------------------------------------------------------
```

3.1.9 (-)-Camphor in Stick S150

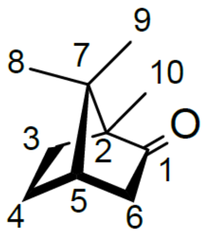

(-)-Camphor

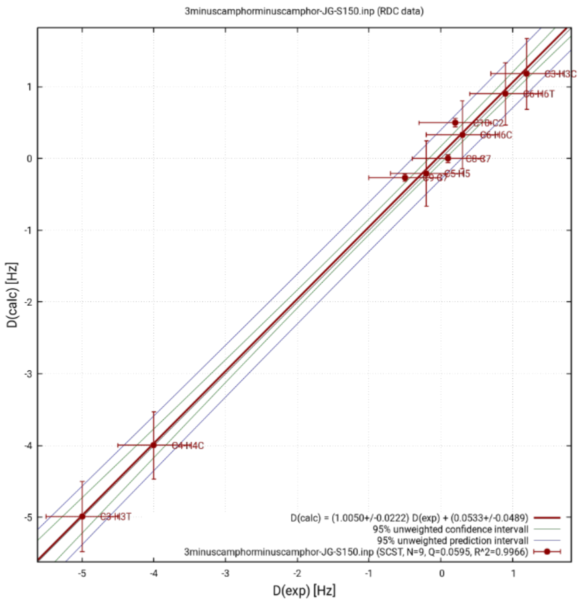


```
================================================================================
info : Start Analysis for Structure '#ak-carvone-1-b3lyp-6311Gdp-1'
in file '3minuscamphorminuscamphor-JG-S150.inp'
================================================================================

--------------------------------------------------------------------------------
info : Start Single-Conformer Single-Tensor (SCST) Fit with 9 RDCs
info : File: '3minuscamphorminuscamphor-JG-S150.inp', Title: '#ak-carvone-1-b3lyp-6311Gdp-1'
--------------------------------------------------------------------------------

--------------------------------------------------------------------------------
SVD Best-Fit Saupe Vector S(zz), S(xx-yy), S(xy), S(xz), S(yz):
 -3.438533e-05  4.394963e-04  1.339148e-06  5.054413e-05 -9.606603e-05
Saupe Tensor (S):                                      Alignment Tensor (A):
  2.369408e-04  1.339148e-06  5.054413e-05               1.579605e-04  8.927653e-07  3.369609e-05
  1.339148e-06 -2.025555e-04 -9.606603e-05               8.927653e-07 -1.350370e-04 -6.404402e-05
  5.054413e-05 -9.606603e-05 -3.438533e-05               3.369609e-05 -6.404402e-05 -2.292356e-05
Trace of Saupe Tensor:         6.776264e-21            Trace of Alignment Tensor:    4.517509e-21
Eigenvectors of Saupe Tensor (S):                      Eigenvectors of Alignment Tensor (A):
  1.873492e-01  9.811980e-01  4.637643e-02               1.873492e-01  9.811980e-01  4.637643e-02
  4.210557e-01 -3.756175e-02 -9.062567e-01               4.210557e-01 -3.756175e-02 -9.062567e-01
 -8.874753e-01  1.893135e-01 -4.201762e-01              -8.874753e-01  1.893135e-01 -4.201762e-01
Eigenvalues of Saupe Tensor S(xx), S(yy), S(zz):       Eigenvalues of Alignment Tensor A(xx), A(yy), A(zz):
  5.223981e-07  2.466416e-04 -2.471640e-04               3.482654e-07  1.644277e-04 -1.647760e-04

Alignment Tensor Irreducible Representation (A0, A1R, A1I, A2R, A2I):
 -5.451213e-05  6.542519e-05 -1.243495e-04  2.844458e-04  1.733416e-06

Tensor Properties: *)
A(axial)      =  -2.471640e-04         # alignment tensor axial component   = 3/2*A(zz) = S(zz)
A(rhombic)    =  -1.640795e-04         # alignment tensor rhombic component = A(xx) - A(yy) = 2/3*(S(xx) - S(yy))
A(rhombicity) =   6.638486e-01         # alignment tensor rhombicity        = A(rhombic) / A(axial)
A(asymmetry)  =   9.957729e-01         # alignment tensor asymmetry         = 3/2*A(rhombicity) = (A(xx) - A(yy))/A(zz) = (S(xx) - S(yy))/S(zz)
GDO           =   2.850993e-04         # generalized degree of order        = sqrt(3/2)*|A(xx),A(yy),A(zz)| = sqrt(2/3)*|S(xx),S(yy),S(zz)|
----------
*) F. Kramer, M.V. Deshmukh, H. Kessler and S.J. Glaser, Concepts Magn. Res. A, 2004, 21A, 21-40.
```

```
--------------------------------------------------------------------------------
Results for Multi-Parameter SVD Fit of Calculated and Experimental Data:
           D(calc)[HZ]     +/- Error    D(exp)[HZ]     +/- Error  Rel. Weights            D(exp)-D(calc)        Normalized Weights    Atom Labels
D[01] =       1.179657            -       1.200000      0.500000      1.000000    r[01] =       0.020343    w[01] =       0.111111    C3-H3C
D[02] =      -4.987712            -      -5.000000      0.500000      1.000000    r[02] =      -0.012288    w[02] =       0.111111    C3-H3T
D[03] =      -3.996905            -      -4.000000      0.500000      1.000000    r[03] =      -0.003095    w[03] =       0.111111    C4-H4C
D[04] =      -0.209758            -      -0.200000      0.500000      1.000000    r[04] =       0.009758    w[04] =       0.111111    C5-H5
D[05] =       0.330589            -       0.300000      0.500000      1.000000    r[05] =      -0.030589    w[05] =       0.111111    C6-H6C
D[06] =       0.900439            -       0.900000      0.500000      1.000000    r[06] =      -0.000439    w[06] =       0.111111    C6-H6T
D[07] =      -0.002138            -       0.100000      0.500000      1.000000    r[07] =       0.102138    w[07] =       0.111111    C8-C7
D[08] =      -0.266179            -      -0.500000      0.500000      1.000000    r[08] =      -0.233821    w[08] =       0.111111    C9-C7
D[09] =       0.496387            -       0.200000      0.500000      1.000000    r[09] =      -0.296387    w[09] =       0.111111    C10-C2

Results for Multi-Parameter Fit of Calculated and Experimental Data:
rank  =             5                # rank of cosine matrix (check input if rank < 5)
cond  =   2.919795e+00               # condition number of cosine matrix (check input and singular values if very large)
aic   =      10.618221               # information criterion (AIC) for 5 degrees of freedom
qfac  =       0.059484               # weighted Q-Factor as defined by Cornilescu
r^2   =       0.995958               # coefficient of determination r^2 = 1 - chi^2 / (weighted sum of squares)

(|D|) =       1.374418      1.377778   # mean absolute (calc./exp.) parameter D[i]
|D|min=       0.002138      0.100000   # min. absolute (calc./exp.) parameter D[i]
|D|max=       4.987712      5.000000   # max. absolute (calc./exp.) parameter D[i]
Drange=      -4.987712      1.179657   # min. and max. (calc.)      parameter D[i]
Drange=      -5.000000      1.200000   # min. and max. (exp.)       parameter D[i]

Results for Linear Regression of Calculated and Experimental Data (N=9):
c(b)  =       0.053262 +/-  0.048861   # linear regression intercept and error
c(m)  =       1.004997 +/-  0.022179   # linear regression slope and error
rmsd  =       0.131045                 # unweighted total root-mean-square deviation
chisq =       0.017173                 # weighted total sum of squared residuals
maerr =       0.078762                 # weighted total mean absolute error (sum of weights = 1.000)
R     =       0.998300                 # weighted Pearson correlation coefficient R
R^2   =       0.996602                 # weighted Pearson correlation coefficient R^2

E(RDC)=       0.077278                 # E(RDC)=1/2*K*(sum of weighted deviations (D(exp)-D(calc))^2), K=1.000

--------------------------------------------------------------------------------
```

3.1.10 (+)-IPC in Stick S151

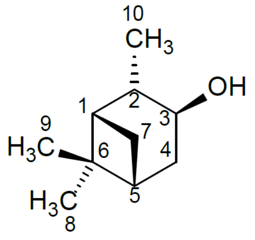

(+)-IPC

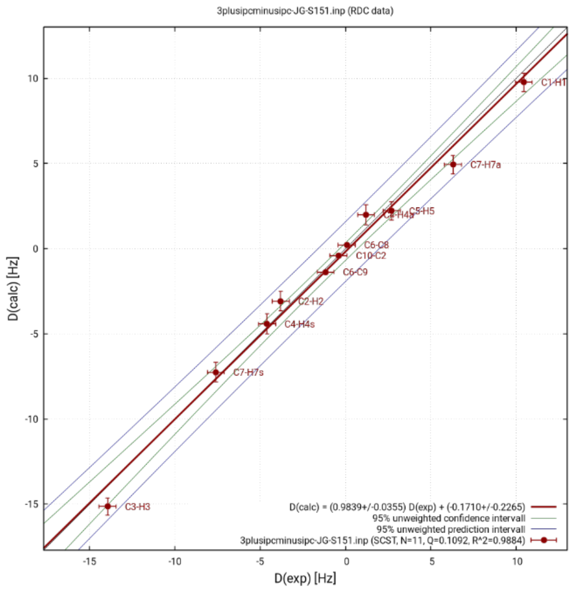


```
================================================================================
info : Start Analysis for Structure 'CalculateOnlyRDCs= 1' in file '3plusipcminusipc-JG-S151.inp'
================================================================================

--------------------------------------------------------------------------------
info : Start Single-Conformer Single-Tensor (SCST) Fit with 11 RDCs
info : File: '3plusipcminusipc-JG-S151.inp', Title: 'CalculateOnlyRDCs= 1'
--------------------------------------------------------------------------------

--------------------------------------------------------------------------------
SVD Best-Fit Saupe Vector S(zz), S(xx-yy), S(xy), S(xz), S(yz):
 -1.090480e-04 -5.067540e-04 -2.751542e-04  3.602620e-04 -3.343929e-04
Saupe Tensor (S):                                       Alignment Tensor (A):
 -1.988530e-04 -2.751542e-04  3.602620e-04               -1.325687e-04 -1.834361e-04  2.401747e-04
 -2.751542e-04  3.079010e-04 -3.343929e-04               -1.834361e-04  2.052674e-04 -2.229286e-04
  3.602620e-04 -3.343929e-04 -1.090480e-04                2.401747e-04 -2.229286e-04 -7.269870e-05
Trace of Saupe Tensor:        1.355253e-20              Trace of Alignment Tensor:    9.035018e-21
Eigenvectors of Saupe Tensor (S):                       Eigenvectors of Alignment Tensor (A):
  5.159760e-01  7.387124e-01 -4.336736e-01                5.159760e-01  7.387124e-01 -4.336736e-01
  6.652281e-01 -2.659917e-02  7.461662e-01                6.652281e-01 -2.659917e-02  7.461662e-01
  5.396669e-01 -6.734957e-01 -5.051368e-01                5.396669e-01 -6.734957e-01 -5.051368e-01
Eigenvalues of Saupe Tensor S(xx), S(yy), S(zz):        Eigenvalues of Alignment Tensor A(xx), A(yy), A(zz):
 -1.767954e-04 -5.174019e-04  6.941973e-04               -1.178636e-04 -3.449346e-04  4.627982e-04

Alignment Tensor Irreducible Representation (A0, A1R, A1I, A2R, A2I):
 -1.728772e-04  4.663293e-04 -4.328439e-04 -3.279756e-04 -3.561643e-04

Tensor Properties: *)
A(axial)      =   6.941973e-04         # alignment tensor axial component   = 3/2*A(zz) = S(zz)
A(rhombic)    =   2.270710e-04         # alignment tensor rhombic component = A(xx) - A(yy) = 2/3*(S(xx) - S(yy))
A(rhombicity) =   3.270986e-01         # alignment tensor rhombicity        = A(rhombic) / A(axial)
A(asymmetry)  =   4.906480e-01         # alignment tensor asymmetry         = 3/2*A(rhombicity) = (A(xx) - A(yy))/A(zz) = (S(xx) - S(yy))/S(zz)
GDO           =   7.215129e-04         # generalized degree of order        = sqrt(3/2)*|A(xx),A(yy),A(zz)| = sqrt(2/3)*|S(xx),S(yy),S(zz)|
----------
*) F. Kramer, M.V. Deshmukh, H. Kessler and S.J. Glaser, Concepts Magn. Res. A, 2004, 21A, 21-40.
```

```
--------------------------------------------------------------------------------
Results for Multi-Parameter SVD Fit of Calculated and Experimental Data:
           D(calc)[HZ]     +/- Error    D(exp)[HZ]     +/- Error  Rel. Weights            D(exp)-D(calc)        Normalized Weights    Atom Labels
D[01] =       9.773409             -     10.450000      0.500000      1.000000    r[01] =       0.676591    w[01] =       0.090909    C1-H1
D[02] =      -3.082609             -     -3.800000      0.500000      1.000000    r[02] =      -0.717391    w[02] =       0.090909    C2-H2
D[03] =     -15.142401             -    -13.950000      0.500000      1.000000    r[03] =       1.192401    w[03] =       0.090909    C3-H3
D[04] =      -4.422832             -     -4.600000      0.500000      1.000000    r[04] =      -0.177168    w[04] =       0.090909    C4-H4s
D[05] =       1.997009             -      1.200000      0.500000      1.000000    r[05] =      -0.797009    w[05] =       0.090909    C4-H4a
D[06] =       2.219336             -      2.700000      0.500000      1.000000    r[06] =       0.480664    w[06] =       0.090909    C5-H5
D[07] =      -7.248418             -     -7.600000      0.500000      1.000000    r[07] =      -0.351582    w[07] =       0.090909    C7-H7s
D[08] =       4.937010             -      6.300000      0.500000      1.000000    r[08] =       1.362990    w[08] =       0.090909    C7-H7a
D[09] =       0.221979             -      0.070000      0.500000      1.000000    r[09] =      -0.151979    w[09] =       0.090909    C6-C8
D[10] =      -1.370541             -     -1.170000      0.500000      1.000000    r[10] =       0.200541    w[10] =       0.090909    C6-C9
D[11] =      -0.399611             -     -0.410000      0.500000      1.000000    r[11] =      -0.010389    w[11] =       0.090909    C10-C2




Results for Multi-Parameter Fit of Calculated and Experimental Data:
rank  =            5                 # rank of cosine matrix (check input if rank < 5)
cond  =   2.128683e+00               # condition number of cosine matrix (check input and singular values if very large)
aic   =     31.346670                # information criterion (AIC) for 5 degrees of freedom
qfac  =      0.109250                # weighted Q-Factor as defined by Cornilescu
r^2   =      0.987774                # coefficient of determination r^2 = 1 - chi^2 / (weighted sum of squares)

(|D|) =      4.619560      4.750000   # mean absolute (calc./exp.) parameter D[i]
|D|min=      0.221979      0.070000   # min. absolute (calc./exp.) parameter D[i]
|D|max=     15.142401     13.950000   # max. absolute (calc./exp.) parameter D[i]
Drange=    -15.142401      9.773409   # min. and max. (calc.)      parameter D[i]
Drange=    -13.950000     10.450000   # min. and max. (exp.)       parameter D[i]

Results for Linear Regression of Calculated and Experimental Data (N=11):
c(b)  =     -0.171035 +/-  0.226513   # linear regression intercept and error
c(m)  =      0.983929 +/-  0.035528   # linear regression slope and error
rmsd  =      0.696528                # unweighted total root-mean-square deviation
chisq =      0.485152                # weighted total sum of squared residuals
maerr =      0.556246                # weighted total mean absolute error (sum of weights = 1.000)
R     =      0.994184                # weighted Pearson correlation coefficient R
R^2   =      0.988402                # weighted Pearson correlation coefficient R^2

E(RDC)=      2.668334                # E(RDC)=1/2*K*(sum of weighted deviations (D(exp)-D(calc))^2), K=1.000

--------------------------------------------------------------------------------
Done in 0.1 seconds on 'Wed Apr  9 16:29:08 2025' - now closing log file '3plusipcminusipc-JG-S151.log'
================================================================================
```

3.1.11 (-)-IPC in Stick S152

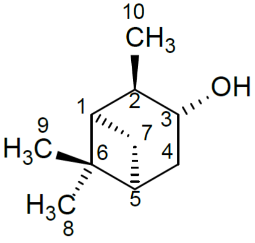

(-)-IPC

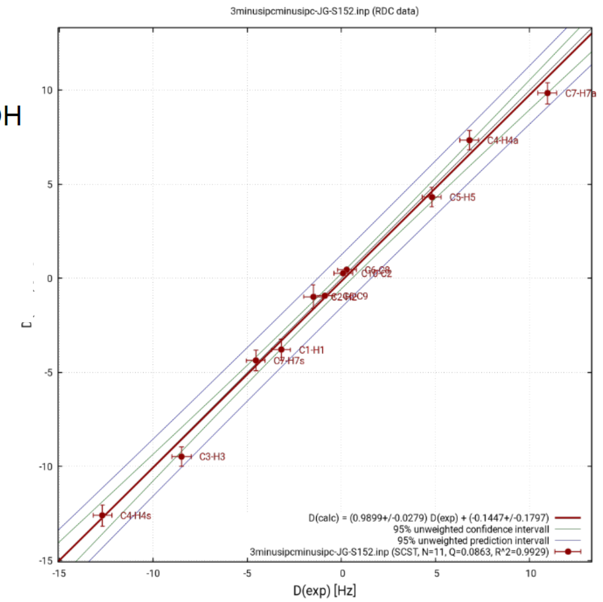


```
--------------------------------------------------------------------------------
info : Start Single-Conformer Single-Tensor (SCST) Fit with 11 RDCs
info : File: '3minusipcminusipc-JG-S152.inp', Title: ''
--------------------------------------------------------------------------------

--------------------------------------------------------------------------------
SVD Best-Fit Saupe Vector S(zz), S(xx-yy), S(xy), S(xz), S(yz):
  4.916383e-04 -3.235317e-04 -1.853349e-04  1.663595e-04 -2.927739e-04
Saupe Tensor (S):                                       Alignment Tensor (A):
 -4.075850e-04 -1.853349e-04  1.663595e-04               -2.717233e-04 -1.235566e-04  1.109063e-04
 -1.853349e-04 -8.405332e-05 -2.927739e-04               -1.235566e-04 -5.603554e-05 -1.951826e-04
  1.663595e-04 -2.927739e-04  4.916383e-04                1.109063e-04 -1.951826e-04  3.277589e-04
Trace of Saupe Tensor:        0.000000e+00              Trace of Alignment Tensor:    0.000000e+00
Eigenvectors of Saupe Tensor (S):                       Eigenvectors of Alignment Tensor (A):
 -3.304402e-01  9.205688e-01 -2.082362e-01               -3.304402e-01  9.205688e-01 -2.082362e-01
  8.293379e-01  3.885259e-01  4.015549e-01                8.293379e-01  3.885259e-01  4.015549e-01
  4.505640e-01 -4.000828e-02 -8.918472e-01                4.505640e-01 -4.000828e-02 -8.918472e-01
Eigenvalues of Saupe Tensor S(xx), S(yy), S(zz):        Eigenvalues of Alignment Tensor A(xx), A(yy), A(zz):
 -1.692674e-04 -4.930356e-04  6.623030e-04               -1.128450e-04 -3.286904e-04  4.415353e-04

Alignment Tensor Irreducible Representation (A0, A1R, A1I, A2R, A2I):
  7.794094e-04  2.153386e-04 -3.789716e-04 -2.093925e-04 -2.399007e-04

Tensor Properties: *)
A(axial)      =   6.623030e-04         # alignment tensor axial component   = 3/2*A(zz) = S(zz)
A(rhombic)    =   2.158454e-04         # alignment tensor rhombic component = A(xx) - A(yy) = 2/3*(S(xx) - S(yy))
A(rhombicity) =   3.259013e-01         # alignment tensor rhombicity        = A(rhombic) / A(axial)
A(asymmetry)  =   4.888520e-01         # alignment tensor asymmetry         = 3/2*A(rhombicity) = (A(xx) - A(yy))/A(zz) = (S(xx) - S(yy))/S(zz)
GDO           =   6.881767e-04         # generalized degree of order        = sqrt(3/2)*|A(xx),A(yy),A(zz)| = sqrt(2/3)*|S(xx),S(yy),S(zz)|
----------
*) F. Kramer, M.V. Deshmukh, H. Kessler and S.J. Glaser, Concepts Magn. Res. A, 2004, 21A, 21-40.
```

```
--------------------------------------------------------------------------------
Results for Multi-Parameter SVD Fit of Calculated and Experimental Data:
           D(calc)[HZ]     +/- Error    D(exp)[HZ]     +/- Error  Rel. Weights            D(exp)-D(calc)        Normalized Weights    Atom Labels
D[01] =      -3.793983            -     -3.200000      0.500000      1.000000    r[01] =       0.593983    w[01] =       0.090909    C1-H1
D[02] =      -0.971387            -     -1.500000      0.500000      1.000000    r[02] =      -0.528613    w[02] =       0.090909    C2-H2
D[03] =      -9.463475            -     -8.500000      0.500000      1.000000    r[03] =       0.963475    w[03] =       0.090909    C3-H3
D[04] =     -12.597294            -    -12.700000      0.500000      1.000000    r[04] =      -0.102706    w[04] =       0.090909    C4-H4s
D[05] =       7.344230            -      6.800000      0.500000      1.000000    r[05] =      -0.544230    w[05] =       0.090909    C4-H4a
D[06] =       4.321683            -      4.800000      0.500000      1.000000    r[06] =       0.478317    w[06] =       0.090909    C5-H5
D[07] =      -4.368429            -     -4.550000      0.500000      1.000000    r[07] =      -0.181571    w[07] =       0.090909    C7-H7s
D[08] =       9.846451            -     10.950000      0.500000      1.000000    r[08] =       1.103549    w[08] =       0.090909    C7-H7a
D[09] =       0.447542            -      0.290000      0.500000      1.000000    r[09] =      -0.157542    w[09] =       0.090909    C6-C8
D[10] =      -0.932374            -     -0.880000      0.500000      1.000000    r[10] =       0.052374    w[10] =       0.090909    C6-C9
D[11] =       0.270207            -      0.100000      0.500000      1.000000    r[11] =      -0.170207    w[11] =       0.090909    C10-C2


Results for Multi-Parameter Fit of Calculated and Experimental Data:
rank  =              5                # rank of cosine matrix (check input if rank < 5)
cond  =   2.128683e+00                # condition number of cosine matrix (check input and singular values if very large)
aic   =      23.613495                # information criterion (AIC) for 5 degrees of freedom
qfac  =       0.086318                # weighted Q-Factor as defined by Cornilescu
r^2   =       0.992443                # coefficient of determination r^2 = 1 - chi^2 / (weighted sum of squares)

(|D|) =       4.941550      4.933636   # mean absolute (calc./exp.) parameter D[i]
|D|min=       0.270207      0.100000   # min. absolute (calc./exp.) parameter D[i]
|D|max=      12.597294     12.700000   # max. absolute (calc./exp.) parameter D[i]
Drange=     -12.597294      9.846451   # min. and max. (calc.)      parameter D[i]
Drange=     -12.700000     10.950000   # min. and max. (exp.)       parameter D[i]

Results for Linear Regression of Calculated and Experimental Data (N=11):
c(b)  =      -0.144694 +/-  0.179666   # linear regression intercept and error
c(m)  =       0.989892 +/-  0.027881   # linear regression slope and error
rmsd  =       0.556235                # unweighted total root-mean-square deviation
chisq =       0.309398                # weighted total sum of squared residuals
maerr =       0.443324                # weighted total mean absolute error (sum of weights = 1.000)
R     =       0.996449                # weighted Pearson correlation coefficient R
R^2   =       0.992911                # weighted Pearson correlation coefficient R^2

E(RDC)=       1.701687                # E(RDC)=1/2*K*(sum of weighted deviations (D(exp)-D(calc))^2), K=1.000

--------------------------------------------------------------------------------
```

3.1.12 (+)-(α)-Pinene in Stick S153

```
================================================================================
info : Start Analysis for Structure '#ak-apinene-ACIFEX-b3lyp-6311Gdp-1.xyz'
in file '3pluspinenminuspinen_S153.inp'
================================================================================

--------------------------------------------------------------------------------
info : Start Single-Conformer Single-Tensor (SCST) Fit with 10 RDCs
info : File: '3pluspinenminuspinen_S153.inp', Title: '#ak-apinene-ACIFEX-b3lyp-6311Gdp-1.xyz'
--------------------------------------------------------------------------------

--------------------------------------------------------------------------------
SVD Best-Fit Saupe Vector S(zz), S(xx-yy), S(xy), S(xz), S(yz):
  9.314798e-05 -7.256646e-05  5.044199e-05  1.809777e-07  2.364422e-05
Saupe Tensor (S):                                     Alignment Tensor (A):
 -8.285722e-05  5.044199e-05  1.809777e-07             -5.523815e-05  3.362799e-05  1.206518e-07
  5.044199e-05 -1.029076e-05  2.364422e-05              3.362799e-05 -6.860506e-06  1.576281e-05
  1.809777e-07  2.364422e-05  9.314798e-05              1.206518e-07  1.576281e-05  6.209866e-05
Trace of Saupe Tensor:        0.000000e+00            Trace of Alignment Tensor:    0.000000e+00
Eigenvectors of Saupe Tensor (S):                     Eigenvectors of Alignment Tensor (A):
  4.615937e-01 -6.769562e-02 -8.845047e-01              4.615937e-01 -6.769562e-02 -8.845047e-01
  8.528119e-01 -2.406368e-01  4.634714e-01              8.528119e-01 -2.406368e-01  4.634714e-01
 -2.442194e-01 -9.682516e-01 -5.334481e-02             -2.442194e-01 -9.682516e-01 -5.334481e-02
Eigenvalues of Saupe Tensor S(xx), S(yy), S(zz):      Eigenvalues of Alignment Tensor A(xx), A(yy), A(zz):
  1.024053e-05  9.903687e-05 -1.092774e-04              6.827018e-06  6.602458e-05 -7.285160e-05

Alignment Tensor Irreducible Representation (A0, A1R, A1I, A2R, A2I):
  1.476704e-04  2.342607e-07  3.060548e-05 -4.696564e-05  6.529298e-05

Tensor Properties: *)
A(axial)      =  -1.092774e-04         # alignment tensor axial component   = 3/2*A(zz) = S(zz)
A(rhombic)    =  -5.919756e-05         # alignment tensor rhombic component = A(xx) - A(yy) = 2/3*(S(xx) - S(yy))
A(rhombicity) =   5.417183e-01         # alignment tensor rhombicity        = A(rhombic) / A(axial)
A(asymmetry)  =   8.125774e-01         # alignment tensor asymmetry         = 3/2*A(rhombicity) = (A(xx) - A(yy))/A(zz) = (S(xx) - S(yy))/S(zz)
GDO           =   1.207055e-04         # generalized degree of order        = sqrt(3/2)*|A(xx),A(yy),A(zz)| = sqrt(2/3)*|S(xx),S(yy),S(zz)|
----------
*) F. Kramer, M.V. Deshmukh, H. Kessler and S.J. Glaser, Concepts Magn. Res. A, 2004, 21A, 21-40.
```

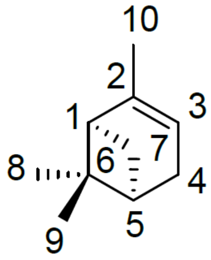

(+)-α-Pinene

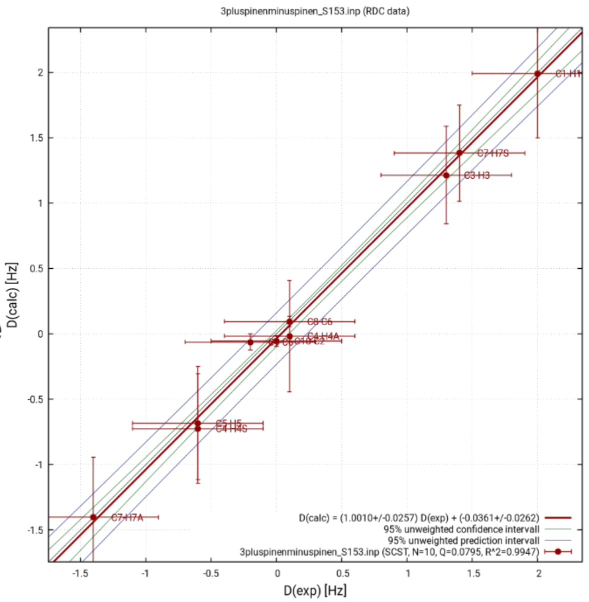

```
--------------------------------------------------------------------------------
Results for Multi-Parameter SVD Fit of Calculated and Experimental Data:
           D(calc)[HZ]     +/- Error    D(exp)[HZ]     +/- Error  Rel. Weights            D(exp)-D(calc)        Normalized Weights    Atom Labels
D[01] =      -0.061634            -     -0.200000      0.500000      1.000000    r[01] =      -0.138366    w[01] =       0.100000    C9-C6
D[02] =      -0.054561            -      0.000000      0.500000      1.000000    r[02] =       0.054561    w[02] =       0.100000    C10-C2
D[03] =       0.094702            -      0.100000      0.500000      1.000000    r[03] =       0.005298    w[03] =       0.100000    C8-C6
D[04] =      -0.723406            -     -0.600000      0.500000      1.000000    r[04] =       0.123406    w[04] =       0.100000    C4-H4S
D[05] =      -0.016343            -      0.100000      0.500000      1.000000    r[05] =       0.116343    w[05] =       0.100000    C4-H4A
D[06] =       1.382497            -      1.400000      0.500000      1.000000    r[06] =       0.017503    w[06] =       0.100000    C7-H7S
D[07] =      -1.402695            -     -1.400000      0.500000      1.000000    r[07] =       0.002695    w[07] =       0.100000    C7-H7A
D[08] =      -0.683355            -     -0.600000      0.500000      1.000000    r[08] =       0.083355    w[08] =       0.100000    C5-H5
D[09] =       1.991114            -      2.000000      0.500000      1.000000    r[09] =       0.008886    w[09] =       0.100000    C1-H1
D[10] =       1.214383            -      1.300000      0.500000      1.000000    r[10] =       0.085617    w[10] =       0.100000    C3-H3


Results for Multi-Parameter Fit of Calculated and Experimental Data:
rank  =             5                # rank of cosine matrix (check input if rank < 5)
cond  =   2.232186e+00               # condition number of cosine matrix (check input and singular values if very large)
aic   =      10.262343               # information criterion (AIC) for 5 degrees of freedom
qfac  =       0.079451               # weighted Q-Factor as defined by Cornilescu
r^2   =       0.993408               # coefficient of determination r^2 = 1 - chi^2 / (weighted sum of squares)

(|D|) =       0.762469      0.770000   # mean absolute (calc./exp.) parameter D[i]
|D|min=       0.016343      0.000000   # min. absolute (calc./exp.) parameter D[i]
|D|max=       1.991114      2.000000   # max. absolute (calc./exp.) parameter D[i]
Drange=      -1.402695      1.991114   # min. and max. (calc.)      parameter D[i]
Drange=      -1.400000      2.000000   # min. and max. (exp.)       parameter D[i]

Results for Linear Regression of Calculated and Experimental Data (N=10):
c(b)  =      -0.036138 +/-  0.026220   # linear regression intercept and error
c(m)  =       1.000992 +/-  0.025724   # linear regression slope and error
rmsd  =       0.080985                 # unweighted total root-mean-square deviation
chisq =       0.006559                 # weighted total sum of squared residuals
maerr =       0.063603                 # weighted total mean absolute error (sum of weights = 1.000)
R     =       0.997369                 # weighted Pearson correlation coefficient R
R^2   =       0.994745                 # weighted Pearson correlation coefficient R^2

E(RDC)=       0.032793                 # E(RDC)=1/2*K*(sum of weighted deviations (D(exp)-D(calc))^2), K=1.000
```

3.1.13 (-)-(α)-Pinene in Stick S154

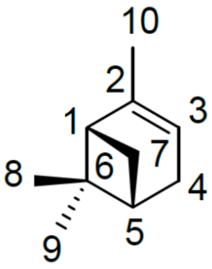

(-)-α-Pinene

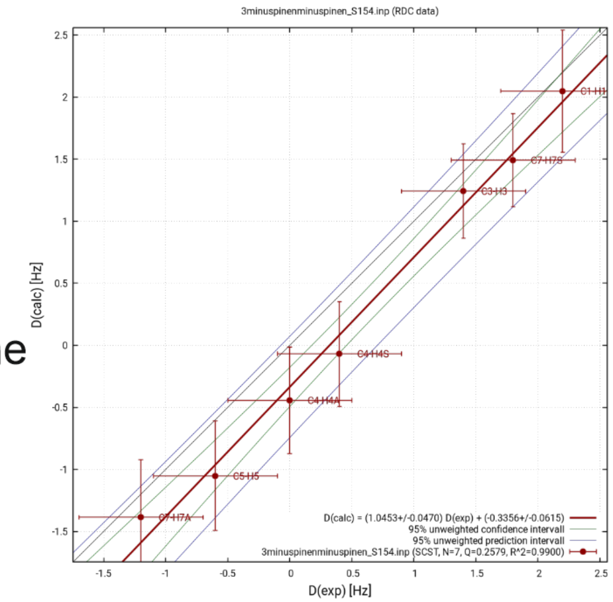


```
================================================================================
info : Start Analysis for Structure '#ak-apinene-ACIFEX-b3lyp-6311Gdp-1.xyz'
in file '3minuspinenminuspinen_S154.inp'
================================================================================

--------------------------------------------------------------------------------
info : Start Single-Conformer Single-Tensor (SCST) Fit with 7 RDCs
info : File: '3minuspinenminuspinen_S154.inp',
Title: '#ak-apinene-ACIFEX-b3lyp-6311Gdp-1.xyz'
--------------------------------------------------------------------------------

--------------------------------------------------------------------------------
SVD Best-Fit Saupe Vector S(zz), S(xx-yy), S(xy), S(xz), S(yz):
  8.115878e-05 -1.036783e-04  5.950044e-05  1.281033e-05  8.665255e-06
Saupe Tensor (S):                                      Alignment Tensor (A):
 -9.241854e-05  5.950044e-05  1.281033e-05              -6.161236e-05  3.966696e-05  8.540221e-06
  5.950044e-05  1.125976e-05  8.665255e-06               3.966696e-05  7.506506e-06  5.776837e-06
  1.281033e-05  8.665255e-06  8.115878e-05               8.540221e-06  5.776837e-06  5.410585e-05
Trace of Saupe Tensor:        0.000000e+00             Trace of Alignment Tensor:    0.000000e+00
Eigenvectors of Saupe Tensor (S):                      Eigenvectors of Alignment Tensor (A):
  3.858138e-01 -1.466955e-01 -9.108393e-01               3.858138e-01 -1.466955e-01 -9.108393e-01
  8.820362e-01 -2.307986e-01  4.107847e-01               8.820362e-01 -2.307986e-01  4.107847e-01
 -2.704807e-01 -9.618796e-01  4.034546e-02              -2.704807e-01 -9.618796e-01  4.034546e-02
Eigenvalues of Saupe Tensor S(xx), S(yy), S(zz):       Eigenvalues of Alignment Tensor A(xx), A(yy), A(zz):
  3.462876e-05  8.519166e-05 -1.198204e-04               2.308584e-05  5.679444e-05 -7.988028e-05

Alignment Tensor Irreducible Representation (A0, A1R, A1I, A2R, A2I):
  1.286635e-04  1.658191e-05  1.121646e-05 -6.710149e-05  7.701840e-05

Tensor Properties: *)
A(axial)      =  -1.198204e-04         # alignment tensor axial component   = 3/2*A(zz) = S(zz)
A(rhombic)    =  -3.370860e-05         # alignment tensor rhombic component = A(xx) - A(yy) = 2/3*(S(xx) - S(yy))
A(rhombicity) =   2.813260e-01         # alignment tensor rhombicity        = A(rhombic) / A(axial)
A(asymmetry)  =   4.219890e-01         # alignment tensor asymmetry         = 3/2*A(rhombicity) = (A(xx) - A(yy))/A(zz) = (S(xx) - S(yy))/S(zz)
GDO           =   1.233253e-04         # generalized degree of order        = sqrt(3/2)*|A(xx),A(yy),A(zz)| = sqrt(2/3)*|S(xx),S(yy),S(zz)|
----------
*) F. Kramer, M.V. Deshmukh, H. Kessler and S.J. Glaser, Concepts Magn. Res. A, 2004, 21A, 21-40.
```

```
--------------------------------------------------------------------------------
Results for Multi-Parameter SVD Fit of Calculated and Experimental Data:
           D(calc)[HZ]     +/- Error    D(exp)[HZ]     +/- Error  Rel. Weights            D(exp)-D(calc)        Normalized Weights    Atom Labels
D[01] =      -0.068412            -      0.400000      0.500000      1.000000    r[01] =       0.468412    w[01] =       0.142857    C4-H4S
D[02] =      -0.443624            -      0.000000      0.500000      1.000000    r[02] =       0.443624    w[02] =       0.142857    C4-H4A
D[03] =       1.491781            -      1.800000      0.500000      1.000000    r[03] =       0.308219    w[03] =       0.142857    C7-H7S
D[04] =      -1.386715            -     -1.200000      0.500000      1.000000    r[04] =       0.186715    w[04] =       0.142857    C7-H7A
D[05] =      -1.052564            -     -0.600000      0.500000      1.000000    r[05] =       0.452564    w[05] =       0.142857    C5-H5
D[06] =       2.049685            -      2.200000      0.500000      1.000000    r[06] =       0.150315    w[06] =       0.142857    C1-H1
D[07] =       1.242008            -      1.400000      0.500000      1.000000    r[07] =       0.157992    w[07] =       0.142857    C3-H3


Results for Multi-Parameter Fit of Calculated and Experimental Data:
rank  =            5                # rank of cosine matrix (check input if rank < 5)
cond  =   2.242905e+00              # condition number of cosine matrix (check input and singular values if very large)
aic   =     13.193775               # information criterion (AIC) for 5 degrees of freedom
qfac  =      0.257948 (!)           # weighted Q-Factor as defined by Cornilescu
r^2   =      0.917807               # coefficient of determination r^2 = 1 - chi^2 / (weighted sum of squares)

(|D|) =      1.104970      1.085714   # mean absolute (calc./exp.) parameter D[i]
|D|min=      0.068412      0.000000   # min. absolute (calc./exp.) parameter D[i]
|D|max=      2.049685      2.200000   # max. absolute (calc./exp.) parameter D[i]
Drange=     -1.386715      2.049685   # min. and max. (calc.)      parameter D[i]
Drange=     -1.200000      2.200000   # min. and max. (exp.)       parameter D[i]

Results for Linear Regression of Calculated and Experimental Data (N=7):
c(b)  =     -0.335593 +/-  0.061488   # linear regression intercept and error
c(m)  =      1.045327 +/-  0.046962   # linear regression slope and error
rmsd  =      0.337733                 # unweighted total root-mean-square deviation
chisq =      0.114063                 # weighted total sum of squared residuals
maerr =      0.309692                 # weighted total mean absolute error (sum of weights = 1.000)
R     =      0.994992                 # weighted Pearson correlation coefficient R
R^2   =      0.990009                 # weighted Pearson correlation coefficient R^2

E(RDC)=      0.399222                 # E(RDC)=1/2*K*(sum of weighted deviations (D(exp)-D(calc))^2), K=1.000
```

3.1.14 (+)-Menthol in Stick S155

```
===============================================================================
info : Start Analysis for Structure '#ak-menthol-1-b3lyp-6311Gdp-1'
in file '7plusmentholminusmenthol_Stick155.inp'
===============================================================================

-------------------------------------------------------------------------------
info : Start Single-Conformer Single-Tensor (SCST) Fit with 13 RDCs
info : File: '7plusmentholminusmenthol_Stick155.inp', Title: '#ak-menthol-1-b3lyp-6311Gdp-1'
-------------------------------------------------------------------------------

-------------------------------------------------------------------------------
SVD Best-Fit Saupe Vector S(zz), S(xx-yy), S(xy), S(xz), S(yz):
  6.876741e-04 -1.549961e-04  2.269687e-05  5.323649e-04 -2.143299e-04
Saupe Tensor (S):                                      Alignment Tensor (A):
 -4.213351e-04  2.269687e-05  5.323649e-04              -2.808901e-04  1.513125e-05  3.549099e-04
  2.269687e-05 -2.663390e-04 -2.143299e-04               1.513125e-05 -1.775594e-04 -1.428866e-04
  5.323649e-04 -2.143299e-04  6.876741e-04               3.549099e-04 -1.428866e-04  4.584494e-04
Trace of Saupe Tensor:        0.000000e+00             Trace of Alignment Tensor:    0.000000e+00
Eigenvectors of Saupe Tensor (S):                      Eigenvectors of Alignment Tensor (A):
 -3.016321e-01 -8.831983e-01 -3.591364e-01              -3.016321e-01 -8.831983e-01 -3.591364e-01
 -9.523386e-01  2.611245e-01  1.576869e-01              -9.523386e-01  2.611245e-01  1.576869e-01
 -4.548950e-02  3.895829e-01 -9.198673e-01              -4.548950e-02  3.895829e-01 -9.198673e-01
Eigenvalues of Saupe Tensor S(xx), S(yy), S(zz):       Eigenvalues of Alignment Tensor A(xx), A(yy), A(zz):
 -2.693880e-04 -6.628743e-04  9.322623e-04              -1.795920e-04 -4.419162e-04  6.215082e-04

Alignment Tensor Irreducible Representation (A0, A1R, A1I, A2R, A2I):
  1.090191e-03  6.891023e-04 -2.774323e-04 -1.003148e-04  2.937922e-05

Tensor Properties: *)
A(axial)       =   9.322623e-04         # alignment tensor axial component   = 3/2*A(zz) = S(zz)
A(rhombic)     =   2.623242e-04         # alignment tensor rhombic component = A(xx) - A(yy) = 2/3*(S(xx) - S(yy))
A(rhombicity)  =   2.813845e-01         # alignment tensor rhombicity        = A(rhombic) / A(axial)
A(asymmetry)   =   4.220768e-01         # alignment tensor asymmetry         = 3/2*A(rhombicity) = (A(xx) - A(yy))/A(zz) = (S(xx) - S(yy))/S(zz)
GDO            =   9.595433e-04         # generalized degree of order        = sqrt(3/2)*|A(xx),A(yy),A(zz)| = sqrt(2/3)*|S(xx),S(yy),S(zz)|
----------
*) F. Kramer, M.V. Deshmukh, H. Kessler and S.J. Glaser, Concepts Magn. Res. A, 2004, 21A, 21-40.
```

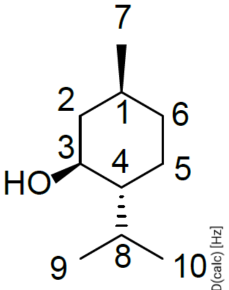

(+)-Menthol

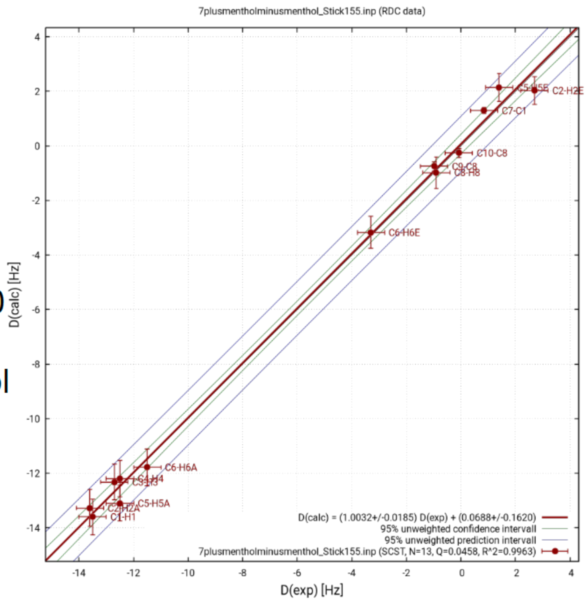

```
--------------------------------------------------------------------------------
Results for Multi-Parameter SVD Fit of Calculated and Experimental Data:
           D(calc)[HZ]     +/- Error    D(exp)[HZ]     +/- Error  Rel. Weights            D(exp)-D(calc)        Normalized Weights    Atom Labels
D[01] =     -13.596176            -    -13.500000      0.500000      1.000000    r[01] =       0.096176    w[01] =       0.076923    C1-H1
D[02] =     -13.273977            -    -13.600000      0.500000      1.000000    r[02] =      -0.326023    w[02] =       0.076923    C2-H2A
D[03] =       2.029577            -      2.700000      0.500000      1.000000    r[03] =       0.670423    w[03] =       0.076923    C2-H2E
D[04] =     -12.321436            -    -12.700000      0.500000      1.000000    r[04] =      -0.378564    w[04] =       0.076923    C3-H3
D[05] =     -12.191386            -    -12.500000      0.500000      1.000000    r[05] =      -0.308614    w[05] =       0.076923    C4-H4
D[06] =     -13.097740            -    -12.500000      0.500000      1.000000    r[06] =       0.597740    w[06] =       0.076923    C5-H5A
D[07] =       2.138525            -      1.400000      0.500000      1.000000    r[07] =      -0.738525    w[07] =       0.076923    C5-H5E
D[08] =      -3.172044            -     -3.300000      0.500000      1.000000    r[08] =      -0.127956    w[08] =       0.076923    C6-H6E
D[09] =     -11.779085            -    -11.500000      0.500000      1.000000    r[09] =       0.279085    w[09] =       0.076923    C6-H6A
D[10] =       1.292580            -      0.850000      0.500000      1.000000    r[10] =      -0.442580    w[10] =       0.076923    C7-C1
D[11] =      -0.972351            -     -0.900000      0.500000      1.000000    r[11] =       0.072351    w[11] =       0.076923    C8-H8
D[12] =      -0.258683            -     -0.070000      0.500000      1.000000    r[12] =       0.188683    w[12] =       0.076923    C10-C8
D[13] =      -0.747984            -     -0.980000      0.500000      1.000000    r[13] =      -0.232016    w[13] =       0.076923    C9-C8

Results for Multi-Parameter Fit of Calculated and Experimental Data:
rank  =              5                 # rank of cosine matrix (check input if rank < 5)
cond  =   2.005893e+01                 # condition number of cosine matrix (check input and singular values if very large)
aic   =      18.364320                 # information criterion (AIC) for 5 degrees of freedom
qfac  =       0.045823                 # weighted Q-Factor as defined by Cornilescu
r^2   =       0.996160                 # coefficient of determination r^2 = 1 - chi^2 / (weighted sum of squares)

(|D|) =       6.682426      6.653846   # mean absolute (calc./exp.) parameter D[i]
|D|min=       0.258683      0.070000   # min. absolute (calc./exp.) parameter D[i]
|D|max=      13.596176     13.600000   # max. absolute (calc./exp.) parameter D[i]
Drange=     -13.596176      2.138525   # min. and max. (calc.)      parameter D[i]
Drange=     -13.600000      2.700000   # min. and max. (exp.)       parameter D[i]

Results for Linear Regression of Calculated and Experimental Data (N=13):
c(b)  =       0.068791 +/-  0.162040   # linear regression intercept and error
c(m)  =       1.003191 +/-  0.018514   # linear regression slope and error
rmsd  =       0.401064                 # unweighted total root-mean-square deviation
chisq =       0.160852                 # weighted total sum of squared residuals
maerr =       0.342980                 # weighted total mean absolute error (sum of weights = 1.000)
R     =       0.998132                 # weighted Pearson correlation coefficient R
R^2   =       0.996268                 # weighted Pearson correlation coefficient R^2

E(RDC)=       1.045540                 # E(RDC)=1/2*K*(sum of weighted deviations (D(exp)-D(calc))^2), K=1.000
```

3.1.15 (-)-Menthol in Stick S156

```
================================================================================
info : Start Analysis for Structure
'#ak-menthol-1-b3lyp-6311Gdp-1' in file '5minusmentholminusmenthol_Stick156.inp'
================================================================================

--------------------------------------------------------------------------------
info : Start Single-Conformer Single-Tensor (SCST) Fit with 11 RDCs
info : File: '5minusmentholminusmenthol_Stick156.inp',
Title: '#ak-menthol-1-b3lyp-6311Gdp-1'
--------------------------------------------------------------------------------

--------------------------------------------------------------------------------
SVD Best-Fit Saupe Vector S(zz), S(xx-yy), S(xy), S(xz), S(yz):
  7.020583e-04 -7.023184e-04 -1.042109e-07  8.811086e-06 -1.949554e-04
Saupe Tensor (S):                                      Alignment Tensor (A):
 -7.021884e-04 -1.042109e-07  8.811086e-06              -4.681256e-04 -6.947396e-08  5.874057e-06
 -1.042109e-07  1.300519e-07 -1.949554e-04              -6.947396e-08  8.670124e-08 -1.299702e-04
  8.811086e-06 -1.949554e-04  7.020583e-04               5.874057e-06 -1.299702e-04  4.680389e-04
Trace of Saupe Tensor:        0.000000e+00             Trace of Alignment Tensor:    0.000000e+00
Eigenvectors of Saupe Tensor (S):                      Eigenvectors of Alignment Tensor (A):
 -3.235237e-03 -9.999775e-01 -5.880812e-03              -3.235237e-03 -9.999775e-01 -5.880812e-03
 -9.680385e-01  1.656987e-03  2.507960e-01              -9.680385e-01  1.656987e-03  2.507960e-01
 -2.507806e-01  6.504238e-03 -9.680221e-01              -2.507806e-01  6.504238e-03 -9.680221e-01
Eigenvalues of Saupe Tensor S(xx), S(yy), S(zz):       Eigenvalues of Alignment Tensor A(xx), A(yy), A(zz):
 -5.037554e-05 -7.022455e-04  7.526211e-04              -3.358369e-05 -4.681637e-04  5.017474e-04

Alignment Tensor Irreducible Representation (A0, A1R, A1I, A2R, A2I):
  1.112995e-03  1.140522e-05 -2.523536e-04 -4.545466e-04 -1.348924e-07

Tensor Properties: *)
A(axial)      =   7.526211e-04        # alignment tensor axial component   = 3/2*A(zz) = S(zz)
A(rhombic)    =   4.345800e-04        # alignment tensor rhombic component = A(xx) - A(yy) = 2/3*(S(xx) - S(yy))
A(rhombicity) =   5.774220e-01        # alignment tensor rhombicity        = A(rhombic) / A(axial)
A(asymmetry)  =   8.661331e-01        # alignment tensor asymmetry         = 3/2*A(rhombicity) = (A(xx) - A(yy))/A(zz) = (S(xx) - S(yy))/S(zz)
GDO           =   8.414768e-04        # generalized degree of order        = sqrt(3/2)*|A(xx),A(yy),A(zz)| = sqrt(2/3)*|S(xx),S(yy),S(zz)|
----------
*) F. Kramer, M.V. Deshmukh, H. Kessler and S.J. Glaser, Concepts Magn. Res. A, 2004, 21A, 21-40.
```

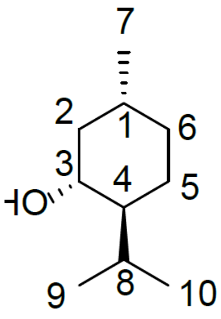

(-)-Menthol

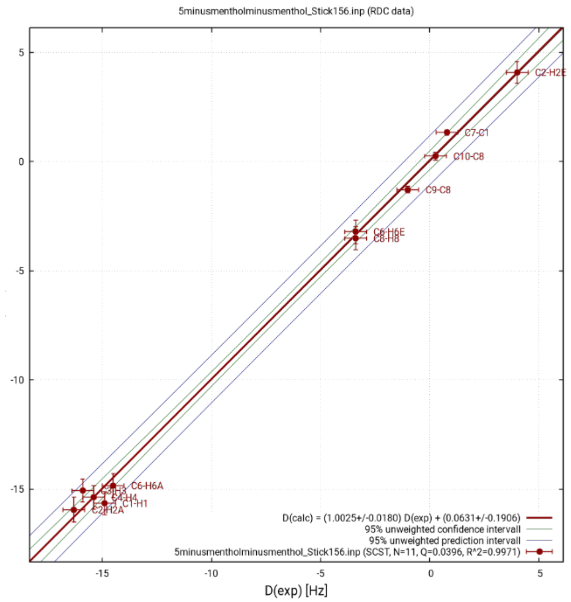

```
--------------------------------------------------------------------------------
Results for Multi-Parameter SVD Fit of Calculated and Experimental Data:
           D(calc)[HZ]     +/- Error    D(exp)[HZ]     +/- Error  Rel. Weights            D(exp)-D(calc)        Normalized Weights    Atom Labels
D[01] =     -15.654796            -    -14.900000      0.500000      1.000000    r[01] =       0.754796    w[01] =       0.090909    C1-H1
D[02] =     -15.949703            -    -16.300000      0.500000      1.000000    r[02] =      -0.350297    w[02] =       0.090909    C2-H2A
D[03] =       4.088193            -      4.000000      0.500000      1.000000    r[03] =      -0.088193    w[03] =       0.090909    C2-H2E
D[04] =     -15.077238            -    -15.900000      0.500000      1.000000    r[04] =      -0.822762    w[04] =       0.090909    C3-H3
D[05] =     -15.383604            -    -15.400000      0.500000      1.000000    r[05] =      -0.016396    w[05] =       0.090909    C4-H4
D[06] =      -3.215486            -     -3.400000      0.500000      1.000000    r[06] =      -0.184514    w[06] =       0.090909    C6-H6E
D[07] =     -14.846818            -    -14.500000      0.500000      1.000000    r[07] =       0.346818    w[07] =       0.090909    C6-H6A
D[08] =       1.333523            -      0.780000      0.500000      1.000000    r[08] =      -0.553523    w[08] =       0.090909    C7-C1
D[09] =      -3.519373            -     -3.400000      0.500000      1.000000    r[09] =       0.119373    w[09] =       0.090909    C8-H8
D[10] =       0.246693            -      0.270000      0.500000      1.000000    r[10] =       0.023307    w[10] =       0.090909    C10-C8
D[11] =      -1.288770            -     -1.010000      0.500000      1.000000    r[11] =       0.278770    w[11] =       0.090909    C9-C8


Results for Multi-Parameter Fit of Calculated and Experimental Data:
rank  =            5                # rank of cosine matrix (check input if rank < 5)
cond  =   1.940082e+01              # condition number of cosine matrix (check input and singular values if very large)
aic   =      17.722523              # information criterion (AIC) for 5 degrees of freedom
qfac  =       0.039639              # weighted Q-Factor as defined by Cornilescu
r^2   =       0.997032              # coefficient of determination r^2 = 1 - chi^2 / (weighted sum of squares)

(|D|) =       8.236745      8.169091   # mean absolute (calc./exp.) parameter D[i]
|D|min=       0.246693      0.270000   # min. absolute (calc./exp.) parameter D[i]
|D|max=      15.949703     16.300000   # max. absolute (calc./exp.) parameter D[i]
Drange=     -15.949703      4.088193   # min. and max. (calc.)      parameter D[i]
Drange=     -16.300000      4.000000   # min. and max. (exp.)       parameter D[i]

Results for Linear Regression of Calculated and Experimental Data (N=11):
c(b)  =       0.063082 +/-  0.190635   # linear regression intercept and error
c(m)  =       1.002524 +/-  0.018037   # linear regression slope and error
rmsd  =       0.418941                 # unweighted total root-mean-square deviation
chisq =       0.175512                 # weighted total sum of squared residuals
maerr =       0.321704                 # weighted total mean absolute error (sum of weights = 1.000)
R     =       0.998547                 # weighted Pearson correlation coefficient R
R^2   =       0.997095                 # weighted Pearson correlation coefficient R^2

E(RDC)=       0.965315                 # E(RDC)=1/2*K*(sum of weighted deviations (D(exp)-D(calc))^2), K=1.000
```

3.1.16 (+)-IPC in Stick S167

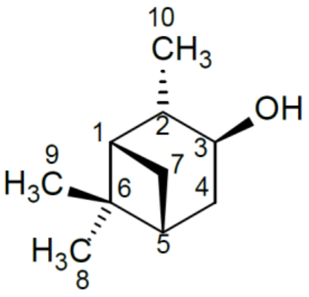

(+)-IPC

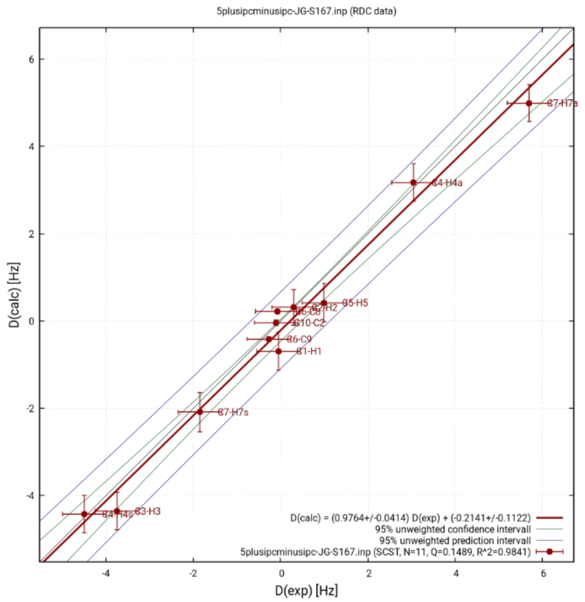


```
================================================================================
info : Start Analysis for Structure '#OutputFileName = ipc_kw188-1' in file
'5plusipcminusipc-JG-S167.inp'
================================================================================

--------------------------------------------------------------------------------
info : Start Single-Conformer Single-Tensor (SCST) Fit with 11 RDCs
info : File: '5plusipcminusipc-JG-S167.inp', Title: '#OutputFileName = ipc_kw188-1'
--------------------------------------------------------------------------------

--------------------------------------------------------------------------------
SVD Best-Fit Saupe Vector S(zz), S(xx-yy), S(xy), S(xz), S(yz):
  1.656592e-04 -2.354967e-04 -1.125815e-04  7.344057e-05 -1.012504e-04
Saupe Tensor (S):                                        Alignment Tensor (A):
 -2.005780e-04 -1.125815e-04  7.344057e-05                -1.337186e-04 -7.505435e-05  4.896038e-05
 -1.125815e-04  3.491878e-05 -1.012504e-04                -7.505435e-05  2.327919e-05 -6.750027e-05
  7.344057e-05 -1.012504e-04  1.656592e-04                 4.896038e-05 -6.750027e-05  1.104395e-04
Trace of Saupe Tensor:        -2.710505e-20              Trace of Alignment Tensor:    -1.807004e-20
Eigenvectors of Saupe Tensor (S):                        Eigenvectors of Alignment Tensor (A):
  2.377499e-01 -9.358510e-01 -2.601113e-01                 2.377499e-01 -9.358510e-01 -2.601113e-01
 -7.861873e-01 -3.426688e-01  5.142836e-01                -7.861873e-01 -3.426688e-01  5.142836e-01
 -5.704249e-01  8.222532e-02 -8.172236e-01                -5.704249e-01  8.222532e-02 -8.172236e-01
Eigenvalues of Saupe Tensor S(xx), S(yy), S(zz):         Eigenvalues of Alignment Tensor A(xx), A(yy), A(zz):
 -4.498676e-06 -2.482531e-04  2.527518e-04                -2.999117e-06 -1.655021e-04  1.685012e-04

Alignment Tensor Irreducible Representation (A0, A1R, A1I, A2R, A2I):
  2.626246e-04  9.506274e-05 -1.310603e-04 -1.524155e-04 -1.457275e-04

Tensor Properties: *)
A(axial)      =   2.527518e-04        # alignment tensor axial component   = 3/2*A(zz) = S(zz)
A(rhombic)    =   1.625030e-04        # alignment tensor rhombic component = A(xx) - A(yy) = 2/3*(S(xx) - S(yy))
A(rhombicity) =   6.429349e-01        # alignment tensor rhombicity        = A(rhombic) / A(axial)
A(asymmetry)  =   9.644024e-01        # alignment tensor asymmetry         = 3/2*A(rhombicity) = (A(xx) - A(yy))/A(zz) = (S(xx) - S(yy))/S(zz)
GDO           =   2.892903e-04        # generalized degree of order        = sqrt(3/2)*|A(xx),A(yy),A(zz)| = sqrt(2/3)*|S(xx),S(yy),S(zz)|
----------
*) F. Kramer, M.V. Deshmukh, H. Kessler and S.J. Glaser, Concepts Magn. Res. A, 2004, 21A, 21-40.
```

```
--------------------------------------------------------------------------------
Results for Multi-Parameter SVD Fit of Calculated and Experimental Data:
           D(calc)[HZ]     +/- Error    D(exp)[HZ]     +/- Error  Rel. Weights            D(exp)-D(calc)        Normalized Weights    Atom Labels
D[01] =      -0.688482            -     -0.050000      0.500000      1.000000    r[01] =       0.638482    w[01] =       0.090909    C1-H1
D[02] =       0.312259            -      0.300000      0.500000      1.000000    r[02] =      -0.012259    w[02] =       0.090909    C2-H2
D[03] =      -4.356000            -     -3.750000      0.500000      1.000000    r[03] =       0.606000    w[03] =       0.090909    C3-H3
D[04] =      -4.425590            -     -4.500000      0.500000      1.000000    r[04] =      -0.074410    w[04] =       0.090909    C4-H4s
D[05] =       3.174392            -      3.050000      0.500000      1.000000    r[05] =      -0.124392    w[05] =       0.090909    C4-H4a
D[06] =       0.421299            -      1.000000      0.500000      1.000000    r[06] =       0.578701    w[06] =       0.090909    C5-H5
D[07] =      -2.085281            -     -1.850000      0.500000      1.000000    r[07] =       0.235281    w[07] =       0.090909    C7-H7s
D[08] =       4.996359            -      5.700000      0.500000      1.000000    r[08] =       0.703641    w[08] =       0.090909    C7-H7a
D[09] =       0.218900            -     -0.070000      0.500000      1.000000    r[09] =      -0.288900    w[09] =       0.090909    C6-C8
D[10] =      -0.413249            -     -0.270000      0.500000      1.000000    r[10] =       0.143249    w[10] =       0.090909    C6-C9
D[11] =      -0.036619            -     -0.100000      0.500000      1.000000    r[11] =      -0.063381    w[11] =       0.090909    C10-C2

Results for Multi-Parameter Fit of Calculated and Experimental Data:
rank  =              5                 # rank of cosine matrix (check input if rank < 5)
cond  =   2.128683e+00                 # condition number of cosine matrix (check input and singular values if very large)
aic   =      17.157673                 # information criterion (AIC) for 5 degrees of freedom
qfac  =       0.148901                 # weighted Q-Factor as defined by Cornilescu
r^2   =       0.977821                 # coefficient of determination r^2 = 1 - chi^2 / (weighted sum of squares)

(|D|) =       1.920766      1.876364   # mean absolute (calc./exp.) parameter D[i]
|D|min=       0.036619      0.050000   # min. absolute (calc./exp.) parameter D[i]
|D|max=       4.996359      5.700000   # max. absolute (calc./exp.) parameter D[i]
Drange=      -4.425590      4.996359   # min. and max. (calc.)      parameter D[i]
Drange=      -4.500000      5.700000   # min. and max. (exp.)       parameter D[i]

Results for Linear Regression of Calculated and Experimental Data (N=11):
c(b)  =      -0.214069 +/-  0.112197   # linear regression intercept and error
c(m)  =       0.976396 +/-  0.041421   # linear regression slope and error
rmsd  =       0.403329                 # unweighted total root-mean-square deviation
chisq =       0.162674                 # weighted total sum of squared residuals
maerr =       0.315336                 # weighted total mean absolute error (sum of weights = 1.000)
R     =       0.991999                 # weighted Pearson correlation coefficient R
R^2   =       0.984061                 # weighted Pearson correlation coefficient R^2

E(RDC)=       0.894709                 # E(RDC)=1/2*K*(sum of weighted deviations (D(exp)-D(calc))^2), K=1.000

--------------------------------------------------------------------------------
```

3.1.17 (-)-IPC in Stick S168

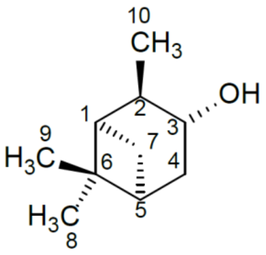

(-)-IPC

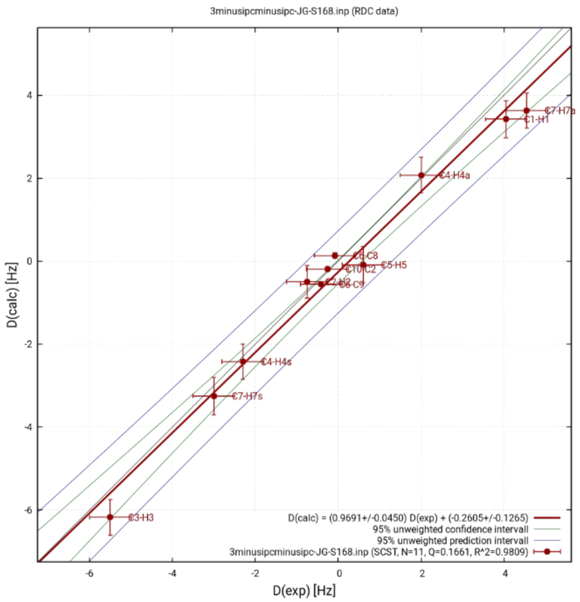


```
================================================================================
info : Start Analysis for Structure '#OutputFileName = ipc_kw188-1'
in file '3minusipcminusipc-JG-S168.inp'
================================================================================

--------------------------------------------------------------------------------
info : Start Single-Conformer Single-Tensor (SCST) Fit with 11 RDCs
info : File: '3minusipcminusipc-JG-S168.inp', Title: '#OutputFileName = ipc_kw188-1'
--------------------------------------------------------------------------------

--------------------------------------------------------------------------------
SVD Best-Fit Saupe Vector S(zz), S(xx-yy), S(xy), S(xz), S(yz):
  1.200498e-07 -3.052500e-04 -1.273335e-04  1.434872e-04 -1.236707e-04
Saupe Tensor (S):                                        Alignment Tensor (A):
 -1.526850e-04 -1.273335e-04  1.434872e-04                -1.017900e-04 -8.488900e-05  9.565814e-05
 -1.273335e-04  1.525650e-04 -1.236707e-04                -8.488900e-05  1.017100e-04 -8.244711e-05
  1.434872e-04 -1.236707e-04  1.200498e-07                 9.565814e-05 -8.244711e-05  8.003323e-08
Trace of Saupe Tensor:        2.032879e-20               Trace of Alignment Tensor:    1.355253e-20
Eigenvectors of Saupe Tensor (S):                        Eigenvectors of Alignment Tensor (A):
  2.692889e-01 -8.844277e-01  3.811446e-01                 2.692889e-01 -8.844277e-01  3.811446e-01
  6.175658e-01 -1.450978e-01 -7.730194e-01                 6.175658e-01 -1.450978e-01 -7.730194e-01
  7.389830e-01  4.435474e-01  5.071191e-01                 7.389830e-01  4.435474e-01  5.071191e-01
Eigenvalues of Saupe Tensor S(xx), S(yy), S(zz):         Eigenvalues of Alignment Tensor A(xx), A(yy), A(zz):
 -5.094372e-05 -2.455351e-04  2.964789e-04                -3.396248e-05 -1.636901e-04  1.976526e-04

Alignment Tensor Irreducible Representation (A0, A1R, A1I, A2R, A2I):
  1.903187e-07  1.857323e-04 -1.600814e-04 -1.975604e-04 -1.648227e-04

Tensor Properties: *)
A(axial)      =   2.964789e-04         # alignment tensor axial component   = 3/2*A(zz) = S(zz)
A(rhombic)    =   1.297276e-04         # alignment tensor rhombic component = A(xx) - A(yy) = 2/3*(S(xx) - S(yy))
A(rhombicity) =   4.375611e-01         # alignment tensor rhombicity        = A(rhombic) / A(axial)
A(asymmetry)  =   6.563416e-01         # alignment tensor asymmetry         = 3/2*A(rhombicity) = (A(xx) - A(yy))/A(zz) = (S(xx) - S(yy))/S(zz)
GDO           =   3.170515e-04         # generalized degree of order        = sqrt(3/2)*|A(xx),A(yy),A(zz)| = sqrt(2/3)*|S(xx),S(yy),S(zz)|
----------
*) F. Kramer, M.V. Deshmukh, H. Kessler and S.J. Glaser, Concepts Magn. Res. A, 2004, 21A, 21-40.
```

```
--------------------------------------------------------------------------------
Results for Multi-Parameter SVD Fit of Calculated and Experimental Data:
           D(calc)[HZ]     +/- Error    D(exp)[HZ]     +/- Error  Rel. Weights            D(exp)-D(calc)        Normalized Weights    Atom Labels
D[01] =       3.419042             -      4.050000      0.500000      1.000000    r[01] =       0.630958    w[01] =       0.090909    C1-H1
D[02] =      -0.498451             -     -0.750000      0.500000      1.000000    r[02] =      -0.251549    w[02] =       0.090909    C2-H2
D[03] =      -6.177864             -     -5.500000      0.500000      1.000000    r[03] =       0.677864    w[03] =       0.090909    C3-H3
D[04] =      -2.423684             -     -2.300000      0.500000      1.000000    r[04] =       0.123684    w[04] =       0.090909    C4-H4s
D[05] =       2.074395             -      2.000000      0.500000      1.000000    r[05] =      -0.074395    w[05] =       0.090909    C4-H4a
D[06] =      -0.088458             -      0.600000      0.500000      1.000000    r[06] =       0.688458    w[06] =       0.090909    C5-H5
D[07] =      -3.250830             -     -3.000000      0.500000      1.000000    r[07] =       0.250830    w[07] =       0.090909    C7-H7s
D[08] =       3.631597             -      4.550000      0.500000      1.000000    r[08] =       0.918403    w[08] =       0.090909    C7-H7a
D[09] =       0.135073             -     -0.080000      0.500000      1.000000    r[09] =      -0.215073    w[09] =       0.090909    C6-C8
D[10] =      -0.559471             -     -0.410000      0.500000      1.000000    r[10] =       0.149471    w[10] =       0.090909    C6-C9
D[11] =      -0.193205             -     -0.260000      0.500000      1.000000    r[11] =      -0.066795    w[11] =       0.090909    C10-C2

Results for Multi-Parameter Fit of Calculated and Experimental Data:
rank  =              5                # rank of cosine matrix (check input if rank < 5)
cond  =   2.128683e+00                # condition number of cosine matrix (check input and singular values if very large)
aic   =      19.580525                # information criterion (AIC) for 5 degrees of freedom
qfac  =       0.166104                # weighted Q-Factor as defined by Cornilescu
r^2   =       0.972374                # coefficient of determination r^2 = 1 - chi^2 / (weighted sum of squares)

(|D|) =       2.041097      2.136364   # mean absolute (calc./exp.) parameter D[i]
|D|min=       0.088458      0.080000   # min. absolute (calc./exp.) parameter D[i]
|D|max=       6.177864      5.500000   # max. absolute (calc./exp.) parameter D[i]
Drange=      -6.177864      3.631597   # min. and max. (calc.)      parameter D[i]
Drange=      -5.500000      4.550000   # min. and max. (exp.)       parameter D[i]

Results for Linear Regression of Calculated and Experimental Data (N=11):
c(b)  =      -0.260531 +/-  0.126546   # linear regression intercept and error
c(m)  =       0.969108 +/-  0.045046   # linear regression slope and error
rmsd  =       0.466625                # unweighted total root-mean-square deviation
chisq =       0.217739                # weighted total sum of squared residuals
maerr =       0.367953                # weighted total mean absolute error (sum of weights = 1.000)
R     =       0.990417                # weighted Pearson correlation coefficient R
R^2   =       0.980926                # weighted Pearson correlation coefficient R^2

E(RDC)=       1.197566                # E(RDC)=1/2*K*(sum of weighted deviations (D(exp)-D(calc))^2), K=1.000
```

3.1.18 (-)-Strychnine in Stick S173

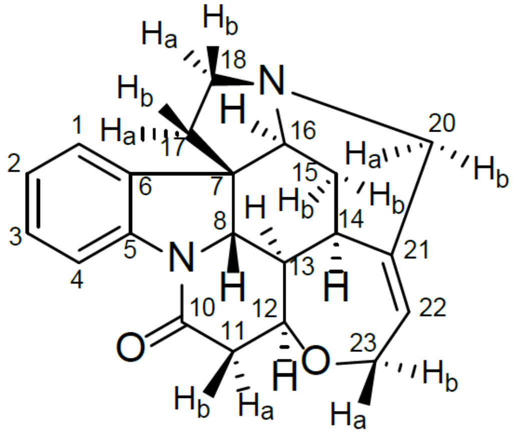

(-)-Strychnine

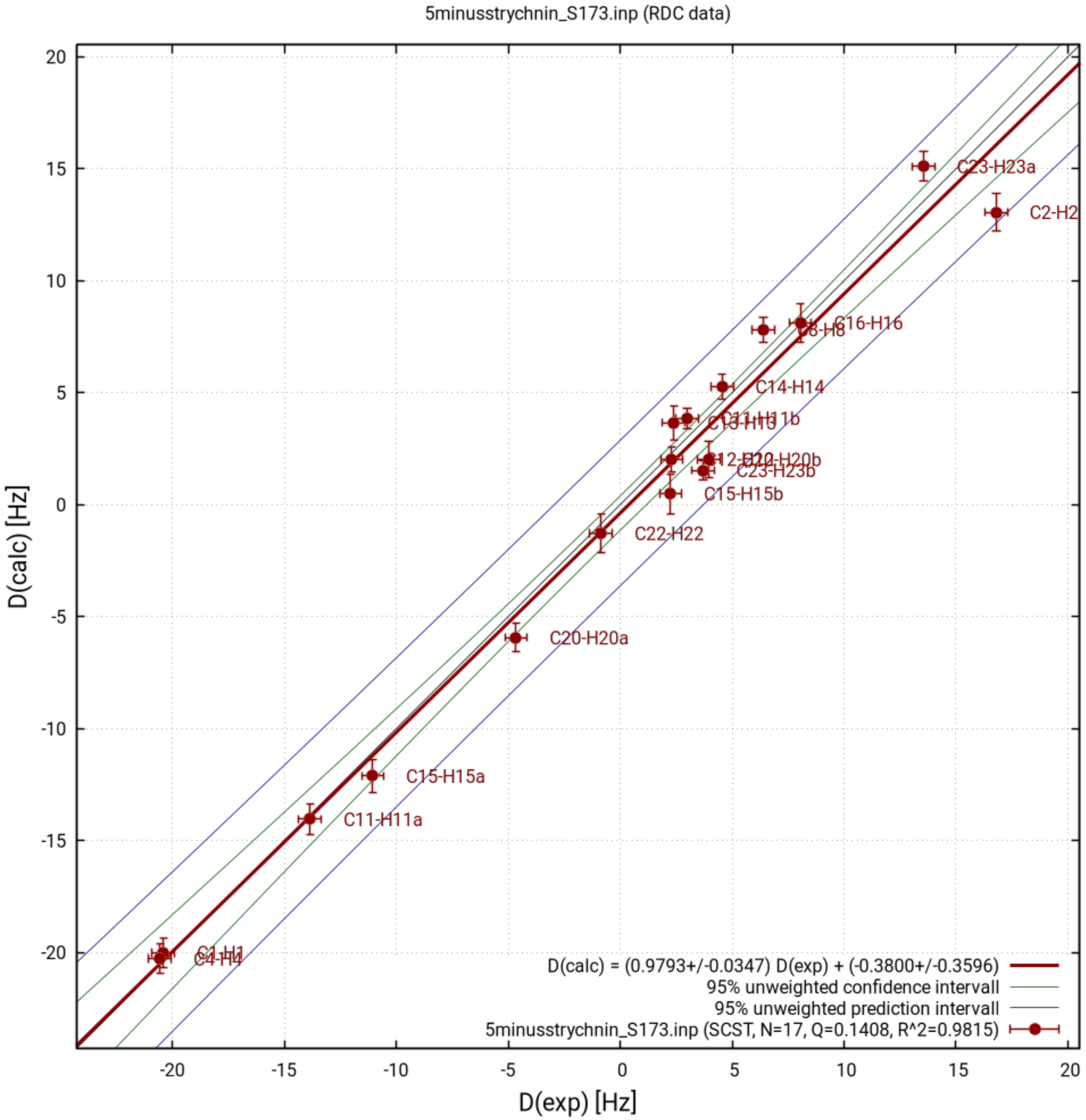

```
================================================================================
info : Start Analysis for Structure '#AK-XXX' in file '5minusstrychnin_S173.inp'
================================================================================

--------------------------------------------------------------------------------
info : Start Single-Conformer Single-Tensor (SCST) Fit with 17 RDCs
info : File: '5minusstrychnin_S173.inp', Title: '#AK-XXX'
--------------------------------------------------------------------------------

--------------------------------------------------------------------------------
SVD Best-Fit Saupe Vector S(zz), S(xx-yy), S(xy), S(xz), S(yz):
 -1.678084e-04 -1.212187e-04 -6.219590e-04  5.456429e-04 -6.189187e-05
Saupe Tensor (S):                                       Alignment Tensor (A):
  2.329485e-05 -6.219590e-04  5.456429e-04                1.552990e-05 -4.146393e-04  3.637619e-04
 -6.219590e-04  1.445136e-04 -6.189187e-05               -4.146393e-04  9.634238e-05 -4.126124e-05
  5.456429e-04 -6.189187e-05 -1.678084e-04                3.637619e-04 -4.126124e-05 -1.118723e-04
Trace of Saupe Tensor:       -2.710505e-20              Trace of Alignment Tensor:   -1.807004e-20
Eigenvectors of Saupe Tensor (S):                       Eigenvectors of Alignment Tensor (A):
  1.791972e-01  7.029853e-01 -6.882587e-01                1.791972e-01  7.029853e-01 -6.882587e-01
  6.670012e-01  4.274407e-01  6.102490e-01                6.670012e-01  4.274407e-01  6.102490e-01
  7.231859e-01 -5.684243e-01 -3.922959e-01                7.231859e-01 -5.684243e-01 -3.922959e-01
Eigenvalues of Saupe Tensor S(xx), S(yy), S(zz):        Eigenvalues of Alignment Tensor A(xx), A(yy), A(zz):
 -8.968782e-05 -7.960782e-04  8.857660e-04               -5.979188e-05 -5.307188e-04  5.905107e-04

Alignment Tensor Irreducible Representation (A0, A1R, A1I, A2R, A2I):
 -2.660319e-04  7.062896e-04 -8.011390e-05 -7.845380e-05 -8.050744e-04

Tensor Properties: *)
A(axial)      =   8.857660e-04         # alignment tensor axial component   = 3/2*A(zz) = S(zz)
A(rhombic)    =   4.709269e-04         # alignment tensor rhombic component = A(xx) - A(yy) = 2/3*(S(xx) - S(yy))
A(rhombicity) =   5.316606e-01         # alignment tensor rhombicity        = A(rhombic) / A(axial)
A(asymmetry)  =   7.974909e-01         # alignment tensor asymmetry         = 3/2*A(rhombicity) = (A(xx) - A(yy))/A(zz) = (S(xx) - S(yy))/S(zz)
GDO           =   9.751464e-04         # generalized degree of order        = sqrt(3/2)*|A(xx),A(yy),A(zz)| = sqrt(2/3)*|S(xx),S(yy),S(zz)|
----------
*) F. Kramer, M.V. Deshmukh, H. Kessler and S.J. Glaser, Concepts Magn. Res. A, 2004, 21A, 21-40.
```

```
--------------------------------------------------------------------------------
Results for Multi-Parameter SVD Fit of Calculated and Experimental Data:
           D(calc)[HZ]     +/- Error    D(exp)[HZ]     +/- Error  Rel. Weights            D(exp)-D(calc)        Normalized Weights    Atom Labels
D[01] =      -1.285877             -     -0.850000      0.500000      1.000000    r[01] =       0.435877    w[01] =       0.058824    C22-H22
D[02] =      13.058214             -     16.800000      0.500000      1.000000    r[02] =       3.741786    w[02] =       0.058824    C2-H2
D[03] =     -20.009340             -    -20.400000      0.500000      1.000000    r[03] =      -0.390660    w[03] =       0.058824    C1-H1
D[04] =     -20.282282             -    -20.550000      0.500000      1.000000    r[04] =      -0.267718    w[04] =       0.058824    C4-H4
D[05] =       2.024406             -      2.300000      0.500000      1.000000    r[05] =       0.275594    w[05] =       0.058824    C12-H12
D[06] =      15.099347             -     13.550000      0.500000      1.000000    r[06] =      -1.549347    w[06] =       0.058824    C23-H23a
D[07] =       1.530947             -      3.700000      0.500000      1.000000    r[07] =       2.169053    w[07] =       0.058824    C23-H23b
D[08] =       8.124061             -      8.050000      0.500000      1.000000    r[08] =      -0.074061    w[08] =       0.058824    C16-H16
D[09] =       7.814103             -      6.400000      0.500000      1.000000    r[09] =      -1.414103    w[09] =       0.058824    C8-H8
D[10] =      -5.936758             -     -4.650000      0.500000      1.000000    r[10] =       1.286758    w[10] =       0.058824    C20-H20a
D[11] =       2.009885             -      3.950000      0.500000      1.000000    r[11] =       1.940115    w[11] =       0.058824    C20-H20b
D[12] =       3.653253             -      2.400000      0.500000      1.000000    r[12] =      -1.253253    w[12] =       0.058824    C13-H13
D[13] =     -14.052923             -    -13.850000      0.500000      1.000000    r[13] =       0.202923    w[13] =       0.058824    C11-H11a
D[14] =       3.851501             -      3.000000      0.500000      1.000000    r[14] =      -0.851501    w[14] =       0.058824    C11-H11b
D[15] =       5.265289             -      4.550000      0.500000      1.000000    r[15] =      -0.715289    w[15] =       0.058824    C14-H14
D[16] =     -12.124623             -    -11.050000      0.500000      1.000000    r[16] =       1.074623    w[16] =       0.058824    C15-H15a
D[17] =       0.491923             -      2.250000      0.500000      1.000000    r[17] =       1.758077    w[17] =       0.058824    C15-H15b

Results for Multi-Parameter Fit of Calculated and Experimental Data:
rank  =              5                # rank of cosine matrix (check input if rank < 5)
cond  =   3.907499e+00                # condition number of cosine matrix (check input and singular values if very large)
aic   =     154.462331                # information criterion (AIC) for 5 degrees of freedom
qfac  =       0.140812                # weighted Q-Factor as defined by Cornilescu
r^2   =       0.980160                # coefficient of determination r^2 = 1 - chi^2 / (weighted sum of squares)

(|D|) =       8.036161      8.135294   # mean absolute (calc./exp.) parameter D[i]
|D|min=       0.491923      0.850000   # min. absolute (calc./exp.) parameter D[i]
|D|max=      20.282282     20.550000   # max. absolute (calc./exp.) parameter D[i]
Drange=     -20.282282     15.099347   # min. and max. (calc.)      parameter D[i]
Drange=     -20.550000     16.800000   # min. and max. (exp.)       parameter D[i]

Results for Linear Regression of Calculated and Experimental Data (N=17):
c(b)  =      -0.380009 +/-  0.359557   # linear regression intercept and error
c(m)  =       0.979254 +/-  0.034736   # linear regression slope and error
rmsd  =       1.457548                # unweighted total root-mean-square deviation
chisq =       2.124446                # weighted total sum of squared residuals
maerr =       1.141220                # weighted total mean absolute error (sum of weights = 1.000)
R     =       0.990694                # weighted Pearson correlation coefficient R
R^2   =       0.981475                # weighted Pearson correlation coefficient R^2

E(RDC)=      18.057791                # E(RDC)=1/2*K*(sum of weighted deviations (D(exp)-D(calc))^2), K=1.000
```

3.1.19 (-)-Sparteine in Stick S176

in *ent*-p1; Multi Conformer Single Tensor Fit

```
================================================================================
info : Start Analysis for Structure '#ak-sparteine-1-b3lyp-6311Gdp-1'
 in file '3minusspartein_JG-S176.inp'
================================================================================
--------------------------------------------------------------------------------
info : Start Scan of Populations for 2/2 Conformers (nsteps = 20)
--------------------------------------------------------------------------------
info : generated and analyzed 21 populations of 2/2 conformers.
info : continue with best-fit populations.
--------------------------------------------------------------------------------
info : Start Gradient-Descent Optimization of Populations for 2/2 Conformers
--------------------------------------------------------------------------------
info : relative weight of used conformers: w(1)=0.7000 w(2)=0.3000 | iteration   1 |
q = 1.042694e-01 e = 1.922951e+00 de =  1.92295e+00 step = 5.000000e-03
info : conformer populations converged after 1/1000 steps (dxmax = 0.000e+00,
q = 1.043e-01, dq = 0.000e+00, e = 1.923e+00, de = 0.000e+00).
info : continue with optimized best-fit populations.
--------------------------------------------------------------------------------
--------------------------------------------------------------------------------
info : Start Multi-Conformer Single-Tensor (MCST) Fit with 2/2 Conformers and 12 RDCs
info : File: '3minusspartein_JG-S176.inp', Title: '#ak-sparteine-1-b3lyp-6311Gdp-1'
--------------------------------------------------------------------------------
info : relative weight of conformers: w(1) = 0.7000; w(2) = 0.3000

--------------------------------------------------------------------------------
Structure File: '3minusspartein_JG-S176.inp' (Title: '#ak-sparteine-1-b3lyp-6311Gdp-1')
--------------------------------------------------------------------------------
Structure with 2 Conformer(s) and 12 RDCs: File '3minusspartein_JG-S176.inp'
            70.00 % :            Conformer 1 with 43 Atoms:  Title 'Sparteine (Conformer 1)'
            30.00 % :            Conformer 2 with 43 Atoms:  Title 'Sparteine (Conformer 2)'
--------------------------------------------------------------------------------

--------------------------------------------------------------------------------
SVD Best-Fit Saupe Vector S(zz), S(xx-yy), S(xy), S(xz), S(yz):
 -3.110639e-05  5.853095e-04  2.289392e-04 -2.956641e-04 -1.190672e-04
Saupe Tensor (S):                                       Alignment Tensor (A):
  3.082080e-04  2.289392e-04 -2.956641e-04                2.054720e-04  1.526261e-04 -1.971094e-04
  2.289392e-04 -2.771016e-04 -1.190672e-04                1.526261e-04 -1.847344e-04 -7.937814e-05
 -2.956641e-04 -1.190672e-04 -3.110639e-05               -1.971094e-04 -7.937814e-05 -2.073759e-05
Trace of Saupe Tensor:          2.710505e-20            Trace of Alignment Tensor:    1.807004e-20
Eigenvectors of Saupe Tensor (S):                       Eigenvectors of Alignment Tensor (A):
  4.777747e-01 -2.901348e-01 -8.291882e-01                4.777747e-01 -2.901348e-01 -8.291882e-01
  6.737710e-02  9.532092e-01 -2.947077e-01                6.737710e-02  9.532092e-01 -2.947077e-01
  8.758948e-01  8.493556e-02  4.749677e-01                8.758948e-01  8.493556e-02  4.749677e-01
Eigenvalues of Saupe Tensor S(xx), S(yy), S(zz):        Eigenvalues of Alignment Tensor A(xx), A(yy), A(zz):
 -2.015415e-04 -3.573949e-04  5.589364e-04               -1.343610e-04 -2.382632e-04  3.726242e-04
Alignment Tensor Irreducible Representation (A0, A1R, A1I, A2R, A2I):
 -4.931392e-05 -3.827127e-04 -1.541226e-04  3.788174e-04  2.963429e-04
```

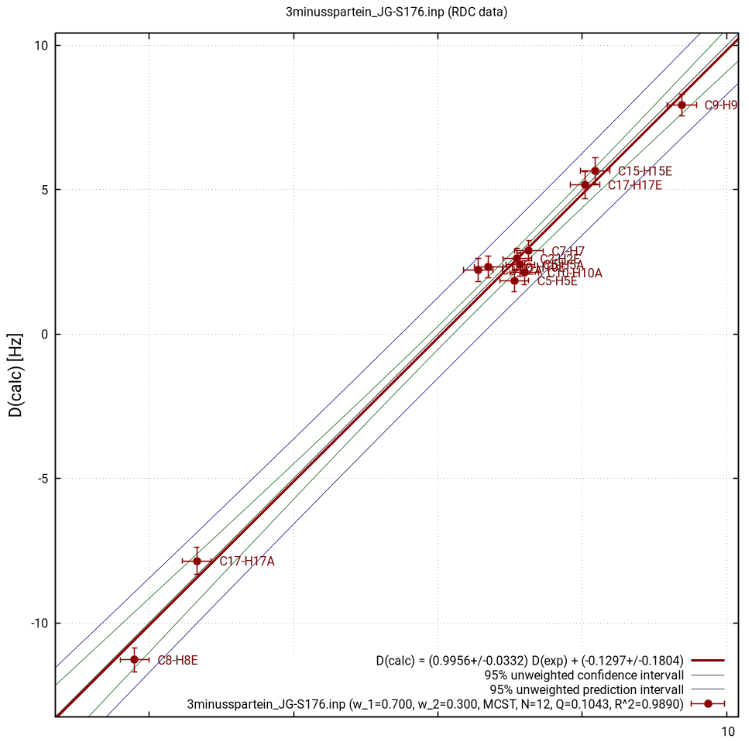

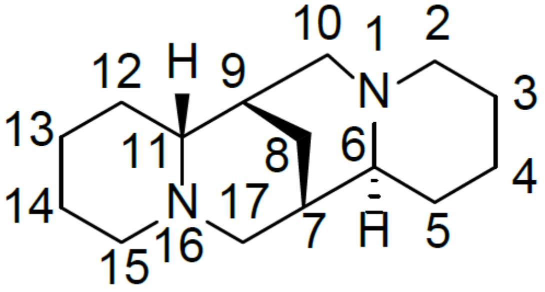

(-)-Sparteine

```
Tensor Properties: *)
A(axial)       =   5.589364e-04         # alignment tensor axial component   = 3/2*A(zz) = S(zz)
A(rhombic)     =   1.039022e-04         # alignment tensor rhombic component = A(xx) - A(yy) = 2/3*(S(xx) - S(yy))
A(rhombicity) =   1.858928e-01         # alignment tensor rhombicity        = A(rhombic) / A(axial)
A(asymmetry)  =   2.788392e-01         # alignment tensor asymmetry         = 3/2*A(rhombicity) = (A(xx) - A(yy))/A(zz) = (S(xx) - S(yy))/S(zz)
GDO            =   5.661330e-04         # generalized degree of order        = sqrt(3/2)*|A(xx),A(yy),A(zz)| = sqrt(2/3)*|S(xx),S(yy),S(zz)|
----------
*) F. Kramer, M.V. Deshmukh, H. Kessler and S.J. Glaser, Concepts Magn. Res. A, 2004, 21A, 21-40.

--------------------------------------------------------------------------------
Results for Multi-Parameter SVD Fit of Calculated and Experimental Data:
           D(calc)[HZ]     +/- Error    D(exp)[HZ]     +/- Error  Rel. Weights            D(exp)-D(calc)        Normalized Weights | RDC Conformers  |    Atom Labels
D[01] =       2.324153           -       1.750000      0.500000      1.000000    r[01] =      -0.574153    w[01] =       0.083333 |   2.186   2.647 |    C10-H10E
D[02] =       2.129967           -       3.000000      0.500000      1.000000    r[02] =       0.870033    w[02] =       0.083333 |   1.967   2.509 |    C10-H10A
D[03] =       2.612443           -       2.750000      0.500000      1.000000    r[03] =       0.137557    w[03] =       0.083333 |   2.263   3.429 |    C2-H2E
D[04] =       2.221593           -       1.400000      0.500000      1.000000    r[04] =      -0.821593    w[04] =       0.083333 |   2.246   2.166 |    C2-H2A
D[05] =       5.652203           -       5.450000      0.500000      1.000000    r[05] =      -0.202203    w[05] =       0.083333 |   7.768   0.716 |    C15-H15E
D[06] =       5.170772           -       5.100000      0.500000      1.000000    r[06] =      -0.070772    w[06] =       0.083333 |   8.080  -1.617 |    C17-H17E
D[07] =      -7.850659           -      -8.350000      0.500000      1.000000    r[07] =      -0.499341    w[07] =       0.083333 | -10.327  -2.072 |    C17-H17A
D[08] =       7.939648           -       8.450000      0.500000      1.000000    r[08] =       0.510352    w[08] =       0.083333 |   8.021   7.750 |    C9-H9
D[09] =       2.882461           -       3.150000      0.500000      1.000000    r[09] =       0.267539    w[09] =       0.083333 |   2.785   3.110 |    C7-H7
D[10] =       2.411607           -       2.850000      0.500000      1.000000    r[10] =       0.438393    w[10] =       0.083333 |   2.343   2.571 |    C5-H5A
D[11] =       1.853401           -       2.650000      0.500000      1.000000    r[11] =       0.796599    w[11] =       0.083333 |   1.466   2.757 |    C5-H5E
D[12] =     -11.281873           -     -10.500000      0.500000      1.000000    r[12] =       0.781873    w[12] =       0.083333 | -10.918 -12.130 |    C8-H8E

Results for Multi-Parameter Fit of Calculated and Experimental Data:
rank  =              5                  # rank of cosine matrix (check input if rank < 5)
cond  =   7.267329e+00                  # condition number of cosine matrix (check input and singular values if very large)
aic   =      27.383605                  # information criterion (AIC) for 6 degrees of freedom
qfac  =       0.104269                  # weighted Q-Factor as defined by Cornilescu
r^2   =       0.988262                  # coefficient of determination r^2 = 1 - chi^2 / (weighted sum of squares)

(|D|) =       4.527565      4.616667   # mean absolute (calc./exp.) parameter D[i]
|D|min=       1.853401      1.400000   # min. absolute (calc./exp.) parameter D[i]
|D|max=      11.281873     10.500000   # max. absolute (calc./exp.) parameter D[i]
Drange=     -11.281873      7.939648   # min. and max. (calc.)      parameter D[i]
Drange=     -10.500000      8.450000   # min. and max. (exp.)       parameter D[i]

Results for Linear Regression of Calculated and Experimental Data (N=12):
c(b)  =      -0.129729 +/-  0.180399   # linear regression intercept and error
c(m)  =       0.995619 +/-  0.033226   # linear regression slope and error
rmsd  =       0.566120                  # unweighted total root-mean-square deviation
chisq =       0.320492                  # weighted total sum of squared residuals
maerr =       0.497534                  # weighted total mean absolute error (sum of weights = 1.000)
R     =       0.994477                  # weighted Pearson correlation coefficient R
R^2   =       0.988985                  # weighted Pearson correlation coefficient R^2

E(RDC)=       1.922951                  # E(RDC)=1/2*K*(sum of weighted deviations (D(exp)-D(calc))^2), K=1.000
```

3.1.20 (+)-IPC in Stick S179 – 300 K out of T-series

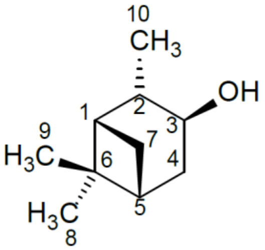

(+)-IPC

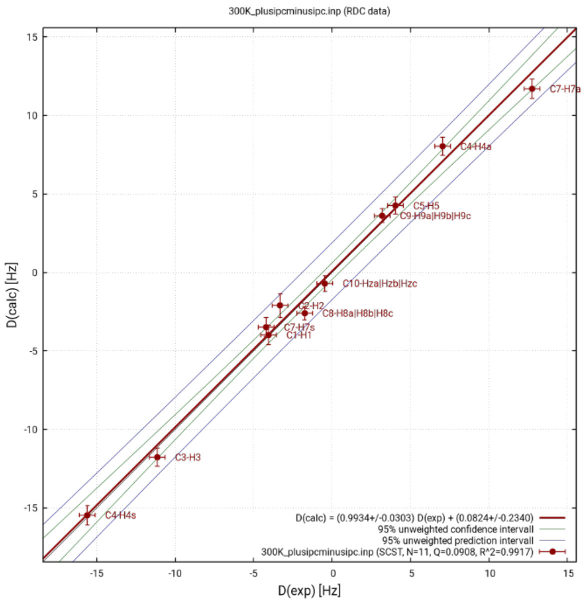


```
================================================================================
info : Start Analysis for Structure '' in file '300K_plusipcminusipc.inp'
================================================================================

--------------------------------------------------------------------------------
info : Start Single-Conformer Single-Tensor (SCST) Fit with 11 RDCs
info : File: '300K_plusipcminusipc.inp', Title: ''
--------------------------------------------------------------------------------

--------------------------------------------------------------------------------
SVD Best-Fit Saupe Vector S(zz), S(xx-yy), S(xy), S(xz), S(yz):
  5.859511e-04 -3.634627e-04 -2.696331e-04  2.350816e-04 -2.963410e-04
Saupe Tensor (S):                                        Alignment Tensor (A):
 -4.747069e-04 -2.696331e-04  2.350816e-04                -3.164713e-04 -1.797554e-04  1.567211e-04
 -2.696331e-04 -1.112442e-04 -2.963410e-04                -1.797554e-04 -7.416279e-05 -1.975607e-04
  2.350816e-04 -2.963410e-04  5.859511e-04                 1.567211e-04 -1.975607e-04  3.906341e-04
Trace of Saupe Tensor:         0.000000e+00              Trace of Alignment Tensor:     0.000000e+00
Eigenvectors of Saupe Tensor (S):                        Eigenvectors of Alignment Tensor (A):
 -3.620783e-01 -8.983621e-01  2.486861e-01                -3.620783e-01 -8.983621e-01  2.486861e-01
  8.195824e-01 -4.339073e-01 -3.741780e-01                 8.195824e-01 -4.339073e-01 -3.741780e-01
  4.440541e-01  6.833701e-02  8.933902e-01                 4.440541e-01  6.833701e-02  8.933902e-01
Eigenvalues of Saupe Tensor S(xx), S(yy), S(zz):         Eigenvalues of Alignment Tensor A(xx), A(yy), A(zz):
 -1.526837e-04 -6.228215e-04  7.755053e-04                -1.017892e-04 -4.152143e-04  5.170035e-04

Alignment Tensor Irreducible Representation (A0, A1R, A1I, A2R, A2I):
  9.289264e-04  3.042937e-04 -3.835889e-04 -2.352362e-04 -3.490178e-04

Tensor Properties: *)
A(axial)      =   7.755053e-04         # alignment tensor axial component   = 3/2*A(zz) = S(zz)
A(rhombic)    =   3.134252e-04         # alignment tensor rhombic component = A(xx) - A(yy) = 2/3*(S(xx) - S(yy))
A(rhombicity) =   4.041561e-01         # alignment tensor rhombicity        = A(rhombic) / A(axial)
A(asymmetry)  =   6.062342e-01         # alignment tensor asymmetry         = 3/2*A(rhombicity) = (A(xx) - A(yy))/A(zz) = (S(xx) - S(yy))/S(zz)
GDO           =   8.216355e-04         # generalized degree of order        = sqrt(3/2)*|A(xx),A(yy),A(zz)| = sqrt(2/3)*|S(xx),S(yy),S(zz)|
----------
*) F. Kramer, M.V. Deshmukh, H. Kessler and S.J. Glaser, Concepts Magn. Res. A, 2004, 21A, 21-40.
```

```
--------------------------------------------------------------------------------
Results for Multi-Parameter SVD Fit of Calculated and Experimental Data:
           D(calc)[HZ]     +/- Error    D(exp)[HZ]     +/- Error  Rel. Weights           D(exp)-D(calc)       Normalized Weights    Atom Labels
D[01] =      -3.977604             -     -4.050000      0.500000      1.000000    r[01] =      -0.072396    w[01] =       0.090909    C1-H1
D[02] =      -2.109185             -     -3.300000      0.500000      1.000000    r[02] =      -1.190815    w[02] =       0.090909    C2-H2
D[03] =     -11.766004             -    -11.150000      0.500000      1.000000    r[03] =       0.616004    w[03] =       0.090909    C3-H3
D[04] =     -15.451299             -    -15.600000      0.500000      1.000000    r[04] =      -0.148701    w[04] =       0.090909    C4-H4s
D[05] =       8.034539             -      7.050000      0.500000      1.000000    r[05] =      -0.984539    w[05] =       0.090909    C4-H4a
D[06] =       4.257230             -      4.050000      0.500000      1.000000    r[06] =      -0.207230    w[06] =       0.090909    C5-H5
D[07] =      -3.478668             -     -4.200000      0.500000      1.000000    r[07] =      -0.721332    w[07] =       0.090909    C7-H7s
D[08] =      11.699377             -     12.750000      0.500000      1.000000    r[08] =       1.050623    w[08] =       0.090909    C7-H7a
D[09] =      -2.590501             -     -1.750000      0.500000      1.000000    r[09] =       0.840501    w[09] =       0.090909    C8-H8a|H8b|H8c
              6.970829             -             -             -             -                         -                          -    | [3av] C8-H8a
             -1.326258             -             -             -             -                         -                          -    | [3av] C8-H8b
            -13.416075             -             -             -             -                         -                          -    | [3av] C8-H8c
D[10] =       3.618283             -      3.200000      0.500000      1.000000    r[10] =      -0.418283    w[10] =       0.090909    C9-H9a|H9b|H9c
             -0.808350             -             -             -             -                         -                          -    | [3av] C9-H9a
              7.038781             -             -             -             -                         -                          -    | [3av] C9-H9b
              4.624418             -             -             -             -                         -                          -    | [3av] C9-H9c
D[11] =      -0.691417             -     -0.450000      0.500000      1.000000    r[11] =       0.241417    w[11] =       0.090909    C10-Hza|Hzb|Hzc
             -8.849511             -             -             -             -                         -                          -    | [3av] C10-Hza
             -1.479869             -             -             -             -                         -                          -    | [3av] C10-Hzb
              8.255130             -             -             -             -                         -                          -    | [3av] C10-Hzc

Results for Multi-Parameter Fit of Calculated and Experimental Data:
rank  =              5                # rank of cosine matrix (check input if rank < 5)
cond  =   2.147425e+00                # condition number of cosine matrix (check input and singular values if very large)
aic   =      31.603721                # information criterion (AIC) for 5 degrees of freedom
qfac  =       0.090821                # weighted Q-Factor as defined by Cornilescu
r^2   =       0.991539                # coefficient of determination r^2 = 1 - chi^2 / (weighted sum of squares)

(|D|) =       6.152191      6.140909   # mean absolute (calc./exp.) parameter D[i]
|D|min=       0.691417      0.450000   # min. absolute (calc./exp.) parameter D[i]
|D|max=      15.451299     15.600000   # max. absolute (calc./exp.) parameter D[i]
Drange=     -15.451299     11.699377   # min. and max. (calc.)      parameter D[i]
Drange=     -15.600000     12.750000   # min. and max. (exp.)       parameter D[i]

Results for Linear Regression of Calculated and Experimental Data (N=11):
c(b)  =       0.082416 +/-  0.233974   # linear regression intercept and error
c(m)  =       0.993445 +/-  0.030326   # linear regression slope and error
rmsd  =       0.700709                 # unweighted total root-mean-square deviation
chisq =       0.490994                 # weighted total sum of squared residuals
maerr =       0.590167                 # weighted total mean absolute error (sum of weights = 1.000)
R     =       0.995833                 # weighted Pearson correlation coefficient R
R^2   =       0.991683                 # weighted Pearson correlation coefficient R^2

E(RDC)=       2.700465                 # E(RDC)=1/2*K*(sum of weighted deviations (D(exp)-D(calc))^2), K=1.000
```

3.1.21 (-)-Sparteine in S180

Multi Conformer Single Tensor Fit

```
================================================================================
info : Start Analysis for Structure '#ak-sparteine-1-b3lyp-6311Gdp-1'
in file '5minusspartein_JG-S180.inp'
================================================================================

--------------------------------------------------------------------------------
info : Start Scan of Populations for 2/2 Conformers (nsteps = 20)
--------------------------------------------------------------------------------
info : generated and analyzed 21 populations of 2/2 conformers.
info : continue with best-fit populations.
--------------------------------------------------------------------------------

--------------------------------------------------------------------------------
info : Start Gradient-Descent Optimization of Populations for 2/2 Conformers
--------------------------------------------------------------------------------
info : relative weight of used conformers: w(1)=0.8500 w(2)=0.1500 | iteration    1 |
q = 1.082420e-01 e = 2.460373e+00 de =  2.46037e+00 step = 5.000000e-03
info : conformer populations converged after 1/1000 steps (dxmax = 0.000e+00,
q = 1.082e-01, dq = 0.000e+00, e = 2.460e+00, de = 0.000e+00).
info : continue with optimized best-fit populations.
--------------------------------------------------------------------------------

--------------------------------------------------------------------------------
info : Start Multi-Conformer Single-Tensor (MCST) Fit with 2/2 Conformers and 11 RDCs
info : File: '5minusspartein_JG-S180.inp', Title: '#ak-sparteine-1-b3lyp-6311Gdp-1'
--------------------------------------------------------------------------------
info : relative weight of conformers: w(1) = 0.8500; w(2) = 0.1500

--------------------------------------------------------------------------------
Structure File: '5minusspartein_JG-S180.inp' (Title: '#ak-sparteine-1-b3lyp-6311Gdp-1')
--------------------------------------------------------------------------------
Structure with 2 Conformer(s) and 11 RDCs: File '5minusspartein_JG-S180.inp'
            85.00 % :            Conformer 1 with 43 Atoms:  Title 'Sparteine (Conformer 1)'
            15.00 % :            Conformer 2 with 43 Atoms:  Title 'Sparteine (Conformer 2)'
--------------------------------------------------------------------------------

--------------------------------------------------------------------------------
SVD Best-Fit Saupe Vector S(zz), S(xx-yy), S(xy), S(xz), S(yz):
 -1.862928e-04  9.248697e-04  2.484285e-04 -2.106075e-04 -2.924299e-04
Saupe Tensor (S):                                        Alignment Tensor (A):
  5.555813e-04  2.484285e-04 -2.106075e-04                 3.703875e-04  1.656190e-04 -1.404050e-04
  2.484285e-04 -3.692884e-04 -2.924299e-04                 1.656190e-04 -2.461923e-04 -1.949532e-04
 -2.106075e-04 -2.924299e-04 -1.862928e-04                -1.404050e-04 -1.949532e-04 -1.241952e-04
Trace of Saupe Tensor:        0.000000e+00               Trace of Alignment Tensor:    0.000000e+00
Eigenvectors of Saupe Tensor (S):                        Eigenvectors of Alignment Tensor (A):
 -4.185333e-01  7.645913e-02 -9.049773e-01                -4.185333e-01  7.645913e-02 -9.049773e-01
  4.817691e-01 -8.260064e-01 -2.925953e-01                 4.817691e-01 -8.260064e-01 -2.925953e-01
 -7.698886e-01 -5.584510e-01  3.088755e-01                -7.698886e-01 -5.584510e-01  3.088755e-01
```

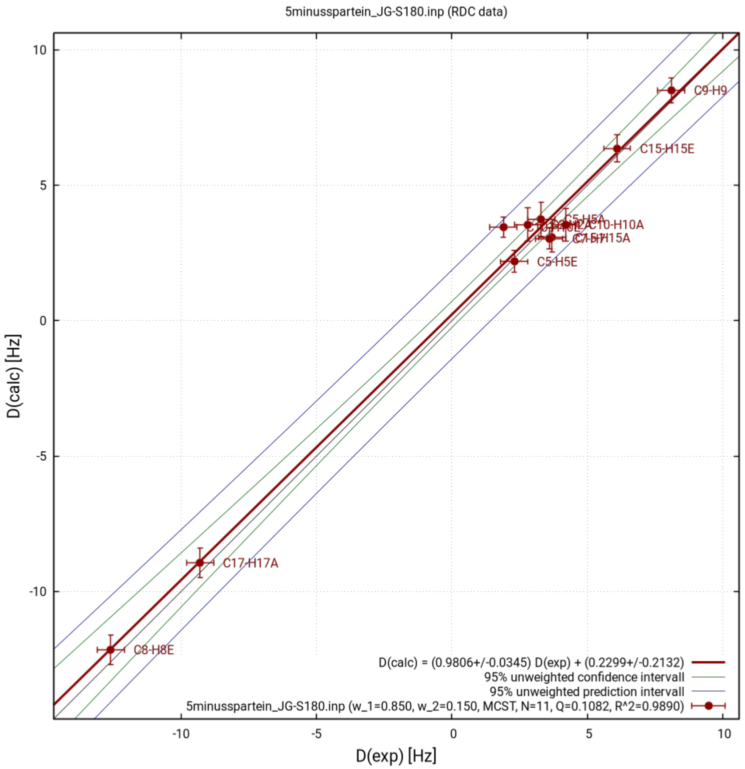

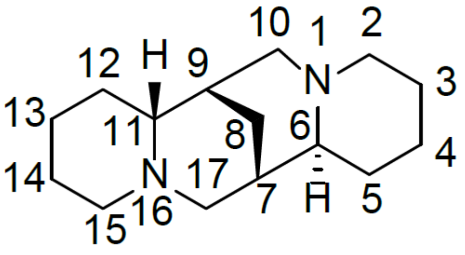

(-)-Sparteine

```
Eigenvalues of Saupe Tensor S(xx), S(yy), S(zz):        Eigenvalues of Alignment Tensor A(xx), A(yy), A(zz):
 -1.177928e-04 -5.899918e-04  7.077845e-04               -7.852853e-05 -3.933278e-04  4.718564e-04

Alignment Tensor Irreducible Representation (A0, A1R, A1I, A2R, A2I):
 -2.953358e-04 -2.726140e-04 -3.785263e-04  5.985837e-04  3.215701e-04

Tensor Properties: *)
A(axial)      =   7.077845e-04         # alignment tensor axial component   = 3/2*A(zz) = S(zz)
A(rhombic)    =   3.147993e-04         # alignment tensor rhombic component = A(xx) - A(yy) = 2/3*(S(xx) - S(yy))
A(rhombicity) =   4.447671e-01         # alignment tensor rhombicity        = A(rhombic) / A(axial)
A(asymmetry)  =   6.671507e-01         # alignment tensor asymmetry         = 3/2*A(rhombicity) = (A(xx) - A(yy))/A(zz) = (S(xx) - S(yy))/S(zz)
GDO           =   7.584741e-04         # generalized degree of order        = sqrt(3/2)*|A(xx),A(yy),A(zz)| = sqrt(2/3)*|S(xx),S(yy),S(zz)|
----------
*) F. Kramer, M.V. Deshmukh, H. Kessler and S.J. Glaser, Concepts Magn. Res. A, 2004, 21A, 21-40.

--------------------------------------------------------------------------------
Results for Multi-Parameter SVD Fit of Calculated and Experimental Data:
           D(calc)[HZ]     +/- Error    D(exp)[HZ]     +/- Error  Rel. Weights            D(exp)-D(calc)        Normalized Weights | RDC Conformers  |     Atom Labels
D[01] =       3.460037             -      1.900000      0.500000      1.000000    r[01] =      -1.560037    w[01] =       0.090909 |   3.498   3.247 |     C10-H10E
D[02] =       3.551296             -      4.200000      0.500000      1.000000    r[02] =       0.648704    w[02] =       0.090909 |   3.418   4.306 |     C10-H10A
D[03] =       3.550366             -      2.800000      0.500000      1.000000    r[03] =      -0.750366    w[03] =       0.090909 |   3.538   3.620 |     C2-H2A
D[04] =       6.359367             -      6.100000      0.500000      1.000000    r[04] =      -0.259367    w[04] =       0.090909 |   7.556  -0.424 |     C15-H15E
D[05] =       3.066812             -      3.700000      0.500000      1.000000    r[05] =       0.633188    w[05] =       0.090909 |   2.945   3.755 |     C15-H15A
D[06] =      -8.943174             -     -9.300000      0.500000      1.000000    r[06] =      -0.356826    w[06] =       0.090909 |  -9.973  -3.108 |     C17-H17A
D[07] =       8.508980             -      8.100000      0.500000      1.000000    r[07] =      -0.408980    w[07] =       0.090909 |   8.496   8.580 |     C9-H9
D[08] =       3.027576             -      3.600000      0.500000      1.000000    r[08] =       0.572424    w[08] =       0.090909 |   3.073   2.770 |     C7-H7
D[09] =       3.745826             -      3.300000      0.500000      1.000000    r[09] =      -0.445826    w[09] =       0.090909 |   3.638   4.358 |     C5-H5A
D[10] =       2.180812             -      2.300000      0.500000      1.000000    r[10] =       0.119188    w[10] =       0.090909 |   2.022   3.081 |     C5-H5E
D[11] =     -12.153075             -    -12.600000      0.500000      1.000000    r[11] =      -0.446925    w[11] =       0.090909 | -11.889 -13.650 |     C8-H8E

Results for Multi-Parameter Fit of Calculated and Experimental Data:
rank  =              5                 # rank of cosine matrix (check input if rank < 5)
cond  =   7.026386e+00                 # condition number of cosine matrix (check input and singular values if very large)
aic   =      31.682983                 # information criterion (AIC) for 6 degrees of freedom
qfac  =       0.108242                 # weighted Q-Factor as defined by Cornilescu
r^2   =       0.987757                 # coefficient of determination r^2 = 1 - chi^2 / (weighted sum of squares)

(|D|) =       5.322484      5.263636   # mean absolute (calc./exp.) parameter D[i]
|D|min=       2.180812      1.900000   # min. absolute (calc./exp.) parameter D[i]
|D|max=      12.153075     12.600000   # max. absolute (calc./exp.) parameter D[i]
Drange=     -12.153075      8.508980   # min. and max. (calc.)      parameter D[i]
Drange=     -12.600000      8.100000   # min. and max. (exp.)       parameter D[i]

Results for Linear Regression of Calculated and Experimental Data (N=11):
c(b)  =       0.229895 +/-  0.213210   # linear regression intercept and error
c(m)  =       0.980566 +/-  0.034505   # linear regression slope and error
rmsd  =       0.668835                 # unweighted total root-mean-square deviation
chisq =       0.447341                 # weighted total sum of squared residuals
maerr =       0.563803                 # weighted total mean absolute error (sum of weights = 1.000)
R     =       0.994474                 # weighted Pearson correlation coefficient R
R^2   =       0.988978                 # weighted Pearson correlation coefficient R^2

E(RDC)=       2.460373                 # E(RDC)=1/2*K*(sum of weighted deviations (D(exp)-D(calc))^2), K=1.000
```

3.1.22 (-)-IPC in Stick S181 – 300K out of T-series

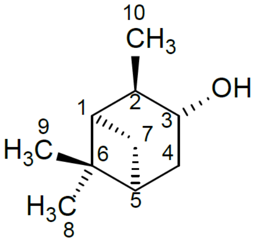

(-)-IPC

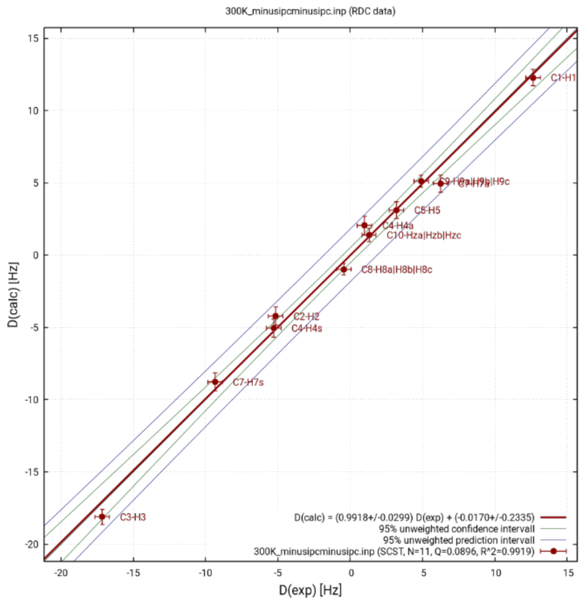


```
================================================================================
info : Start Analysis for Structure '' in file '300K_minusipcminusipc.inp'
================================================================================

--------------------------------------------------------------------------------
info : Start Single-Conformer Single-Tensor (SCST) Fit with 11 RDCs
info : File: '300K_minusipcminusipc.inp', Title: ''
--------------------------------------------------------------------------------

--------------------------------------------------------------------------------
SVD Best-Fit Saupe Vector S(zz), S(xx-yy), S(xy), S(xz), S(yz):
 -1.568504e-04 -5.701348e-04 -3.074102e-04  4.436025e-04 -4.101745e-04
Saupe Tensor (S):                                        Alignment Tensor (A):
 -2.066422e-04 -3.074102e-04  4.436025e-04                -1.377615e-04 -2.049401e-04  2.957350e-04
 -3.074102e-04  3.634926e-04 -4.101745e-04                -2.049401e-04  2.423284e-04 -2.734497e-04
  4.436025e-04 -4.101745e-04 -1.568504e-04                 2.957350e-04 -2.734497e-04 -1.045669e-04
Trace of Saupe Tensor:         2.710505e-20              Trace of Alignment Tensor:    1.807004e-20
Eigenvectors of Saupe Tensor (S):                        Eigenvectors of Alignment Tensor (A):
  5.744507e-01  6.910183e-01 -4.387483e-01                 5.744507e-01  6.910183e-01 -4.387483e-01
  6.657491e-01 -8.258555e-02  7.415914e-01                 6.657491e-01 -8.258555e-02  7.415914e-01
  4.762189e-01 -7.181040e-01 -5.074861e-01                 4.762189e-01 -7.181040e-01 -5.074861e-01
Eigenvalues of Saupe Tensor S(xx), S(yy), S(zz):         Eigenvalues of Alignment Tensor A(xx), A(yy), A(zz):
 -1.951637e-04 -6.308931e-04  8.260567e-04                -1.301091e-04 -4.205954e-04  5.507045e-04

Alignment Tensor Irreducible Representation (A0, A1R, A1I, A2R, A2I):
 -2.486597e-04  5.742068e-04 -5.309370e-04 -3.689962e-04 -3.979171e-04

Tensor Properties: *)
A(axial)      =   8.260567e-04         # alignment tensor axial component   = 3/2*A(zz) = S(zz)
A(rhombic)    =   2.904863e-04         # alignment tensor rhombic component = A(xx) - A(yy) = 2/3*(S(xx) - S(yy))
A(rhombicity) =   3.516542e-01         # alignment tensor rhombicity        = A(rhombic) / A(axial)
A(asymmetry)  =   5.274812e-01         # alignment tensor asymmetry         = 3/2*A(rhombicity) = (A(xx) - A(yy))/A(zz) = (S(xx) - S(yy))/S(zz)
GDO           =   8.635140e-04         # generalized degree of order        = sqrt(3/2)*|A(xx),A(yy),A(zz)| = sqrt(2/3)*|S(xx),S(yy),S(zz)|
----------
*) F. Kramer, M.V. Deshmukh, H. Kessler and S.J. Glaser, Concepts Magn. Res. A, 2004, 21A, 21-40.
```

```
-------------------------------------------------------------------------------
Results for Multi-Parameter SVD Fit of Calculated and Experimental Data:
           D(calc)[HZ]     +/- Error    D(exp)[HZ]     +/- Error  Rel. Weights            D(exp)-D(calc)       Normalized Weights    Atom Labels
D[01] =      12.284343             -     12.650000      0.500000      1.000000    r[01] =       0.365657    w[01] =       0.090909    C1-H1
D[02] =      -4.229754             -     -5.150000      0.500000      1.000000    r[02] =      -0.920246    w[02] =       0.090909    C2-H2
D[03] =     -18.112606             -    -17.150000      0.500000      1.000000    r[03] =       0.962606    w[03] =       0.090909    C3-H3
D[04] =      -5.044267             -     -5.300000      0.500000      1.000000    r[04] =      -0.255733    w[04] =       0.090909    C4-H4s
D[05] =       2.050762             -      1.000000      0.500000      1.000000    r[05] =      -1.050762    w[05] =       0.090909    C4-H4a
D[06] =       3.118032             -      3.200000      0.500000      1.000000    r[06] =       0.081968    w[06] =       0.090909    C5-H5
D[07] =      -8.780355             -     -9.350000      0.500000      1.000000    r[07] =      -0.569645    w[07] =       0.090909    C7-H7s
D[08] =       4.962520             -      6.250000      0.500000      1.000000    r[08] =       1.287480    w[08] =       0.090909    C7-H7a
D[09] =      -0.977893             -     -0.450000      0.500000      1.000000    r[09] =       0.527893    w[09] =       0.090909    C8-H8a|H8b|H8c
             10.656371             -             -             -             -                         -                         -    | [3av] C8-H8a
              5.136773             -             -             -             -                         -                         -    | [3av] C8-H8b
            -18.726824             -             -             -             -                         -                         -    | [3av] C8-H8c
D[10] =       5.110845             -      4.900000      0.500000      1.000000    r[10] =      -0.210845    w[10] =       0.090909    C9-H9a|H9b|H9c
              5.211322             -             -             -             -                         -                         -    | [3av] C9-H9a
             10.389447             -             -             -             -                         -                         -    | [3av] C9-H9b
             -0.268235             -             -             -             -                         -                         -    | [3av] C9-H9c
D[11] =       1.397780             -      1.300000      0.500000      1.000000    r[11] =      -0.097780    w[11] =       0.090909    C10-Hza|Hzb|Hzc
              9.142439             -             -             -             -                         -                         -    | [3av] C10-Hza
             -5.239902             -             -             -             -                         -                         -    | [3av] C10-Hzb
              0.290803             -             -             -             -                         -                         -    | [3av] C10-Hzc

Results for Multi-Parameter Fit of Calculated and Experimental Data:
rank  =              5                 # rank of cosine matrix (check input if rank < 5)
cond  =   2.147425e+00                 # condition number of cosine matrix (check input and singular values if very large)
aic   =      31.592701                 # information criterion (AIC) for 5 degrees of freedom
qfac  =       0.089601                 # weighted Q-Factor as defined by Cornilescu
r^2   =       0.991900                 # coefficient of determination r^2 = 1 - chi^2 / (weighted sum of squares)

(|D|) =       6.006287      6.063636   # mean absolute (calc./exp.) parameter D[i]
|D|min=       0.977893      0.450000   # min. absolute (calc./exp.) parameter D[i]
|D|max=      18.112606     17.150000   # max. absolute (calc./exp.) parameter D[i]
Drange=     -18.112606     12.284343   # min. and max. (calc.)      parameter D[i]
Drange=     -17.150000     12.650000   # min. and max. (exp.)       parameter D[i]

Results for Linear Regression of Calculated and Experimental Data (N=11):
c(b)  =      -0.017026 +/-  0.233540   # linear regression intercept and error
c(m)  =       0.991766 +/-  0.029871   # linear regression slope and error
rmsd  =       0.700531                 # unweighted total root-mean-square deviation
chisq =       0.490743                 # weighted total sum of squared residuals
maerr =       0.575510                 # weighted total mean absolute error (sum of weights = 1.000)
R     =       0.995943                 # weighted Pearson correlation coefficient R
R^2   =       0.991902                 # weighted Pearson correlation coefficient R^2

E(RDC)=       2.699088                 # E(RDC)=1/2*K*(sum of weighted deviations (D(exp)-D(calc))^2), K=1.000
```

3.1.23 (-)-Strychnine in Stick S185

in *ent*-p1

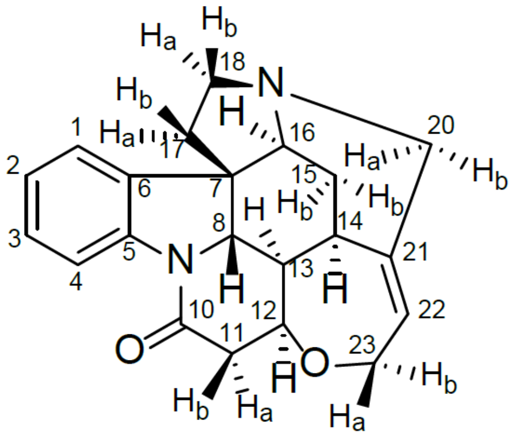

(-)-Strychnine

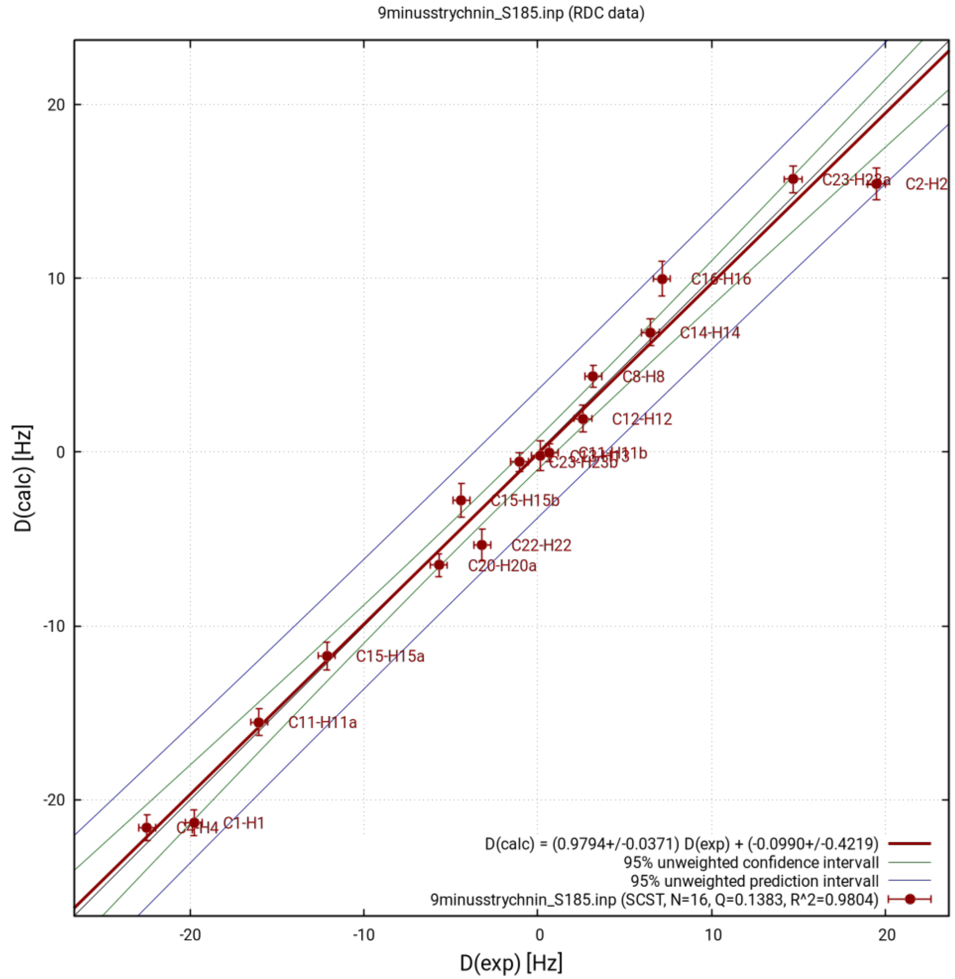

```
================================================================================
info : Start Analysis for Structure '#AK-XXX' in file '9minusstrychnin_S185.inp'
================================================================================

--------------------------------------------------------------------------------
info : Start Single-Conformer Single-Tensor (SCST) Fit with 16 RDCs
info : File: '9minusstrychnin_S185.inp', Title: '#AK-XXX'
--------------------------------------------------------------------------------

--------------------------------------------------------------------------------
SVD Best-Fit Saupe Vector S(zz), S(xx-yy), S(xy), S(xz), S(yz):
 -2.436099e-04 -1.496568e-04 -5.927241e-04  7.481667e-04 -3.798326e-06
Saupe Tensor (S):                                      Alignment Tensor (A):
  4.697655e-05 -5.927241e-04  7.481667e-04               3.131770e-05 -3.951494e-04  4.987778e-04
 -5.927241e-04  1.966333e-04 -3.798326e-06              -3.951494e-04  1.310889e-04 -2.532217e-06
  7.481667e-04 -3.798326e-06 -2.436099e-04               4.987778e-04 -2.532217e-06 -1.624066e-04
Trace of Saupe Tensor:        2.710505e-20             Trace of Alignment Tensor:    1.807004e-20
Eigenvectors of Saupe Tensor (S):                      Eigenvectors of Alignment Tensor (A):
  2.193978e-01  7.107763e-01  6.683275e-01               2.193978e-01  7.107763e-01  6.683275e-01
  7.679541e-01 -5.483211e-01  3.310446e-01               7.679541e-01 -5.483211e-01  3.310446e-01
  6.017567e-01  4.406144e-01 -6.661440e-01               6.017567e-01  4.406144e-01 -6.661440e-01
Eigenvalues of Saupe Tensor S(xx), S(yy), S(zz):       Eigenvalues of Alignment Tensor A(xx), A(yy), A(zz):
  2.432093e-05  9.680204e-04 -9.923414e-04               1.621395e-05  6.453470e-04 -6.615609e-04

Alignment Tensor Irreducible Representation (A0, A1R, A1I, A2R, A2I):
 -3.862023e-04  9.684398e-04 -4.916619e-06 -9.685915e-05 -7.672323e-04

Tensor Properties: *)
A(axial)      =  -9.923414e-04         # alignment tensor axial component   = 3/2*A(zz) = S(zz)
A(rhombic)    =  -6.291330e-04         # alignment tensor rhombic component = A(xx) - A(yy) = 2/3*(S(xx) - S(yy))
A(rhombicity) =   6.339885e-01         # alignment tensor rhombicity        = A(rhombic) / A(axial)
A(asymmetry)  =   9.509827e-01         # alignment tensor asymmetry         = 3/2*A(rhombicity) = (A(xx) - A(yy))/A(zz) = (S(xx) - S(yy))/S(zz)
GDO           =   1.132077e-03         # generalized degree of order        = sqrt(3/2)*|A(xx),A(yy),A(zz)| = sqrt(2/3)*|S(xx),S(yy),S(zz)|
----------
*) F. Kramer, M.V. Deshmukh, H. Kessler and S.J. Glaser, Concepts Magn. Res. A, 2004, 21A, 21-40.
```

```
--------------------------------------------------------------------------------
Results for Multi-Parameter SVD Fit of Calculated and Experimental Data:
           D(calc)[HZ]     +/- Error    D(exp)[HZ]     +/- Error  Rel. Weights            D(exp)-D(calc)        Normalized Weights    Atom Labels
D[01] =      -5.343967             -     -3.200000      0.500000      1.000000    r[01] =       2.143967    w[01] =       0.062500    C22-H22
D[02] =      15.449867             -     19.500000      0.500000      1.000000    r[02] =       4.050133    w[02] =       0.062500    C2-H2
D[03] =     -21.325998             -    -19.800000      0.500000      1.000000    r[03] =       1.525998    w[03] =       0.062500    C1-H1
D[04] =     -21.606556             -    -22.500000      0.500000      1.000000    r[04] =      -0.893444    w[04] =       0.062500    C4-H4
D[05] =       1.922478             -      2.600000      0.500000      1.000000    r[05] =       0.677522    w[05] =       0.062500    C12-H12
D[06] =      15.694200             -     14.700000      0.500000      1.000000    r[06] =      -0.994200    w[06] =       0.062500    C23-H23a
D[07] =      -0.561539             -     -1.050000      0.500000      1.000000    r[07] =      -0.488461    w[07] =       0.062500    C23-H23b
D[08] =       9.977666             -      7.150000      0.500000      1.000000    r[08] =      -2.827666    w[08] =       0.062500    C16-H16
D[09] =       4.353226             -      3.200000      0.500000      1.000000    r[09] =      -1.153226    w[09] =       0.062500    C8-H8
D[10] =      -6.516068             -     -5.700000      0.500000      1.000000    r[10] =       0.816068    w[10] =       0.062500    C20-H20a
D[11] =      -0.220391             -      0.150000      0.500000      1.000000    r[11] =       0.370391    w[11] =       0.062500    C13-H13
D[12] =     -15.538543             -    -16.050000      0.500000      1.000000    r[12] =      -0.511457    w[12] =       0.062500    C11-H11a
D[13] =      -0.033805             -      0.650000      0.500000      1.000000    r[13] =       0.683805    w[13] =       0.062500    C11-H11b
D[14] =       6.883235             -      6.500000      0.500000      1.000000    r[14] =      -0.383235    w[14] =       0.062500    C14-H14
D[15] =     -11.725582             -    -12.150000      0.500000      1.000000    r[15] =      -0.424418    w[15] =       0.062500    C15-H15a
D[16] =      -2.766636             -     -4.400000      0.500000      1.000000    r[16] =      -1.633364    w[16] =       0.062500    C15-H15b

Results for Multi-Parameter Fit of Calculated and Experimental Data:
rank  =              5                # rank of cosine matrix (check input if rank < 5)
cond  =   3.718153e+00                # condition number of cosine matrix (check input and singular values if very large)
aic   =     168.663946                # information criterion (AIC) for 5 degrees of freedom
qfac  =       0.138299                # weighted Q-Factor as defined by Cornilescu
r^2   =       0.980325                # coefficient of determination r^2 = 1 - chi^2 / (weighted sum of squares)

(|D|) =       8.744985      8.706250   # mean absolute (calc./exp.) parameter D[i]
|D|min=       0.033805      0.150000   # min. absolute (calc./exp.) parameter D[i]
|D|max=      21.606556     22.500000   # max. absolute (calc./exp.) parameter D[i]
Drange=     -21.606556     15.694200   # min. and max. (calc.)      parameter D[i]
Drange=     -22.500000     19.500000   # min. and max. (exp.)       parameter D[i]

Results for Linear Regression of Calculated and Experimental Data (N=16):
c(b)  =      -0.098999 +/-  0.421864   # linear regression intercept and error
c(m)  =       0.979422 +/-  0.037055   # linear regression slope and error
rmsd  =       1.574523                 # unweighted total root-mean-square deviation
chisq =       2.479124                 # weighted total sum of squared residuals
maerr =       1.223585                 # weighted total mean absolute error (sum of weights = 1.000)
R     =       0.990129                 # weighted Pearson correlation coefficient R
R^2   =       0.980355                 # weighted Pearson correlation coefficient R^2

E(RDC)=      19.832993                 # E(RDC)=1/2*K*(sum of weighted deviations (D(exp)-D(calc))^2), K=1.000
```

3.1.24 Cholesterine in Stick S187

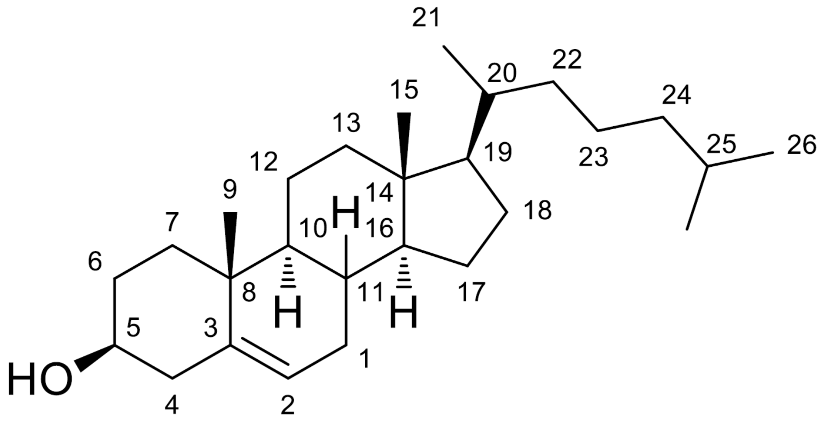

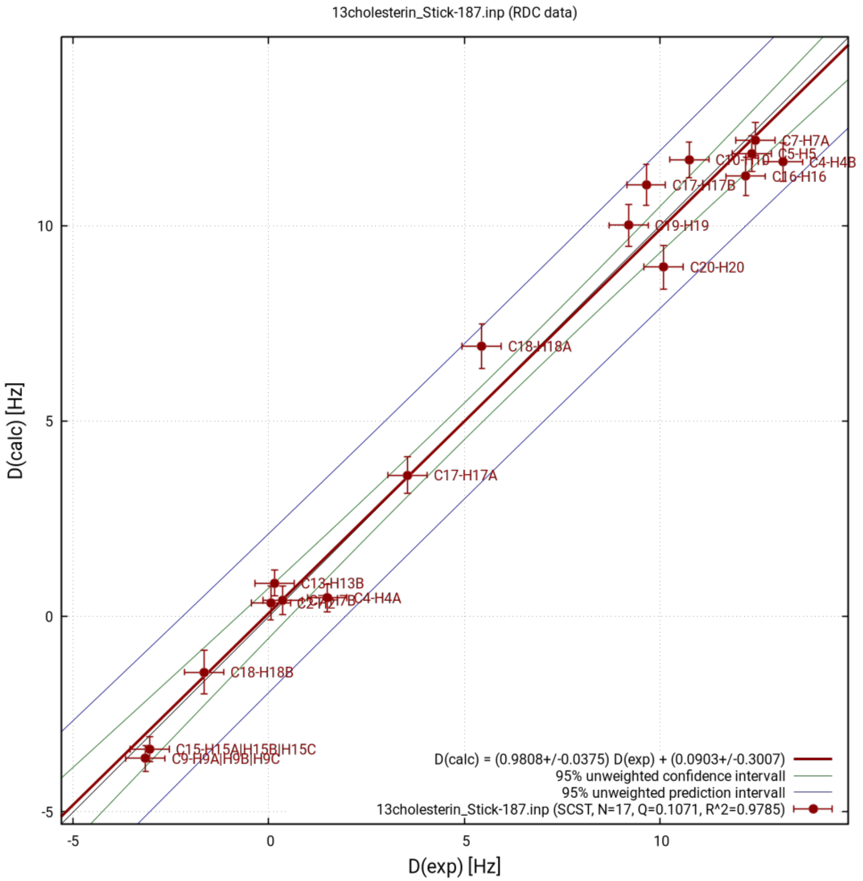


```
==================================================================================
info : Start Analysis for Structure '' in file '13cholesterin_Stick-187.inp'
==================================================================================

----------------------------------------------------------------------------------
info : Start Single-Conformer Single-Tensor (SCST) Fit with 17 RDCs
info : File: '13cholesterin_Stick-187.inp', Title: ''
----------------------------------------------------------------------------------

----------------------------------------------------------------------------------
SVD Best-Fit Saupe Vector S(zz), S(xx-yy), S(xy), S(xz), S(yz):
 -4.654190e-04  1.833498e-04 -2.319962e-04 -2.739196e-04  1.192482e-04
Saupe Tensor (S):                                        Alignment Tensor (A):
  3.243844e-04 -2.319962e-04 -2.739196e-04                 2.162563e-04 -1.546642e-04 -1.826131e-04
 -2.319962e-04  1.410346e-04  1.192482e-04                -1.546642e-04  9.402304e-05  7.949877e-05
 -2.739196e-04  1.192482e-04 -4.654190e-04                -1.826131e-04  7.949877e-05 -3.102793e-04
Trace of Saupe Tensor:         0.000000e+00              Trace of Alignment Tensor:    0.000000e+00
Eigenvectors of Saupe Tensor (S):                        Eigenvectors of Alignment Tensor (A):
 -5.162705e-01  2.797702e-01 -8.094402e-01                -5.162705e-01  2.797702e-01 -8.094402e-01
 -8.519122e-01 -7.085720e-02  5.188689e-01                -8.519122e-01 -7.085720e-02  5.188689e-01
  8.780939e-02  9.574486e-01  2.749211e-01                 8.780939e-02  9.574486e-01  2.749211e-01
```

```
Eigenvalues of Saupe Tensor S(xx), S(yy), S(zz):        Eigenvalues of Alignment Tensor A(xx), A(yy), A(zz):
 -1.184964e-05 -5.542844e-04  5.661341e-04               -7.899762e-06 -3.695230e-04  3.774227e-04

Alignment Tensor Irreducible Representation (A0, A1R, A1I, A2R, A2I):
 -7.378431e-04 -3.545663e-04  1.543569e-04  1.186656e-04 -3.002999e-04

Tensor Properties: *)
A(axial)      =   5.661341e-04         # alignment tensor axial component   = 3/2*A(zz) = S(zz)
A(rhombic)    =   3.616232e-04         # alignment tensor rhombic component = A(xx) - A(yy) = 2/3*(S(xx) - S(yy))
A(rhombicity) =   6.387589e-01         # alignment tensor rhombicity        = A(rhombic) / A(axial)
A(asymmetry)  =   9.581384e-01         # alignment tensor asymmetry         = 3/2*A(rhombicity) = (A(xx) - A(yy))/A(zz) = (S(xx) - S(yy))/S(zz)
GDO           =   6.469825e-04         # generalized degree of order        = sqrt(3/2)*|A(xx),A(yy),A(zz)| = sqrt(2/3)*|S(xx),S(yy),S(zz)|
----------
*) F. Kramer, M.V. Deshmukh, H. Kessler and S.J. Glaser, Concepts Magn. Res. A, 2004, 21A, 21-40.


--------------------------------------------------------------------------------
Results for Multi-Parameter SVD Fit of Calculated and Experimental Data:
           D(calc)[HZ]     +/- Error    D(exp)[HZ]     +/- Error  Rel. Weights            D(exp)-D(calc)        Normalized Weights    Atom Labels
D[01] =       0.344298             -      0.050000      0.500000      1.000000    r[01] =      -0.294298    w[01] =       0.058824    C2-H2
D[02] =       0.482076             -      1.500000      0.500000      1.000000    r[02] =       1.017924    w[02] =       0.058824    C4-H4A
D[03] =      11.630148             -     13.150000      0.500000      1.000000    r[03] =       1.519852    w[03] =       0.058824    C4-H4B
D[04] =      11.847375             -     12.350000      0.500000      1.000000    r[04] =       0.502625    w[04] =       0.058824    C5-H5
D[05] =      12.182377             -     12.450000      0.500000      1.000000    r[05] =       0.267623    w[05] =       0.058824    C7-H7A
D[06] =       0.414756             -      0.350000      0.500000      1.000000    r[06] =      -0.064756    w[06] =       0.058824    C7-H7B
D[07] =      -3.630801             -     -3.150000      0.500000      1.000000    r[07] =       0.480801    w[07] =       0.058824    C9-H9A|H9B|H9C
             -3.468674             -             -             -             -                       -                       -    | [3av] C9-H9A
             -7.350082             -             -             -             -                       -                       -    | [3av] C9-H9B
             -0.073646             -             -             -             -                       -                       -    | [3av] C9-H9C
D[08] =      11.691929             -     10.750000      0.500000      1.000000    r[08] =      -0.941929    w[08] =       0.058824    C10-H10
D[09] =       0.854741             -      0.150000      0.500000      1.000000    r[09] =      -0.704741    w[09] =       0.058824    C13-H13B
D[10] =      -3.395217             -     -3.050000      0.500000      1.000000    r[10] =       0.345217    w[10] =       0.058824    C15-H15A|H15B|H15C
             -1.951823             -             -             -             -                       -                       -    | [3av] C15-H15A
             -8.983512             -             -             -             -                       -                       -    | [3av] C15-H15B
              0.749683             -             -             -             -                       -                       -    | [3av] C15-H15C
D[11] =      11.266106             -     12.200000      0.500000      1.000000    r[11] =       0.933894    w[11] =       0.058824    C16-H16
D[12] =       3.620021             -      3.550000      0.500000      1.000000    r[12] =      -0.070021    w[12] =       0.058824    C17-H17A
D[13] =      11.044179             -      9.650000      0.500000      1.000000    r[13] =      -1.394179    w[13] =       0.058824    C17-H17B
D[14] =       6.917526             -      5.450000      0.500000      1.000000    r[14] =      -1.467526    w[14] =       0.058824    C18-H18A
D[15] =      -1.421222             -     -1.650000      0.500000      1.000000    r[15] =      -0.228778    w[15] =       0.058824    C18-H18B
D[16] =      10.009329             -      9.200000      0.500000      1.000000    r[16] =      -0.809329    w[16] =       0.058824    C19-H19
D[17] =       8.944361             -     10.100000      0.500000      1.000000    r[17] =       1.155639    w[17] =       0.058824    C20-H20

Results for Multi-Parameter Fit of Calculated and Experimental Data:
rank  =              5                 # rank of cosine matrix (check input if rank < 5)
cond  =   1.346469e+01                 # condition number of cosine matrix (check input and singular values if very large)
aic   =      60.050781                 # information criterion (AIC) for 5 degrees of freedom
qfac  =       0.107080                 # weighted Q-Factor as defined by Cornilescu
r^2   =       0.978499                 # coefficient of determination r^2 = 1 - chi^2 / (weighted sum of squares)

(|D|) =       6.452733      6.397059   # mean absolute (calc./exp.) parameter D[i]
|D|min=       0.344298      0.050000   # min. absolute (calc./exp.) parameter D[i]
|D|max=      12.182377     13.150000   # max. absolute (calc./exp.) parameter D[i]
Drange=      -3.630801     12.182377   # min. and max. (calc.)      parameter D[i]
Drange=      -3.150000     13.150000   # min. and max. (exp.)       parameter D[i]
```

```
Results for Linear Regression of Calculated and Experimental Data (N=17):
c(b)  =       0.090329 +/-  0.300690   # linear regression intercept and error
c(m)  =       0.980832 +/-  0.037530   # linear regression slope and error
rmsd  =       0.857928                 # unweighted total root-mean-square deviation
chisq =       0.736041                 # weighted total sum of squared residuals
maerr =       0.717596                 # weighted total mean absolute error (sum of weights = 1.000)
R     =       0.989197                 # weighted Pearson correlation coefficient R
R^2   =       0.978511                 # weighted Pearson correlation coefficient R^2

E(RDC)=       6.256348                 # E(RDC)=1/2*K*(sum of weighted deviations (D(exp)-D(calc))^2), K=1.000
```

3.1.25 (-)-Perilla acid in Stick S188 @ 310K

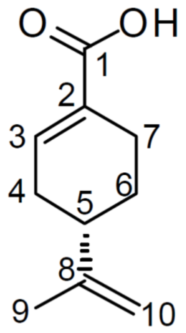

(-)-Perilla acid

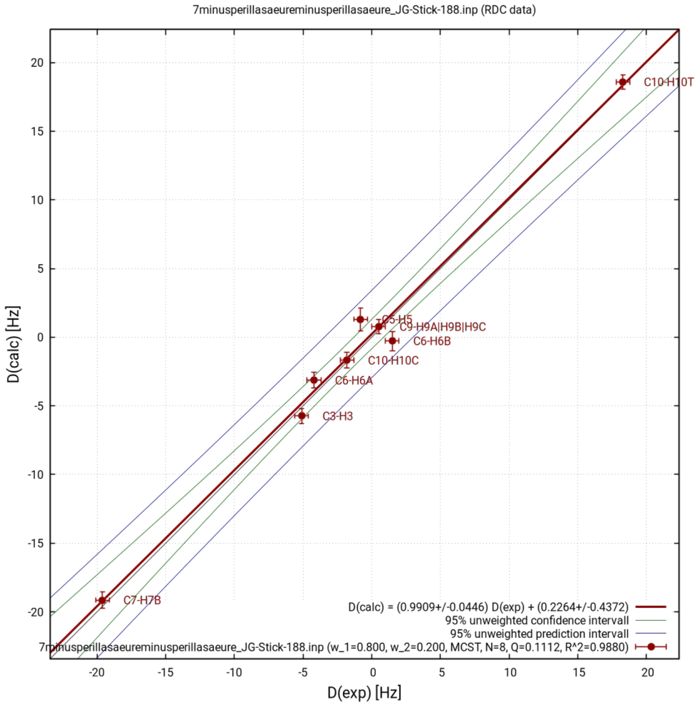

Multi-Conformer-Single Tensor Fit

```
================================================================================
info : Start Analysis for Structure ''
in file '7minusperillasaeureminusperillasaeure_JG-Stick-188.inp'
================================================================================

--------------------------------------------------------------------------------
info : Start Scan of Populations for 2/2 Conformers (nsteps = 20)
--------------------------------------------------------------------------------
info : generated and analyzed 21 populations of 2/2 conformers.
info : continue with best-fit populations.
--------------------------------------------------------------------------------
info : Start Gradient-Descent Optimization of Populations for 2/2 Conformers
--------------------------------------------------------------------------------
info : relative weight of used conformers: w(1)=0.8000 w(2)=0.2000 | iteration   1 |
q = 1.111769e-01 e = 4.753034e+00 de =  4.75303e+00 step = 5.000000e-03
info : conformer populations converged after 1/1000 steps (dxmax = 0.000e+00,
q = 1.112e-01, dq = 0.000e+00, e = 4.753e+00, de = 0.000e+00).
info : continue with optimized best-fit populations.
--------------------------------------------------------------------------------

--------------------------------------------------------------------------------
info : Start Multi-Conformer Single-Tensor (MCST) Fit with 2/2 Conformers and 8 RDCs
info : File: '7minusperillasaeureminusperillasaeure_JG-Stick-188.inp', Title: ''
--------------------------------------------------------------------------------
info : relative weight of conformers: w(1) = 0.8000; w(2) = 0.2000

--------------------------------------------------------------------------------
Structure File: '7minusperillasaeureminusperillasaeure_JG-Stick-188.inp' (Title: '')
--------------------------------------------------------------------------------
Structure with 2 Conformer(s) and 8 RDCs: File '7minusperillasaeureminusperillasaeure_JG-Stick-188.inp'
            80.00 % :            Conformer 1 with 26 Atoms:  Title 'ohoh'
            20.00 % :            Conformer 2 with 26 Atoms:  Title 'ohoh'
--------------------------------------------------------------------------------

--------------------------------------------------------------------------------
SVD Best-Fit Saupe Vector S(zz), S(xx-yy), S(xy), S(xz), S(yz):
  6.093381e-04 -8.088375e-04  2.541769e-04  3.622508e-04 -7.038389e-04
Saupe Tensor (S):                                       Alignment Tensor (A):
 -7.090878e-04  2.541769e-04  3.622508e-04               -4.727252e-04  1.694512e-04  2.415005e-04
  2.541769e-04  9.974968e-05 -7.038389e-04                1.694512e-04  6.649979e-05 -4.692259e-04
  3.622508e-04 -7.038389e-04  6.093381e-04                2.415005e-04 -4.692259e-04  4.062254e-04
Trace of Saupe Tensor:        0.000000e+00              Trace of Alignment Tensor:    0.000000e+00
Eigenvectors of Saupe Tensor (S):                       Eigenvectors of Alignment Tensor (A):
 -5.589186e-01  8.245763e-01  8.765831e-02               -5.589186e-01  8.245763e-01  8.765831e-02
 -7.162381e-01 -4.267882e-01 -5.521366e-01               -7.162381e-01 -4.267882e-01 -5.521366e-01
 -4.178672e-01 -3.713836e-01  8.291328e-01               -4.178672e-01 -3.713836e-01  8.291328e-01
Eigenvalues of Saupe Tensor S(xx), S(yy), S(zz):        Eigenvalues of Alignment Tensor A(xx), A(yy), A(zz):
 -1.125360e-04 -1.003801e-03  1.116337e-03               -7.502397e-05 -6.692008e-04  7.442248e-04
```

```
Alignment Tensor Irreducible Representation (A0, A1R, A1I, A2R, A2I):
  9.660026e-04  4.689037e-04 -9.110613e-04 -5.234866e-04  3.290109e-04

Tensor Properties: *)
A(axial)      =   1.116337e-03         # alignment tensor axial component   = 3/2*A(zz) = S(zz)
A(rhombic)    =   5.941769e-04         # alignment tensor rhombic component = A(xx) - A(yy) = 2/3*(S(xx) - S(yy))
A(rhombicity) =   5.322557e-01         # alignment tensor rhombicity        = A(rhombic) / A(axial)
A(asymmetry)  =   7.983836e-01         # alignment tensor asymmetry         = 3/2*A(rhombicity) = (A(xx) - A(yy))/A(zz) = (S(xx) - S(yy))/S(zz)
GDO           =   1.229225e-03         # generalized degree of order        = sqrt(3/2)*|A(xx),A(yy),A(zz)| = sqrt(2/3)*|S(xx),S(yy),S(zz)|
----------
*) F. Kramer, M.V. Deshmukh, H. Kessler and S.J. Glaser, Concepts Magn. Res. A, 2004, 21A, 21-40.

--------------------------------------------------------------------------------
Results for Multi-Parameter SVD Fit of Calculated and Experimental Data:
           D(calc)[HZ]     +/- Error    D(exp)[HZ]     +/- Error  Rel. Weights            D(exp)-D(calc)        Normalized Weights | RDC Conformers  |    Atom Labels
D[01] =       0.767343             -      0.500000      0.500000      1.000000    r[01] =      -0.267343    w[01] =       0.125000 |  -0.432   5.566 |    C9-H9A|H9B|H9C
D[02] =      -3.130313             -     -4.200000      0.500000      1.000000    r[02] =      -1.069687    w[02] =       0.125000 |  -1.909  -8.015 |    C6-H6A
D[03] =      -0.268791             -      1.500000      0.500000      1.000000    r[03] =       1.768791    w[03] =       0.125000 |  -1.795   5.837 |    C6-H6B
D[04] =       1.309116             -     -0.800000      0.500000      1.000000    r[04] =      -2.109116    w[04] =       0.125000 |  -0.258   7.579 |    C5-H5
D[05] =      18.585810             -     18.300000      0.500000      1.000000    r[05] =      -0.285810    w[05] =       0.125000 |  18.337  19.581 |    C10-H10T
D[06] =      -1.677186             -     -1.800000      0.500000      1.000000    r[06] =      -0.122814    w[06] =       0.125000 |   2.787 -19.536 |    C10-H10C
D[07] =      -5.734982             -     -5.100000      0.500000      1.000000    r[07] =       0.634982    w[07] =       0.125000 |  -4.705  -9.854 |    C3-H3
D[08] =     -19.138049             -    -19.600000      0.500000      1.000000    r[08] =      -0.461951    w[08] =       0.125000 | -19.260 -18.650 |    C7-H7B

Results for Multi-Parameter Fit of Calculated and Experimental Data:
rank  =             5                  # rank of cosine matrix (check input if rank < 5)
cond  =   7.260726e+00                 # condition number of cosine matrix (check input and singular values if very large)
aic   =      50.024272                 # information criterion (AIC) for 6 degrees of freedom
qfac  =       0.111177                 # weighted Q-Factor as defined by Cornilescu
r^2   =       0.987382                 # coefficient of determination r^2 = 1 - chi^2 / (weighted sum of squares)

(|D|) =       6.326449      6.475000   # mean absolute (calc./exp.) parameter D[i]
|D|min=       0.268791      0.500000   # min. absolute (calc./exp.) parameter D[i]
|D|max=      19.138049     19.600000   # max. absolute (calc./exp.) parameter D[i]
Drange=     -19.138049     18.585810   # min. and max. (calc.)      parameter D[i]
Drange=     -19.600000     18.300000   # min. and max. (exp.)       parameter D[i]

Results for Linear Regression of Calculated and Experimental Data (N=8):
c(b)  =       0.226431 +/-  0.437174   # linear regression intercept and error
c(m)  =       0.990937 +/-  0.044588   # linear regression slope and error
rmsd  =       1.090073                 # unweighted total root-mean-square deviation
chisq =       1.188259                 # weighted total sum of squared residuals
maerr =       0.840062                 # weighted total mean absolute error (sum of weights = 1.000)
R     =       0.993981                 # weighted Pearson correlation coefficient R
R^2   =       0.987998                 # weighted Pearson correlation coefficient R^2

E(RDC)=       4.753034                 # E(RDC)=1/2*K*(sum of weighted deviations (D(exp)-D(calc))^2), K=1.000
```

3.1.26 Cholesterine in Stick S190

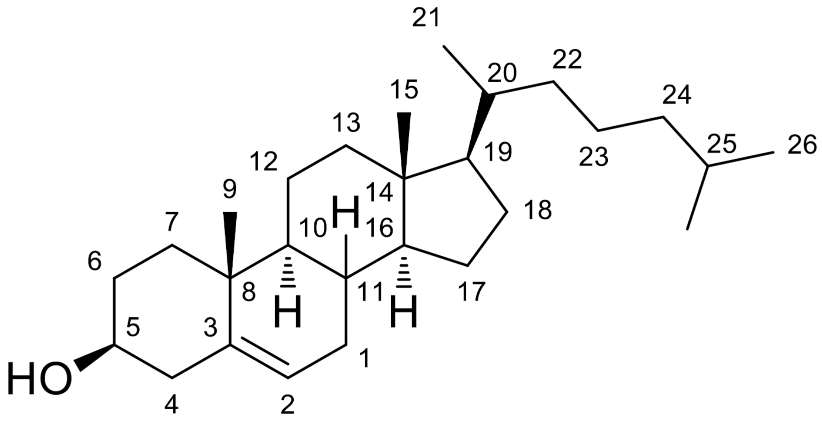

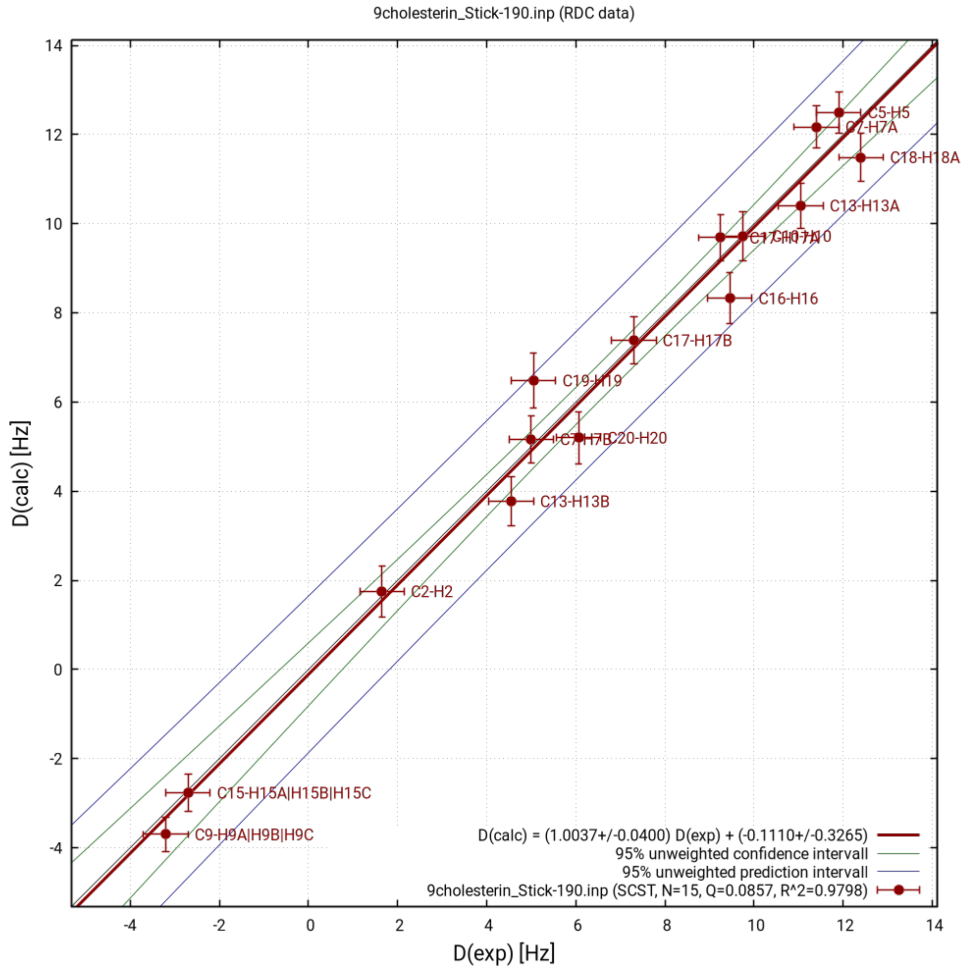


```
================================================================================
info : Start Analysis for Structure '' in file '9cholesterin_Stick-190.inp'
================================================================================

--------------------------------------------------------------------------------
info : Start Single-Conformer Single-Tensor (SCST) Fit with 15 RDCs
info : File: '9cholesterin_Stick-190.inp', Title: ''
--------------------------------------------------------------------------------

--------------------------------------------------------------------------------
SVD Best-Fit Saupe Vector S(zz), S(xx-yy), S(xy), S(xz), S(yz):
 -3.316300e-04  8.163186e-04 -4.377954e-05 -3.453193e-04  2.502978e-04
Saupe Tensor (S):                                       Alignment Tensor (A):
  5.739743e-04 -4.377954e-05 -3.453193e-04                3.826495e-04 -2.918636e-05 -2.302129e-04
 -4.377954e-05 -2.423443e-04  2.502978e-04               -2.918636e-05 -1.615629e-04  1.668652e-04
 -3.453193e-04  2.502978e-04 -3.316300e-04               -2.302129e-04  1.668652e-04 -2.210867e-04
Trace of Saupe Tensor:         5.421011e-20             Trace of Alignment Tensor:    3.614007e-20
Eigenvectors of Saupe Tensor (S):                       Eigenvectors of Alignment Tensor (A):
 -2.942464e-01  2.182962e-01 -9.304654e-01               -2.942464e-01  2.182962e-01 -9.304654e-01
 -8.263495e-01 -5.472435e-01  1.329326e-01               -8.263495e-01 -5.472435e-01  1.329326e-01
 -4.801724e-01  8.080045e-01  3.414134e-01               -4.801724e-01  8.080045e-01  3.414134e-01
```

```
Eigenvalues of Saupe Tensor S(xx), S(yy), S(zz):        Eigenvalues of Alignment Tensor A(xx), A(yy), A(zz):
 -1.124911e-04 -5.944450e-04  7.069362e-04               -7.499409e-05 -3.962967e-04  4.712908e-04

Alignment Tensor Irreducible Representation (A0, A1R, A1I, A2R, A2I):
 -5.257433e-04 -4.469873e-04  3.239897e-04  5.283285e-04 -5.666899e-05

Tensor Properties: *)
A(axial)      =   7.069362e-04         # alignment tensor axial component   = 3/2*A(zz) = S(zz)
A(rhombic)    =   3.213026e-04         # alignment tensor rhombic component = A(xx) - A(yy) = 2/3*(S(xx) - S(yy))
A(rhombicity) =   4.545002e-01         # alignment tensor rhombicity        = A(rhombic) / A(axial)
A(asymmetry)  =   6.817502e-01         # alignment tensor asymmetry         = 3/2*A(rhombicity) = (A(xx) - A(yy))/A(zz) = (S(xx) - S(yy))/S(zz)
GDO           =   7.597271e-04         # generalized degree of order        = sqrt(3/2)*|A(xx),A(yy),A(zz)| = sqrt(2/3)*|S(xx),S(yy),S(zz)|
----------
*) F. Kramer, M.V. Deshmukh, H. Kessler and S.J. Glaser, Concepts Magn. Res. A, 2004, 21A, 21-40.

--------------------------------------------------------------------------------
Results for Multi-Parameter SVD Fit of Calculated and Experimental Data:
           D(calc)[HZ]     +/- Error    D(exp)[HZ]     +/- Error  Rel. Weights           D(exp)-D(calc)       Normalized Weights    Atom Labels
D[01] =       1.751610             -      1.650000      0.500000      1.000000    r[01] =      -0.101610    w[01] =       0.066667    C2-H2
D[02] =      12.498203             -     11.900000      0.500000      1.000000    r[02] =      -0.598203    w[02] =       0.066667    C5-H5
D[03] =      12.174524             -     11.400000      0.500000      1.000000    r[03] =      -0.774524    w[03] =       0.066667    C7-H7A
D[04] =       5.165320             -      5.000000      0.500000      1.000000    r[04] =      -0.165320    w[04] =       0.066667    C7-H7B
D[05] =      -3.700884             -     -3.200000      0.500000      1.000000    r[05] =       0.500884    w[05] =       0.066667    C9-H9A|H9B|H9C
             -8.752962             -             -             -             -                          -                          -    | [3av] C9-H9A
             -7.786741             -             -             -             -                          -                          -    | [3av] C9-H9B
              5.437051             -             -             -             -                          -                          -    | [3av] C9-H9C
D[06] =       9.723606             -      9.750000      0.500000      1.000000    r[06] =       0.026394    w[06] =       0.066667    C10-H10
D[07] =      10.406748             -     11.050000      0.500000      1.000000    r[07] =       0.643252    w[07] =       0.066667    C13-H13A
D[08] =       3.776474             -      4.550000      0.500000      1.000000    r[08] =       0.773526    w[08] =       0.066667    C13-H13B
D[09] =      -2.764961             -     -2.700000      0.500000      1.000000    r[09] =       0.064961    w[09] =       0.066667    C15-H15A|H15B|H15C
             -5.561496             -             -             -             -                          -                          -    | [3av] C15-H15A
             -5.462282             -             -             -             -                          -                          -    | [3av] C15-H15B
              2.728895             -             -             -             -                          -                          -    | [3av] C15-H15C
D[10] =       8.327979             -      9.450000      0.500000      1.000000    r[10] =       1.122021    w[10] =       0.066667    C16-H16
D[11] =       9.688765             -      9.250000      0.500000      1.000000    r[11] =      -0.438765    w[11] =       0.066667    C17-H17A
D[12] =       7.387822             -      7.300000      0.500000      1.000000    r[12] =      -0.087822    w[12] =       0.066667    C17-H17B
D[13] =      11.490766             -     12.400000      0.500000      1.000000    r[13] =       0.909234    w[13] =       0.066667    C18-H18A
D[14] =       6.479786             -      5.050000      0.500000      1.000000    r[14] =      -1.429786    w[14] =       0.066667    C19-H19
D[15] =       5.200046             -      6.060000      0.500000      1.000000    r[15] =       0.859954    w[15] =       0.066667    C20-H20

Results for Multi-Parameter Fit of Calculated and Experimental Data:
rank  =              5                 # rank of cosine matrix (check input if rank < 5)
cond  =   1.542845e+01                 # condition number of cosine matrix (check input and singular values if very large)
aic   =      39.331925                 # information criterion (AIC) for 5 degrees of freedom
qfac  =       0.085683                 # weighted Q-Factor as defined by Cornilescu
r^2   =       0.978844                 # coefficient of determination r^2 = 1 - chi^2 / (weighted sum of squares)

(|D|) =       7.369166      7.380667   # mean absolute (calc./exp.) parameter D[i]
|D|min=       1.751610      1.650000   # min. absolute (calc./exp.) parameter D[i]
|D|max=      12.498203     12.400000   # max. absolute (calc./exp.) parameter D[i]
Drange=      -3.700884     12.498203   # min. and max. (calc.)      parameter D[i]
Drange=      -3.200000     12.400000   # min. and max. (exp.)       parameter D[i]
```

```
Results for Linear Regression of Calculated and Experimental Data (N=15):
c(b)  =      -0.111047 +/-  0.326527   # linear regression intercept and error
c(m)  =       1.003655 +/-  0.040015   # linear regression slope and error
rmsd  =       0.699189                 # unweighted total root-mean-square deviation
chisq =       0.488865                 # weighted total sum of squared residuals
maerr =       0.566417                 # weighted total mean absolute error (sum of weights = 1.000)
R     =       0.989825                 # weighted Pearson correlation coefficient R
R^2   =       0.979754                 # weighted Pearson correlation coefficient R^2

E(RDC)=       3.666491                 # E(RDC)=1/2*K*(sum of weighted deviations (D(exp)-D(calc))^2), K=1.000
```

3.1.27 (-)-Perilla acid (S191) @ 310K

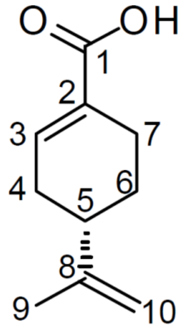

(-)-Perilla acid

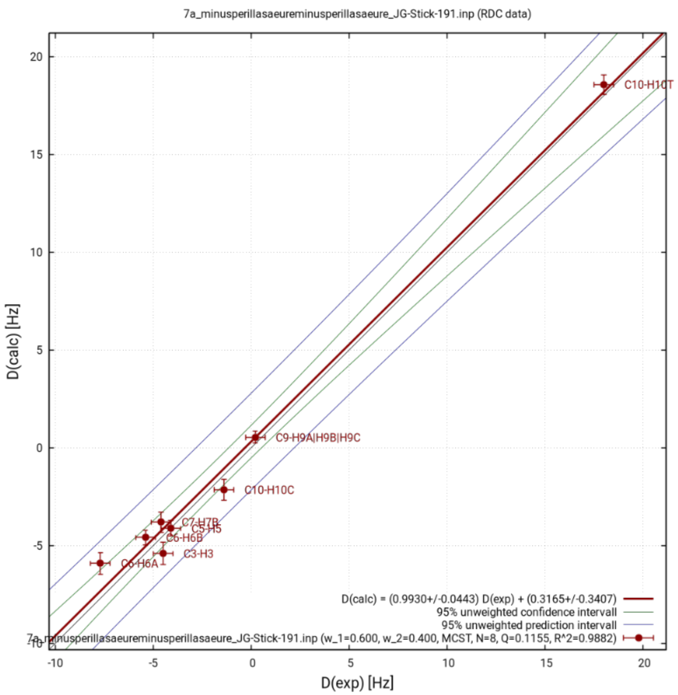

Multi-Conformer-Single-Tensor-Fit

```
================================================================================
info : Start Analysis for Structure ''
in file '7a_minusperillasaeureminusperillasaeure_JG-Stick-191.inp'
================================================================================

--------------------------------------------------------------------------------
info : Start Scan of Populations for 2/2 Conformers (nsteps = 20)
--------------------------------------------------------------------------------
info : generated and analyzed 21 populations of 2/2 conformers.
info : continue with best-fit populations.
--------------------------------------------------------------------------------

--------------------------------------------------------------------------------
info : Start Gradient-Descent Optimization of Populations for 2/2 Conformers
--------------------------------------------------------------------------------
info : relative weight of used conformers: w(1)=0.6000 w(2)=0.4000 | iteration   1
| q = 1.154995e-01 e = 3.152743e+00 de =  3.15274e+00 step = 5.000000e-03
info : conformer populations converged after 1/1000 steps (dxmax = 0.000e+00,
q = 1.155e-01, dq = 0.000e+00, e = 3.153e+00, de = 0.000e+00).
info : continue with optimized best-fit populations.
--------------------------------------------------------------------------------

--------------------------------------------------------------------------------
info : Start Multi-Conformer Single-Tensor (MCST) Fit with 2/2 Conformers and 8 RDCs
info : File: '7a_minusperillasaeureminusperillasaeure_JG-Stick-191.inp', Title: ''
--------------------------------------------------------------------------------
info : relative weight of conformers: w(1) = 0.6000; w(2) = 0.4000

--------------------------------------------------------------------------------
Structure File: '7a_minusperillasaeureminusperillasaeure_JG-Stick-191.inp' (Title: '')
--------------------------------------------------------------------------------
Structure with 2 Conformer(s) and 8 RDCs:
File '7a_minusperillasaeureminusperillasaeure_JG-Stick-191.inp'
            60.00 % :            Conformer 1 with 26 Atoms
            40.00 % :            Conformer 2 with 26 Atoms
--------------------------------------------------------------------------------

--------------------------------------------------------------------------------
SVD Best-Fit Saupe Vector S(zz), S(xx-yy), S(xy), S(xz), S(yz):
  2.697564e-04 -1.335762e-03  4.535128e-05 -4.300658e-06 -1.596060e-04
Saupe Tensor (S):                                        Alignment Tensor (A):
 -8.027592e-04  4.535128e-05 -4.300658e-06               -5.351728e-04  3.023419e-05 -2.867105e-06
  4.535128e-05  5.330028e-04 -1.596060e-04                3.023419e-05  3.553352e-04 -1.064040e-04
 -4.300658e-06 -1.596060e-04  2.697564e-04               -2.867105e-06 -1.064040e-04  1.798376e-04
Trace of Saupe Tensor:       -5.421011e-20               Trace of Alignment Tensor:   -3.614007e-20
Eigenvectors of Saupe Tensor (S):                        Eigenvectors of Alignment Tensor (A):
  1.541960e-02 -3.034159e-02  9.994206e-01                1.541960e-02 -3.034159e-02  9.994206e-01
  4.249809e-01 -9.045629e-01 -3.401862e-02                4.249809e-01 -9.045629e-01 -3.401862e-02
  9.050710e-01  4.252592e-01 -1.053401e-03                9.050710e-01  4.252592e-01 -1.053401e-03
```

```
Eigenvalues of Saupe Tensor S(xx), S(yy), S(zz):        Eigenvalues of Alignment Tensor A(xx), A(yy), A(zz):
  1.947393e-04  6.095590e-04 -8.042983e-04                1.298262e-04  4.063727e-04 -5.361989e-04


Alignment Tensor Irreducible Representation (A0, A1R, A1I, A2R, A2I):
  4.276532e-04 -5.566846e-06 -2.065968e-04 -8.645167e-04  5.870349e-05

Tensor Properties: *)
A(axial)      =  -8.042983e-04         # alignment tensor axial component   = 3/2*A(zz) = S(zz)
A(rhombic)    =  -2.765465e-04         # alignment tensor rhombic component = A(xx) - A(yy) = 2/3*(S(xx) - S(yy))
A(rhombicity) =   3.438357e-01         # alignment tensor rhombicity        = A(rhombic) / A(axial)
A(asymmetry)  =   5.157535e-01         # alignment tensor asymmetry         = 3/2*A(rhombicity) = (A(xx) - A(yy))/A(zz) = (S(xx) - S(yy))/S(zz)
GDO           =   8.391986e-04         # generalized degree of order        = sqrt(3/2)*|A(xx),A(yy),A(zz)| = sqrt(2/3)*|S(xx),S(yy),S(zz)|
----------
*) F. Kramer, M.V. Deshmukh, H. Kessler and S.J. Glaser, Concepts Magn. Res. A, 2004, 21A, 21-40.

--------------------------------------------------------------------------------
Results for Multi-Parameter SVD Fit of Calculated and Experimental Data:
           D(calc)[HZ]     +/- Error    D(exp)[HZ]     +/- Error  Rel. Weights            D(exp)-D(calc)        Normalized Weights | RDC Conformers  |    Atom Labels
D[01] =       0.528546             -      0.200000      0.500000      1.000000    r[01] =      -0.328546    w[01] =       0.125000 |   0.439   0.663 |    C9-H9A|H9B|H9C
D[02] =      -5.910367             -     -7.700000      0.500000      1.000000    r[02] =      -1.789633    w[02] =       0.125000 |  -5.056  -7.192 |    C6-H6A
D[03] =      -4.594403             -     -5.400000      0.500000      1.000000    r[03] =      -0.805597    w[03] =       0.125000 |  -4.492  -4.748 |    C6-H6B
D[04] =      -4.129984             -     -4.100000      0.500000      1.000000    r[04] =       0.029984    w[04] =       0.125000 |  -4.033  -4.275 |    C5-H5
D[05] =      18.565050             -     18.000000      0.500000      1.000000    r[05] =      -0.565050    w[05] =       0.125000 |  18.615  18.490 |    C10-H10T
D[06] =      -2.150502             -     -1.400000      0.500000      1.000000    r[06] =       0.750502    w[06] =       0.125000 |  -1.856  -2.593 |    C10-H10C
D[07] =      -5.408261             -     -4.500000      0.500000      1.000000    r[07] =       0.908261    w[07] =       0.125000 |  -4.800  -6.320 |    C3-H3
D[08] =      -3.801628             -     -4.600000      0.500000      1.000000    r[08] =      -0.798372    w[08] =       0.125000 |  -4.859  -2.215 |    C7-H7B

Results for Multi-Parameter Fit of Calculated and Experimental Data:
rank  =              5                 # rank of cosine matrix (check input if rank < 5)
cond  =   8.058356e+00                 # condition number of cosine matrix (check input and singular values if very large)
aic   =      37.221944                 # information criterion (AIC) for 6 degrees of freedom
qfac  =       0.115500                 # weighted Q-Factor as defined by Cornilescu
r^2   =       0.986334                 # coefficient of determination r^2 = 1 - chi^2 / (weighted sum of squares)

(|D|) =       5.636093      5.737500   # mean absolute (calc./exp.) parameter D[i]
|D|min=       0.528546      0.200000   # min. absolute (calc./exp.) parameter D[i]
|D|max=      18.565050     18.000000   # max. absolute (calc./exp.) parameter D[i]
Drange=      -5.910367     18.565050   # min. and max. (calc.)      parameter D[i]
Drange=      -7.700000     18.000000   # min. and max. (exp.)       parameter D[i]

Results for Linear Regression of Calculated and Experimental Data (N=8):
c(b)  =       0.316519 +/-  0.340710   # linear regression intercept and error
c(m)  =       0.993021 +/-  0.044325   # linear regression slope and error
rmsd  =       0.887798                 # unweighted total root-mean-square deviation
chisq =       0.788186                 # weighted total sum of squared residuals
maerr =       0.746993                 # weighted total mean absolute error (sum of weights = 1.000)
R     =       0.994076                 # weighted Pearson correlation coefficient R
R^2   =       0.988187                 # weighted Pearson correlation coefficient R^2

E(RDC)=       3.152743                 # E(RDC)=1/2*K*(sum of weighted deviations (D(exp)-D(calc))^2), K=1.000
```

3.1.28 (+)-IPC (S282): in dichloromethane @ 300K

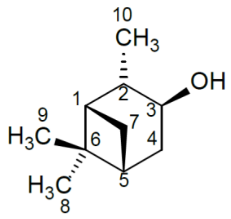

(+)-IPC

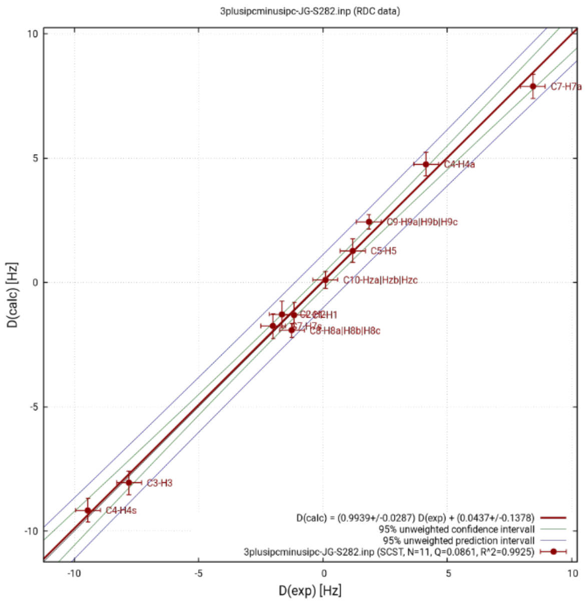


```
================================================================================
info : Start Analysis for Structure '' in file '3plusipcminusipc-JG-S282.inp'
================================================================================

--------------------------------------------------------------------------------
info : Start Single-Conformer Single-Tensor (SCST) Fit with 11 RDCs
info : File: '3plusipcminusipc-JG-S282.inp', Title: ''
--------------------------------------------------------------------------------

--------------------------------------------------------------------------------
SVD Best-Fit Saupe Vector S(zz), S(xx-yy), S(xy), S(xz), S(yz):
  3.274463e-04 -2.978840e-04 -2.166685e-04  1.722128e-04 -1.515353e-04
Saupe Tensor (S):                                        Alignment Tensor (A):
 -3.126652e-04 -2.166685e-04  1.722128e-04                -2.084434e-04 -1.444456e-04  1.148085e-04
 -2.166685e-04 -1.478115e-05 -1.515353e-04                -1.444456e-04 -9.854102e-06 -1.010236e-04
  1.722128e-04 -1.515353e-04  3.274463e-04                 1.148085e-04 -1.010236e-04  2.182975e-04
Trace of Saupe Tensor:        0.000000e+00               Trace of Alignment Tensor:    0.000000e+00
Eigenvectors of Saupe Tensor (S):                        Eigenvectors of Alignment Tensor (A):
  3.105024e-01 -8.999142e-01 -3.061741e-01                 3.105024e-01 -8.999142e-01 -3.061741e-01
 -8.088849e-01 -4.193212e-01  4.121589e-01                -8.088849e-01 -4.193212e-01  4.121589e-01
 -4.992930e-01  1.196833e-01 -8.581273e-01                -4.992930e-01  1.196833e-01 -8.581273e-01
Eigenvalues of Saupe Tensor S(xx), S(yy), S(zz):         Eigenvalues of Alignment Tensor A(xx), A(yy), A(zz):
 -2.514659e-05 -4.365266e-04  4.616732e-04                -1.676439e-05 -2.910177e-04  3.077821e-04

Alignment Tensor Irreducible Representation (A0, A1R, A1I, A2R, A2I):
  5.191108e-04  2.229152e-04 -1.961500e-04 -1.927931e-04 -2.804594e-04

Tensor Properties: *)
A(axial)      =   4.616732e-04         # alignment tensor axial component   = 3/2*A(zz) = S(zz)
A(rhombic)    =   2.742533e-04         # alignment tensor rhombic component = A(xx) - A(yy) = 2/3*(S(xx) - S(yy))
A(rhombicity) =   5.940422e-01         # alignment tensor rhombicity        = A(rhombic) / A(axial)
A(asymmetry)  =   8.910633e-01         # alignment tensor asymmetry         = 3/2*A(rhombicity) = (A(xx) - A(yy))/A(zz) = (S(xx) - S(yy))/S(zz)
GDO           =   5.191852e-04         # generalized degree of order        = sqrt(3/2)*|A(xx),A(yy),A(zz)| = sqrt(2/3)*|S(xx),S(yy),S(zz)|
----------
*) F. Kramer, M.V. Deshmukh, H. Kessler and S.J. Glaser, Concepts Magn. Res. A, 2004, 21A, 21-40.
```

```
-------------------------------------------------------------------------------
Results for Multi-Parameter SVD Fit of Calculated and Experimental Data:
           D(calc)[HZ]     +/- Error    D(exp)[HZ]     +/- Error  Rel. Weights            D(exp)-D(calc)       Normalized Weights    Atom Labels
D[01] =      -1.300396             -     -1.150000      0.500000      1.000000    r[01] =       0.150396    w[01] =       0.090909    C1-H1
D[02] =      -1.278153             -     -1.650000      0.500000      1.000000    r[02] =      -0.371847    w[02] =       0.090909    C2-H2
D[03] =      -8.057748             -     -7.800000      0.500000      1.000000    r[03] =       0.257748    w[03] =       0.090909    C3-H3
D[04] =      -9.161451             -     -9.450000      0.500000      1.000000    r[04] =      -0.288549    w[04] =       0.090909    C4-H4s
D[05] =       4.756720             -      4.150000      0.500000      1.000000    r[05] =      -0.606720    w[05] =       0.090909    C4-H4a
D[06] =       1.272840             -      1.200000      0.500000      1.000000    r[06] =      -0.072840    w[06] =       0.090909    C5-H5
D[07] =      -1.755243             -     -2.000000      0.500000      1.000000    r[07] =      -0.244757    w[07] =       0.090909    C7-H7s
D[08] =       7.884572             -      8.450000      0.500000      1.000000    r[08] =       0.565428    w[08] =       0.090909    C7-H7a
D[09] =      -1.931227             -     -1.250000      0.500000      1.000000    r[09] =       0.681227    w[09] =       0.090909    C8-H8a|H8b|H8c
              4.273186             -             -             -             -                         -                          -    | [3av] C8-H8a
             -1.121331             -             -             -             -                         -                          -    | [3av] C8-H8b
             -8.945535             -             -             -             -                         -                          -    | [3av] C8-H8c
D[10] =       2.439804             -      1.850000      0.500000      1.000000    r[10] =      -0.589804    w[10] =       0.090909    C9-H9a|H9b|H9c
             -0.829320             -             -             -             -                         -                          -    | [3av] C9-H9a
              4.286092             -             -             -             -                         -                          -    | [3av] C9-H9b
              3.862639             -             -             -             -                         -                          -    | [3av] C9-H9c
D[11] =       0.107465             -      0.100000      0.500000      1.000000    r[11] =      -0.007465    w[11] =       0.090909    C10-Hza|Hzb|Hzc
             -4.407046             -             -             -             -                         -                          -    | [3av] C10-Hza
             -0.948565             -             -             -             -                         -                          -    | [3av] C10-Hzb
              5.678008             -             -             -             -                         -                          -    | [3av] C10-Hzc

Results for Multi-Parameter Fit of Calculated and Experimental Data:
rank  =              5                # rank of cosine matrix (check input if rank < 5)
cond  =   2.147425e+00                # condition number of cosine matrix (check input and singular values if very large)
aic   =      17.502432                # information criterion (AIC) for 5 degrees of freedom
qfac  =       0.086059                # weighted Q-Factor as defined by Cornilescu
r^2   =       0.992439                # coefficient of determination r^2 = 1 - chi^2 / (weighted sum of squares)

(|D|) =       3.631420      3.550000   # mean absolute (calc./exp.) parameter D[i]
|D|min=       0.107465      0.100000   # min. absolute (calc./exp.) parameter D[i]
|D|max=       9.161451      9.450000   # max. absolute (calc./exp.) parameter D[i]
Drange=      -9.161451      7.884572   # min. and max. (calc.)      parameter D[i]
Drange=      -9.450000      8.450000   # min. and max. (exp.)       parameter D[i]

Results for Linear Regression of Calculated and Experimental Data (N=11):
c(b)  =       0.043737 +/-  0.137788   # linear regression intercept and error
c(m)  =       0.993898 +/-  0.028717   # linear regression slope and error
rmsd  =       0.412928                 # unweighted total root-mean-square deviation
chisq =       0.170510                 # weighted total sum of squared residuals
maerr =       0.348798                 # weighted total mean absolute error (sum of weights = 1.000)
R     =       0.996264                 # weighted Pearson correlation coefficient R
R^2   =       0.992543                 # weighted Pearson correlation coefficient R^2

E(RDC)=       0.937804                 # E(RDC)=1/2*K*(sum of weighted deviations (D(exp)-D(calc))^2), K=1.000

-------------------------------------------------------------------------------
```

3.1.29 (+)-IPC in Stick S284: @ 300K in THF

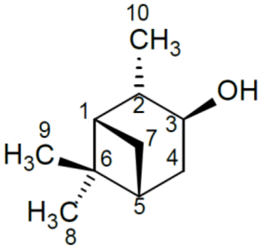

(+)-IPC

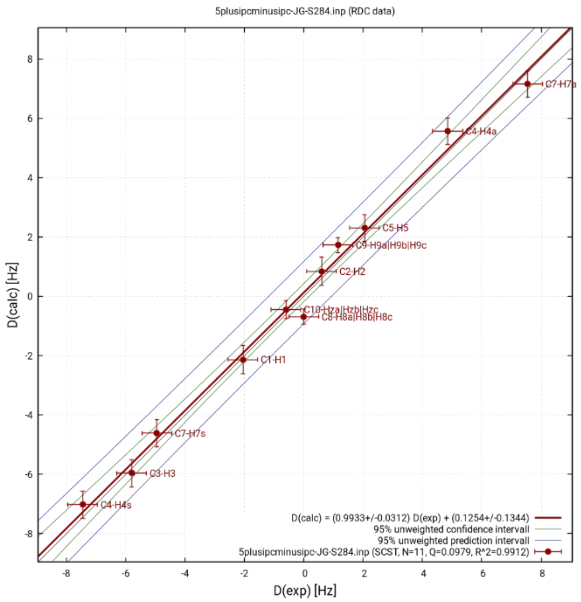


```
===============================================================================
info : Start Analysis for Structure '' in file '5plusipcminusipc-JG-S284.inp'
===============================================================================

-------------------------------------------------------------------------------
info : Start Single-Conformer Single-Tensor (SCST) Fit with 11 RDCs
info : File: '5plusipcminusipc-JG-S284.inp', Title: ''
-------------------------------------------------------------------------------

-------------------------------------------------------------------------------
SVD Best-Fit Saupe Vector S(zz), S(xx-yy), S(xy), S(xz), S(yz):
  2.832907e-04 -3.204682e-04 -1.031561e-04  7.912356e-05 -2.159915e-04
Saupe Tensor (S):                                       Alignment Tensor (A):
 -3.018794e-04 -1.031561e-04  7.912356e-05               -2.012530e-04 -6.877074e-05  5.274904e-05
 -1.031561e-04  1.858871e-05 -2.159915e-04               -6.877074e-05  1.239247e-05 -1.439943e-04
  7.912356e-05 -2.159915e-04  2.832907e-04                5.274904e-05 -1.439943e-04  1.888605e-04
Trace of Saupe Tensor:        0.000000e+00              Trace of Alignment Tensor:    0.000000e+00
Eigenvectors of Saupe Tensor (S):                       Eigenvectors of Alignment Tensor (A):
 -2.095005e-01 -9.640542e-01  1.634290e-01               -2.095005e-01 -9.640542e-01  1.634290e-01
  8.273189e-01 -2.638539e-01 -4.959079e-01                8.273189e-01 -2.638539e-01 -4.959079e-01
  5.212034e-01  3.131497e-02  8.528577e-01                5.212034e-01  3.131497e-02  8.528577e-01
Eigenvalues of Saupe Tensor S(xx), S(yy), S(zz):        Eigenvalues of Alignment Tensor A(xx), A(yy), A(zz):
 -9.136195e-05 -3.326826e-04  4.240445e-04               -6.090797e-05 -2.217884e-04  2.826964e-04

Alignment Tensor Irreducible Representation (A0, A1R, A1I, A2R, A2I):
  4.491096e-04  1.024189e-04 -2.795831e-04 -2.074098e-04 -1.335271e-04

Tensor Properties: *)
A(axial)      =   4.240445e-04         # alignment tensor axial component   = 3/2*A(zz) = S(zz)
A(rhombic)    =   1.608804e-04         # alignment tensor rhombic component = A(xx) - A(yy) = 2/3*(S(xx) - S(yy))
A(rhombicity) =   3.793951e-01         # alignment tensor rhombicity        = A(rhombic) / A(axial)
A(asymmetry)  =   5.690927e-01         # alignment tensor asymmetry         = 3/2*A(rhombicity) = (A(xx) - A(yy))/A(zz) = (S(xx) - S(yy))/S(zz)
GDO           =   4.463470e-04         # generalized degree of order        = sqrt(3/2)*|A(xx),A(yy),A(zz)| = sqrt(2/3)*|S(xx),S(yy),S(zz)|
----------
*) F. Kramer, M.V. Deshmukh, H. Kessler and S.J. Glaser, Concepts Magn. Res. A, 2004, 21A, 21-40.
```

```
-------------------------------------------------------------------------------
Results for Multi-Parameter SVD Fit of Calculated and Experimental Data:
           D(calc)[HZ]     +/- Error    D(exp)[HZ]     +/- Error  Rel. Weights           D(exp)-D(calc)       Normalized Weights    Atom Labels
D[01] =      -2.130493             -     -2.050000      0.500000      1.000000    r[01] =       0.080493    w[01] =       0.090909    C1-H1
D[02] =       0.844529             -      0.600000      0.500000      1.000000    r[02] =      -0.244529    w[02] =       0.090909    C2-H2
D[03] =      -5.960824             -     -5.800000      0.500000      1.000000    r[03] =       0.160824    w[03] =       0.090909    C3-H3
D[04] =      -7.021862             -     -7.450000      0.500000      1.000000    r[04] =      -0.428138    w[04] =       0.090909    C4-H4s
D[05] =       5.569390             -      4.850000      0.500000      1.000000    r[05] =      -0.719390    w[05] =       0.090909    C4-H4a
D[06] =       2.314790             -      2.050000      0.500000      1.000000    r[06] =      -0.264790    w[06] =       0.090909    C5-H5
D[07] =      -4.614944             -     -4.950000      0.500000      1.000000    r[07] =      -0.335056    w[07] =       0.090909    C7-H7s
D[08] =       7.157637             -      7.550000      0.500000      1.000000    r[08] =       0.392363    w[08] =       0.090909    C7-H7a
D[09] =      -0.685346             -      0.000000      0.500000      1.000000    r[09] =       0.685346    w[09] =       0.090909    C8-H8a|H8b|H8c
              4.493174             -             -             -             -                          -                          -    | [3av] C8-H8a
              0.676829             -             -             -             -                          -                          -    | [3av] C8-H8b
             -7.226041             -             -             -             -                          -                          -    | [3av] C8-H8c
D[10] =       1.730627             -      1.150000      0.500000      1.000000    r[10] =      -0.580627    w[10] =       0.090909    C9-H9a|H9b|H9c
              0.901309             -             -             -             -                          -                          -    | [3av] C9-H9a
              4.543509             -             -             -             -                          -                          -    | [3av] C9-H9b
             -0.252937             -             -             -             -                          -                          -    | [3av] C9-H9c
D[11] =      -0.442453             -     -0.600000      0.500000      1.000000    r[11] =      -0.157547    w[11] =       0.090909    C10-Hza|Hzb|Hzc
             -4.516682             -             -             -             -                          -                          -    | [3av] C10-Hza
              1.178777             -             -             -             -                          -                          -    | [3av] C10-Hzb
              2.010545             -             -             -             -                          -                          -    | [3av] C10-Hzc

Results for Multi-Parameter Fit of Calculated and Experimental Data:
rank  =              5                # rank of cosine matrix (check input if rank < 5)
cond  =   2.147425e+00                # condition number of cosine matrix (check input and singular values if very large)
aic   =      17.843740                # information criterion (AIC) for 5 degrees of freedom
qfac  =       0.097895                # weighted Q-Factor as defined by Cornilescu
r^2   =       0.990324                # coefficient of determination r^2 = 1 - chi^2 / (weighted sum of squares)

(|D|) =       3.497536      3.368182   # mean absolute (calc./exp.) parameter D[i]
|D|min=       0.442453      0.000000   # min. absolute (calc./exp.) parameter D[i]
|D|max=       7.157637      7.550000   # max. absolute (calc./exp.) parameter D[i]
Drange=      -7.021862      7.157637   # min. and max. (calc.)      parameter D[i]
Drange=      -7.450000      7.550000   # min. and max. (exp.)       parameter D[i]

Results for Linear Regression of Calculated and Experimental Data (N=11):
c(b)  =       0.125431 +/-  0.134387   # linear regression intercept and error
c(m)  =       0.993267 +/-  0.031159   # linear regression slope and error
rmsd  =       0.422217                # unweighted total root-mean-square deviation
chisq =       0.178267                # weighted total sum of squared residuals
maerr =       0.368100                # weighted total mean absolute error (sum of weights = 1.000)
R     =       0.995601                # weighted Pearson correlation coefficient R
R^2   =       0.991221                # weighted Pearson correlation coefficient R^2

E(RDC)=       0.980467                # E(RDC)=1/2*K*(sum of weighted deviations (D(exp)-D(calc))^2), K=1.000
```

3.1.30 (-)-IPC in Stick S285 @ 300K in THF

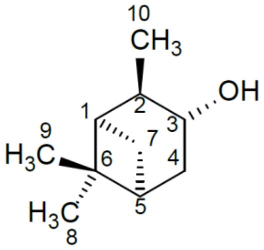

(-)-IPC

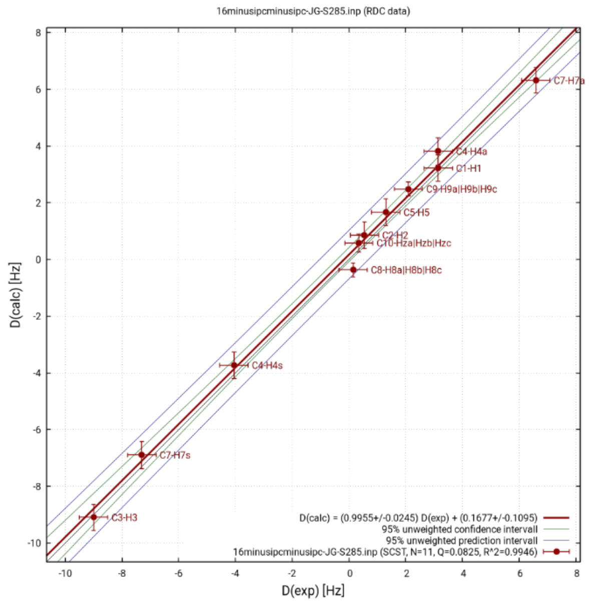


```
================================================================================
info : Start Analysis for Structure '' in file '16minusipcminusipc-JG-S285.inp'
================================================================================

--------------------------------------------------------------------------------
info : Start Single-Conformer Single-Tensor (SCST) Fit with 11 RDCs
info : File: '16minusipcminusipc-JG-S285.inp', Title: ''
--------------------------------------------------------------------------------

--------------------------------------------------------------------------------
SVD Best-Fit Saupe Vector S(zz), S(xx-yy), S(xy), S(xz), S(yz):
  4.578139e-05 -4.614795e-04 -1.645644e-04  1.448781e-04 -2.707260e-04
Saupe Tensor (S):                                        Alignment Tensor (A):
 -2.536304e-04 -1.645644e-04  1.448781e-04                -1.690869e-04 -1.097096e-04  9.658540e-05
 -1.645644e-04  2.078490e-04 -2.707260e-04                -1.097096e-04  1.385660e-04 -1.804840e-04
  1.448781e-04 -2.707260e-04  4.578139e-05                 9.658540e-05 -1.804840e-04  3.052092e-05
Trace of Saupe Tensor:         0.000000e+00              Trace of Alignment Tensor:     0.000000e+00
Eigenvectors of Saupe Tensor (S):                        Eigenvectors of Alignment Tensor (A):
  9.675554e-02  9.528992e-01 -2.874394e-01                 9.675554e-02  9.528992e-01 -2.874394e-01
  6.240546e-01  1.668968e-01  7.633488e-01                 6.240546e-01  1.668968e-01  7.633488e-01
  7.753672e-01 -2.532361e-01 -5.785129e-01                 7.753672e-01 -2.532361e-01 -5.785129e-01
Eigenvalues of Saupe Tensor S(xx), S(yy), S(zz):         Eigenvalues of Alignment Tensor A(xx), A(yy), A(zz):
 -1.540337e-04 -3.209551e-04  4.749888e-04                -1.026891e-04 -2.139701e-04  3.166592e-04

Alignment Tensor Irreducible Representation (A0, A1R, A1I, A2R, A2I):
  7.257865e-05  1.875327e-04 -3.504324e-04 -2.986735e-04 -2.130150e-04

Tensor Properties: *)
A(axial)      =   4.749888e-04         # alignment tensor axial component   = 3/2*A(zz) = S(zz)
A(rhombic)    =   1.112809e-04         # alignment tensor rhombic component = A(xx) - A(yy) = 2/3*(S(xx) - S(yy))
A(rhombicity) =   2.342812e-01         # alignment tensor rhombicity        = A(rhombic) / A(axial)
A(asymmetry)  =   3.514218e-01         # alignment tensor asymmetry         = 3/2*A(rhombicity) = (A(xx) - A(yy))/A(zz) = (S(xx) - S(yy))/S(zz)
GDO           =   4.846668e-04         # generalized degree of order        = sqrt(3/2)*|A(xx),A(yy),A(zz)| = sqrt(2/3)*|S(xx),S(yy),S(zz)|
----------
*) F. Kramer, M.V. Deshmukh, H. Kessler and S.J. Glaser, Concepts Magn. Res. A, 2004, 21A, 21-40.
```

```
-------------------------------------------------------------------------------
Results for Multi-Parameter SVD Fit of Calculated and Experimental Data:
           D(calc)[HZ]     +/- Error    D(exp)[HZ]     +/- Error  Rel. Weights            D(exp)-D(calc)       Normalized Weights    Atom Labels
D[01] =       3.219134             -      3.150000      0.500000      1.000000    r[01] =      -0.069134    w[01] =       0.090909    C1-H1
D[02] =       0.856596             -      0.550000      0.500000      1.000000    r[02] =      -0.306596    w[02] =       0.090909    C2-H2
D[03] =      -9.094658             -     -9.000000      0.500000      1.000000    r[03] =       0.094658    w[03] =       0.090909    C3-H3
D[04] =      -3.730820             -     -4.050000      0.500000      1.000000    r[04] =      -0.319180    w[04] =       0.090909    C4-H4s
D[05] =       3.815476             -      3.150000      0.500000      1.000000    r[05] =      -0.665476    w[05] =       0.090909    C4-H4a
D[06] =       1.664889             -      1.300000      0.500000      1.000000    r[06] =      -0.364889    w[06] =       0.090909    C5-H5
D[07] =      -6.894485             -     -7.300000      0.500000      1.000000    r[07] =      -0.405515    w[07] =       0.090909    C7-H7s
D[08] =       6.315795             -      6.600000      0.500000      1.000000    r[08] =       0.284205    w[08] =       0.090909    C7-H7a
D[09] =      -0.365472             -      0.150000      0.500000      1.000000    r[09] =       0.515472    w[09] =       0.090909    C8-H8a|H8b|H8c
              5.625689             -             -             -             -                       -                        -    | [3av] C8-H8a
              3.385977             -             -             -             -                       -                        -    | [3av] C8-H8b
            -10.108082             -             -             -             -                       -                        -    | [3av] C8-H8c
D[10] =       2.487413             -      2.100000      0.500000      1.000000    r[10] =      -0.387413    w[10] =       0.090909    C9-H9a|H9b|H9c
              3.469809             -             -             -             -                       -                        -    | [3av] C9-H9a
              5.558276             -             -             -             -                       -                        -    | [3av] C9-H9b
             -1.565846             -             -             -             -                       -                        -    | [3av] C9-H9c
D[11] =       0.584278             -      0.350000      0.500000      1.000000    r[11] =      -0.234278    w[11] =       0.090909    C10-Hza|Hzb|Hzc
              1.289254             -             -             -             -                       -                        -    | [3av] C10-Hza
              0.570934             -             -             -             -                       -                        -    | [3av] C10-Hzb
             -0.107355             -             -             -             -                       -                        -    | [3av] C10-Hzc

Results for Multi-Parameter Fit of Calculated and Experimental Data:
rank  =              5                # rank of cosine matrix (check input if rank < 5)
cond  =   2.147425e+00                # condition number of cosine matrix (check input and singular values if very large)
aic   =      16.006078                # information criterion (AIC) for 5 degrees of freedom
qfac  =       0.082493                # weighted Q-Factor as defined by Cornilescu
r^2   =       0.993170                # coefficient of determination r^2 = 1 - chi^2 / (weighted sum of squares)

(|D|) =       3.548092      3.427273   # mean absolute (calc./exp.) parameter D[i]
|D|min=       0.365472      0.150000   # min. absolute (calc./exp.) parameter D[i]
|D|max=       9.094658      9.000000   # max. absolute (calc./exp.) parameter D[i]
Drange=      -9.094658      6.315795   # min. and max. (calc.)      parameter D[i]
Drange=      -9.000000      6.600000   # min. and max. (exp.)       parameter D[i]

Results for Linear Regression of Calculated and Experimental Data (N=11):
c(b)  =       0.167688 +/-  0.109523   # linear regression intercept and error
c(m)  =       0.995475 +/-  0.024454   # linear regression slope and error
rmsd  =       0.369461                 # unweighted total root-mean-square deviation
chisq =       0.136502                 # weighted total sum of squared residuals
maerr =       0.331529                 # weighted total mean absolute error (sum of weights = 1.000)
R     =       0.997295                 # weighted Pearson correlation coefficient R
R^2   =       0.994598                 # weighted Pearson correlation coefficient R^2

E(RDC)=       0.750760                 # E(RDC)=1/2*K*(sum of weighted deviations (D(exp)-D(calc))^2), K=1.000

-------------------------------------------------------------------------------
```

Literature

[1] H. E. Gottlieb, V. Kotlyar, A. Nudelman, *J. Org. Chem.* **1997**, *62*, 7512-7515.

[2] P. Lesot, P. Berdagué, A. Meddour, A. Kreiter, M. Noll, M. Reggelin, *ChemPlusChem* **2019**, *84*, 144-153.

[3] a) P. Berdagué, B. Gouilleux, M. Noll, S. Immel, M. Reggelin, P. Lesot, *Phys. Chem. Chem. Phys.* **2022**, *24*, 7338-7348; b) T. Julien, B. Gouilleux, B. Rousseau, M. Reggelin, P. Lesot, *Chemistry–Methods* **2025**, *n/a*, e2500011.

[4] K. K. L. Cheuk, J. W. Y. Lam, J. Chen, L. M. Lai, B. Z. Tang, *Macromolecules* **2003**, *36*, 5947-5959.

[5] N.-C. Meyer, A. Krupp, V. Schmidts, C. M. Thiele, M. Reggelin, *Angew. Chem. Int. Ed.* **2012**, *51*, 8334-8338.

[6] K. Okoshi, K. Sakajiri, J. Kumaki, E. Yashima, *Macromolecules* **2005**, *38*, 4061-4064.

[7] Y. Misumi, T. Masuda, *Macromolecules* **1998**, *31*, 7572-7573.

[8] P. Trigo-Mourino, C. Merle, M. R. M. Koos, B. Luy, R. R. Gil, *Chem. Eur. J.* **2013**, *19*, 7013-7019.

[9] A. Enthart, J. C. Freudenberger, J. Furrer, H. Kessler, B. Luy, *J. Magn. Reson.* **2008**, *192*, 314-322.

[10] a) S. Immel, M. Köck, M. Reggelin, *Chem. Eur. J.* **2018**, *24*, 13918-13930; b) ConArch+, https://www.chemie.tu-darmstadt.de/reggelin/der_arbeitskreis_reggelin/conarch_plus/index.en.jsp.

[11] M. J. Frisch, G. W. Trucks, H. B. Schlegel, G. E. Scuseria, M. A. Robb, J. R. Cheeseman, G. Scalmani, V. Barone, B. Mennucci, G. A. Petersson, H. Nakatsuji, M. Caricato, X. Li, H. P. Hratchian, A. F. Izmaylov, J. Bloino, G. Zheng, J. L. Sonnenberg, M. Hada, M. Ehara, K. Toyota, R. Fukuda, J. Hasegawa, M. Ishida, T. Nakajima, Y. Honda, O. Kitao, H. Nakai, T. Vreven, J. A. Montgomery, J. J. E. Peralta, F. Ogliaro, M. Bearpark, J. J. Heyd, E. Brothers, K. N. Kudin, V. N. Staroverov, R. Kobayashi, J. Normand, K. Raghavachari, A. Rendell, J. C. Burant, S. S. Iyengar, J. Tomasi, M. Cossi, N. Rega, J. M. Millam, M. Klene, J. E. Knox, J. B. Cross, V. Bakken, C. Adamo, J. Jaramillo, R. Gomperts, R. E. Stratmann, O. Yazyev, A. J. Austin, R. Cammi, C. Pomelli, J. W. Ochterski, R. L. Martin, K. Morokuma, V. G. Zakrzewski, G. A. Voth, P. Salvador, J. J. Dannenberg, S. Dapprich, A. D. Daniels, O. Farkas, J. B. Foresman, J. V. Ortiz, J. Cioslowski, D. J. Fox, *Wallingford CT* **2009**.

[12] S. Immel, M. Köck, M. Reggelin, *Chirality* **2019**, *31*, 384-400.

[13] F. Kramer, M. V. Deshmukh, H. Kessler, S. J. Glaser, *Concepts Magn. Reson., Part A* **2004**, *21A*, 10-21.
